# Time-dependent trade-offs among intravenous iron formulations for iron-deficiency anemia: a longitudinal systematic review and network meta-analysis

**DOI:** 10.1016/j.htct.2026.106435

**Published:** 2026-04-01

**Authors:** Francino Machado de Azevedo Filho, Mariana Millan Fachi, Layssa Andrade Oliveira, Haliton Alves de Oliveira, Rosa Camila Lucchetta

**Affiliations:** Hospital Alemão Oswaldo Cruz - Health Technology Assessment Unit (UATS), Brazil

**Keywords:** Anemia, Iron, Hypophosphatemia, Meta-analysis, Decision-making

## Abstract

Intravenous iron formulations differ in benefits and risks that evolve across clinically relevant timepoints. A comprehensive network meta-analysis of randomized trials compared different intravenous iron formulations with oral iron and with each other. In addition to overall estimates, results are presented by predefined timepoints (4, 8, 12 and 24 weeks) for hemoglobin, ferritin, transferrin saturation, serious adverse events, and hypophosphatemia. Over 4-8 weeks, ferric carboxymaltose more consistently increased iron stores (ferritin/transferrin saturation) compared with the alternatives at the cost of a higher risk of hypophosphatemia. In 12-24 weeks, differences in iron stores attenuated and safety considerations became the main driver of choice, with ferric derisomaltose and iron sucrose generally favored when mineral safety is prioritized (e.g., for chronic kidney disease and inflammatory bowel disease). Effects on hemoglobin were broadly comparable between ferric carboxymaltose and ferric derisomaltose across most timepoints. These findings support conditional, scenario-specific decisions rather than a single ‘best’ formulation: ferric carboxymaltose when rapid repletion is critical and monitoring for hypophosphatemia is feasible; ferric derisomaltose and iron sucrose when safety predominates, or longer-term maintenance is planned. This windowed presentation facilitates pragmatic evidence translation for clinical decision-making while maintaining transparency through standard network meta-analysis diagnostics and certainty assessments.

## Introduction

Iron-deficiency anemia (IDA) is a common and significant cause of global morbidity, affecting more than 1.9 billion people (∼27 % of the population) [1,2]. Common etiologies include gastrointestinal disorders, malignancies and inflammatory bowel disease (IBD) [3].

Diagnosis and monitoring consider hemoglobin (Hb), transferrin saturation (TSAT) and ferritin levels, which reflect both circulating and stored iron. Their combined interpretation aids differentiation of etiologies and treatment decisions [4].

Multiple intravenous (IV) iron formulations are available, each with distinct pharmacokinetic and tolerability profiles. Compared with oral iron, IV formulations provide faster and more complete repletion of iron stores, particularly in patients with inflammation, intolerance, or poor absorption [5, 6, 7].

However, the temporal dynamics of response and safety, critical for therapeutic planning, are rarely analyzed. This systematic review and network meta-analysis (NMA) compared IV formulations across clinically relevant decision time intervals (4, 8, 12, 24 weeks) to inform time-sensitive and context-specific clinical choices.

## Methods

### Study design and reporting

This systematic review was designed in accordance with the guidelines of the Cochrane Handbook and reported following the PRISMA 2020 and PRISMA-NMA guidelines (Supplement 1) [8,9]. The protocol was registered in PROSPERO (CRD420251065465).

### Research question and eligibility criteria

**PICO framework:** In adults with IDA (P), how do intravenous (IV) iron formulations (I), compared with other IV formulations or oral ferrous sulfate (C), affect hemoglobin, ferritin, transferrin saturation, hypophosphatemia, any adverse events, and serious adverse events (O) when summarized within pre-specified clinical decision time intervals at 4, 8, 12, and 24 weeks (T)?

Randomized controlled trials of adults with IDA were included in this study to compare iron dextran, sodium ferric gluconate complex (SFG), iron sucrose (ISC), ferumoxytol (FXM), ferric carboxymaltose (FCM) and ferric derisomaltose (FDI) (versus oral ferrous sulfate and with each other). The primary outcome investigated was the hemoglobin level; secondary outcomes included ferritin, TSAT, adverse events, serious adverse events, and hypophosphatemia. Trials reporting obstetric and surgical IDA were excluded.

### Data sources and search strategy

A systematic search was performed in February 2025 of PubMed/MEDLINE, the Cochrane Library, and Embase using structured descriptors to target the six IV iron formulations [10]. No language or date restrictions were applied (Supplement 2).

### Study selection and data extraction

Two reviewers independently screened and extracted data using the Rayyan platform [11]. Extracted variables included baseline characteristics, intervention details, and outcomes at all reported timepoints. Discrepancies were resolved by consensus. Calculations and imputations followed the guidelines of the Cochrane Handbook [9].

### Risk of bias and certainty of evidence

Risk of Bias Tool (RoB 2) [12] was used to assess the risk of bias at the outcome level. Certainty for NMA estimates used CINeMA [13,14] (overall and subgroups) and GRADE (high/moderate/low/very low) was used for pairwise analyses [15]. Downgrading reasons are detailed in Supplement 3.

### Data synthesis and analysis

Mean differences and risk ratios (RRs) were estimated with 95 % confidence intervals (CIs). For connected networks, frequentist random-effects NMAs were performed using NMA Studio; otherwise, random-effects pairwise meta-analyses were conducted in R (version 4.2.3) [16].

Analyses covered overall, renal, and gastrointestinal patient populations across all timepoints; oral ferrous sulfate (OFS) results were reported as a common comparator, though the focus remained on IV formulations.

Multi-arm trials were handled via variance-covariance modeling to prevent double-counting.

### Network geometry, transitivity, consistency

Nodes represented iron formulations, and edges indicated direct comparisons. The network geometry is shown in Supplement 3.

Transitivity was evaluated a priori by comparing baseline Hb, TSAT, and ferritin distributions across studies (Supplement 10).

Consistency was tested using design-by-treatment (global) and node-splitting (local) approaches in NMA Studio with p-values <0.05 indicating inconsistencies (Supplement 11).

### Decision time intervals and visualization

Timepoints at 4 and 8 weeks (early response), 12 weeks (intermediate, when available), and 24 weeks (maintenance/safety) were prespecified to reflect time-sensitive decisions.

When trials reported additional timepoints (e.g., 1–3–5–6–10 weeks), each value was mapped to the nearest time interval using a ± two-week tolerance rule for the early timepoint and ± four-week tolerance for 24 weeks.

**Fig. 1:**
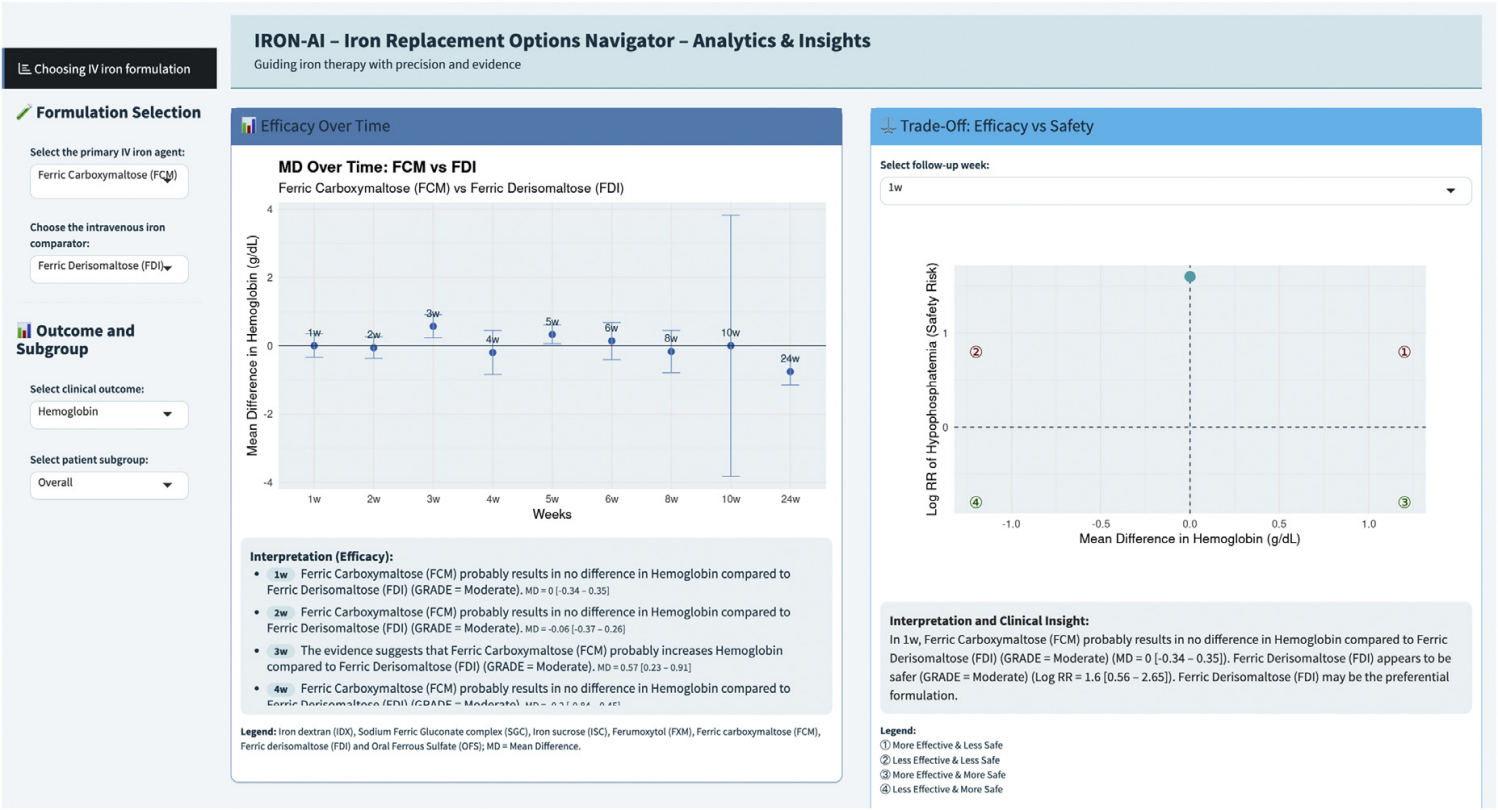
IRON-AI week-by-week visualization and trade-off. The interface displays weekly trajectories of comparative effects. Interpretation follows prespecified timepoints at 4, 8, 12, and 24 weeks adopted in the NMA. Visualization only; no additional analyses are performed.

The RON-AI visualization tool was used to display week-by-week trends derived from these analyses without performing additional computations (Figure 1).

## Results

### Search results and study characteristics

**Fig. 2:**
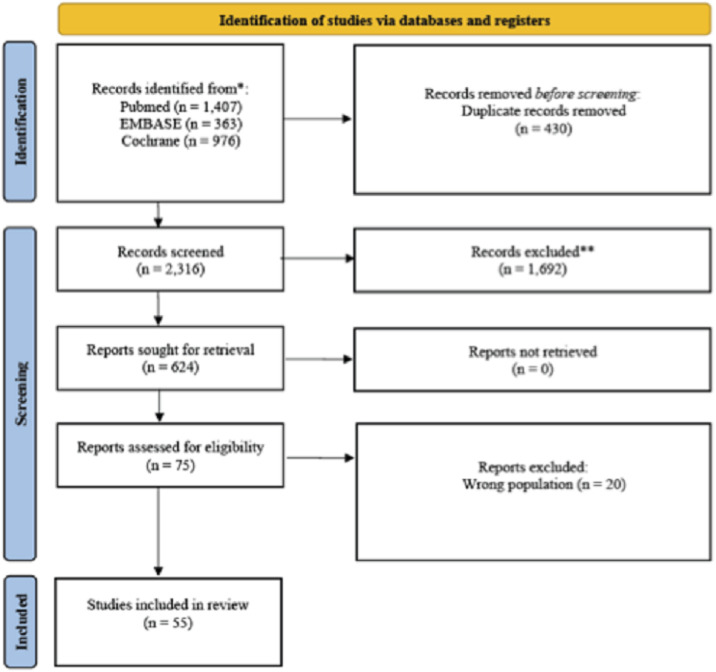
PRISM flow diagram showing selection process.

Fifty-five randomized controlled trials (n = 23,302) published between 2005 and 2024 were included in this study. Six IV formulations were evaluated. Baseline characteristics and risk-of-bias summaries are provided in Supplement Tables 5-8; the PRISMA flow diagram is shown in Figure 2.

Studies were categorized into overall, renal, and gastrointestinal subgroups (Supplement Tables 6 and 7).

### Time-windowed findings

At 4-8 weeks, FCM more consistently increased ferritin/TSAT than alternatives but with a higher hypophosphatemia signal; at 10-24 weeks, differences in stores attenuated and safety considerations predominated, with FDI and ISC generally preferable. Hemoglobin effects were broadly similar between FCM and FDI across most timepoints (Table 1, Table 2, Table 3).

**Table 1:** Comparative efficacy of iron formulations on hemoglobin levels by timepoint (Overall analysis).

IDX: Iron dextran; SFG: Sodium ferric gluconate complex; ISC: Iron sucrose; FXM: ferumoxytol; FCM: Ferric carboxymaltose; FDI: Ferric derisomaltose; OFS: Oral Ferrous Sulfate; NR: Not reported; 95 % CI: 95 % Confidence interval.

Asterisk indicates statistically significant estimate. Shading of the cells indicates the certainty of evidence as follows: green (high); yellow (moderate); orange (low) and red (very low).

**Table 2:** Comparative efficacy of iron formulations on ferritin levels by timepoint (overall analysis).

IDX: Iron dextran; SFG: Sodium ferric gluconate complex; ISC: Iron sucrose; FXM: ferumoxytol; FCM: Ferric carboxymaltose; FDI: Ferric derisomaltose; OFS: Oral Ferrous Sulfate; NR: Not reported; 95 % CI: 95 % Confidence interval.

Asterisk indicates statistically significant estimates. Shading of cells indicates the certainty of evidence as follows: green (high), yellow (moderate), orange (low) and red (very low).

**Table 3:** Comparative efficacy of iron formulations on transferrin saturation by timepoint (Overall analysis).

IDX: Iron dextran; SFG: Sodium ferric gluconate complex; ISC: Iron sucrose; FXM: ferumoxytol; FCM: Ferric carboxymaltose; FDI: Ferric derisomaltose; OFS: Oral Ferrous Sulfate; NR: Not reported; 95 % CI: 95 % Confidence interval.

Asterisk indicates statistically significant estimates. Shading of the cells indicates the certainty of evidence as follows: green (high), yellow (moderate), orange (low) and red (very low).

The 12-week timepoint contained fewer direct observations in several head-to-head contrasts; as a result, estimates relied more on indirect evidence and were downgraded for imprecision (Table 1, Table 2, Table 3; Supplement Tables).

### Change in hemoglobin levels

Across timepoints, FCM versus FDI showed small and variable mean differences, with no consistent clinically important differences. Early contrasts favored FCM over some alternatives at isolated timepoints (e.g., weeks 3-5), while FDI versus ISC showed modest early gains (week 1-3).

By 12-24 weeks, between-formulation differences generally attenuated. Full estimates by timepoint/comparator and certainty are shown in Table 1 (overall) and corresponding Supplement Tables 12 and 13.

### Change in Ferritin levels

At 4-8 weeks, FCM consistently increased ferritin versus OFS and most IV comparators; FDI and ISC also outperformed OFS at several timepoints. Some head-to-head contrasts (e.g., FCM versus FDI and ISC) favored FCM early, but differences narrowed by 10-24 weeks. Timepoint-specific mean differences and certainty are shown in Table 2 (overall) and Supplement Tables 14 and 15.

### Change in transferrin saturation levels

FCM showed early increases compared to several comparators (Weeks 1–5), while FDI > ISC at some early timepoints. By 8-12 weeks, differences attenuated. Full estimates and certainty by timepoint and comparator are provided in Table 3 (overall) and Supplement Tables 16 and 17.

### Serious adverse events

Differences were observed only in the renal subgroup: FCM was associated with a higher risk compared to OFS (RR = 1.27; 95 % CI: 1.04-1.56), whereas FDI suggested a lower risk compared to iron dextran (RR = 0.10; 95 % CI: 0.01-0.97) (Supplement Tables 21–23).

### Hypophosphatemia

FCM was associated with a higher risk of hypophosphatemia, especially in the renal and gastrointestinal subgroups. FDI and ISC had more favorable profiles: in gastrointestinal trials, FDI versus FCM (RR = 0.21; 95 % CI: 0.10-0.46) and ISC versus FCM (RR = 0.04; 95 % CI: 0.02-0.09) indicated substantially lower risks (Supplement Tables 25 and 26).

### Heterogeneity and inconsistency

Localized inconsistency (p-value <0.05) occurred in some pairwise contrasts, largely due to baseline heterogeneity. Global coherence remained acceptable, supporting pooled estimates.

#### IRON-AI

Figure 1 illustrates the IRON-AI *interface* displaying week-by-week efficacy–safety trade-offs aligned with NMA-derived decision time intervals.

## Discussion

This network meta-analysis indicates time- and scenario-dependent differences: early gains in iron stores with FCM coincide with a higher hypophosphatemia signal, whereas FDI and ISC provide a more favorable mineral safety profile for maintenance horizons; Hb effects were small and variable across timepoints.

It is worth noting that a prior study reported a more favorable safety profile for FCM, contrasting with the present results. However, that study lacked a formal assessment of evidence certainty and included a narrower population scope [17].

Importantly, this analysis highlights persistent concerns regarding hypophosphatemia, a known adverse event associated with FCM, which occurred more frequently than with other agents [18, 19, 20]. This review extends current knowledge by evaluating outcomes across distinct subgroups and multiple timepoints. Few previous studies have used direct or indirect network meta-analysis in patients with different anemia etiologies [20, 21, 22, 23].

The present results also suggest that FXM and SFG may be viable alternatives when safety is prioritized, despite modest gains [24]. Some formulations, however, did not demonstrate superiority over OFS, underscoring the need for cautious clinical interpretation and corroboration in future trials [25, 26, 27].

Some pairwise contrasts showed local inconsistency (p-value <0.05), likely reflecting heterogeneity in baseline characteristics. Despite this, overall network coherence was acceptable, supporting the pooled estimates and their interpretation [28].

Limitations include heterogeneous regimens and follow-ups, sparse timepoints for some comparisons, and higher measurement bias risk for adverse events. Interpretation of hypophosphatemia is limited by heterogeneous thresholds across trials (e.g., <2.0 versus <2.5 mg/dL) and outcome ascertainment; standardization in future randomized controlled trials is warranted. These factors may attenuate precision and generalizability for specific comparisons.

Clinically, these results emphasize the trade-off between efficacy and safety that should guide individualized decision-making. FCM demonstrated superior improvements in ferritin and TSAT, with sustained effects for up to two months, making it appropriate for rapid iron repletion or severe anemia. However, its higher risk of hypophosphatemia warrants caution in patients at risk of mineral metabolism disorders. FDI and ISC offer balanced efficacy with better safety profiles and may be preferable for maintenance or in vulnerable populations.

This review underscores the need for high-quality, head-to-head randomized trials with standardized safety outcomes - particularly for serious adverse events and hypophosphatemia - and the inclusion of cost-effectiveness and quality-of-life assessments to inform clinical and policy decisions.

## Conclusion

In summary, the current findings support time- and scenario-dependent choices rather than a single preferred formulation. When rapid repletion over 4-8 weeks is clinically valuable (e.g. active IBD), FCM may be considered for its more consistent gains in ferritin/TSAT provided that serum phosphate is proactively monitored given the risk of hypophosphatemia. In safety-first or maintenance horizons (12-24 weeks), particularly in chronic kidney disease and inflammatory bowel disease, FDI or ISC are generally preferable owing to more favorable mineral safety profiles with broadly comparable hemoglobin effects. From a formulary stewardship perspective, restricting FCM to contexts where short-term repletion and reliable monitoring are feasible, listing FDI and ISC as options where safety predominates, and avoiding routine escalation from oral iron in the absence of a clear indication appear reasonable.

## Data availability statement

The data that support the findings of this study are available from the corresponding author upon reasonable request.

## Conflicts of interest

No conflicts of interest related to the design, conduct, or reporting of this study.

## Appendix Supplementary materials

Image, application 1

## Footnotes

Supplementary material associated with this article can be found in the online version at doi:10.1016/j.htct.2026.106435.
