# Supplementary material for: Time-dependent trade-offs among intravenous iron formulations for iron-deficiency anemia: a longitudinal systematic review and network meta-analysis
Source: Hematol Transfus Cell Ther. 2026 Apr 1;48(2):106435. doi: 10.1016/j.htct.2026.106435 (PMC13068807; doi:10.1016/j.htct.2026.106435)
Supplement: Supplementary file 1 [file mmc1.docx]

**Supplementary material**

**Comparative Efficacy and Safety of Intravenous Iron Formulations for Treating Iron Deficiency Anemia: A Systematic Review and Network Meta-Analysis**

**Summary**

[Supplement 1 – PRISMA Checklist 2](#_Toc210755369)

[Supplement 2 – Search Strategy. 5](#_Toc210755370)

[Supplement 3 – CINeMA and GRADE 10](#_Toc210755371)

[Supplement 4 – Studies excluded. 20](#_Toc210755372)

[Supplement 5 - Reported outcomes by study 22](#_Toc210755373)

[Supplement 6 - Studies’ characteristics 24](#_Toc210755374)

[Supplement 7 – Patients characteristics. 43](#_Toc210755375)

[Supplement 8 – Risk of bias Appraisal 48](#_Toc210755376)

[Supplement 9 – Geometry of Network 54](#_Toc210755377)

[Supplement 10 – Transitivity Assumption 66](#_Toc210755378)

[Supplement 11 - Assessment of inconsistency 101](#_Toc210755379)

[Supplement 12 - Results to Hb - Subgroup Analysis: Renal 107](#_Toc210755380)

[Supplement 13 - Results to Hb - Subgroup Analysis: Gastrointestinal 108](#_Toc210755381)

[Supplement 14 - Results to Ferritin - Subgroup Analysis: Renal 109](#_Toc210755382)

[Supplement 15 - Results to Ferritin - Subgroup Analysis: GI 110](#_Toc210755383)

[Supplement 16 - Results to Transferrin Saturation (TSAT) - Subgroup Analysis: Renal 111](#_Toc210755384)

[Supplement 17- Results to Transferrin Saturation (TSAT) - Subgroup Analysis: GI 113](#_Toc210755385)

[Supplement 18 - Results to Any Adverse Event - Overall Analysis 114](#_Toc210755386)

[Supplement 19 - Results to Any Adverse Event - Renal Analysis 116](#_Toc210755387)

[Supplement 20 - Results to Any Adverse Event - GI Analysis 118](#_Toc210755388)

[Supplement 21 - Results to Serious Adverse Event - Overall Analysis 119](#_Toc210755389)

[Supplement 22 - Results to Serious Adverse Event - Renal Analysis 121](#_Toc210755390)

[Supplement 23 - Results to Serious Adverse Event - GI Analysis 123](#_Toc210755391)

[Supplement 24 - Results to Hypophosphatemia - Overall Analysis 124](#_Toc210755392)

[Supplement 25 - Results to Hypophosphatemia - Renal Analysis 126](#_Toc210755393)

[Supplement 26 - Results to Hypophosphatemia - GI Analysis 127](#_Toc210755394)

# Supplement 1 – PRISMA Checklist

| **Section and Topic** | **Item #** | **Checklist item** | **Location where item is reported** |
| --- | --- | --- | --- |
| **TITLE** | | |  |
| Title | 1 | Identify the report as a systematic review. | Main Text p1-2 |
| **ABSTRACT** | | |  |
| Abstract | 2 | See the PRISMA 2020 for Abstracts checklist. | Main Text p1-2 |
| **INTRODUCTION** | | |  |
| Rationale | 3 | Describe the rationale for the review in the context of existing knowledge. | Main Text p3 |
| Objectives | 4 | Provide an explicit statement of the objective (s) or question (s) the review addresses. | Main Text p4 |
| **METHODS** | | |  |
| Eligibility criteria | 5 | Specify the inclusion and exclusion criteria for the review and how studies were grouped for the syntheses. | Main Text p4 |
| Information sources | 6 | Specify all databases, registers, websites, organisations, reference lists and other sources searched or consulted to identify studies. Specify the date when each source was last searched or consulted. | Main Text p5 |
| Search strategy | 7 | Present the full search strategies for all databases, registers and websites, including any filters and limits used. | Supplement 2 |
| Selection process | 8 | Specify the methods used to decide whether a study met the inclusion criteria of the review, including how many reviewers screened each record and each report retrieved, whether they worked independently, and if applicable, details of automation tools used in the process. | Main Text p5 |
| Data collection process | 9 | Specify the methods used to collect data from reports, including how many reviewers collected data from each report, whether they worked independently, any processes for obtaining or confirming data from study investigators, and if applicable, details of automation tools used in the process. | Main Text p5-6 |
| Data items | 10a | List and define all outcomes for which data were sought. Specify whether all results that were compatible with each outcome domain in each study were sought (e.g. for all measures, time points, analyses) , and if not, the methods used to decide which results to collect. | Main Text p5-6 |
|  | 10b | List and define all other variables for which data were sought (e.g. participant and intervention characteristics, funding sources) . Describe any assumptions made about any missing or unclear information. | Main Text p5-6 |
| Study risk of bias assessment | 11 | Specify the methods used to assess risk of bias in the included studies, including details of the tool (s) used, how many reviewers assessed each study and whether they worked independently, and if applicable, details of automation tools used in the process. | Main Text p6 |
| Effect measures | 12 | Specify for each outcome the effect measure (s) (e.g. risk ratio, mean difference) used in the synthesis or presentation of results. | Main Text p6 |
| Synthesis methods | 13a | Describe the processes used to decide which studies were eligible for each synthesis (e.g. tabulating the study intervention characteristics and comparing against the planned groups for each synthesis (item #5) ) . | Main Text p6-7 |
|  | 13b | Describe any methods required to prepare the data for presentation or synthesis, such as handling of missing summary statistics, or data conversions. | Main Text p6-7 |
|  | 13c | Describe any methods used to tabulate or visually display results of individual studies and syntheses. | Main Text p6-7 |
|  | 13d | Describe any methods used to synthesize results and provide a rationale for the choice (s) . If meta-analysis was performed, describe the model (s) , method (s) to identify the presence and extent of statistical heterogeneity, and software package (s) used. | Main Text p6-7 |
|  | 13e | Describe any methods used to explore possible causes of heterogeneity among study results (e.g. subgroup analysis, meta-regression) . | Main Text p6-7 |
|  | 13f | Describe any sensitivity analyses conducted to assess robustness of the synthesized results. | Main Text p6-7 |
| Reporting bias assessment | 14 | Describe any methods used to assess risk of bias due to missing results in a synthesis (arising from reporting biases) . | Main Text p6 |
| Certainty assessment | 15 | Describe any methods used to assess certainty (or confidence) in the body of evidence for an outcome. | Main Text p6 |
| **RESULTS** | | |  |
| Study selection | 16a | Describe the results of the search and selection process, from the number of records identified in the search to the number of studies included in the review, ideally using a flow diagram. | Main Text p7-8 |
|  | 16b | Cite studies that might appear to meet the inclusion criteria, but which were excluded, and explain why they were excluded. | Supplement 4 |
| Study characteristics | 17 | Cite each included study and present its characteristics. | Supplement 6-7 |
| Risk of bias in studies | 18 | Present assessments of risk of bias for each included study. | Supplement 8 |
| Results of individual studies | 19 | For all outcomes, present, for each study: (a) summary statistics for each group (where appropriate) and (b) an effect estimates and its precision (e.g. confidence/credible interval) , ideally using structured tables or plots. | Supplement 11-25 |
| Results of syntheses | 20a | For each synthesis, briefly summarize the characteristics and risk of bias among contributing studies. | Main Text p10 and Supplement 8 |
|  | 20b | Present results of all statistical syntheses conducted. If meta-analysis was done, present for each the summary estimate and its precision (e.g. confidence/credible interval) and measures of statistical heterogeneity. If comparing groups, describe the direction of the effect. | Supplement 9-24 |
|  | 20c | Present results of all investigations of possible causes of heterogeneity among study results. | NR |
|  | 20d | Present results of all sensitivity analyses conducted to assess the robustness of the synthesized results. | Main Text p10 |
| Reporting biases | 21 | Present assessments of risk of bias due to missing results (arising from reporting biases) for each synthesis assessed. | Supplement 8 |
| Certainty of evidence | 22 | Present assessments of certainty (or confidence) in the body of evidence for each outcome assessed. | Main Text p10 and Supplement 3 |
| **DISCUSSION** | | |  |
| Discussion | 23a | Provide a general interpretation of the results in the context of other evidence. | Main Text p14-15 |
|  | 23b | Discuss any limitations of the evidence included in the review. | Main Text p14-15 |
|  | 23c | Discuss any limitations of the review processes used. | Main Text p14-15 |
|  | 23d | Discuss implications of the results for practice, policy, and future research. | Main Text p14-15 |
| **OTHER INFORMATION** | | |  |
| Registration and protocol | 24a | Provide registration information for the review, including register name and registration number, or state that the review was not registered. | Main Text p1 |
|  | 24b | Indicate where the review protocol can be accessed, or state that a protocol was not prepared. | Main Text p1,4 |
|  | 24c | Describe and explain any amendments to information provided at registration or in the protocol. | Main Text p1,4 |
| Support | 25 | Describe sources of financial or non-financial support for the review, and the role of the funders or sponsors in the review. | Main Text p1 |
| Competing interests | 26 | Declare any competing interests of review authors. | Main Text p16 |
| Availability of data, code and other materials | 27 | Report which of the following are publicly available and where they can be found: template data collection forms; data extracted from included studies; data used for all analyses; analytic code; any other materials used in the review. | All Supplements |

From: Page MJ, McKenzie JE, Bossuyt PM, Boutron I, Hoffmann TC, Mulrow CD, et al. The PRISMA 2020 statement: an updated guideline for reporting systematic reviews. BMJ 2021;372:n71. doi: 10.1136/bmj.n71. This work is licensed under CC BY 4.0. To view a copy of this license, visit <https://creativecommons.org/licenses/by/4.0/>.

| **ADDITIONAL PRISMA – NMA** | | | |
| --- | --- | --- | --- |
| **Section and Topic** | **Item #** | **Checklist item** | **Location where item is reported** |
| S1. Geometry of the network | S1 | Describe methods used to explore the geometry of the treatment network under study and potential biases related to it. This should include how the evidence base has been graphically summarized for presentation, and what characteristics were compiled and used to describe the evidence base to readers | Main Text - Methods |
| S2. Assessment of inconsistency | S2 | Describe the statistical methods used to evaluate the agreement of direct and indirect evidence in the treatment network (s) studied. Describe efforts taken to address its presence when found. | Main Text - Methods |
| S3 - Presentation of network structure | S3 | Provide a network graph of the included studies to enable visualization of the geometry of the treatment network. | Supplement 9 |
| S4 - Summary of network geometry | S4 | Provide a brief overview of characteristics of the treatment network. This may include commentary on the abundance of trials and randomized patients for the different interventions and pairwise comparisons in the network, gaps of evidence in the treatment network, and potential biases reflected by the network structure. | Main Text - Results |
| S5 - Exploration for inconsistency | S5 | Describe results from investigations of inconsistency. This may include such information as measures of model fit to compare consistency and inconsistency models, P values from statistical tests, or summary of inconsistency estimates from different parts of the treatment network | Supplement 10 |

# Supplement 2 – Search Strategy.

**PubMed**

| **Nº** | **Query** | **Results** |
| --- | --- | --- |
| 1 | "Iron-Dextran Complex"[Mesh] | 1,407 |
| 2 | ( ( ( ( ( ( ( ( ( (Iron Dextran Complex[Title/Abstract]) OR (Ferridextran[Title/Abstract]) ) OR (Dextran-Iron Complex[Title/Abstract]) ) OR (Dextran Iron Complex[Title/Abstract]) ) OR (InFed[Title/Abstract]) ) OR (Dexferrum[Title/Abstract]) ) OR (Feosol[Title/Abstract]) ) OR (Icar[Title/Abstract]) ) OR (Imferon[Title/Abstract]) ) OR (Imposil[Title/Abstract]) ) OR (Dextrofer[Title/Abstract]) | 850 |
| 3 | ("Iron-Dextran Complex"[Mesh]) OR ( ( ( ( ( ( ( ( ( ( (Iron Dextran Complex[Title/Abstract]) OR (Ferridextran[Title/Abstract]) ) OR (Dextran-Iron Complex[Title/Abstract]) ) OR (Dextran Iron Complex[Title/Abstract]) ) OR (InFed[Title/Abstract]) ) OR (Dexferrum[Title/Abstract]) ) OR (Feosol[Title/Abstract]) ) OR (Icar[Title/Abstract]) ) OR (Imferon[Title/Abstract]) ) OR (Imposil[Title/Abstract]) ) OR (Dextrofer[Title/Abstract]) ) | 1,955 |
| 4 | "ferrous gluconate" [Supplementary Concept] | 93 |
| 5 | ( ( ( ( ( ( (Fergon[Title/Abstract]) OR (Dextriferron[Title/Abstract]) ) OR (FeG Iron[Title/Abstract]) ) OR (Vitaferro Brause[Title/Abstract]) ) OR (Ferrum Verla[Title/Abstract]) ) OR (Losferron[Title/Abstract]) ) OR (Loesferron[Title/Abstract]) ) OR (Simron[Title/Abstract]) | 227 |
| 6 | ("ferrous gluconate" [Supplementary Concept]) OR ( ( ( ( ( (Fergon[Title/Abstract]) OR (Dextriferron[Title/Abstract]) ) OR (FeG Iron[Title/Abstract]) ) OR (Losferron[Title/Abstract]) ) OR (Loesferron[Title/Abstract]) ) OR (Simron[Title/Abstract]) ) | 318 |
| 7 | "Ferric Oxide, Saccharated"[Mesh] | 641 |
| 8 | ( ( ( ( ( ( ( ( (Saccharated Ferric Oxide[Title/Abstract]) OR (Ferric Saccharate[Title/Abstract]) ) OR (Iron Sucrose[Title/Abstract]) ) OR (Iron-Saccharate[Title/Abstract]) ) OR (Iron Saccharate[Title/Abstract]) ) OR (Ferri-Saccharate[Title/Abstract]) ) OR (Ferri Saccharate[Title/Abstract]) ) OR (Iron Oxide (Saccharated[Title/Abstract]) ) ) OR (Venofer[Title/Abstract]) ) OR (Hippiron[Title/Abstract]) | 887 |
| 9 | ( ( ( ( ( ( ( ( (Saccharated Ferric Oxide[Title/Abstract]) OR (Ferric Saccharate[Title/Abstract]) ) OR (Iron Sucrose[Title/Abstract]) ) OR (Iron-Saccharate[Title/Abstract]) ) OR (Iron Saccharate[Title/Abstract]) ) OR (Ferri-Saccharate[Title/Abstract]) ) OR (Ferri Saccharate[Title/Abstract]) ) OR (Iron Oxide (Saccharated[Title/Abstract]) ) ) OR (Venofer[Title/Abstract]) ) OR (Hippiron[Title/Abstract]) | 1,058 |
| 10 | "Ferrosoferric Oxide"[Mesh] | 5,046 |
| 11 | ( (Magnetite[Title/Abstract]) OR (Feraheme[Title/Abstract]) ) OR (Ferumoxytol[Title/Abstract]) | 8,186 |
| 12 | ("Ferrosoferric Oxide"[Mesh]) OR ( ( (Magnetite[Title/Abstract]) OR (Feraheme[Title/Abstract]) ) OR (Ferumoxytol[Title/Abstract]) ) | 11,903 |
| 13 | "ferric carboxymaltose" [Supplementary Concept] | 503 |
| 14 | ( ( ( ( (iron carboxymaltose[Title/Abstract]) OR (iron dextri-maltose[Title/Abstract]) ) OR (Ferinject[Title/Abstract]) ) OR (VIT-45[Title/Abstract]) ) OR (VIT 45[Title/Abstract]) ) OR (injectafer[Title/Abstract]) | 101 |
| 15 | ("ferric carboxymaltose" [Supplementary Concept]) OR ( ( ( ( ( (iron carboxymaltose[Title/Abstract]) OR (iron dextri-maltose[Title/Abstract]) ) OR (Ferinject[Title/Abstract]) ) OR (VIT-45[Title/Abstract]) ) OR (VIT 45[Title/Abstract]) ) OR (injectafer[Title/Abstract]) ) | 559 |
| 16 | "iron isomaltoside 1000" [Supplementary Concept] | 84 |
| 17 | "iron isomaltoside"[Title/Abstract] | 127 |
| 18 | "ferric derisomaltose"[Text Word] | 102 |
| 19 | ( ("iron isomaltoside 1000" [Supplementary Concept]) OR (iron isomaltoside[Title/Abstract]) ) OR (ferric derisomaltose[Text Word]) | 215 |
| 20 | ( ( ( ( ( ("Iron-Dextran Complex"[Mesh]) OR ( ( ( ( ( ( ( ( ( ( (Iron Dextran Complex[Title/Abstract]) OR (Ferridextran[Title/Abstract]) ) OR (Dextran-Iron Complex[Title/Abstract]) ) OR (Dextran Iron Complex[Title/Abstract]) ) OR (InFed[Title/Abstract]) ) OR (Dexferrum[Title/Abstract]) ) OR (Feosol[Title/Abstract]) ) OR (Icar[Title/Abstract]) ) OR (Imferon[Title/Abstract]) ) OR (Imposil[Title/Abstract]) ) OR (Dextrofer[Title/Abstract]) ) ) OR ( ("ferrous gluconate" [Supplementary Concept]) OR ( ( ( ( ( (Fergon[Title/Abstract]) OR (Dextriferron[Title/Abstract]) ) OR (FeG Iron[Title/Abstract]) ) OR (Losferron[Title/Abstract]) ) OR (Loesferron[Title/Abstract]) ) OR (Simron[Title/Abstract]) ) ) ) OR ( ( ( ( ( ( ( ( ( (Saccharated Ferric Oxide[Title/Abstract]) OR (Ferric Saccharate[Title/Abstract]) ) OR (Iron Sucrose[Title/Abstract]) ) OR (Iron-Saccharate[Title/Abstract]) ) OR (Iron Saccharate[Title/Abstract]) ) OR (Ferri-Saccharate[Title/Abstract]) ) OR (Ferri Saccharate[Title/Abstract]) ) OR (Iron Oxide (Saccharated[Title/Abstract]) ) ) OR (Venofer[Title/Abstract]) ) OR (Hippiron[Title/Abstract]) ) ) OR ( ("Ferrosoferric Oxide"[Mesh]) OR ( ( (Magnetite[Title/Abstract]) OR (Feraheme[Title/Abstract]) ) OR (Ferumoxytol[Title/Abstract]) ) ) ) OR ( ("ferric carboxymaltose" [Supplementary Concept]) OR ( ( ( ( ( (iron carboxymaltose[Title/Abstract]) OR (iron dextri-maltose[Title/Abstract]) ) OR (Ferinject[Title/Abstract]) ) OR (VIT-45[Title/Abstract]) ) OR (VIT 45[Title/Abstract]) ) OR (injectafer[Title/Abstract]) ) ) ) OR ( ( ("iron isomaltoside 1000" [Supplementary Concept]) OR (iron isomaltoside[Title/Abstract]) ) OR (ferric derisomaltose[Text Word]) ) | 14,695 |
| 21 | "Anemia, Iron-Deficiency"[Mesh] | 11,928 |
| 22 | ( ( ( ( ( (Anemia, Iron Deficiency[Title/Abstract]) OR (Iron-Deficiency Anemia[Title/Abstract]) ) OR (Iron Deficiency Anemia[Title/Abstract]) ) OR (Anemias, Iron-Deficiency[Title/Abstract]) ) OR (Anemias, Iron Deficiency[Title/Abstract]) ) OR (Iron-Deficiency Anemias[Title/Abstract]) ) OR (Iron Deficiency Anemias[Title/Abstract]) | 9,468 |
| 23 | ("Anemia, Iron-Deficiency"[Mesh]) OR ( ( ( ( ( ( (Anemia, Iron Deficiency[Title/Abstract]) OR (Iron-Deficiency Anemia[Title/Abstract]) ) OR (Iron Deficiency Anemia[Title/Abstract]) ) OR (Anemias, Iron-Deficiency[Title/Abstract]) ) OR (Anemias, Iron Deficiency[Title/Abstract]) ) OR (Iron-Deficiency Anemias[Title/Abstract]) ) OR (Iron Deficiency Anemias[Title/Abstract]) ) | 17,017 |
| 24 | ( ("Anemia, Iron-Deficiency"[Mesh]) OR ( ( ( ( ( ( (Anemia, Iron Deficiency[Title/Abstract]) OR (Iron-Deficiency Anemia[Title/Abstract]) ) OR (Iron Deficiency Anemia[Title/Abstract]) ) OR (Anemias, Iron-Deficiency[Title/Abstract]) ) OR (Anemias, Iron Deficiency[Title/Abstract]) ) OR (Iron-Deficiency Anemias[Title/Abstract]) ) OR (Iron Deficiency Anemias[Title/Abstract]) ) ) AND ( ( ( ( ( ( ("Iron-Dextran Complex"[Mesh]) OR ( ( ( ( ( ( ( ( ( ( (Iron Dextran Complex[Title/Abstract]) OR (Ferridextran[Title/Abstract]) ) OR (Dextran-Iron Complex[Title/Abstract]) ) OR (Dextran Iron Complex[Title/Abstract]) ) OR (InFed[Title/Abstract]) ) OR (Dexferrum[Title/Abstract]) ) OR (Feosol[Title/Abstract]) ) OR (Icar[Title/Abstract]) ) OR (Imferon[Title/Abstract]) ) OR (Imposil[Title/Abstract]) ) OR (Dextrofer[Title/Abstract]) ) ) OR ( ("ferrous gluconate" [Supplementary Concept]) OR ( ( ( ( ( (Fergon[Title/Abstract]) OR (Dextriferron[Title/Abstract]) ) OR (FeG Iron[Title/Abstract]) ) OR (Losferron[Title/Abstract]) ) OR (Loesferron[Title/Abstract]) ) OR (Simron[Title/Abstract]) ) ) ) OR ( ( ( ( ( ( ( ( ( (Saccharated Ferric Oxide[Title/Abstract]) OR (Ferric Saccharate[Title/Abstract]) ) OR (Iron Sucrose[Title/Abstract]) ) OR (Iron-Saccharate[Title/Abstract]) ) OR (Iron Saccharate[Title/Abstract]) ) OR (Ferri-Saccharate[Title/Abstract]) ) OR (Ferri Saccharate[Title/Abstract]) ) OR (Iron Oxide (Saccharated[Title/Abstract]) ) ) OR (Venofer[Title/Abstract]) ) OR (Hippiron[Title/Abstract]) ) ) OR ( ("Ferrosoferric Oxide"[Mesh]) OR ( ( (Magnetite[Title/Abstract]) OR (Feraheme[Title/Abstract]) ) OR (Ferumoxytol[Title/Abstract]) ) ) ) OR ( ("ferric carboxymaltose" [Supplementary Concept]) OR ( ( ( ( ( (iron carboxymaltose[Title/Abstract]) OR (iron dextri-maltose[Title/Abstract]) ) OR (Ferinject[Title/Abstract]) ) OR (VIT-45[Title/Abstract]) ) OR (VIT 45[Title/Abstract]) ) OR (injectafer[Title/Abstract]) ) ) ) OR ( ( ("iron isomaltoside 1000" [Supplementary Concept]) OR (iron isomaltoside[Title/Abstract]) ) OR (ferric derisomaltose[Text Word]) ) ) | 1,031 |
| 25 | ( ("Anemia, Iron-Deficiency"[Mesh]) OR ( ( ( ( ( ( (Anemia, Iron Deficiency[Title/Abstract]) OR (Iron-Deficiency Anemia[Title/Abstract]) ) OR (Iron Deficiency Anemia[Title/Abstract]) ) OR (Anemias, Iron-Deficiency[Title/Abstract]) ) OR (Anemias, Iron Deficiency[Title/Abstract]) ) OR (Iron-Deficiency Anemias[Title/Abstract]) ) OR (Iron Deficiency Anemias[Title/Abstract]) ) ) AND ( ( ( ( ( ( ("Iron-Dextran Complex"[Mesh]) OR ( ( ( ( ( ( ( ( ( ( (Iron Dextran Complex[Title/Abstract]) OR (Ferridextran[Title/Abstract]) ) OR (Dextran-Iron Complex[Title/Abstract]) ) OR (Dextran Iron Complex[Title/Abstract]) ) OR (InFed[Title/Abstract]) ) OR (Dexferrum[Title/Abstract]) ) OR (Feosol[Title/Abstract]) ) OR (Icar[Title/Abstract]) ) OR (Imferon[Title/Abstract]) ) OR (Imposil[Title/Abstract]) ) OR (Dextrofer[Title/Abstract]) ) ) OR ( ("ferrous gluconate" [Supplementary Concept]) OR ( ( ( ( ( (Fergon[Title/Abstract]) OR (Dextriferron[Title/Abstract]) ) OR (Losferron[Title/Abstract]) ) OR (Loesferron[Title/Abstract]) ) OR (Simron[Title/Abstract]) ) ) ) OR ( ( ( ( ( ( ( ( ( (Saccharated Ferric Oxide[Title/Abstract]) OR (Ferric Saccharate[Title/Abstract]) ) OR (Iron Sucrose[Title/Abstract]) ) OR (Iron-Saccharate[Title/Abstract]) ) OR (Iron Saccharate[Title/Abstract]) ) OR (Ferri-Saccharate[Title/Abstract]) ) OR (Ferri Saccharate[Title/Abstract]) ) OR (Iron Oxide (Saccharated[Title/Abstract]) ) ) OR (Venofer[Title/Abstract]) ) OR (Hippiron[Title/Abstract]) ) ) OR ( ("Ferrosoferric Oxide"[Mesh]) OR ( ( (Magnetite[Title/Abstract]) OR (Feraheme[Title/Abstract]) ) OR (Ferumoxytol[Title/Abstract]) ) ) ) OR ( ("ferric carboxymaltose" [Supplementary Concept]) OR ( ( ( ( ( (iron carboxymaltose[Title/Abstract]) OR (iron dextri-maltose[Title/Abstract]) ) OR (Ferinject[Title/Abstract]) ) OR (VIT-45[Title/Abstract]) ) OR (VIT 45[Title/Abstract]) ) OR (injectafer[Title/Abstract]) ) ) ) OR ( ( ("iron isomaltoside 1000" [Supplementary Concept]) OR (iron isomaltoside[Title/Abstract]) ) OR (ferric derisomaltose[Text Word]) ) ) | 244+45 |

**Cochrane Library**

| **Terms** | **Number** |
| --- | --- |
| #1 MeSH descriptor: [Anemia, Iron-Deficiency] explode all trees 1802  #2 Anemia, Iron Deficiency 4222  #3 Iron Deficiency Anemias 68  #4 Iron Deficiency Anemia 4222  #5 Iron-Deficiency Anemias 58  #6 Anemias, Iron Deficiency 68  #7 Anemias, Iron-Deficiency 58  #8 Iron-Deficiency Anemia 3976  #9 {OR #1-#8} 4230  #10 MeSH descriptor: [Iron-Dextran Complex] explode all trees 79  #11 Imperon 0  #12 Norferan 0  #13 Dextran Iron Complex 111  #14 Dextran-Iron Complex 6  #15 Iron Dextran Complex 111  #16 Ferridextran 0  #17 Dexferrum 2  #18 Feosol 4  #19 InFed 3  #20 Imposil 0  #21 Imferon 12  #22 Hematran 0  #23 Icar 56  #24 Dextrofer 0  #25 Imfergen 0  #26 {OR #10-#25} 173  #27 Ferrous gluconate 123  #28 Fergon 2  #29 Dextriferron 3  #30 FeG Iron 60  #31 Vitaferro Brause 0  #32 Ferrum Verla 0  #33 Losferron 5  #34 Loesferron 1  #35 Simron 0  #36 {OR #27-#35} 188  #37 MeSH descriptor: [Ferric Oxide, Saccharated] explode all trees 234  #38 Venofer 94  #39 Iron Oxide (Saccharated) 251  #40 Iron Saccharate 69  #41 Ferric Saccharate 35  #42 Iron-Saccharate 64  #43 Ferri Saccharate 0  #44 Ferri-Saccharate 0  #45 Saccharated Ferric Oxide 248  #46 Iron Sucrose 578  #47 Hippiron 0  #48 {OR #37-#47} 679  #49 MeSH descriptor: [Ferrosoferric Oxide] explode all trees 99  #50 Ferumoxytol 144  #51 Feraheme 15  #52 Ferriferrous Oxide 0  #53 Oxide, Ferrosoferric 101  #54 Oxide, Ferriferrous 0  #55 Magnetite 63  #56 {OR #49-#55} 210  #57 Ferric carboxymaltose 638  #58 iron carboxymaltose 638  #59 iron dextri-maltose 0  #60 Ferinject 159  #61 VIT-45 8  #62 VIT 45 220  #63 injectafer 27  #64 {OR #57-#63} 900  #65 Iron isomaltoside 189  #66 iron isomaltoside 1000 124  #67 ferric derisomaltose 97  #68 {OR #65-#67} 257  #69 #26 OR #36 OR #48 OR #56 OR #64 OR #68 2080  #70 #9 AND #69 976 | 976 |

**EMBASE**

| **Nº** | **Query** | **Number** |
| --- | --- | --- |
| **#28** | #27 AND ('clinical trial'/de OR 'clinical trial topic'/de OR 'controlled clinical trial'/de OR 'phase 3 clinical trial'/de OR 'phase 3 clinical trial topic'/de OR 'randomized controlled trial'/de OR 'randomized controlled trial topic'/de) | 363 |
| **#27** | #26 AND [embase]/lim NOT ([embase]/lim AND [medline]/lim) | 1381 |
| **#26** | #22 AND #25 | 3199 |
| **#25** | #23 OR #24 | 41012 |
| **#24** | 'anaemia, hypochromic' OR 'anaemia, iron deficiency' OR 'anaemia, iron-deficiency' OR 'anaemia, microcytic hypochromic' OR 'anemia, hypochromic' OR 'anemia, iron deficiency' OR 'anemia, iron-deficiency' OR 'anemia, microcytic hypochromic' OR 'asiderotic anaemia' OR 'asiderotic anemia' OR 'ferriprive anaemia' OR 'ferriprive anemia' OR 'hypochrome anaemia' OR 'hypochrome anemia' OR 'hypochromic anaemia' OR 'hypochromic anemia' OR 'hypochromic iron deficiency anaemia' OR 'hypochromic iron deficiency anemia' OR 'hypochromic microcytic anaemia' OR 'hypochromic microcytic anemia' OR 'hypoferrous anaemia' OR 'hypoferrous anemia' OR 'iron deficiency anaemia' OR 'iron deficient anaemia' OR 'iron deficient anemia' OR 'iron refractory anaemia' OR 'iron refractory anemia' OR 'iron-deficiency anaemia' OR 'iron-deficiency anemia' OR 'microcytic hypochromic anaemia' OR 'microcytic hypochromic anemia' OR 'sideropenic anaemia' OR 'sideropenic anemia' OR 'iron deficiency anemia' | 40986 |
| **#23** | 'iron deficiency anemia'/exp | 36775 |
| **#22** | #3 OR #6 OR #9 OR #12 OR #15 OR #18 OR #21 | 42843 |
| **#21** | #19 OR #20 | 467 |
| **#20** | 'dextrifer' OR 'dextrifer s' OR 'dextrifer-s' OR 'ferric hydroxide polymaltose' OR 'ferric hydroxide polymaltose complex' OR 'ferrosig' OR 'ferrous polymaltosate' OR 'ferrous polymaltose complex' OR 'hemafer' OR 'iron hydroxide polymaltose' OR 'iron polymaltosate' OR 'iron polymaltose complex' OR 'iron polymaltose hydroxide' OR 'polymaltose iron hydroxide complex' OR 'teferrol' OR 'iron polymaltose' | 467 |
| **#19** | 'iron polymaltose'/exp | 432 |
| **#18** | #16 OR #17 | 903 |
| **#17** | ' (1-6) alpha dextro glucopyranan (1-6) dextro glucitol iron (iii) complex' OR 'diafer' OR 'ferric derisomaltose' OR 'iron isomaltoside 1000' OR 'isofer' OR 'isomaltose iron' OR 'isomaltose, ferric complex' OR 'jilazo' OR 'monofer' OR 'monoferric' OR 'monoferro' OR 'monover' OR 'ns 32' OR 'ns32' OR 'iron isomaltose' | 903 |
| **#16** | 'iron isomaltose'/exp | 420 |
| **#15** | #13 OR #14 | 2446 |
| **#14** | 'eiseninject' OR 'ferinject' OR 'injectafer' OR 'iroprem' OR 'poly [dextro glucopyranosyl (1-4) ] dextro gluconic acid complex of hydrated iron oxide' OR 'renegy' OR 'z 213' OR 'z213' OR 'ferric carboxymaltose' | 2446 |
| **#13** | 'ferric carboxymaltose'/exp | 2291 |
| **#12** | #10 OR #11 | 1919 |
| **#11** | 'ami 7228' OR 'ami7228' OR 'feraheme' OR 'rienso' OR 'ferumoxytol' | 1919 |
| **#10** | 'ferumoxytol'/exp | 1793 |
| **#9** | #7 OR #8 | 3303 |
| **#8** | 'alvofer' OR 'colliron' OR 'faremio' OR 'fer (iron saccharate) ' OR 'ferion' OR 'feriv' OR 'fermed' OR 'ferri saccharate' OR 'ferric hydroxide sucrose' OR 'ferric hydroxide sucrose complex' OR 'ferric oxide saccharate' OR 'ferric oxide, saccharated' OR 'ferric saccharate' OR 'ferrinemia' OR 'ferrisaccharate' OR 'ferrivenin' OR 'ferrologic' OR 'ferroprol' OR 'ferrous saccharate' OR 'ferrovin' OR 'fesin' OR 'hemafer s' OR 'hemafer-s' OR 'idafer' OR 'iron (iii) hydroxide sucrose complex' OR 'iron sucrose' OR 'ironcrose' OR 'iviron' OR 'nefro-fer' OR 'nefrofer' OR 'neo ferrum' OR 'nephroferol' OR 'proferrin' OR 'referen' OR 'reoxyl' OR 'saccharate ferric' OR 'saccharate iron' OR 'saccharated ferric oxide' OR 'saccharated iron oxide' OR 'sucro fer' OR 'sucrofer' OR 'sucroven' OR 'veniron' OR 'venofer' OR 'venotrix' OR 'xi 921' OR 'xi921' OR 'iron saccharate' | 3303 |
| **#7** | 'iron saccharate'/exp | 3013 |
| **#6** | #4 OR #5 | 32729 |
| **#5** | 'bis (gluconato) iron' OR 'epitone' OR 'fenton' OR 'feragluc' OR 'ferdiv' OR 'fergon' OR 'ferlucon' OR 'ferolib' OR 'ferosac' OR 'ferralet' OR 'ferreluc' OR 'ferretti' OR 'ferrnat' OR 'ferrobival' OR 'ferrodue' OR 'ferrogluconate' OR 'ferroglyconicum' OR 'ferrogyn' OR 'ferrolysine' OR 'ferronat c' OR 'ferronicum' OR 'ferruten' OR 'glistron' OR 'gloros' OR 'gluco ferrum' OR 'glucofer' OR 'glucoferro' OR 'grofer' OR 'hemototal' OR 'iron bis (gluconate) ' OR 'iron gluconate' OR 'iron sodium gluconate' OR 'irox' OR 'loesferron' OR 'losferron' OR 'nionate' OR 'novifer' OR 'novoferrogluc' OR 'prifer' OR 'prontoferro' OR 'rafesac' OR 'simron' OR 'sustemial' OR 'sym fer' OR 'symfer' OR 'viofer' OR 'vitaferri' OR 'vitaferro' OR 'vitaferro brause' OR 'zaofer' OR 'ferrous gluconate' | 32729 |
| **#4** | 'ferrous gluconate'/exp | 3408 |
| **#3** | #1 OR #2 | 4073 |
| **#2** | 'anaemex' OR 'cosmofer' OR 'dexferrum' OR 'dexiron' OR 'dextrafer' OR 'dextran fe' OR 'dextran ferrous' OR 'dextran iron' OR 'dextran iron complex' OR 'driken' OR 'fenate' OR 'fer dextran' OR 'fercayl' OR 'ferric dextran' OR 'ferridextran' OR 'ferrisat' OR 'ferrodex' OR 'ferrodextran' OR 'ferrous dextran' OR 'ferrum lek' OR 'fervetag' OR 'hibiron' OR 'imferdex' OR 'imferon' OR 'impheron' OR 'imposil' OR 'infed' OR 'infufer' OR 'iron dextran complex' OR 'iron-dextran complex' OR 'ironate' OR 'jerndextran' OR 'monofar' OR 'proferdex' OR 'uniferon' OR 'uniferon f' OR 'uniferon f 2' OR 'uniferron f' OR 'iron dextran' | 4073 |
| **#1** | 'iron dextran'/exp | 3293 |

# Supplement 3 – CINeMA and GRADE

**CINeMA – Hb level – 1 week – Overall analysis**

| **Comparison** | **Number of studies** | **Within-study bias** | **Reporting bias** | **Indirectness** | **Imprecision** | **Heterogeneity** | **Incoherence** | **Confidence rating** | **Reason (s) for downgrading** |
| --- | --- | --- | --- | --- | --- | --- | --- | --- | --- |
| **FCM:FDI** | 2 | No concerns | Low risk | No concerns | Major concerns | No concerns | No concerns | Moderate | ["Imprecision"] |
| **FCM:ISC** | 1 | Some concerns | Low risk | No concerns | Major concerns | No concerns | No concerns | Low | ["Within-study bias","Imprecision"] |
| **FDI:ISC** | 2 | Some concerns | Low risk | No concerns | No concerns | No concerns | No concerns | Moderate | ["Within-study bias"] |
| **FDI:OFS** | 1 | No concerns | Low risk | No concerns | Major concerns | No concerns | No concerns | Moderate | ["Imprecision"] |
| **ISC:PCB** | 1 | Major concerns | Low risk | No concerns | Major concerns | No concerns | No concerns | Very low | ["Within-study bias","Imprecision"] |
| **FCM:OFS** | 0 | No concerns | Low risk | No concerns | Major concerns | No concerns | No concerns | Moderate | ["Imprecision"] |
| **FCM:PCB** | 0 | Some concerns | Low risk | No concerns | Major concerns | No concerns | No concerns | Low | ["Within-study bias","Imprecision"] |
| **FDI:PCB** | 0 | Some concerns | Low risk | No concerns | Major concerns | No concerns | No concerns | Low | ["Within-study bias","Imprecision"] |
| **ISC:OFS** | 0 | No concerns | Low risk | No concerns | Major concerns | No concerns | No concerns | Moderate | ["Imprecision"] |
| **OFS:PCB** | 0 | Some concerns | Low risk | No concerns | Major concerns | No concerns | No concerns | Low | ["Within-study bias","Imprecision"] |

**CINeMA – Hb – 2 week – GOverall analysis**

| **Comparison** | **Number of studies** | **Within-study bias** | **Reporting bias** | **Indirectness** | **Imprecision** | **Heterogeneity** | **Incoherence** | **Confidence rating** | **Reason (s) for downgrading** |
| --- | --- | --- | --- | --- | --- | --- | --- | --- | --- |
| **FCM:FDI** | 3 | No concerns | Low risk | No concerns | Major concerns | No concerns | No concerns | Moderate | ["Imprecision"] |
| **FCM:ISC** | 1 | No concerns | Low risk | No concerns | Major concerns | No concerns | Major concerns | Very low | ["Imprecision","Incoherence"] |
| **FCM:OFS** | 1 | Some concerns | Low risk | No concerns | Major concerns | No concerns | No concerns | Low | ["Within-study bias","Imprecision"] |
| **FDI:ISC** | 4 | Some concerns | Low risk | No concerns | Major concerns | No concerns | Major concerns | Very low | ["Within-study bias","Imprecision","Incoherence"] |
| **FDI:OFS** | 2 | No concerns | Low risk | No concerns | Major concerns | No concerns | No concerns | Moderate | ["Imprecision"] |
| **FXM:ISC** | 1 | Some concerns | Low risk | No concerns | Major concerns | No concerns | Major concerns | Very low | ["Within-study bias","Imprecision","Incoherence"] |
| **FXM:PCB** | 1 | Some concerns | Low risk | No concerns | No concerns | No concerns | Major concerns | Low | ["Within-study bias","Incoherence"] |
| **FCM:FXM** | 0 | Some concerns | Low risk | No concerns | Major concerns | No concerns | Major concerns | Very low | ["Within-study bias","Imprecision","Incoherence"] |
| **FCM:PCB** | 0 | Some concerns | Low risk | No concerns | No concerns | No concerns | Major concerns | Low | ["Within-study bias","Incoherence"] |
| **FDI:FXM** | 0 | Some concerns | Low risk | No concerns | Major concerns | No concerns | Major concerns | Very low | ["Within-study bias","Imprecision","Incoherence"] |
| **FDI:PCB** | 0 | Some concerns | Low risk | No concerns | No concerns | No concerns | Major concerns | Low | ["Within-study bias","Incoherence"] |
| **FXM:OFS** | 0 | Some concerns | Low risk | No concerns | Major concerns | No concerns | Major concerns | Very low | ["Within-study bias","Imprecision","Incoherence"] |
| **ISC:OFS** | 0 | No concerns | Low risk | No concerns | Major concerns | No concerns | Major concerns | Low | ["Imprecision","Incoherence"] |
| **ISC:PCB** | 0 | Some concerns | Low risk | No concerns | No concerns | No concerns | Major concerns | Low | ["Within-study bias","Incoherence"] |
| **OFS:PCB** | 0 | Some concerns | Low risk | No concerns | No concerns | Major concerns | Major concerns | Very low | ["Within-study bias","Heterogeneity","Incoherence"] |

**CINeMA – Hb 3 week – Overall analysis**

| **Comparison** | **Number of studies** | **Within-study bias** | **Reporting bias** | **Indirectness** | **Imprecision** | **Heterogeneity** | **Incoherence** | **Confidence rating** | **Reason (s) for downgrading** |
| --- | --- | --- | --- | --- | --- | --- | --- | --- | --- |
| **FCM:FDI** | 2 | No concerns | Low risk | No concerns | No concerns | Major concerns | No concerns | Low | ["Heterogeneity"] |
| **FCM:OFS** | 1 | Some concerns | Low risk | No concerns | No concerns | No concerns | No concerns | Moderate | ["Within-study bias"] |
| **FDI:ISC** | 1 | Some concerns | Low risk | No concerns | Major concerns | No concerns | Major concerns | Very low | ["Within-study bias","Imprecision","Incoherence"] |
| **FDI:OFS** | 1 | Some concerns | Low risk | No concerns | Major concerns | No concerns | Major concerns | Very low | ["Within-study bias","Imprecision","Incoherence"] |
| **FFM:FXM** | 1 | No concerns | Low risk | No concerns | Major concerns | No concerns | Major concerns | Low | ["Imprecision","Incoherence"] |
| **FXM:ISC** | 1 | Some concerns | Low risk | No concerns | Major concerns | No concerns | Major concerns | Very low | ["Within-study bias","Imprecision","Incoherence"] |
| **FXM:PCB** | 1 | Some concerns | Low risk | No concerns | No concerns | No concerns | Major concerns | Low | ["Within-study bias","Incoherence"] |
| **IDX:ISC** | 1 | Major concerns | Low risk | No concerns | Major concerns | No concerns | Major concerns | Very low | ["Within-study bias","Imprecision","Incoherence"] |
| **ISC:OFS** | 2 | No concerns | Low risk | No concerns | Major concerns | No concerns | Major concerns | Very low | ["Imprecision","Incoherence"] |
| **FCM:FFM** | 0 | Some concerns | Low risk | No concerns | No concerns | Major concerns | Major concerns | Very low | ["Within-study bias","Heterogeneity","Incoherence"] |
| **FCM:FXM** | 0 | Some concerns | Low risk | No concerns | No concerns | Major concerns | Major concerns | Very low | ["Within-study bias","Heterogeneity","Incoherence"] |
| **FCM:IDX** | 0 | Some concerns | Low risk | No concerns | No concerns | Major concerns | Major concerns | Very low | ["Within-study bias","Heterogeneity","Incoherence"] |
| **FCM:ISC** | 0 | Some concerns | Low risk | No concerns | No concerns | No concerns | Major concerns | Low | ["Within-study bias","Incoherence"] |
| **FCM:PCB** | 0 | Some concerns | Low risk | No concerns | No concerns | No concerns | Major concerns | Low | ["Within-study bias","Incoherence"] |
| **FDI:FFM** | 0 | Some concerns | Low risk | No concerns | Major concerns | No concerns | Major concerns | Very low | ["Within-study bias","Imprecision","Incoherence"] |
| **FDI:FXM** | 0 | Some concerns | Low risk | No concerns | Major concerns | No concerns | Major concerns | Very low | ["Within-study bias","Imprecision","Incoherence"] |
| **FDI:IDX** | 0 | Some concerns | Low risk | No concerns | Major concerns | No concerns | Major concerns | Very low | ["Within-study bias","Imprecision","Incoherence"] |
| **FDI:PCB** | 0 | Some concerns | Low risk | No concerns | No concerns | No concerns | Major concerns | Low | ["Within-study bias","Incoherence"] |
| **FFM:IDX** | 0 | Some concerns | Low risk | No concerns | Major concerns | No concerns | Major concerns | Very low | ["Within-study bias","Imprecision","Incoherence"] |
| **FFM:ISC** | 0 | Some concerns | Low risk | No concerns | Major concerns | No concerns | Major concerns | Very low | ["Within-study bias","Imprecision","Incoherence"] |
| **FFM:OFS** | 0 | No concerns | Low risk | No concerns | Major concerns | No concerns | Major concerns | Very low | ["Imprecision","Incoherence"] |
| **FFM:PCB** | 0 | Some concerns | Low risk | No concerns | No concerns | No concerns | Major concerns | Very low | ["Within-study bias","Incoherence"] |
| **FXM:IDX** | 0 | Major concerns | Low risk | No concerns | Major concerns | No concerns | Major concerns | Very low | ["Within-study bias","Imprecision","Incoherence"] |
| **FXM:OFS** | 0 | Some concerns | Low risk | No concerns | Major concerns | No concerns | Major concerns | Very low | ["Within-study bias","Imprecision","Incoherence"] |
| **IDX:OFS** | 0 | Some concerns | Low risk | No concerns | Major concerns | No concerns | Major concerns | Very low | ["Within-study bias","Imprecision","Incoherence"] |
| **IDX:PCB** | 0 | Some concerns | Low risk | No concerns | No concerns | Major concerns | Major concerns | Very low | ["Within-study bias","Incoherence"] |
| **ISC:PCB** | 0 | Some concerns | Low risk | No concerns | No concerns | No concerns | Major concerns | Very low | ["Within-study bias","Incoherence"] |
| **OFS:PCB** | 0 | Some concerns | Low risk | No concerns | No concerns | No concerns | Major concerns | Very low | ["Within-study bias","Incoherence"] |

**CINeMA – Hb 4 week – Overall analysis**

| **Comparison** | **Number of studies** | **Within-study bias** | **Reporting bias** | **Indirectness** | **Imprecision** | **Heterogeneity** | **Incoherence** | **Confidence rating** | **Reason (s) for downgrading** |
| --- | --- | --- | --- | --- | --- | --- | --- | --- | --- |
| **CAI:FCM** | 1 | No concerns | Low risk | No concerns | No concerns | Major concerns | Major concerns | Low | ["Heterogeneity","Incoherence"] |
| **FCM:ISC** | 1 | No concerns | Low risk | No concerns | Major concerns | No concerns | No concerns | Moderate | ["Imprecision"] |
| **FCM:OFM** | 1 | No concerns | Low risk | No concerns | No concerns | Major concerns | Major concerns | Low | ["Heterogeneity","Incoherence"] |
| **FCM:OFS** | 1 | Some concerns | Low risk | No concerns | Major concerns | No concerns | No concerns | Low | ["Within-study bias","Imprecision"] |
| **FDI:ISC** | 2 | No concerns | Low risk | No concerns | Major concerns | No concerns | No concerns | Moderate | ["Imprecision"] |
| **FDI:OFS** | 2 | No concerns | Low risk | No concerns | Major concerns | No concerns | No concerns | Moderate | ["Imprecision"] |
| **FSC:ISC** | 1 | Some concerns | Low risk | No concerns | Major concerns | No concerns | Major concerns | Very low | ["Within-study bias","Imprecision","Incoherence"] |
| **FXM:ISC** | 1 | Some concerns | Low risk | No concerns | Major concerns | No concerns | Major concerns | Very low | ["Within-study bias","Imprecision","Incoherence"] |
| **FXM:PCB** | 1 | Some concerns | Low risk | No concerns | No concerns | Major concerns | Major concerns | Very low | ["Within-study bias","Heterogeneity","Incoherence"] |
| **ISC:OLS** | 1 | No concerns | Low risk | No concerns | Major concerns | No concerns | Major concerns | Low | ["Imprecision","Incoherence"] |
| **CAI:FDI** | 0 | No concerns | Low risk | No concerns | No concerns | Major concerns | Major concerns | Low | ["Heterogeneity","Incoherence"] |
| **CAI:FSC** | 0 | No concerns | Low risk | No concerns | No concerns | Major concerns | Major concerns | Low | ["Heterogeneity","Incoherence"] |
| **CAI:FXM** | 0 | No concerns | Low risk | No concerns | No concerns | Major concerns | Major concerns | Low | ["Heterogeneity","Incoherence"] |
| **CAI:ISC** | 0 | No concerns | Low risk | No concerns | No concerns | Major concerns | Major concerns | Low | ["Heterogeneity","Incoherence"] |
| **CAI:OFM** | 0 | No concerns | Low risk | No concerns | No concerns | Major concerns | Major concerns | Low | ["Heterogeneity","Incoherence"] |
| **CAI:OFS** | 0 | No concerns | Low risk | No concerns | No concerns | Major concerns | Major concerns | Low | ["Heterogeneity","Incoherence"] |
| **CAI:OLS** | 0 | No concerns | Low risk | No concerns | No concerns | Major concerns | Major concerns | Low | ["Heterogeneity","Incoherence"] |
| **CAI:PCB** | 0 | Some concerns | Low risk | No concerns | Major concerns | No concerns | Major concerns | Very low | ["Within-study bias","Imprecision","Incoherence"] |
| **FCM:FDI** | 0 | No concerns | Low risk | No concerns | Major concerns | No concerns | Major concerns | Very low | ["Imprecision","Incoherence"] |
| **FCM:FSC** | 0 | Some concerns | Low risk | No concerns | Major concerns | No concerns | Major concerns | Very low | ["Within-study bias","Imprecision","Incoherence"] |
| **FCM:FXM** | 0 | Some concerns | Low risk | No concerns | Major concerns | No concerns | Major concerns | Very low | ["Within-study bias","Imprecision","Incoherence"] |
| **FCM:OLS** | 0 | No concerns | Low risk | No concerns | Major concerns | No concerns | Major concerns | Very low | ["Imprecision","Incoherence"] |
| **FCM:PCB** | 0 | Some concerns | Low risk | No concerns | No concerns | Major concerns | Major concerns | Very low | ["Within-study bias","Heterogeneity","Incoherence"] |
| **FDI:FSC** | 0 | Some concerns | Low risk | No concerns | Major concerns | No concerns | Major concerns | Very low | ["Within-study bias","Imprecision","Incoherence"] |
| **FDI:FXM** | 0 | Some concerns | Low risk | No concerns | Major concerns | No concerns | Major concerns | Very low | ["Within-study bias","Imprecision","Incoherence"] |
| **FDI:OFM** | 0 | No concerns | Low risk | No concerns | No concerns | Major concerns | Major concerns | Low | ["Heterogeneity","Incoherence"] |
| **FDI:OLS** | 0 | No concerns | Low risk | No concerns | Major concerns | No concerns | Major concerns | Low | ["Imprecision","Incoherence"] |
| **FDI:PCB** | 0 | Some concerns | Low risk | No concerns | No concerns | Major concerns | Major concerns | Very low | ["Within-study bias","Heterogeneity","Incoherence"] |
| **FSC:FXM** | 0 | Some concerns | Low risk | No concerns | Major concerns | No concerns | Major concerns | Very low | ["Within-study bias","Imprecision","Incoherence"] |
| **FSC:OFM** | 0 | No concerns | Low risk | No concerns | Major concerns | No concerns | Major concerns | Low | ["Imprecision","Incoherence"] |
| **FSC:OFS** | 0 | Some concerns | Low risk | No concerns | Major concerns | No concerns | Major concerns | Very low | ["Within-study bias","Imprecision","Incoherence"] |
| **FSC:OLS** | 0 | Some concerns | Low risk | No concerns | Major concerns | No concerns | Major concerns | Very low | ["Within-study bias","Imprecision","Incoherence"] |
| **FSC:PCB** | 0 | Some concerns | Low risk | No concerns | No concerns | Major concerns | Major concerns | Very low | ["Within-study bias","Heterogeneity","Incoherence"] |
| **FXM:OFM** | 0 | No concerns | Low risk | No concerns | Major concerns | No concerns | Major concerns | Low | ["Imprecision","Incoherence"] |
| **FXM:OFS** | 0 | Some concerns | Low risk | No concerns | Major concerns | No concerns | Major concerns | Very low | ["Within-study bias","Imprecision","Incoherence"] |
| **FXM:OLS** | 0 | Some concerns | Low risk | No concerns | Major concerns | No concerns | Major concerns | Very low | ["Within-study bias","Imprecision","Incoherence"] |
| **ISC:OFM** | 0 | No concerns | Low risk | No concerns | No concerns | Major concerns | Major concerns | Very low | ["Heterogeneity","Incoherence"] |
| **ISC:OFS** | 0 | No concerns | Low risk | No concerns | Major concerns | No concerns | Major concerns | Very low | ["Imprecision","Incoherence"] |
| **ISC:PCB** | 0 | Some concerns | Low risk | No concerns | No concerns | Major concerns | Major concerns | Very low | ["Within-study bias","Heterogeneity","Incoherence"] |
| **OFM:OFS** | 0 | No concerns | Low risk | No concerns | Major concerns | No concerns | Major concerns | Low | ["Imprecision","Incoherence"] |
| **OFM:OLS** | 0 | No concerns | Low risk | No concerns | Major concerns | No concerns | Major concerns | Low | ["Imprecision","Incoherence"] |
| **OFM:PCB** | 0 | Some concerns | Low risk | No concerns | Major concerns | No concerns | Major concerns | Very low | ["Within-study bias","Imprecision","Incoherence"] |
| **OFS:OLS** | 0 | No concerns | Low risk | No concerns | Major concerns | No concerns | Major concerns | Low | ["Imprecision","Incoherence"] |
| **OFS:PCB** | 0 | Some concerns | Low risk | No concerns | No concerns | Major concerns | Major concerns | Very low | ["Within-study bias","Heterogeneity","Incoherence"] |
| **OLS:PCB** | 0 | Some concerns | Low risk | No concerns | No concerns | Major concerns | Major concerns | Very low | ["Within-study bias","Heterogeneity","Incoherence"] |

**CINeMA – Hb – 5 week – Overall analysis**

| **Comparison** | **Number of studies** | **Within-study bias** | **Reporting bias** | **Indirectness** | **Imprecision** | **Heterogeneity** | **Incoherence** | **Confidence rating** | **Reason (s) for downgrading** |
| --- | --- | --- | --- | --- | --- | --- | --- | --- | --- |
| **FCM:FDI** | 3 | No concerns | Low risk | No concerns | No concerns | Major concerns | Major concerns | Low | ["Heterogeneity","Incoherence"] |
| **FCM:FXM** | 1 | No concerns | Low risk | No concerns | No concerns | Major concerns | Major concerns | Low | ["Heterogeneity","Incoherence"] |
| **FCM:IVSC** | 1 | Major concerns | Low risk | No concerns | No concerns | No concerns | Major concerns | Low | ["Within-study bias","Incoherence"] |
| **FCM:OFS** | 1 | Major concerns | Low risk | No concerns | No concerns | No concerns | Major concerns | Low | ["Within-study bias","Incoherence"] |
| **FDI:ISC** | 1 | Some concerns | Low risk | No concerns | Major concerns | No concerns | Major concerns | Very low | ["Within-study bias","Imprecision","Incoherence"] |
| **FFM:FXM** | 1 | No concerns | Low risk | No concerns | No concerns | Major concerns | Major concerns | Low | ["Heterogeneity","Incoherence"] |
| **FXM:ISC** | 3 | Some concerns | Low risk | No concerns | No concerns | Major concerns | Major concerns | Very low | ["Within-study bias","Heterogeneity","Incoherence"] |
| **FXM:PCB** | 1 | No concerns | Low risk | No concerns | No concerns | No concerns | Major concerns | Moderate | ["Incoherence"] |
| **FCM:FFM** | 0 | No concerns | Low risk | No concerns | No concerns | No concerns | Major concerns | Moderate | ["Incoherence"] |
| **FCM:ISC** | 0 | Some concerns | Low risk | No concerns | No concerns | Major concerns | Major concerns | Very low | ["Within-study bias","Heterogeneity","Incoherence"] |
| **FCM:PCB** | 0 | No concerns | Low risk | No concerns | No concerns | No concerns | Major concerns | Moderate | ["Incoherence"] |
| **FDI:FFM** | 0 | No concerns | Low risk | No concerns | Major concerns | No concerns | Major concerns | Low | ["Imprecision","Incoherence"] |
| **FDI:FXM** | 0 | Some concerns | Low risk | No concerns | Major concerns | No concerns | Major concerns | Very low | ["Within-study bias","Imprecision","Incoherence"] |
| **FDI:IVSC** | 0 | Some concerns | Low risk | No concerns | Major concerns | No concerns | Major concerns | Very low | ["Within-study bias","Imprecision","Incoherence"] |
| **FDI:OFS** | 0 | Some concerns | Low risk | No concerns | No concerns | Major concerns | Major concerns | Very low | ["Within-study bias","Heterogeneity","Incoherence"] |
| **FDI:PCB** | 0 | No concerns | Low risk | No concerns | No concerns | No concerns | Major concerns | Moderate | ["Incoherence"] |
| **FFM:ISC** | 0 | No concerns | Low risk | No concerns | Major concerns | No concerns | Major concerns | Low | ["Imprecision","Incoherence"] |
| **FFM:IVSC** | 0 | Some concerns | Low risk | No concerns | Major concerns | No concerns | Major concerns | Very low | ["Within-study bias","Imprecision","Incoherence"] |
| **FFM:OFS** | 0 | Some concerns | Low risk | No concerns | Major concerns | No concerns | Major concerns | Very low | ["Within-study bias","Imprecision","Incoherence"] |
| **FFM:PCB** | 0 | No concerns | Low risk | No concerns | No concerns | No concerns | Major concerns | Moderate | ["Incoherence"] |
| **FXM:IVSC** | 0 | Some concerns | Low risk | No concerns | Major concerns | No concerns | Major concerns | Very low | ["Within-study bias","Imprecision","Incoherence"] |
| **FXM:OFS** | 0 | Some concerns | Low risk | No concerns | No concerns | Major concerns | Major concerns | Very low | ["Within-study bias","Incoherence"] |
| **ISC:IVSC** | 0 | Some concerns | Low risk | No concerns | Major concerns | No concerns | Major concerns | Very low | ["Within-study bias","Imprecision","Incoherence"] |
| **ISC:OFS** | 0 | Some concerns | Low risk | No concerns | No concerns | Major concerns | Major concerns | Very low | ["Within-study bias","Incoherence"] |
| **ISC:PCB** | 0 | No concerns | Low risk | No concerns | No concerns | No concerns | Major concerns | Moderate | ["Incoherence"] |
| **IVSC:OFS** | 0 | Major concerns | Low risk | No concerns | Major concerns | No concerns | Major concerns | Very low | ["Within-study bias","Imprecision","Incoherence"] |
| **IVSC:PCB** | 0 | Some concerns | Low risk | No concerns | No concerns | No concerns | Major concerns | Low | ["Within-study bias","Incoherence"] |
| **OFS:PCB** | 0 | Some concerns | Low risk | No concerns | No concerns | No concerns | Major concerns | Low | ["Within-study bias","Incoherence"] |

**CINeMA – Hb – 6 week – Overall analysis**

| **Comparison** | **Number of studies** | **Within-study bias** | **Reporting bias** | **Indirectness** | **Imprecision** | **Heterogeneity** | **Incoherence** | **Confidence rating** | **Reason (s) for downgrading** |
| --- | --- | --- | --- | --- | --- | --- | --- | --- | --- |
| **FCM:FDI** | 1 | No concerns | Low risk | No concerns | Major concerns | No concerns | No concerns | Moderate | ["Imprecision"] |
| **FCM:ISC** | 2 | No concerns | Low risk | No concerns | Major concerns | No concerns | No concerns | Moderate | ["Imprecision"] |
| **FCM:OFS** | 2 | Some concerns | Low risk | No concerns | Major concerns | No concerns | Major concerns | Very low | ["Within-study bias","Imprecision","Incoherence"] |
| **FCM:PCB** | 3 | Some concerns | Low risk | No concerns | No concerns | No concerns | Some concerns | Low | ["Within-study bias","Incoherence"] |
| **FDI:ISC** | 1 | No concerns | Low risk | No concerns | Major concerns | No concerns | No concerns | Moderate | ["Imprecision"] |
| **ISC:OFS** | 3 | No concerns | Low risk | No concerns | Major concerns | No concerns | Major concerns | Low | ["Imprecision","Incoherence"] |
| **OFS:SGC** | 1 | Some concerns | Low risk | No concerns | Major concerns | No concerns | Some concerns | Very low | ["Within-study bias","Imprecision","Incoherence"] |
| **FCM:SGC** | 0 | Some concerns | Low risk | No concerns | Major concerns | No concerns | Some concerns | Very low | ["Within-study bias","Imprecision","Incoherence"] |
| **FDI:OFS** | 0 | No concerns | Low risk | No concerns | Major concerns | No concerns | Some concerns | Low | ["Imprecision","Incoherence"] |
| **FDI:PCB** | 0 | No concerns | Low risk | No concerns | No concerns | No concerns | Some concerns | Moderate | ["Incoherence"] |
| **FDI:SGC** | 0 | Some concerns | Low risk | No concerns | Major concerns | No concerns | Some concerns | Very low | ["Within-study bias","Imprecision","Incoherence"] |
| **ISC:PCB** | 0 | Some concerns | Low risk | No concerns | No concerns | No concerns | Some concerns | Low | ["Within-study bias","Incoherence"] |
| **ISC:SGC** | 0 | Some concerns | Low risk | No concerns | Major concerns | No concerns | Some concerns | Very low | ["Within-study bias","Imprecision","Incoherence"] |
| **OFS:PCB** | 0 | Some concerns | Low risk | No concerns | No concerns | No concerns | Some concerns | Low | ["Within-study bias","Incoherence"] |
| **PCB:SGC** | 0 | Some concerns | Low risk | No concerns | No concerns | Major concerns | Some concerns | Very low | ["Within-study bias","Heterogeneity","Incoherence"] |

**CINeMA – Hb – 8 week – Overall analysis**

| **Comparison** | **Number of studies** | **Within-study bias** | **Reporting bias** | **Indirectness** | **Imprecision** | **Heterogeneity** | **Incoherence** | **Confidence rating** | **Reason (s) for downgrading** |
| --- | --- | --- | --- | --- | --- | --- | --- | --- | --- |
| **CTL:FCM** | 1 | No concerns | Low risk | No concerns | Major concerns | No concerns | No concerns | Moderate | ["Imprecision"] |
| **FCM:ISC** | 2 | Some concerns | Low risk | No concerns | Major concerns | No concerns | No concerns | Low | ["Within-study bias","Imprecision"] |
| **FCM:OFS** | 1 | Some concerns | Low risk | No concerns | Major concerns | No concerns | No concerns | Low | ["Within-study bias","Imprecision"] |
| **FCM:SUI** | 1 | No concerns | Low risk | No concerns | No concerns | No concerns | No concerns | High | [] |
| **FDI:ISC** | 3 | Some concerns | Low risk | No concerns | Major concerns | No concerns | No concerns | Low | ["Within-study bias","Imprecision"] |
| **FDI:OFS** | 2 | No concerns | Low risk | No concerns | Major concerns | No concerns | No concerns | Moderate | ["Imprecision"] |
| **FSC:ISC** | 1 | Some concerns | Low risk | No concerns | No concerns | No concerns | No concerns | Moderate | ["Within-study bias"] |
| **ISC:OFS** | 2 | No concerns | Low risk | No concerns | Major concerns | No concerns | No concerns | Moderate | ["Imprecision"] |
| **ISC:OLS** | 1 | No concerns | Low risk | No concerns | No concerns | Major concerns | No concerns | Moderate | ["Heterogeneity"] |
| **CTL:FDI** | 0 | Some concerns | Low risk | No concerns | Major concerns | No concerns | No concerns | Low | ["Within-study bias","Imprecision"] |
| **CTL:FSC** | 0 | Some concerns | Low risk | No concerns | Major concerns | No concerns | No concerns | Low | ["Within-study bias","Imprecision"] |
| **CTL:ISC** | 0 | Some concerns | Low risk | No concerns | Major concerns | No concerns | No concerns | Low | ["Within-study bias","Imprecision"] |
| **CTL:OFS** | 0 | No concerns | Low risk | No concerns | Major concerns | No concerns | No concerns | Moderate | ["Imprecision"] |
| **CTL:OLS** | 0 | No concerns | Low risk | No concerns | Major concerns | No concerns | No concerns | Moderate | ["Imprecision"] |
| **CTL:SUI** | 0 | No concerns | Low risk | No concerns | No concerns | No concerns | No concerns | High | [] |
| **FCM:FDI** | 0 | Some concerns | Low risk | No concerns | Major concerns | No concerns | No concerns | Low | ["Within-study bias","Imprecision"] |
| **FCM:FSC** | 0 | Some concerns | Low risk | No concerns | No concerns | No concerns | No concerns | Moderate | ["Within-study bias"] |
| **FCM:OLS** | 0 | Some concerns | Low risk | No concerns | No concerns | Major concerns | No concerns | Low | ["Within-study bias","Heterogeneity"] |
| **FDI:FSC** | 0 | Some concerns | Low risk | No concerns | No concerns | No concerns | No concerns | Moderate | ["Within-study bias"] |
| **FDI:OLS** | 0 | No concerns | Low risk | No concerns | No concerns | No concerns | No concerns | High | [] |
| **FDI:SUI** | 0 | Some concerns | Low risk | No concerns | No concerns | No concerns | No concerns | Moderate | ["Within-study bias"] |
| **FSC:OFS** | 0 | Some concerns | Low risk | No concerns | No concerns | No concerns | No concerns | Moderate | ["Within-study bias"] |
| **FSC:OLS** | 0 | Some concerns | Low risk | No concerns | Major concerns | No concerns | No concerns | Low | ["Within-study bias","Imprecision"] |
| **FSC:SUI** | 0 | Some concerns | Low risk | No concerns | No concerns | No concerns | No concerns | Moderate | ["Within-study bias"] |
| **ISC:SUI** | 0 | Some concerns | Low risk | No concerns | No concerns | No concerns | No concerns | Moderate | ["Within-study bias"] |
| **OFS:OLS** | 0 | No concerns | Low risk | No concerns | No concerns | No concerns | No concerns | High | [] |
| **OFS:SUI** | 0 | No concerns | Low risk | No concerns | No concerns | No concerns | No concerns | High | [] |
| **OLS:SUI** | 0 | No concerns | Low risk | No concerns | No concerns | No concerns | No concerns | High | [] |

**GRADE**

**Outcome Hb**

| **Comparison** | **Number of studies** | **Study bias** | **Inconsistency** | **Indirectness** | **Imprecision** | **Publication Bias** | **Confidence rating** |
| --- | --- | --- | --- | --- | --- | --- | --- |
| **FCM:FDI – 10w** | 1 | Not serious | Not serious | Not serious | Serious* | Not serious | Moderate |

**Outcome Ferritin**

| **Comparison** | **Number of studies** | **Study bias** | **Inconsistency** | **Indirectness** | **Imprecision** | **Publication Bias** | **Confidence rating** |
| --- | --- | --- | --- | --- | --- | --- | --- |
| **FCM:FDI – 7w** | 1 | Not serious | Not serious | Not serious | Serious* | Not serious | Moderate |
| **FCM:FDI – 10w** | 1 | Not serious | Not serious | Not serious | Serious* | Not serious | Moderate |
| **ISC:OFS – 20w** | 1 | Not serious | Not serious | Not serious | Serious* | Not serious | Moderate |

**Transferrin Saturation**

| **Comparison** | **Number of studies** | **Study bias** | **Inconsistency** | **Indirectness** | **Imprecision** | **Publication Bias** | **Confidence rating** |
| --- | --- | --- | --- | --- | --- | --- | --- |
| **FCM:FDI – 7w** | 1 | Not serious | Not serious | Not serious | Serious* | Not serious | Moderate |
| **FCM:FDI – 10w** | 1 | Not serious | Not serious | Not serious | Serious* | Not serious | Moderate |

**Legend: * CI95% large.**

# Supplement 4 – Studies excluded.

| **Studies excluded** |
| --- |
| 1. Barish CF, Koch T, Butcher A, Morris D, Bregman DB. Safety and Efficacy of Intravenous Ferric Carboxymaltose (750 mg) in the Treatment of Iron Deficiency Anemia: Two Randomized, Controlled Trials. Anemia. 2012:172104. doi: 10.1155/2012/172104. Epub 2012 Sep 10. PMID: 22997572; PMCID: PMC3444829. |
| 1. Choi KY, Koh IJ, Kim MS, Kim C, In Y. Intravenous Ferric Carboxymaltose Improves Response to Postoperative Anemia Following Total Knee Arthroplasty: A Prospective Randomized Controlled Trial in Asian Cohort. J Clin Med. 2022 Apr 22;11 (9) :2357. doi: 10.3390/jcm11092357. PMID: 35566482; PMCID: PMC9103711. |
| 1. Edwards TJ, Noble EJ, Durran A, Mellor N, Hosie KB. Randomized clinical trial of preoperative intravenous iron sucrose to reduce blood transfusion in anaemic patients after colorectal cancer surgery. Br J Surg. 2009 Oct;96 (10) :1122-8. doi: 10.1002/bjs.6688. PMID: 19731228. |
| 1. Assouline B, Benoliel A, Zamberg I, Legouis D, Delhumeau C, Favre M, Andrès A, Toso C, Samii K, Schiffer E. Intravenous iron supplementation after liver surgery: Impact on anemia, iron, and hepcidin levels-a randomized controlled trial. Surgery. 2021 Sep;170 (3) :813-821. doi: 10.1016/j.surg.2021.03.020. Epub 2021 Apr 20. PMID: 33888314. |
| 1. Xu H, Duan Y, Yuan X, Wu H, Sun H, Ji H. Intravenous Iron Versus Placebo in the Management of Postoperative Functional Iron Deficiency Anemia in Patients Undergoing Cardiac Valvular Surgery: A Prospective, Single-Blinded, Randomized Controlled Trial. J Cardiothorac Vasc Anesth. 2019 Nov;33 (11) :2941-2948. doi: 10.1053/j.jvca.2019.01.063. Epub 2019 Feb 8. PMID: 30930140. |
| 1. Froessler B, Palm P, Weber I, Hodyl NA, Singh R, Murphy EM. The Important Role for Intravenous Iron in Perioperative Patient Blood Management in Major Abdominal Surgery: A Randomized Controlled Trial. Ann Surg. 2016 Jul;264 (1) :41-6. doi: 10.1097/SLA.0000000000001646. PMID: 26817624; PMCID: PMC4902320. |
| 1. Garrido-Martín P, Nassar-Mansur MI, de la Llana-Ducrós R, Virgos-Aller TM, Rodríguez Fortunez PM, Ávalos-Pinto R, Jimenez-Sosa A, Martínez-Sanz R. The effect of intravenous and oral iron administration on perioperative anaemia and transfusion requirements in patients undergoing elective cardiac surgery: a randomized clinical trial. Interact Cardiovasc Thorac Surg. 2012 Dec;15 (6) :1013-8. doi: 10.1093/icvts/ivs344. Epub 2012 Aug 31. PMID: 22940889; PMCID: PMC3501294. |
| 1. Keeler BD, Simpson JA, Ng O, Padmanabhan H, Brookes MJ, Acheson AG; IVICA Trial Group. Randomized clinical trial of preoperative oral versus intravenous iron in anaemic patients with colorectal cancer. Br J Surg. 2017 Feb;104 (3) :214-221. doi: 10.1002/bjs.10328. Epub 2017 Jan 16. PMID: 28092401. |
| 1. Khalafallah AA, Yan C, Al-Badri R, Robinson E, Kirkby BE, Ingram E, Gray Z, Khelgi V, Robertson IK, Kirkby BP. Intravenous ferric carboxymaltose versus standard care in the management of postoperative anaemia: a prospective, open-label, randomised controlled trial. Lancet Haematol. 2016 Sep;3 (9) :e415-25. doi: 10.1016/S2352-3026 (16) 30078-3. Epub 2016 Aug 4. PMID: 27570088. |
| 1. Kim HH, Park EH, Lee SH, Yoo KJ, Youn YN. Effect of Preoperative Administration of Intravenous Ferric Carboxymaltose in Patients with Iron Deficiency Anemia after Off-Pump Coronary Artery Bypass Grafting: A Randomized Controlled Trial. J Clin Med. 2023 Feb 21;12 (5) :1737. doi: 10.3390/jcm12051737. PMID: 36902524; PMCID: PMC10003059. |
| 1. Kvaslerud AB, Bardan S, Andresen K, Kløve SF, Fagerland MW, Edvardsen T, Gullestad L, Broch K. Intravenous iron supplement for iron deficiency in patients with severe aortic stenosis scheduled for transcatheter aortic valve implantation: results of the IIISAS randomised trial. Eur J Heart Fail. 2022 Jul;24 (7) :1269-1279. doi: 10.1002/ejhf.2557. Epub 2022 Jun 3. PMID: 35579454; PMCID: PMC9544901. |
| 1. Laso-Morales MJ, Vives R, Bisbe E, García-Erce JA, Muñoz M, Martínez-López F, Carol-Boeris F, Pontes-García C. Single-dose intravenous ferric carboxymaltose infusion versus multiple fractionated doses of intravenous iron sucrose in the treatment of post-operative anaemia in colorectal cancer patients: a randomised controlled trial. Blood Transfus. 2022 Jul;20 (4) :310-318. doi: 10.2450/2021.0157-21. Epub 2021 Oct 15. PMID: 34694223; PMCID: PMC9256505. |
| 1. Lee S, Ryu KJ, Lee ES, Lee KH, Lee JJ, Kim T. Comparative efficacy and safety of intravenous ferric carboxymaltose and iron sucrose for the treatment of preoperative anemia in patients with menorrhagia: An open-label, multicenter, randomized study. J Obstet Gynaecol Res. 2019 Apr;45 (4) :858-864. doi: 10.1111/jog.13893. PMID: 30932300. |
| 1. Park HS, Kim TY, Kim HJ, Ro YJ, Jang HY, Koh WU. The Effect of Intraoperative Ferric Carboxymaltose in Joint Arthroplasty Patients: A Randomized Trial. J Clin Med. 2019 Oct 13;8 (10) :1674. doi: 10.3390/jcm8101674. PMID: 31614940; PMCID: PMC6832232. |
| 1. Song JW, Soh S, Shim JK, Lee S, Lee SH, Kim HB, Kim MY, Kwak YL. Effect of Perioperative Intravenous Iron Supplementation for Complex Cardiac Surgery on Transfusion Requirements: A Randomized, Double-blinded Placebo-controlled Trial. Ann Surg. 2022 Feb 1;275 (2) :232-239. doi: 10.1097/SLA.0000000000005011. PMID: 34171864. |
| 1. Auerbach M, Achebe MM, Thomsen LL, Derman RJ. Efficacy and safety of ferric derisomaltose (FDI) compared with iron sucrose (IS) in patients with iron deficiency anemia after bariatric surgery. Obes Surg. 2022 Mar;32 (3) :810-818. doi: 10.1007/s11695-021-05858-0. Epub 2022 Jan 8. PMID: 35000068; PMCID: PMC8866325. |
| 1. Bisbe E, Moltó L, Arroyo R, Muniesa JM, Tejero M. Randomized trial comparing ferric carboxymaltose vs oral ferrous glycine sulphate for postoperative anaemia after total knee arthroplasty. Br J Anaesth. 2014 Sep;113 (3) :402-9. doi: 10.1093/bja/aeu092. Epub 2014 Apr 29. PMID: 24780615. |
| 1. Borstlap WAA, Buskens CJ, Tytgat KMAJ, Tuynman JB, Consten ECJ, Tolboom RC, Heuff G, van Geloven N, van Wagensveld BA, C A Wientjes CA, Gerhards MF, de Castro SMM, Jansen J, van der Ven AWH, van der Zaag E, Omloo JM, van Westreenen HL, Winter DC, Kennelly RP, Dijkgraaf MGW, Tanis PJ, Bemelman WA. Multicentre randomized controlled trial comparing ferric (III) carboxymaltose infusion with oral iron supplementation in the treatment of preoperative anaemia in colorectal cancer patients. BMC Surg. 2015 Jun 28;15:78. doi: 10.1186/s12893-015-0065-6. Erratum in: BMC Surg. 2015 Oct 08;15:110. doi: 10.1186/s12893-015-0090-5. van Geloven, N [corrected to van Geloven, A A W]. PMID: 26123286; PMCID: PMC4485873. |
| 1. Covic A, Mircescu G. The safety and efficacy of intravenous ferric carboxymaltose in anaemic patients undergoing haemodialysis: a multi-centre, open-label, clinical study. Nephrol Dial Transplant. 2010 Aug;25 (8) :2722-30. doi: 10.1093/ndt/gfq069. Epub 2010 Feb 26. PMID: 20190247; PMCID: PMC2905444. |
| 1. Ray R, Ford I, Cleland JGF, Graham F, Ahmed FZ, Al-Mohammad A, Cowburn PJ, Critoph C, Kalra PA, Lane RE, Ludman A, Pellicori P, Petrie MC, Robertson M, Seed A, Squire I, Kalra PR; INVESTIGATORS; CLINICAL TRIALS UNIT AND DATA AND STATISTICAL CENTRE; NURSING AND ADMINISTRATIVE SUPPORT; SPONSOR SUPPORT. The Impact of Ferric Derisomaltose on Cardiovascular and Noncardiovascular Events in Patients With Anemia, Iron Deficiency, and Heart Failure With Reduced Ejection Fraction. J Card Fail. 2024 May;30 (5) :682-690. doi: 10.1016/j.cardfail.2023.10.006. Epub 2023 Nov 4. PMID: 37926238; PMCID: PMC11096866. |

# Supplement 5 - Reported outcomes by study

| **1st Author** | **Year** | **AAE** | **SAE** | **Hypo** | **Hb** | **TSAT** | **Ferr** |
| --- | --- | --- | --- | --- | --- | --- | --- |
| Adkinson | 2018 | X | X |  | X | X | X |
| Agarwal | 2006 | X | X |  | X | X | X |
| Agarwal | 2015 |  | X |  | X | X | X |
| Ambrosy | 2021 | X | X | X | X | X | X |
| Anirban | 2008 | X | X |  | X | X | X |
| Anker | 2009 |  |  |  | X | X | X |
| Auerbach | 2019 | X | X | X | X | X | X |
| Bae | 2023 |  |  |  | X | X | X |
| Bailie | 2010 | X |  | X |  |  |  |
| Beck-da-Silva | 2014 |  |  |  |  |  | X |
| Bertani | 2021 |  |  |  | X |  | X |
| Bhandari | 2015 | X | X | X | X | X | X |
| Birgegard | 2016 | X | X |  | X | X | X |
| Boomershine | 2018 | X |  |  | X | X | X |
| Charytan | 2013 | X | X |  | X | X | X |
| Derman | 2017 | X | X |  | X | X | X |
| Dhoot | 2020 |  | X |  | X | X | X |
| Emrich | 2020 |  |  | X | X | X | X |
| Evstatiev | 2011 | X | X | X | X | X | X |
| Ferrer-Barcelo | 2019 |  |  |  | X | X |  |
| Ford | 2016 | X | X |  | X | X | X |
| Gybel-Brask | 2018 | X | X |  | X | X | X |
| Hedenus | 2014 | X | X |  | X | X | X |
| Hetzel | 2014 | X | X |  | X | X | X |
| Howaldt | 2022 | X | X |  | X |  | X |
| Howard | 2022 |  |  |  | X | X | X |
| Ikuta | 2019 | X |  |  | X | X | X |
| Jin | 2024 | X | X | X | X | X | X |
| Kalra | 2016 | X | X | X | X | X | X |
| Kalra | 2022 |  | X |  | X | X | X |
| Kulnigg | 2008 | X | X |  | X | X | X |
| Li | 2008 |  |  |  | X | X | X |
| Lindgreen | 2009 |  |  |  |  | X |  |
| Macdougall | 2014b | X | X |  | X | X | X |
| Macdougall | 2019 | X | X |  | X | X | X |
| Macdougall | 2014a | X | X |  | X | X | X |
| Mahey | 2016 |  |  | X | X | X | X |
| Noronha | 2017 |  |  |  | X |  |  |
| Onken | 2013 | X | X | X | X | X | X |
| Onken | 2014 | X | X | X | X | X | X |
| Patel | 2024 |  |  |  | X |  |  |
| Pieracci | 2014 |  |  |  | X | X | X |
| Provenzano | 2009 | X | X |  | X | X | X |
| Qunibi | 2010 |  | X |  | X | X | X |
| Reinisch | 2013 | X | X |  | X | X | X |
| Roger | 2017 | X | X |  |  |  |  |
| Saroj Vadhan | 2014 | X | X |  | X | X |  |
| Schroder | 2005 | X |  |  | X | X | X |
| Tabish | 2024 |  |  | X | X |  |  |
| Van Wyck | 2005 |  |  |  | X | X | X |
| Waziri | 2016 | X |  |  | X | X | X |
| Wolf | 2018 |  |  | X | X | X | X |
| Wolf | 2020C1 | X |  | X | X | X | X |
| Wolf | 2020C2 | X |  | X | X | X | X |
| Zahr | 2024 |  |  |  | X | X | X |
| Zoller | 2023 | X | X | X | X | X | X |

**Legend:** AAE – Any adverse event, SAE – Serious Adverse Event, Hypo – Hypophosphatemia, Hb – Hemoglobin, TSAT – Transferrin Saturation, Ferr – Ferritin.

# Supplement 6 - Studies’ characteristics

| **1^st^ Author** | **Year** | **Title** | **Trial name and/ or Trial number** | **Study design** | **Randomization** | **Blinding** | **Study setting** | **Inclusion criteria** | **Arms** | **Dose** | **Frequency of administration** |
| --- | --- | --- | --- | --- | --- | --- | --- | --- | --- | --- | --- |
| Adkinson | 2018 | Comparative safety of intravenous ferumoxytol versus ferric carboxymaltose in iron deficiency anemia: a randomized trial | NCT02694978 | Phase 3, randomized, multicenter | Centralized interactive web response system | Double-blind | Multicenter | 18 years with IDA (hemoglobin <12.0 g/dL for women and <14.0 g/dL for men and transferrin saturation 20% or ferritin 100 ng/mL within 60 days of dosing) , and a history of unsatisfactory oral iron therapy or intolerance, or whom oral iron was considered medically inappropriate | FXM | 510 mg | Day 1, 7 to 8 days later for a total cumulative dose of 1.02 g |
|  |  |  |  |  |  |  |  |  | FCM | 750 mg | Day 1, 7 to 8 days later for a total cumulative dose of 1.50 g |
| Agarwal | 2006 | A Randomized Controlled Trial of Oral versus Intravenous Iron in Chronic Kidney Disease | - | Randomized, controlled, multicenter | Computer-generated randomization schedule | Open-label | Multicenter | Adult (≥18 years) anemic, iron-deficient non-dialysis CKD (ND-CKD) patients (6 stage 3) not receiving erythropoiesis-stimulating agents (ESAs) . | SGC | 250 mg | Once weekly |
|  |  |  |  |  |  |  |  |  | OFS | 325 mg | 6 consecutive weeks |
| Agarwal | 2015 | A randomized trial of intravenous and oral iron in chronic kidney disease | REVOKE | Single center randomized trial | Permuted blocks | Open-label | Single center | Adult (≥18 years) have an estimated GFR (eGFR) by the 4-component MDRD formula of >20 and ≤ 60 ml/min/1.73m2 using IDMS-calibrated creatinine [32], anemia and iron deficiency. | ISC | 200 mg | 0, 2, 4, 6, and 8 weeks |
|  |  |  |  |  |  |  |  |  | OFS | 325 mg | 8 consecutive weeks |
| Ambrosy | 2021 | Safety and Efficacy of Intravenous Ferric Derisomaltose Compared to Iron Sucrose for Iron Deficiency Anemia in Patients with Chronic Kidney Disease With and Without Heart Failure | FERWON-NEPHRO NCT02940860 | Randomized,  multicenter trial | According to the product label | Open-label | Multicenter | Non-dialysis-dependent chronic kidney disease (NDD-CKD) Iron deficiency anemia; Hemoglobin ≤11 g/dL; Serum ferritin ≤100 ng/mL (or ≤300 ng/mL if TSAT ≤30%) ; Stable dose of erythropoiesis-stimulating agents for 4 weeks prior to randomization (if used) | FDI | 1000 mg | Single dose |
|  |  |  |  |  |  |  |  |  | ISC | 200 mg | Up to 5 times within the first 2 weeks |
|  |  |  |  |  |  |  |  |  | FDI | 1000 mg | Single dose |
|  |  |  |  |  |  |  |  |  | ISC | 200 mg | Up to 5 times within the first 2 weeks |
| Anirban | 2008 | The comparative safety of various intravenous iron preparations in chronic kidney disease patients | NI | Head-to–head, prospective, randomized | Computer-generated numbers | Open-label | Single center | Adult CKD patients either on conservative management or on renal replacement therapy; provided written consent to be included in the study; discontinued oral iron use during the study period | IDX | 100 mg | Twice a week |
|  |  |  |  |  |  |  |  |  | SGC | 125 mg | Once a week |
|  |  |  |  |  |  |  |  |  | ISC | 100 mg | Twice a week |
| Anker | 2009 | Ferric carboxymaltose in patients with heart failure and iron deficiency | FAIR-HF study NCT00520780 | Randomised, Controlled,  Phase III Study | Central interactive voice-response system | Double-blind | Multicenter | Chronic heart failure NYHA class II or III; Left ventricular ejection fraction ≤40% (NYHA II) or 45% (NYHA III) ; Iron deficiency (ferritin <100 μg/L or 100-299 μg/L with transferrin saturation <20%) ; Hemoglobin level 95-135 g/L; ambulatory status | FCM | 200 mg | Weekly during correction phase, monthly during maintenance phase |
|  |  |  |  |  |  |  |  |  | PCB | 4ml Saline Solution | Weekly during correction phase, monthly during maintenance phase |
| Auerbach | 2019 | A prospective, multi-center, randomized comparison of iron isomaltoside 1000 versus iron sucrose in patients with iron deficiency anemia; the FERWON-IDA trial | FERWON-IDA NCT02940886 | Multicenter and randomized | Stratified block randomization | Open-label | Multicenter | ≥18 years with IDA of different etiologies, and had intolerance or lack of response to oral iron or screening hemoglobin (Hb) , with Hb ≤11 g/dL, transferrin saturation (TSAT) <20%, and s-ferritin <100 ng/mL | FDI | 1000 mg | Single dose |
|  |  |  |  |  |  |  |  |  | ISC | 200 mg | Five times |
| Bae | 2023 | Ferric carboxymaltose effects on restless legs syndrome and on brain iron in patients with iron deficiency anemia | NI | Randomized, placebo-controlled | NR | Double-blind | Single center | Over 18 years old; diagnosed with IDA (Iron Deficiency Anemia) ; Diagnosed with RLS (Restless Legs Syndrome) ; RLS symptoms more than three times per week; IRLS score over 15; Off all RLS medications for more than 14 days prior to baseline assessment | FCM | 1500 mg | Twice |
|  |  |  |  |  |  |  |  |  | PCB | 100 ml | Twice |
| Bailie | 2010 | Safety and tolerability of intravenous ferric carboxymaltose in patients with iron deficiency anemia | - | Phase 3, multicenter, randomized,noninferiorityand crossover study | NR | Double-blind | Multicenter | Adult (≥18 years) anemic, iron-deficiency patients | FCM | 15 mg/kg (maximum dose of 1000 mg) | Day 0.On day 7, subjects were crossed over |
|  |  |  |  |  |  |  |  |  | PCB | - | Day 0.On day 7, subjects were crossed over |
| Beck-da-Silva | 2013 | IRON-HF study: A randomized trial to assess the effects of iron in heart failure patients with anemia | IRON-HF | Randomized, placebo controlled clinical trial | NR | Double-blind | Multicenter | Adult (≥18 years) Stable ambulatory HF patients, with ejection fraction below 40% who were anemic by the World Health Organization (WHO) criteria | ISC | 200 mg | Once a week, for 5 weeks and placebo of oral presentation, three times a day, for 8 weeks. |
|  |  |  |  |  |  |  |  |  | OFS | 200 mg | Three times a day, for 8 weeks and placebo of IV presentation once a week, for 5 weeks. |
|  |  |  |  |  |  |  |  |  | PCB | - | Three times a day, for 8 weeks and placebo of IV presentation once a week, for 5 weeks. |
| Bertani | 2021 | Oral Sucrosomial Iron Is as Effective as Intravenous Ferric Carboxy-Maltose in Treating Anemia in Patients with Ulcerative Colitis | - | Prospective, randomized study | Computer-generated random | Open-label | Single center | Patients, who: (1) Were affected by UC in remission; (2) were 18-year older; (3) had mild-to-moderate anemia related to iron deficiency; (4) agreed to sign the informed consent to participate in the study. | FCM | 1000 mg | Unclear |
|  |  |  |  |  |  |  |  |  | SUI | 60 mg after 30 mg | 8 weeks since baseline and then 30 mg for 4 weeks |
| Bhandari | 2015 | A randomized, open-label trial of iron isomaltoside 1000 (Monofer®) compared with iron sucrose (Venofer®)  as maintenance therapy in haemodialysis patients | NCT01222884 | Randomized, non-inferiority trial | Interactive web response system | Open-label | Multicenter | Subjects ≥18 years of age with a diagnosis of CKD and on haemodialysis therapy for at least 90 days Hb concentration between 9.5 and 12.5 g/dL (inclusive both values) both at screening visit 1a and screening visit 1b (screening visits were separated by at least 1 week) , Serum-ferritin <800 ng/mL, TSAT <35%, erythropoiesis stimulating agent treatment with dose stable for the previous 4 weeks prior to screening, no IV iron or an average of no >100 mg/week for the previous 4 weeks, life expectancy beyond 12 months | FDI | 500 mg | Once |
|  |  |  |  |  |  |  |  |  | ISC | 500 mg | Once |
| Birgegard | 2016 | A Randomized Noninferiority Trial of Intravenous Iron Isomaltoside versus Oral Iron Sulfate in Patients with Nonmyeloid Malignancies and Anemia Receiving Chemotherapy: The PROFOUND Trial | PROFOUND TRIAL NCT0114-5638 | Randomized, non-inferiority trial | Stratified block randomization methodology | Open-label | Multicenter | Adult (≥18 years) diagnosed with nonmyeloid malignancies, receiving chemotherapy (at least 1 day) prior to screening and had at least two more chemotherapy cycles planned, and had a Hb level lower than 12.0 g/dl, TSAT less than 50%, serum ferritin level lower than 800 lg/L | FDI | Calculated by onbody weight and Hb | 1–4 doses at weekly intervals) |
|  |  |  |  |  |  |  |  |  | OFS | 200 mg | Daily for 12 weeks with 200 mg given as 100 mg twice/day |
| Boomershine | 2018 | A Blinded, Randomized, Placebo-Controlled Study to Investigate the Efficacy and Safety of Ferric Carboxymaltose in Iron-Deficient Patients with Fibromyalgia | NCT02409459 | Randomized, Placebo-Controlled Study | Electronic data capture system | Double-blind | Single center | Men and women ≥ 18 years old provided they had a diagnosis of fibromyalgia, score ≥ 60 on the FIQR and used narcotics for ≥ 30 days. | FCM | 1500 mg | Maximum of 2 doses |
|  |  |  |  |  |  |  |  |  | PCB | 15 cc Saline Solution | Maximum of 2 doses |
| Charytan | 2013 | Intravenous ferric carboxymaltose versus standard medical care in the treatment of iron deficiency anemia in patients with chronic kidney disease: a randomized, active-controlled, multi-center study | NCT00548691 | Phase 3 and randomized trial | Centralized interactive voice-response system | Open-label | Multicenter | Age 18-85 years; At least a 3-month history of NDD-CKD or At least a 6-month history of HD-CKD; For NDD-CKD: Hb ≤11.5 g/dL, TSAT ≤30%, ferritin ≤300 ng/mL; For HD-CKD: Hb ≤12.5 g/dL,; TSAT ≤30%, ferritin ≤500 ng/mL; NDD-CKD patients: No IV iron use within 1 month prior to the study; HD-CKD patients: no anticipated need for >200 mg of IV iron during the 30-day study period | FCM | NDD-CKD = 15 mg/kg up to 1000 mg | Single dose |
|  |  |  |  |  |  |  |  |  | SCA | Determined by the investigator | determined by the investigator |
|  |  |  |  |  |  |  |  |  | FCM | HD-CKD 200 mg | Single dose |
|  |  |  |  |  |  |  |  |  | SCA | Determined by the investigator | Determined by the investigator |
| Derman | 2017 | A randomized trial of iron isomaltoside versus iron sucrose in patients with iron deficiency anemia | PROVIDE NCT02130063 | Randomized, non inferiority trial | NR | Open-label | Multicenter | Patients 18 years of age or older; moderate-to-severe iron deficiency anemia (IDA) ; documented intolerance or unresponsiveness to oral iron; Hemoglobin (Hb) <11.0 g/dL; Transferrin saturation (TSAT) <20%; Serum ferritin (s-ferritin) <100 ng/mL | FDI | 1000 mg to 2000 mg | Single or two administrations - one week apart |
|  |  |  |  |  |  |  |  |  | ISC | 200 mg maximum cumulative 2000 mg | Twice a week |
| Dhoot | 2020 | Effect of ferric-carboxy maltose on oxygen kinetics and functional status in heart failure patients with iron deficiency | - | Prospective, parallel, randomized controlled trial | Sequence of random numbers | Open-label | Unclear | Severe anemia (Hb<8 g/dl, requiring blood transfusion within 30 days) , chronic liver disease, vitamin B12 deficiency (<200 pg/dl) , serum folate deficiency (7 nmol/l) | FCM | Unclear | Unclear |
|  |  |  |  |  |  |  |  |  | SCA | Unclear | Unclear |
| Emrich | 2020 | Hypophosphatemia after high-dose iron repletion with ferric carboxymaltose and ferric derisomaltose-the randomized controlled HOMe aFers study | HOMe aFers study  NCT02905539 | Randomized and comparative study | Computer-based system | Double-blind | Single center | Adult women (18 years or older) with iron deficiencyanemia due to uterine bleeding in whom oral iron reple-tion was not tolerated or not efficient were invited toparticipate in our HOMe aFers trial. Anemia was defined following the WHO criteria ashemoglobin (Hb) < 12 g/dL | FDI | 20 mg/kg up maximum of 1000 mg | Once |
|  |  |  |  |  |  |  |  |  | FCM | 20 mg/kg up maximum of 1000 mg | Once |
| Evstatiev | 2011 | FERGIcor, a randomized controlled trial on ferric carboxymaltose for iron deficiency anemia in inflammatory bowel disease | FERGIcor Trial NCT00810030 | Randomized, controlled | Computer-generated list and stratified by gender and disease | Open-label | Multicenter | Iron deficiency anemia (Hb 7-12 g/dL [female] or 7-13 g/dL [male]) ; Ferritin <100 g/L; Mild to moderate IBD Crohn's disease (CD) (CD with CDAI <220 or UC with CAI ≤7) or IBD in remission (CDAI <150 or CAI ≤4) ; Normal levels of vitamin B-12 and folic acid; Age 18 years or older, nonpregnant | FCM | 500 mg or 1000 mg | Once week |
|  |  |  |  |  |  |  |  |  | ISC | 200 mg | 5 weeks |
| Ford | 2016 | Ferumoxytol versus placebo in iron deficiency anemia: efficacy, safety, and quality of life in patients with gastrointestinal disorders | NCT01114139 | Phase III, randomized, placebo-controlled, | Unclear | Double-blind | Multicenter | Males and nonpregnant; nonbreastfeeding females aged 18 years or older; Serum hemoglobin (Hgb) level between 70 g/L and less than 100 g/L; Transferrin saturation (TSAT) value less than 20%; Failure of oral iron therapy or intolerance to oral iron | FXM | 510 mg | Two doses |
|  |  |  |  |  |  |  |  |  | PCB | NR | Two doses |
| Gybel-Brask | 2018 | Intravenous iron isomaltoside improves hemoglobin concentration and iron stores in female iron-deficient blood donors: a randomized double-blind placebo-controlled clinical trial | NCT01895231 | Randomized, comparative trial | Permuted block randomization | Double-blind | Single center | Women at least 18 years of age, who were first-time donors and had a plasma ferritin (p-ferritin) concentration of less than 60 ng/mL were | FDI | NR | Single dose |
|  |  |  |  |  |  |  |  |  | PCB | - | Single dose |
| Hedenus | 2014 | Intravenous iron alone resolves anemia in patients with functional iron deficiency and lymphoid malignancies undergoing chemotherapy | NCT01101399 | Rrandomized, controlled, prospective trial | Predefined list using computer-generated randomization | Open-label | Multicenter | Adult patients with lymphoid malignancies (indolent non-Hodgkin’s lymphoma, multiple myeloma, or chronic lymphocytic leukemia) , anemia [hemoglobin (Hb) 8.5–10.5 g/dL], and FID [TSAT B 20 % and serum ferritin [30 ng/mL (women) or [40 ng/mL (men) ] who had received antineoplastic therapy for C8 weeks | FCM | 500 mg or 1000 mg based kg | Once a week |
|  |  |  |  |  |  |  |  |  | CTL | Unclear | Unclear |
| Hetzel | 2014 | A Phase III, randomized, open-label trial of ferumoxytol compared with iron sucrose for the treatment of iron deficiency anemia in patients with a history of unsatisfactory oral iron therapy | NCT01114204 | Active-controlled, phase III study | Unclear | Open-label | Multicenter | Male and female patients  18 years of age, Hgb >7 to <10 g dL21, transferrin saturation (TSAT) <20%, and history of unsatisfactory oral iron therapy or intolerance to oral iron | FXM | 510 mg | Day 1 and 5 |
|  |  |  |  |  |  |  |  |  | ISC | 200 mg | Five nonconsecutive days over a 14-day |
| Howaldt | 2022 | Long-Term Effectiveness of Oral Ferric Maltol vs Intravenous Ferric Carboxymaltose for the Treatment of Iron-Deficiency Anemia in Patients With Inflammatory Bowel Disease: A Randomized Controlled Noninferiority Trial | EudraCT 2015-002496-26 NCT02680756 | Phase 3b, randomized controlled trial | Centrally (unclear) | Open-label | Multicenter | Patients aged 18 years or older, with quiescent or mild to moderate IBD and they had IDA | OFM | 30 mg | Twice daily for ≥12 weeks |
|  |  |  |  |  |  |  |  |  | FCM | 1000 mg to 5500 mg | 12 week |
| Howard | 2022 | Supplementation with Iron in Pulmonary Arterial Hypertension | NCT01447628 | Randomized, comparative study | Stratified by sex with an appropriate fixed block size | Double-blind | Multicenter | Patients with idiopathic or heritable PAH and iron deficiency and who had been stable on their current therapy for the preceding 1 month were recruited. PAH was defined by a resting mean pulmonary artery pressure >25 mm Hg, pulmonary artery wedge pressure <15 mm Hg, and normal or reduced cardiac output on right heart catheterization. | FCM | 1,000 mg (or 15 mg/kg if weight,66.7 kg) | Single infusion - crossover after 12 weeks |
|  |  |  |  |  |  |  |  |  | PCB | NR | Single infusion - crossover after 12 weeks |
|  |  |  |  |  |  |  | Single center |  | IDX | 20 mg/kg | Single infusion - crossover after 12 weeks |
|  |  |  |  |  |  |  |  |  | PCB | NR | Single infusion - crossover after 12 weeks |
| Ikuta | 2019 | Comparison of efficacy and safety between intravenous ferric carboxymaltose and saccharated ferric oxide in Japanese patients with iron-deficiency anemia due to hypermenorrhea: a multi-center, randomized, open-label noninferiority study | NCT02731534 | Randomized, noninferiority  study | Central registration system for allocation | Open-label | Multicenter | Mean Hb levels 6.0-11.0 g/dL; with less than 1.0 g/dL difference between two measurements; Serum ferritin levels < 12 ng/mL in Screening; Awareness of hypermenorrhea in each menstrual cycle for 6 months before screening; Outpatients aged 18-50 years | FCM | 500 mg | Intervals of at least1 week |
|  |  |  |  |  |  |  |  |  | SFO | 80 mg or 120 mg | 2 or 3 times per week |
| Jin | 2024 | A randomized, controlled, open label non-inferiority trial of intravenous ferric carboxymaltose versus iron sucrose in patients with iron deficiency anemia in China | NCT03591406 | Randomized, controlled trial | Interactive response technology system. | Open-label | Multicenter | 18 years or older, Hb levels < 11 g/dL for females or < 12 g/dL for males, microcytic hypochromic anemia (mean corpuscular Hb concentration < 320 g/L, mean corpuscular volume (MCV) < 80 fL, mean corpuscular Hb (MCH) < 27 pg) , transferrin saturation (TSAT) < 16%, and serum ferritin < 100 μg/L in the presence of underlying inflammatory conditions, as indicated by highsensitivity C-reactive protein (hsCRP) above the normal range (1–3 mg/L) , or serum ferritin < 15 μg/L in the absence of inflammatory conditions (hsCRP within the normal range) | FCM | 500-1000 mg | Unclar |
|  |  |  |  |  |  |  |  |  | ISC | Calculated by onbody weight and Hb | with a maximum of three doses per week and up to a total of 11 injections |
| Kalra | 2016 | A randomized trial of iron isomaltoside 1000 versus oral iron in non-dialysis-dependent chronic kidney disease patients with anaemia | NCT01102413 | Randomized, non-inferiority trial | Stratified block - web  response system | Open-label | Multicenter | ≥18 years of age with estimated glomerular filtration rate (eGFR) between 15 and 59 mL/min/1.73 m2, Hb <11.0 g/dL, either or both serum ferritin <200 μg/L and TSAT <20% and had not received ESA treatment within 8 weeks prior to screening | FDI | 500 mg | Once weekly (2 doses) |
|  |  |  |  |  |  |  |  |  | OFS | 100 mg | Daily for 8 weeks |
| Kalra | 2022 | Intravenous ferric derisomaltose in patients with heart failure and iron deficiency in the UK (IRONMAN) : an investigator-initiated, prospective, randomised, openlabel, blinded-endpoint trial | IRONMAN NCT02642562 | Investigator-initiated, randomized, blinded-endpoint, event-driven trial | Web-based system | Open-label | Multicenter | 18 years or older, with new or established symptomatic heart failure, evidence of iron deficiency (serum ferritin <100 μg/L or transferrin saturation <20%) , and a left ventricular ejection fraction of 45% or less within the preceding 24 months | FDI | According to weight and haemoglobin | Unclear |
|  |  |  |  |  |  |  |  |  | SCA | Unclear | Unclear |
|  |  |  |  |  |  |  |  |  | FDI | According to weight and haemoglobin | Unclear |
|  |  |  |  |  |  |  |  |  | SCA | Unclear | Unclear |
| Kulnigg | 2008 | A Novel Intravenous Iron Formulation for Treatment of Anemia in Inflammatory Bowel Disease: The Ferric Carboxymaltose (FERINJECT ) Randomized Controlled Trial | FERINJECT | Randomized, controlled phase III study | Central randomization system | Open-label | Multicenter | Patients with IDA (defined by Hb ≤10 g/dL and transferrin saturation [TfS] <20%, or serum ferritin <100 μg/L) | FCM | According to weight | Weekly - maximum 3 doses |
|  |  |  |  |  |  |  |  |  | OFS | According to weight | Weekly - maximum 3 doses |
| Li | 2008 | Intravenous Iron Sucrose in Chinese Hemodialysis Patients with Renal Anemia | - | Randomized, controlled, parallel-group trial. | Computer-generated random number list | NR | Single center | Patients were included in the study if they were on maintenance hemodialysis, hemodialysis frequency 2–3 times/week, their condition had been stable for at least 1 month, they had serum ferritin (SF) ! 500 ng/ml, transferrin saturation (TSAT) ! 30%, Hb concentration 60–90 g/l, and Hct of 0.18–0.27. | ISC | 100 mg | Twice a week in the first 8 weeks, then once a week after this. |
|  |  |  |  |  |  |  |  |  | FSC | 200 mg | 12 weeks |
| Lindgreen | 2009 | Intravenous iron sucrose is superior to oral iron sulphate for correcting anaemia and restoring iron stores in IBD patients: A randomized, controlled, evaluator-blind, multicentre study | - | Randomized, controlled, evaluator-blind study | Over the Internet, by applying the minimization method | Single-blind (observer blind) | Multicenter | Males and females aged 1885 years suffering from UC or CD. The patients had B-Hb levels B115 g/L, verified at least twice (within 3 months) and S-ferritin concentrations B300 mg/L and iron deficiency defined by S-iron, transferrin and transferrin saturation (TSAT) . | ISC | 200 mg | Once a week or every second |
|  |  |  |  |  |  |  |  |  | OFS | 100 mg | Once a week |
| Ferrer-Barcelo | 2019 | Randomised clinical trial: intravenous vs oral iron for the treatment of anaemia after acute gastrointestinal bleeding | - | Randomized unblinded study | Alternating sequence in order of enrolment controlled by the principle investigator | Open-label | Single center | Non‐variceal (aged >18 years) and subsequent diagnosis of anaemia secondary to acute GIB (Hb <10 g/dL on the day of hospital discharge, Day 0) | FCM | 1500 or 2000 mg | Day 0 and Day 7 |
|  |  |  |  |  |  |  |  |  | OFS | 650 mg | 6 weeks |
| Macdougall | 2014b | A randomized comparison of ferumoxytol and iron sucrose for treating iron deficiency anemia in patients with CKD | NCT01052779 | Randomized,  international, phase II trial | Unclear | Open-label | Multicenter | Hemoglobin < 11.0 g/dl; Transferrin saturation < 30%; eGFR < 60 ml/min per 1.73 m^2 or a diagnosis of CKD (e.g., nephropathy or nephritis) ; Age ≥ 18 years; Hemoglobin ≥ 7 g/dl | FXM | 510 mg | 2 dose until 1.02 g |
|  |  |  |  |  |  |  |  |  | ISC | 100 or 200 mg | Between 3 or 5 dose - Max 1.0g |
| Macdougall | 2019 | Ferumoxytol for iron deficiency anemia in patients undergoing hemodialysis. The FACT randomized controlled trial | FACT NCT01227616 | Phase 4, randomized study | Unclear | Open-label | Multicenter | Adults (aged ≥ 18 years) with CKD undergoing hemodialysis for ≥ 3 months before screening with IDA (defined as hemoglobin levels at screening of < 11.5 g/dL and transferrin saturation (TSAT) < 30%) were included. | FXM | 1.02 g | Day 1 and Day 5 -8 |
|  |  |  |  |  |  |  |  |  | ISC | 1g | Daily for 10 days |
| Macdougall | 2014a | FIND-CKD: a randomized trial of intravenous ferric carboxymaltose versus oral iron in patients with chronic kidney disease and iron deficiency anaemia | FIND-CKD NCT00994318 | Prospective, randomized study | Block via central interactive | Open-label | Multicenter | Adult (≥18 years) patients with nondialysis-dependent CKD were eligible if (i) at least one Hb level was between 9 and 11 g/dL within 4 weeks of randomization; (ii) any ferritin level was <100 or <200 μg/L with transferrin saturation (TSAT) <20%, within 4 weeks of randomization; (iii) estimated glomerular filtration rate (eGFR) was ≤60 mL/ min/1.73 m2 [Modification of Diet in Renal Disease-4 (MDRD-4) equation [22]], the rate of eGFR loss was ≤12 mL/min/1.73 m²/year and predicted eGFR at 12 months was ≥15 mL/min/1.73 m2; and (iv) no ESA had been administered within 4 months of randomization. | FCM | Adjusterd by ferritin level | Day 0, 7 and every 4 week |
|  |  |  |  |  |  |  |  |  | OFS | 100 mg | Twice dailt to Week 52 |
| Mahey | 2016 | Randomized controlled trial comparing ferric carboxymaltose and iron sucrose for treatment of iron deficiency anemia due to abnormal uterine bleeding | CTRI/2015/09/006224 | Prospective randomized controlled trial | Computerized table | Open-label | Single center | Eligible patients were older than 18 years of age, had a84 serum hemoglobin concentration of 60.0–109.9 g/L, were experiencing85 heavy uterine bleeding with a pictorial bleeding assessment chart86 (PBAC) score higher than 100, and did not intend to conceive during87 the 3-month study period. | FCM | Up to 1000 mg | Once per week |
|  |  |  |  |  |  |  |  |  | ISC | 300 mg | Twice a week |
| Noronha | 2017 | Phase III randomized trial comparing intravenous to oral iron in patients with cancer-related iron deficiency anemia not on erythropoiesis stimulating agents | CTRI/2016/01/006520 | Randomized controlled phase III trial. | Computer generated schedule with block | Open-label | Single center | Over 18 years old with malignancy requiring chemotherapy, who had hemoglobin (Hb) level <12 g/dL with at least one feature indicating iron deficiency: serum ferritin <100 mcg/mL, transferrin saturation <20% or hypochromic red blood cells >10%. Vitamin B12 and folate levels had to be adequate. | ISC | Calculated by onbody weight and Hb | Two doses,before cycle 1 and 2 |
|  |  |  |  |  |  |  |  |  | OFS | 100 mg | Three times a day |
| Onken | 2013 | A multicenter, randomized, active-controlled study to investigate the efficacy and safety of intravenous ferric carboxymaltose in patients with iron deficiency anemia | NCT00982007 | Randomized, active-controlled trial | Interactive voice response system | Open-label | Multicenter | Age at least 18 years; Screening hemoglobin (Hb) not more than 11 g/dL; Ferritin level not more than 100 ng/mL or not more than 300 ng/mL when TSAT not more than 30%; Met all other eligibility criteria (refer to Appendix Table S1) ; Tolerated a 14-day run-in of oral ferrous sulfate without severe side effects; Unsatisfactory response to oral iron (Hb increase <1 g/dL from baseline with ≥67% compliance) ; Ferritin and TSAT values within inclusion criteria ranges after run-in | FCM | 15 mg/kg (maximum dose of 750 mg) | Days 0 and 7 |
|  |  |  |  |  |  |  |  |  | OFS | 325 mg | Three times a day |
|  |  |  |  |  |  |  |  |  | FCM | 15 mg/kg (maximum dose of 750 mg) | Days 0 and 7 |
|  |  |  |  |  |  |  |  |  | IVSC | Variable | Variable |
| Onken | 2014 | Ferric carboxymaltose in patients with iron-deficiency anemia and impaired renal function: the REPAIR-IDA trial | REPAIR-IDA NCT00981045 | Randomized,  active-controlled, noninferiority trial | Interactive voice response system | Single-blind | Multicenter | Age ≥18 years; Hemoglobin ≤11.5 g/dL; Chronic renal impairment with GFR <60 mL/min/1.73 m^2 on two consecutive measurements, or GFR <90 mL/min/1.73 m^2 with additional kidney damage or elevated cardiovascular risk; Two hemoglobin concentrations within 0.7 mg/dL of each other, measured within 7 days, averaging ≤11.5 g/dL; Ferritin ≤100 ng/mL, or ferritin ≤300 ng/mL with TSAT ≤30%; Stable ESA dose (±20%) for 4 weeks prior to randomization; Iron-deficiency anemia diagnosis | FCM | 15 mg/kg (maximum dose of 750 mg) | Days 0 and 7 |
|  |  |  |  |  |  |  |  |  | ISC | 200 mg | Days 0, 7 and 14, with two additional doses |
| Patel | 2024 | Randomised Controlled Trial Evaluating the Impact of Intravenous Iron (ferric carboxymaltose) Supplementation Among Epithelial Ovarian Cancer Patients with Anemia | “Registry- India” (REF/2019/05/025907) | Parallel-group randomized controlled trial | Web based service | Single-blind (observer blind) | Single center | Age 18–75 years; diagnosis of epithelial ovarian carcinoma; patients on the ≤ 3rd cycle of adjuvant chemotherapy after primary cytoreductive surgery or who underwent interval cytoreductive surgery after receiving ≤ 3 neoadjuvant chemotherapy; normal liver and kidney function; no prior radiotherapy; Eastern Cooperative Oncology Group performance status of ≤ 2; baseline hemoglobin levels of ≤ 10 g/dl during cancer treatment or decrease of > 2 g/dl in hemoglobin level during cancer treatment. | FCM | 1000 mg | Single tablet |
|  |  |  |  |  |  |  |  |  | FAS | 100 mg | Twice a day for 90 days |
| Pieracci | 2014 | A multicenter, randomized clinical trial of IV iron supplementation for anemia of traumatic critical illness* | NCT01180894 | Randomized, placebo controlled | Computer-generated block | Single-blind | Multicenter | Anemia (hemoglobin < 12 g/dL) ; Age ≥ 18 years; ≤ 72 hours from ICU admission; Expected ICU LOS ≥ 5 days | ISC | 100 mg | Thrice weekly |
|  |  |  |  |  |  |  |  |  | PCB | 100 mg | Thrice weekly |
| Provenzano | 2009 | Ferumoxytol as an Intravenous Iron Replacement Therapy in Hemodialysis Patients | NCT00233597 | Randomized, controlled, phase III trial | Telephone based system | Open-label | Multicenter | >18 yr of age, on HD for at least 90 d, hemoglobin 11.5 g/dl, transferrin saturation (TSAT) 30%, serum ferritin 600 ng/ml, and stable (25%) dose ESA therapy for at least 10 d before dosing. | FXM | 2x510 mg | Two doses |
|  |  |  |  |  |  |  |  |  | FFM | 200 mg | 200 mg/daily for 21 d. |
| Qunibi | 2010 | A randomized controlled trial comparing intravenous ferric carboxymaltose with oral iron for treatment of iron deficiency anaemia of non-dialysis-dependent chronic kidney disease patients | - | Phase 3, randomized trial | Stratified by severity | Open-label | Multicenter | Subjects ≥12 years of age with estimated glomerular filtration rate (GFR) ≤45 mL/min/1.73 m2, Hb level ≤11 g/dL, TSAT ≤25% and ferritin ≤300 ng/mL were enrolled. | FCM | 15 mg/kg (maximum dose of 1000 mg) | Day 17 and Day 31 |
|  |  |  |  |  |  |  |  |  | OFS | 325 mg | Meals three times daily |
| Reinisch | 2013 | A Randomized, Open-Label, Non-Inferiority Study of Intravenous Iron Isomaltoside 1,000 (Monofer) Compared With Oral Iron for Treatment of Anemia in IBD (PROCEED) | - | Randomized, comparative, noninferiority study | Permuted block randomization | Open-label | Multicenter | ≥ 18 years of age with a diagnosis of IBD and a score of ≤ 5 on the Harvey – Bradshaw index for Crohn ’ s disease (25) or a partial Mayo score of ≤ 6 for ulcerative colitis (26) , a Hb < 12 g / dl (7.45 mmol / l) , and a transferrin saturation (TSAT) < 20 % | FDI | 1000 mg | Single once weekly |
|  |  |  |  |  |  |  |  |  | OFS | 200 mg | Daily for 8 weeks |
| Roger | 2017 | Safety of intravenous ferric carboxymaltose versus oral iron +C90in patients with nondialysis-dependent CKD: an analysis of the 1-year FIND-CKD trial | FIND-CKD NCT00994318 | Randomized, three-arm study | NR | Open-label | Multicenter | Adult patients with nondialysis-dependent CKD were eligible if (i) at least one Hb level was 9–11 g/dL; (ii) any ferritin level was <100 mg/L, or <200 mg/L with TSAT <20%; (iii) estimated glomerular filtration rate (eGFR) was 60 mL/min/1.73 m2 | FCM | 400-600 | Unclear |
|  |  |  |  |  |  |  |  |  | FCM | 100-200 | Unclear |
|  |  |  |  |  |  |  |  |  | OFS | 304 mg | (100mg of iron) twice daily to Week 52 |
| Saroj Vadhan | 2014 | Efficacy and safety of IV ferumoxytol for adults with iron deficiency anemia previously unresponsive to or unable to tolerate oral iron | NCT01114139 | Randomized, placebo-controlled study | Interactive Voice system | Double-blind | Multicenter | Women 18 years of age with a history of IDA, defined as a hemoglobin (Hgb) level <10.0 g/dL and a transferrin saturation (TSAT) <20%, and a history of unsatisfactory oral iron therapy or in whom oral iron could not be used | FXM | 510 mg | Day 1, with a second dose 2–8 days later |
|  |  |  |  |  |  |  |  |  | PCB | NR | NR |
| Schroder | 2005 | Intravenous Iron Sucrose versus Oral Iron Supplementation for the Treatment of Iron Deficiency Anemia in Patients with Inflammatory Bowel Disease—A Randomized, Controlled, Open-Label, Multicenter Study | - | Randomized, controlled, clinical trial | Computer-generated random number table | Open-label | Multicenter | Patients with an IDA as defined by a hemoglobin (Hb) concentration of ≤1.05 g/L (females)  or Hb ≤1.10 g/L (males) plus a transferrin saturation (TSAT) ≤20% and/or serum ferritin concentrations ≤20 μg/L | ISC | 7 mg/kg | One dose |
|  |  |  |  |  |  |  |  |  | OFS | 100-200 mg | 100–200 mg per day for 6 wks |
| Tabish | 2024 | Randomized Controlled Trial of Intravenous Ferric Carboxymaltose vs Oral Iron to Treat Iron Deficiency Anemia After Variceal Bleed in Patients With Cirrhosis | CTRI/2022/11047650 | Randomized controlled trial with a superiority design | Computer-generated random number table | Open-label | Single center | Patients 18 years and older, who had cirrhosis due to any etiology, and who had presented with variceal bleed with hemoglobin less than 10 g/dL and iron deficiency (ferritin,100 ng/L) | FCM | 1500 to 2000 mg | Day 0 and day 7 |
|  |  |  |  |  |  |  |  |  | CAI | 100 mg | 12 weeks |
| Van Wyck | 2005 | A randomized, controlled trial comparing IV iron sucrose to oral iron in anemic patients with nondialysis-dependent CKD | - | Phase III, randomized, trial | NR | Open-label | Multicenter | Anemic patients with stage 3 to 5 ND-CKD who required iron supplementation, | ISC | 1000 mg | Day 0 and day 14 |
|  |  |  |  |  |  |  |  |  | OFS | 500 mg | Daily, for 56 days |
| Waziri | 2016 | Comparison of intravenous low molecular weight iron dextran and intravenous iron sucrose for the correction of anaemia in pre-dialysis chronic kidney disease patients: a randomized single-centre study in Nigeria | NI | Randomized trial and prospective | Permuted block | Open-label | Single center | Pre-dialysis stages 3-5 CKD patients; Aged ≥18 years; ESA and intravenous iron naïve; Hb ≤11.0 g/dL; Ferritin <200 ng/mL; TSAT <25% | IDX | 250 mg | Four divided doses |
|  |  |  |  |  |  |  |  |  | ISC | 200 mg | Five divided doses over 10 days |
| Wolf | 2018 | Randomized trial of intravenous iron-induced hypophosphatemia | FIRM trial NCT02694978 | Phase III, Randomized, Multicenter | Centralized Interactive Voice or web Response System | Double-blind | Multicenter | ≥18 years; Hemoglobin <12.0 g/dl for women or <14.0 g/dl for men; Transferrin saturation (TSAT) ≤20%; Ferritin ≤100 ng/ml; Previous intolerance or insufficient response to oral iron, not pregnant | FXM | 510 mg | Day 1 and Day 8 |
|  |  |  |  |  |  |  |  |  | FCM | 750 mg | Day 1 and Day 8 |
| Wolf | 2020 | Effects of Iron Isomaltoside vs Ferric Carboxymaltose on Hypophosphatemia in Iron-Deficiency Anemia Two Randomized Clinical Trials | NCT03238911 and NCT03237065 | Randomized clinical trial | Interactive web response system | Double-blind | Multicenter | 18 years and older; Iron-deficiency anemia (hemoglobin ≤11 g/dL) ; Serum ferritin ≤100 ng/mL; Intolerance or unresponsiveness to 1 month or more of oral iron; Body weight 50 kg or more; Estimated glomerular filtration rate ≥65 mL/min/1.73 m^; Serum phosphate level ≥2.5 mg/dL; No acute bleeding >500 mL within 72 hours before study inclusion; No hemochromatosis or other iron-storage disorder; No intravenous iron use within 30 days prior to screening | FDI | 1000 mg | Single dose |
|  |  |  |  |  |  |  |  |  | FCM | 750 mg | Day 0 and 7 |
| Zahr | 2024 | Oral Liposomal Iron Versus Injectable Iron Sucrose for Anemia Treatment in Non-dialysis Chronic Kidney Disease Patients: A Noninferiority Study | NCT06556134 | Randomized controlled trial | Sequentially numbered, sealed envelopes. | NR | Single center | Participants were required to be over 18 years of age, with an estimated glomerular filtration rate (eGFR) of ≤60 mL/min/1.73 m² (using the Modification of Diet in Renal Disease equation, MDRD) , Hb levels ≤12 g/dL, ferritin levels ≤100 ng/mL, transferrin saturation (TSAT) ≤25%, and parathyroid hormone (PTH) serum levels between 30 and 300 pg/mL | ISC | 100 mg | Weekly for three months |
|  |  |  |  |  |  |  |  |  | OLS | 30 mg | Three months |
| Zoller | 2023 | Hypophosphataemia following ferric derisomaltose and ferric carboxymaltose in patients with iron deficiency anaemia due to inflammatory bowel disease (PHOSPHARE-IBD) : a randomised clinical trial | PHOSPHARE-IBD 2017-002452-87 | Randomised, clinical trial | Interactive web  response system | Double-blind | Multicenter | Adults aged ≥18 years; Diagnosed with IBD and IDA; Haemoglobin (Hb) <13 g/dl; Serum ferritin ≤100 ng/mL; History of intolerance or unresponsiveness to oral iron, or clinical need to administer iron rapidly; Body weight ≥50 kg; Estimated glomerular filtration rate ≥65 mL/min/1.73 m^; Serum phosphate >2.5 mg/dL. | FDI | 20 mg/kg (maximum dose of 1000 mg) | Single or double infusion |
|  |  |  |  |  |  |  |  |  | FCM | 20 mg/kg (maximum dose of 1000 mg) | Single or double infusion |

**Legend:** NR: - No reported, CAI - Carbonil iron tablet, CTL – Control, FAS - Ferrous ascorbate, FCM - Ferric carboxymaltose, FDI - Ferric derisomaltose, FFM - Ferrous fumarate, FSC - Ferrous succinate, FXM – Ferumoxytol, IDX - Iron dextran, ISC - Iron sucrose, ISFG - Iron sucrose or sodium ferric gluconate, IVSC - Intravenous Standard-of-care, OFM - Oral ferric maltol, OFS - Oral Ferrous Sulfate, OLS - Oral lipossomal iron, PCB – Placebo, SCA - Standard of care, SFO - Saccharated ferric oxide, SGC – Sodium ferric gluconate complex, SUI – Sucrosomial Iron.

# Supplement 7 – Patients characteristics.

| **Study** | **Treatment** | **n** | **% male** | **Age**  **∑** ± **SD** | **Weight**  **∑** ±**SD** | **Hb**  **∑** ±**SD** | **TSAT**  **∑** ±**SD** | **FER**  **∑** ± **SD** | **Sponsor** | **Patients** |
| --- | --- | --- | --- | --- | --- | --- | --- | --- | --- | --- |
| Adkinson, 2018 | FXM | 997 | 25.5 | 55.6±17.3 | 84.3±24.9 | 10.4±1.5 | 13.9±28.4 | 54.3±115.4 | AMAG Pharmaceuticals, Inc | Anemia caused by different etiologies |
|  | FCM | 1000 | 22.4 | 54.8±17.0 | 85.8 (26.0) | 10.4 (1.5) | 13.9 (25.0) | 55.1±120.2 |  |  |
| Agarwal, 2006 | SGC | 44 | 44.4 | 65.5±12.9 | 86.6±21.5 | 10.5±0.9 | 17.2±7.9 | 72.5±72.9 | Watson Laboratories Inc. | Chronic kidney disease |
|  | OFS | 45 | 38.5 | 62.3±15.2 | 90.0±30.7 | 10.7±0.9 | 17.9±6.5 | 66.4±52.2 |  |  |
| Agarwal, 2015 | ISC | 67 | 74.6 | 63.2±10.7 | NR | 10.7±1 | 17.4±5.1 | 173±138 | Amgen | Chronic kidney disease |
|  | OFS | 69 | 78.3 | 67.8±11.5 | NR | 10.5±1 | 17.3±6.7 | 133±155 |  |  |
| Ambrosy, 2021 | FDI | 158 | 45 | 69 (IQR 62−80) | 93 (IQR 73−111) | 9.6 (IQR 8.7−10.1) | 14 (IQR 11−19) | 70 (IQR 29−124) | Pharmacosmos A/S | Chronic Kidney Disease With and Without Heart Failure |
|  | ISC | 86 | 49 | 70 (IQR 60−77) | 86 (IQR 74−101) | 9.5 IQR (8.7−10.1) | 14 IQR (10−19) | 58 (IQR 28−122) |  |  |
|  | FDI | 861 | 37 | 69 (IQR 61−76) | 82 (IQR 69−97) | 9.8 IQR (9.0−10.5) | 16 IQR (11−21) | 52 (IQR 23−111) |  |  |
|  | ISC | 120 | 33 | 71 (IQR 64−78) | 78 (IQR 68−92) | 9.8 IQR (9.1−10.5) | 17 IQR (12−21) | 60 (IQR 25−124) |  |  |
| Anirban, 2008 | IDX | 113 | 63.7 | 40.26±13.21 | NR | 7.7±1.21 | 17.5±5.96 | 140.1±78.26 | Not described | Chronic Kidney Disease |
|  | SGC | 110 | 71.8 | 44.87±13.8 | NR | 7.8±1.43 | 17.5±4.95 | 126.8±79.93 |  |  |
|  | ISC | 116 | 72.4 | 44.93±13.54 | NR | 7.7±1.37 | 17±5.49 | 126.2±88.38 |  |  |
| Anker, 2009 | FCM | 304 | 47.7 | 67.8±10.3 | 77.0±14.2 | 11.9 ±1.3 | 17.7±12.6 | 52.5±54.5 | Vifor Pharma | Heart Failure |
|  | PCB | 155 | 45.2 | 67.4±11.1 | 77.6±16.3 | 11.9 ±1.4 | 16.7±8.4 | 60.1±66.5 |  |  |
| Auerbach, 2019 | FDI | 1009 | 11.6 | 44.1±14.8 | NR | 9.25±1.28 | 7.43±10.93 | 14.4±42.6 | Pharmacosmos A/S | IDA caused by different etiologies |
|  | ISC | 503 | 9.3 | 43.8±14.4 | NR | 9.17±1.27 | 6.69±7.44 | 11.9±37.6 |  |  |
| Bae, 2023 | FCM | 15 | 13.3 | 42.10±7.11 | NR | 10.16±1.55 | 4.67 ± 3.64 | 3.73±2.56 | Partly supported by NIH NIBIB | Restless legs |
|  | PCB | 14 | 0.7 | 46.25±4.53 | NR | 10.08±1.01 | 6.75±3.62 | 3.05±0.91 |  |  |
| Bailie, 2010 | FCM | 594 | 11.8 | 41.9±16.7 | 80.6±20.7 | 10.±1.2 | 9.46±5.9 | 32.9±50.2 | NR | IDA caused by different etiologies |
|  | PCB | 559 | NR | NR | NR | NR | NR | NR |  |  |
| Beck-da-Silva, 2014 | ISC | 10 | 66.7 | 66.9±8.3 | NR | 11.2±0.6 | 18.9± 9.7 | 185± 146 | NR | Heart failure patients |
|  | OFS | 7 | 75 | 63.5±16.2 | NR | 11.3±0.5 | 18.8± 8.6 | 101± 135 |  |  |
|  | PCB | 6 | 66.7 | 68.9±10.1 | NR | 10.9±0.7 | 13.5± 5.8 | 95± 128 |  |  |
| Bertani, 2021 | FCM | 20 | 60 | 43.5 (IQR 31.5–66.8) | NR | 10.3 (IQR 9.0–11.0) | NR | 10  (IQR 5–13) | Pharmanutra S.p.A (Pisa, Italy) | Ulcerative colitis |
|  | SUI | 20 | 55 | 42.0 (IQR 29.5–60.8) | NR | 11.1 (IQR 9.9–11.6) | NR | 16 (IQR 9–25) |  |  |
| Bhandari, 2015 | FDI | 234 | 67.1 | 60.1±16.21 | NR | 11.2±0.66 | 21.6±5.95 | 367±180 | Pharmacosmos A/S. | Chronic kidney disease and on haemodialysis patients |
|  | ISC | 117 | 63.2 | 59.5±15.39 | NR | 11±0.76 | 22.6±6.76 | 384±184 |  |  |
| Birgegard, 2016 | FDI | 231 | 34.6 | 55±12 | NR | NR | NR | NR | NR | Nonmyeloid Malignancies |
|  | OFS | 119 | 24.4 | 54±11 | NR | NR | NR | NR |  |  |
| Boomershine, 2018 | FCM | 41 | 97.6 | 41.2±11.1 | NR | 12.3±1.2 | 14.6±5.3 | 19.0±12.4 | Luitpold Pharmaceuticals, Inc | Fibromyalgia |
|  | PCB | 40 | 2.4 | 43.9±10.8 | NR | 12.3±1.2 | 13.6±5.5 | 18.1±11.6 |  |  |
| Charytan, 2013 | FCM | 50 | 56 | 54.8±14.4 | 93.1 ± 24.5 | 11.2 ± 0.7 | 22.6±4.9 | 273.2±112.3 | Luitpold Pharmaceuticals, Inc | Chronic kidney disease: |
|  | SCA | 47 | 78.7 | 57.1±12.6 | 88.4 ± 21.1 | 11.3±0.7 | 24.6±4.8 | 247.9±135.6 |  |  |
|  | FCM | 204 | 32.8 | 64.4±11.7 | 91.8 ± 25.8 | 10.4±0.8 | 19.1±5.8 | 89.3±71.8 |  |  |
|  | SCA | 212 | 32.5 | 64.5±11.6 | 89.6 ± 24.5 | 10.2±0.9 | 18.5±6.3 | 91.8±87.0 |  |  |
| Derman, 2017 | FDI | 330 | 10 | 49±16 | 86±23 | 9.4±1.2 | 5.8±5.0 | 14.3±32.8 | Not described | IDA caused by different etiologies |
|  | ISC | 161 | 9.3 | 47±15 | 82±21 | 9.4±1.3 | 6.4±5.9 | 15.6±47.2 |  |  |
| Dhoot, 2020 | FCM | 35 | 57.1 | 51±11.6 | NR | 11±1.4 | NR | 40.1 ±27.2 | Not described | Heart failure patients |
|  | SCA | 35 | 60 | 54.8 ±9 | NR | 11.3 ±0.9 | NR | 45.5 ±35.1 |  |  |
| Emrich, 2020 | FDI | 13 | 0 | 40 ±10 | NR | 10.1±1.4 | 344±37 | 6 (IQR 4-8) | Pharmacosmos A/S | Anemia due to uterine bleeding |
|  | FCM | 13 | 0 | 34 ±11 | NR | 10.7±1.2 | 325±34 | 8 (IQR 6-12) |  |  |
| Evstatiev, 2011 | FCM | 244 | 40.2 | 39.5 (IQR 18.0–81.0) | NR | 10.1±1.5 | 9.0±9.1 | 14.8±24.6 | Vifor Pharma | Inflammatory Bowel Disease |
|  | ISC | 239 | 42.3 | 38 (IQR 18.0–78.0) | NR | 10.3±1.5 | 9.6±9.5 | 17.8±27.6 |  |  |
| Ferrer-Barcelo,2019 | FCM | 29 | 58.6 | 57.8±15.3 | 72.5±10.5 | 9.3±0.5 | 16±12.5 | 85.4±82.2 | Walter Fürst (SFL Regulatory Affairs & Scientific Communications, Switzerland) and funded by Vifor Pharma España. | Anaemia after acute gastrointestinal bleeding |
|  | OFS | 32 | 68.7 | 62.5±18.3 | 76.9±16.4 | 9.2±0.7 | 14.9 ± 8.9 | 78.5±62.2 |  |  |
| Ford, 2016 | FXM | 173 | 21.4 | 47.4±16.85 | NR | 8.9±8.9 | 6.5±12.97 | NR | AMAG Pharmaceuticals, Inc. | Gastrointestinal disorders |
|  | PCB | 58 | 24.1 | 52.1±15.93 | NR | 8.7±7.3 | 4.7±3.53 | NR |  |  |
| Gybel-Brask, 2018 | FDI | 43 | 0 | 23.2±3.75 | 63.2± 8.4 | 12.3±0.6 | 15.2±8.3 | 16.4±6.5 | Pharmacosmos A/S (Holbaek, Denmark) . | iron-deficient blood donors |
|  | PCB | 42 | 0 | 24.9±6.01 | 64.2±9.2 | 12.4±0.8 | 14.1±7.2 | 14.0±6.1 |  |  |
| Hedenus, 2014 | FCM | 8 | 62.5 | 69.5 (IQR 41–79) | 67.8 (IQR 59.0–103.7) | 9.5 (IQR 9.0–10.5) | 16 (IQR 3–35) | 216 (IQR 65–800) | Vifor (International) AG | Lymphoid malignancies undergoing chemotherapy |
|  | CTL | 11 | 63.6 | 71.0 (IQR 26–88) | 66.4 (IQR 49.0–78.0) | 9.8 (IQR 8.4–10.) | 18 (IQR 0–31) | 322 (IQR 8–707) |  |  |
| Hetzel, 2014 | FXM | 406 | 15.8 | 48.0±14.89 | 68.5±14.28 | 8.9 | 6.1±10.0 | 26.8±84.4 | AMAG Pharmaceuticals | Anemia with a history of unsatisfactory oral iron therapy |
|  | ISC | 199 | 19.6 | 48.9±14.66 | 70.9±15.74 | 8.8 | 5.5±10.4 | 20.1±63.6 |  |  |
| Howaldt, 2022 | OFM | 125 | 46 | 40.0±14.6 | NR | 10.0±1.1 | NR | 16.6±71.6 | NR | Inflammatory Bowel Disease |
|  | FCM | 125 | 38 | 40.4±15.5 | NR | 10.1±1.0 | NR | 9.3±12.2 |  |  |
| Howard, 2022 | FCM | 39 | 25.6 | 49 ± 14.5 | NR | 1.4±4. | NR | 17.0 ± 21.8 | British Heart Foundation (RG/10/16/28575) . | Pulmonary Arterial Hypertension |
|  | PCB | 38 | 25.6 | 49±14.5 | NR | 13.7±2.7 | NR | 17.0±21.8 |  |  |
|  | IDX | 17 | 11.8 | 30 ± 11.0 | NR | NR | NR | 11.0±7.0 |  |  |
|  | PCB | 17 | 11.8 | 30±11.0 | NR | 13.0±2.9 | NR | 11.0±7.0 |  |  |
| Ikuta, 2019 | FCM | 119 | 0 | 41.3±6.2 | 55.7±9.22 | 9.1±1.15 | 4.7 (95% CI 4.35. 5.10) | 4.3 (95% CI 4.09. 4.53) | Zeria Pharmaceutical Co., Ltd. | Anemia due to hypermenorrhea |
|  | SFO | 119 | 0 | 41.4±6.1 | 55.2±8.49 | 9.2±1.08 | 4.6 (95% CI 4.22. 4.92) | 4.4 (95% CI 4.12. 4.71) |  |  |
| Jin, 2024 | FCM | 187 | 7.5 | 39.9±9.9 | 59.8±9.4 | 7.74±1.49 | 4.82±1.90 | 4.47±2.15 | Vifor Pharma, Glattbrugg, Switzerland | iron deficiency anemia |
|  | ISC | 180 | 6.1 | 38.9±8.7 | 60.4±9.4 | 8.06±1.45 | 4.92±2.91 | 4.93±6.10 |  |  |
| Kalra, 2016 | FDI | 233 | 39.5 | 57.63±15.54 | NR | 9.73±1.09 | 19.20±36.79 | 80.18±114.33 | NR | Non-dialysis dependent chronic kidney disease |
|  | OFS | 118 | 54.2 | 57.94±16.34 | NR | 9.60±1.17 | 16.97±11.67 | 110.35±109.58 |  |  |
| Kalra, 2022 | FDI | 569 | 75 | 73·2 (IQR 66·7–80·1) | NR | 12·1 (IQR 11·2–12·8) | 15% (IQR 11–20) | 49·0 (IQR 30·0–86·0) | British Heart Foundation Pharmacosmos | Heart failure |
|  | SCA | 568 | 72 | 73·5 (IQR 67·1–79·1) | NR | 12·1 (IQR 11·2–12·9) | 15% (IQR 10–19) | 50·0 (IQR 30·0–85·0) |  |  |
| Kulnigg, 2008 | FCM | 136 | 40.4 | 40.0 (IQR 19–78) | NR | 8.7 (IQR 5.0–11.5) | 4.0 (IQR 1–32) | 5.0 (IQR 1–399) | Vifor (International) , Inc. | Inflammatory Bowel Disease |
|  | OFS | 60 | 40 | 45.0 (IQR 20–78) | NR | 9.1 (IQR 5.3–11.1) | 6.0 (IQR 1–64) | 6.5 (IQR 1–383) |  |  |
| Li, 2008 | ISC | 70 | 44.3 | 53.6±13.8 | NR | 8.1±8.3 | 22.3±12.4 | 192.6±154.4 | NR | Renal anemia |
|  | FSC | 66 | 39.4 | 54.9±12.6 | NR | 8.2±7.8 | 21.3±16.8 | 194.7±158.4 |  |  |
| Lindgreen, 2009 | ISC | 45 | 40.6 | 42.1±15.0 | NR | 10.5 ±9.0 | 7.1±5.3 | 14.0±17.6 | Renapharma AB, Uppsala, Sweden. | inflammatory bowel disease |
|  | OFS | 46 | 32.6 | 42.8±16.5 | NR | 10.4 ±11.4 | 6.5±4.8 | 12.4±14.5 |  |  |
| Macdougall, 2014a | FCM High Ferritin | 153 | 40.5 | 69.5±12.6 | NR | 10.3±0.7 | 16.2±16.7 | 57.7±48.1 | Vifor Pharma | Chronic kidney disease |
|  | FCM Low Ferritin | 152 | 35.5 | 68.2±13.3 | NR | 10.5±0.8 | 16.1±8.3 | 56.4±49.2 |  |  |
|  | OFS | 308 | 37.7 | 69.3±13.4 | NR | 10.4±0.7 | 15.5±7.6 | 57.3±42.4 |  |  |
| Macdougall, 2014b | FXM | 80 | 49 | 62±15 | NR | NR | NR | NR | AMAG Pharmaceuticals, Inc. | Chronic KidneyDisease |
|  | ISC | 82 | 52 | 63±15 | NR | NR | NR | NR |  |  |
| Macdougall, 2019 | FXM | 196 | 58.2 | 59.3±14.1 | 86.8±23.4 | 10.4 | NR | NR | AMAG Pharmaceuticals | Chronic kidney disease  undergoing hemodialysis |
|  | ISC | 97 | 58.8 | 57.6±13.6 | 83.2±21.0 | 10.3 | NR | NR |  |  |
| Mahey, 2016 | FCM | 30 | 0 | 36.3 ±9.0 | 56.4±12.0 | 7.4±12.3 | NR | 10.0 (IQR 3.9–28.0) | NR | IDA due to abnormal uterine bleeding |
|  | ISC | 30 | 0 | 35.2 ±7.5 | 55.6±9.3 | 7.7±12.0 | NR | 8.8 (IQR 2.3–20.0) |  |  |
| Noronha, 2017 | ISC | 94 | 46.8 | 55.0 (IQR 29-73) | NR | 10.2 (IQR 7.2-11.9) | NR | NR | Tata Memorial Center Research Administration Council | Cancer |
|  | OFS | 98 | 34.7 | 50.0 (IQR 18-73) | NR | 10.1 (IQR 7.2-12.5) | NR | NR |  |  |
| Onken, 2013 | FCM | 246 | 5.3 | 43.1±17.2 | 82.8±22.5 | 10.59±1.0 | 22.1±14.8 | 31.3±67.7 | Luitpold Pharmaceuticals | IDA caused by different etiologies |
|  | OFS | 253 | 5.9 | 43.5±17.7 | 84.2±24.8 | 10.62±1.1 | 22.4±15.1 | 28.2±39.2 |  |  |
|  | FCM | 253 | 5.5 | 43.6±16.9 | 79.5±20.4 | 9.12 ±1.6 | 11.5±12.2 | 25.9±63.8 |  |  |
|  | IVSC | 245 | 5.7 | 42.6±15.5 | 84.7±25.9 | 9.02±1.5 | 10.3±9.7 | 14.9±29.3 |  |  |
| Onken, 2014 | FCM | 1276 | 36.5 | 67.5±13.0 | NR | 10.31±0.833 | 19.79±7.777 | 73.01±64.624 | Luitpold Pharmaceuticals. | Impaired renal function |
|  | ISC | 1285 | 36.3 | 67.2±13.0 | NR | 10.32 ±0.826 | 19.56±7.397 | 75.05±64.116 |  |  |
| Patel, 2024 | FCM | 33 | 0 | 56 (95% CI 53-59) | NR | 9.3 (95% CI 9.14–9.63) | 16.5 (95% CI 11.49-21.52) | 243 (95% CI 188–299) | Amrita Research fund | Cancer |
|  | FAS | 36 | 0 | 54 (95% CI 51-58) | NR | 9.3 (95% CI 9–9.61) | 15 (95% CI 12-17.48) | 215 (95% CI 166-263) |  |  |
| Pieracci, 2014 | ISC | 75 | 77.3 | 41.6 (95% CI 18–83) | NR | 8.9 | 9 | 282.8 | National Trauma Institute  and by Prime Award | Traumatic Critical Illness |
|  | PCB | 75 | 61.3 | 40.4 (95% CI 18–87) | NR | 9.9 | 8 | 316.2 |  |  |
| Provenzano, 2009 | FXM | 114 | 50 | 59.5±14.3 | NR | 10.59±0.67 | 15.71±7.21 | 341±159 ng/mL | AMAG Pharmaceuticals | Hemodialysis |
|  | FFM | 116 | 62.9 | 60.8±13.0 | NR | 10.69±0.57 | 15.91±6.29 | 358±172ng/mL |  |  |
| Qunibi, 2010 | FCM | 147 | 36.1 | 65.4 ± 12.6 | 84.6 ± 23.0 | 10.1 ± 0.74 | 15.4±5.5 | 111.8±85.1 | American Regent | Chronic Kidney Disease |
|  | OFS | 103 | 29.1 | 66.8 ± 13.5 | 89.5 ± 27.2 | 10.0 ± 0.86 | 15.8±5.6 | 104.8±75.1 |  |  |
| Reinisch, 2013 | FDI | 219 | 37 | Med 36 | NR | 9.64±1.65 | 8.2±9.7 | 32.8±90.8 | Pharmacosmos A / S. | Inflammatory bowel diseases |
|  | OFS | 108 | 38 | Med 35 | NR | 9.61±1.82 | 6.2±4.4 | 18.3±36.0 |  |  |
| Roger, 2017 | FCM - High | 154 | 40.3 | 69.5±12.6 | NR | 10.3±0.7 | 16.2±16.6 | 58±48 | Vifor Pharma,Glattbrugg, Switzerland. | Chronic Kidney Disease |
|  | FCM- Low | 150 | 36 | 68.1±13.3 | NR | 10.5±0.8 | 16.1±8.3 | 55±48 |  |  |
|  | OFS | 312 | 37.2 | 69.3±13.4 | NR | 10.4±0.7 | 15.5±7.6 | 57±42 |  |  |
| Saroj Vadhan, 2014 | FXM | 608 | 10.9 | 44.8 ± 13.82 | 78.8± 24.42 | 8.9 ± 0.89 | 7.0±12.9 | 22.6±80.7 | AMAG Pharmaceuticals, Inc. | Iron deficiency anemia |
|  | PCB | 200 | 11 | 46.0± 13.58 | 79.0 ± 23.81 | 8.8 ± 0.89 | 5.4±4.9 | 20.7±59.8 |  |  |
| Schroder, 2005 | ISC | 22 | 22.7 | 35 (IQR 29-41) | 60 (IQR 54-65) | 0.98 (IQR 0.88–1.04) | 6.5 (IQR 4.2–7.8) | 33.8 (IQR 26.9–49.1) | Vifor Int., St. Gallen, Switzerland. | Inflammatory Bowel Disease |
|  | OFS | 24 | 22.7 | 33 (IQR 25-47) | 61 (IQR 55-70) | 0.96 (IQR 0.93–1.01) | 5.5 (IQR 4.0–8.0) | 36.6 (IQR 28.4–42.5) |  |  |
| Tabish, 2024 | FCM | 48 | 89.6 | 45.96±12.07 | 61.92±10.11 | 8.32±1.15 | 0.07 (IQR 0.06–0.10) | 30.00 (IQR 16.00–60.00) | None | Cirrhosis |
|  | CAI | 44 | 84.1 | 46.11±12.25 | 61.52±10.65 | 8.21±1.05 | 0.09 (IQR 0.07–0.14) | 40.00 (IQR 18.60–70.00) |  |  |
| Van Wyck, 2006 | ISC | 79 | 32.9 | 62.3 | 85.6 | 10.2 | 16.4 | 92.6 | American Regent, Inc. | Chronic Kidney Disease |
|  | OFS | 82 | 31.7 | 63.9 | 83.4 | 10.1 | 16.7 | 103.8 |  |  |
| Waziri, 2016 | IDX | 33 | 44.1 | 53.2±12.3 | 75.5±12.9 | 9.3±1.2 | 25.1±6.8 | 121.6±72.0 | Study was provided by the investigators | Chronic kidney disease |
|  | ISC | 34 | 51.5 | 51±13.0 | 71.2±17.6 | 9.1±1.1 | 23.8±7.8 | 100.7±83.5 |  |  |
| Wolf, 2018 | FXM | 997 | 25.5 | 55.6±17.3 | 84.3±24.9 | 10.4±1.5 | 9 (95% CI 5.0–16.0) | 32.3 (95% CI 13.4–121.7) | AMAG Pharmaceuticals, Inc. | Previously did not tolerate or insufficiently responded to oral iron |
|  | FCM | 1000 | 22.4 | 54.8±17.0 | 85.8±26.0 | 10.4±1.5 | 9 (95% CI 5.0–17.0) | 33.9 (95% CI 12.3–123.2) |  |  |
| Wolf, 2021a | FDI | 63 | 3.2 | 43.9±10.4 | 80.6±16.6 | 9.8±1.3 | 5.6 (IQR 3.5-9.7) | 6.1 (IQR 2.9-12.9) | Pharmacosmos A/S | Anemia caused by different etiologies |
|  | FCM | 60 | 5 | 46.3±11.6 | 77.4±20.2 | 9.6±1.3 | 4.7 (IQR 3.6-7.7) | 4.8 (IQR 3.1-7.5) |  |  |
| Wolf, 2021b | FDI | 62 | 6.5 | 42.2±12.9 | 90.1±29.2 | 9.6±1.2 | 5.2 (IQR 3.5-8.8) | 4.8 (IQR 2.8-8.7) | Pharmacosmos A/S | Anemia caused by different etiologies |
|  | FCM | 57 | 5.3 | 43.1±11.5 | 84.2±20.1 | 9.3±1.4 | 4.8 (IQR 3.2-9.2) | 5.1 (IQR 2.7-8.8) |  |  |
| Zahr, 2024 | ISC | 13 | 46.2 | 55.79±18.53 | 69.64±11.1 | 10.03±0.66 | 11.33±3.05 | 60.71±35.89 | None | Chronic Kidney Disease |
|  | OLS | 14 | 28.6 | 59.29±14.34 | 73.29±12.99 | 9.82±1.21 | 12.89±9.3 | 64.45±37.41 |  |  |
| Zoller, 2023 | FDI | 48 | 45.8 | 42.3±14.1 | 79.8±15.4 | 10.5± 15 | 9.3±8.4 | 9.5±9.6 | Pharmacosmos A/S | Inflammatory bowel disease |
|  | FCM | 49 | 49 | 41.9±14.7 | 80.6±16.6 | 10.4±14 | 7.1±4.4 | 14.6±28.7 |  |  |

**Legend:** NR: - No reported, CAI - Carbonil iron tablet, CTL – Control, FAS - Ferrous ascorbate, FCM - Ferric carboxymaltose, FDI - Ferric derisomaltose, FFM - Ferrous fumarate, FSC - Ferrous succinate, FXM – Ferumoxytol, IDX - Iron dextran, IQR -interquartile range, ISC - Iron sucrose, ISFG - Iron sucrose or sodium ferric gluconate, IVSC - Intravenous Standard-of-care, OFM - Oral ferric maltol, OFS - Oral Ferrous Sulfate, OLS - Oral lipossomal iron, PCB – Placebo, SCA - Standard of care, SFO - Saccharated ferric oxide, SGC – Sodium ferric gluconate complex, SUI - Sucrosial Iron.

# Supplement 8 – Risk of bias Appraisal

S8.1 - Outcome Hemoglobin


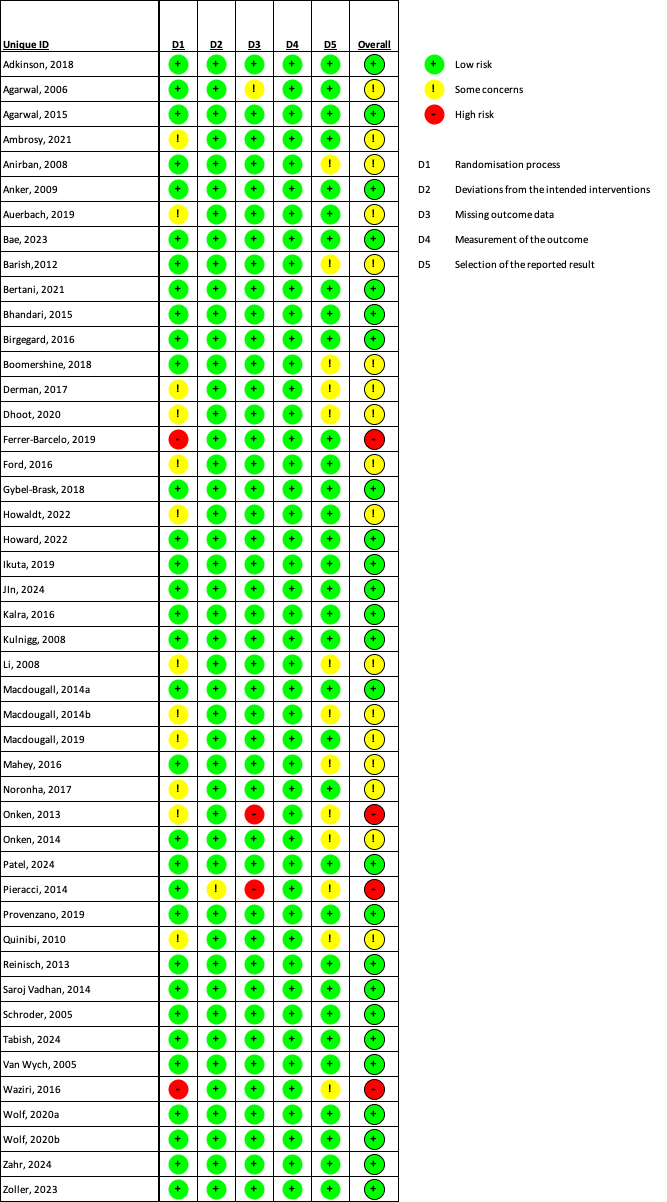


S8.2 - Outcome Transferrin Saturation


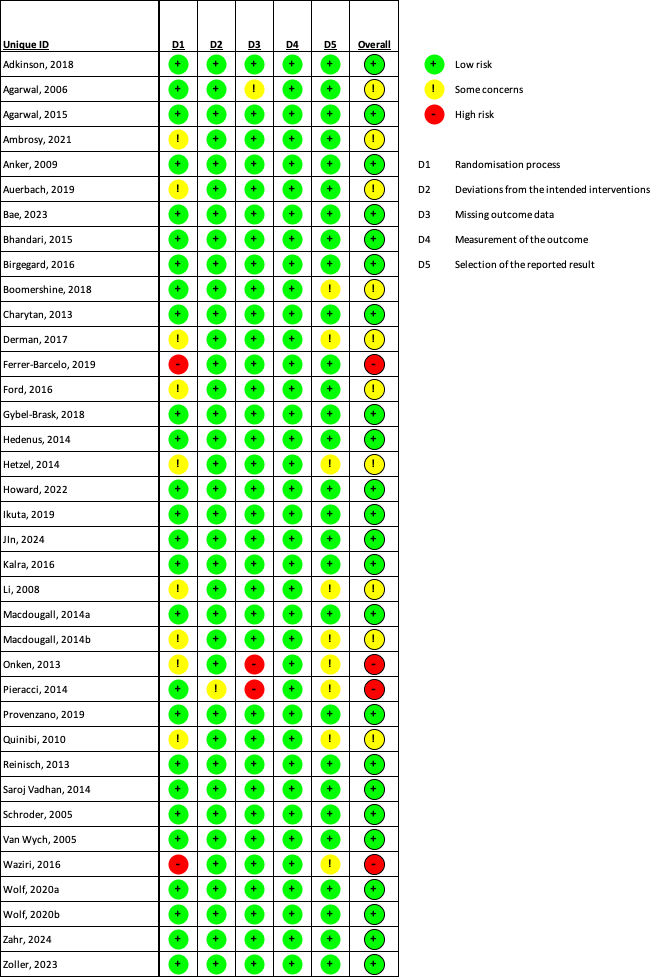


S8.3 - Outcome Ferritin


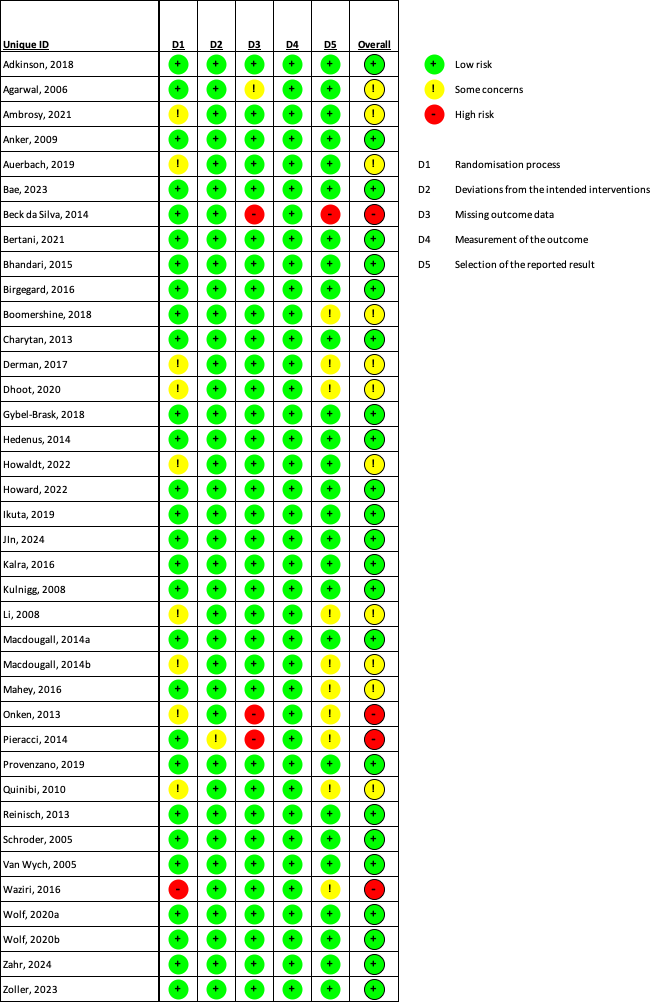


S8.4 - Outcome Any Adverse Event


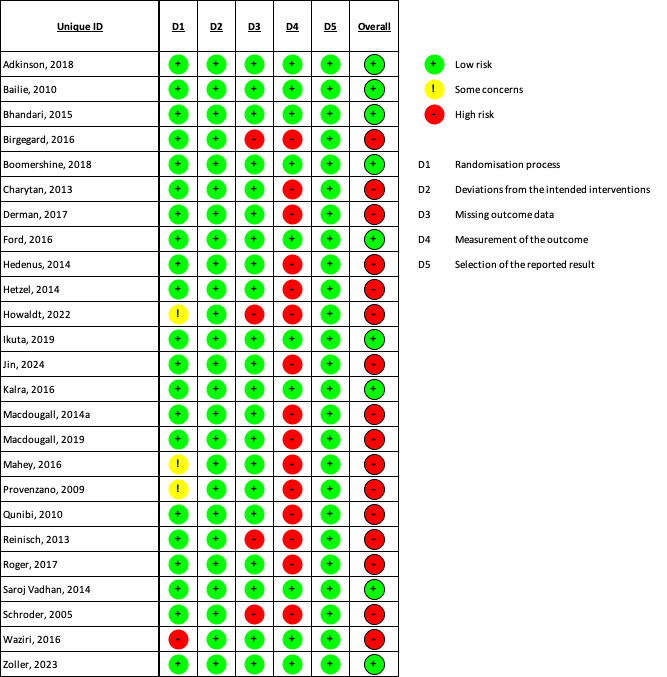


S8.5 - Outcome Serious Adverse Event


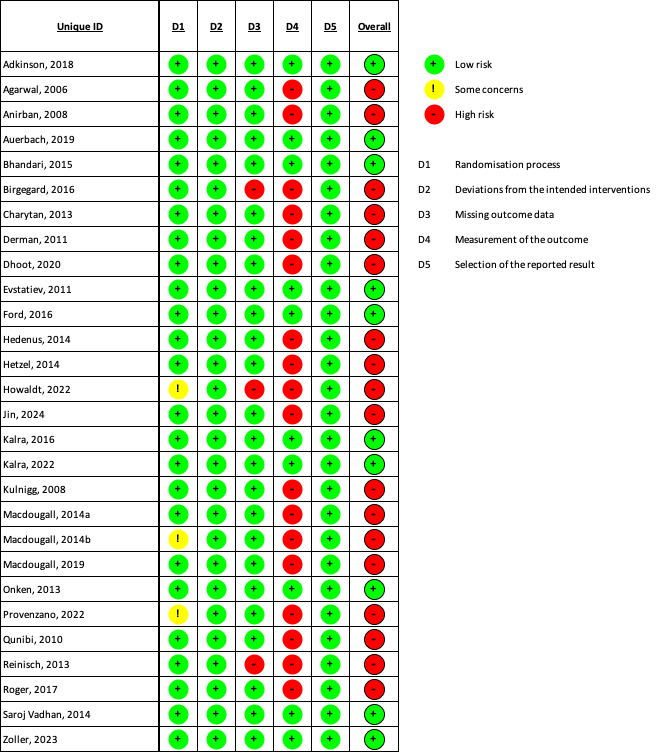


S8.6 - Outcome Hypophosphatemia


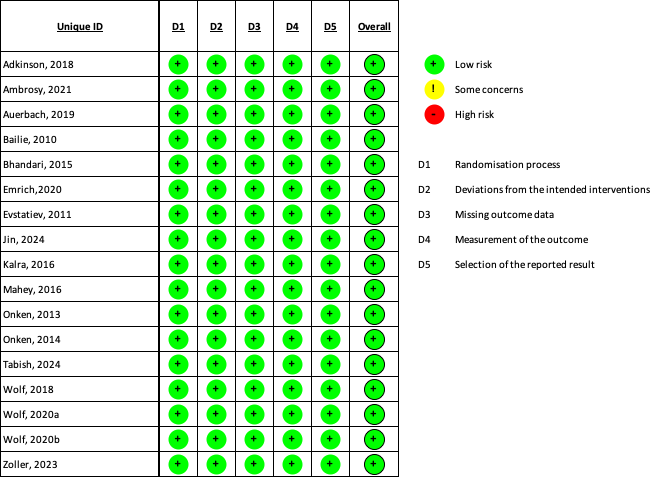


# Supplement 9 – Geometry of Network

**S9.1 – Hemoglobin change – Overall analysis**


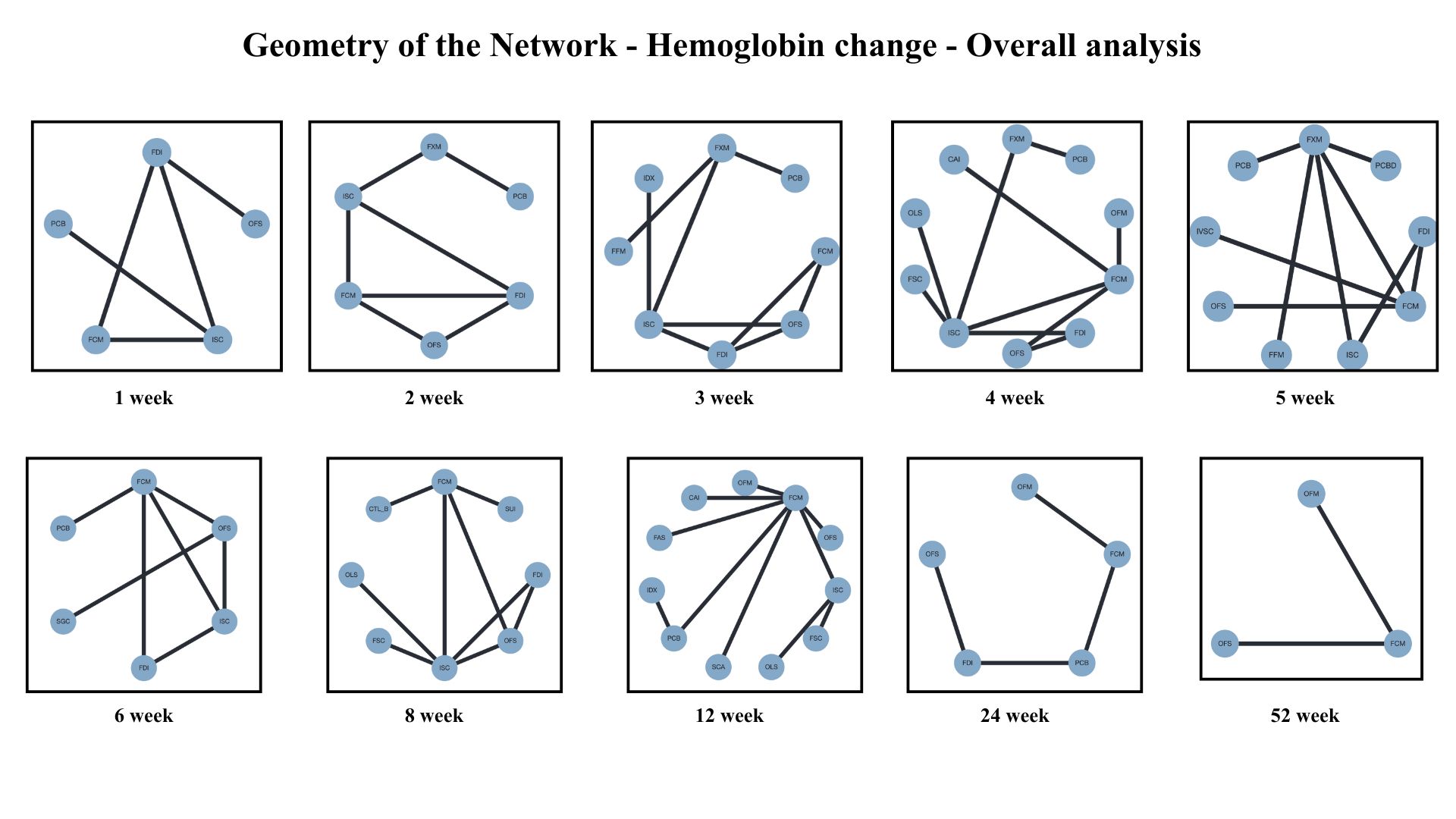


**S9.2 – Hemoglobin change – Renal analysis**


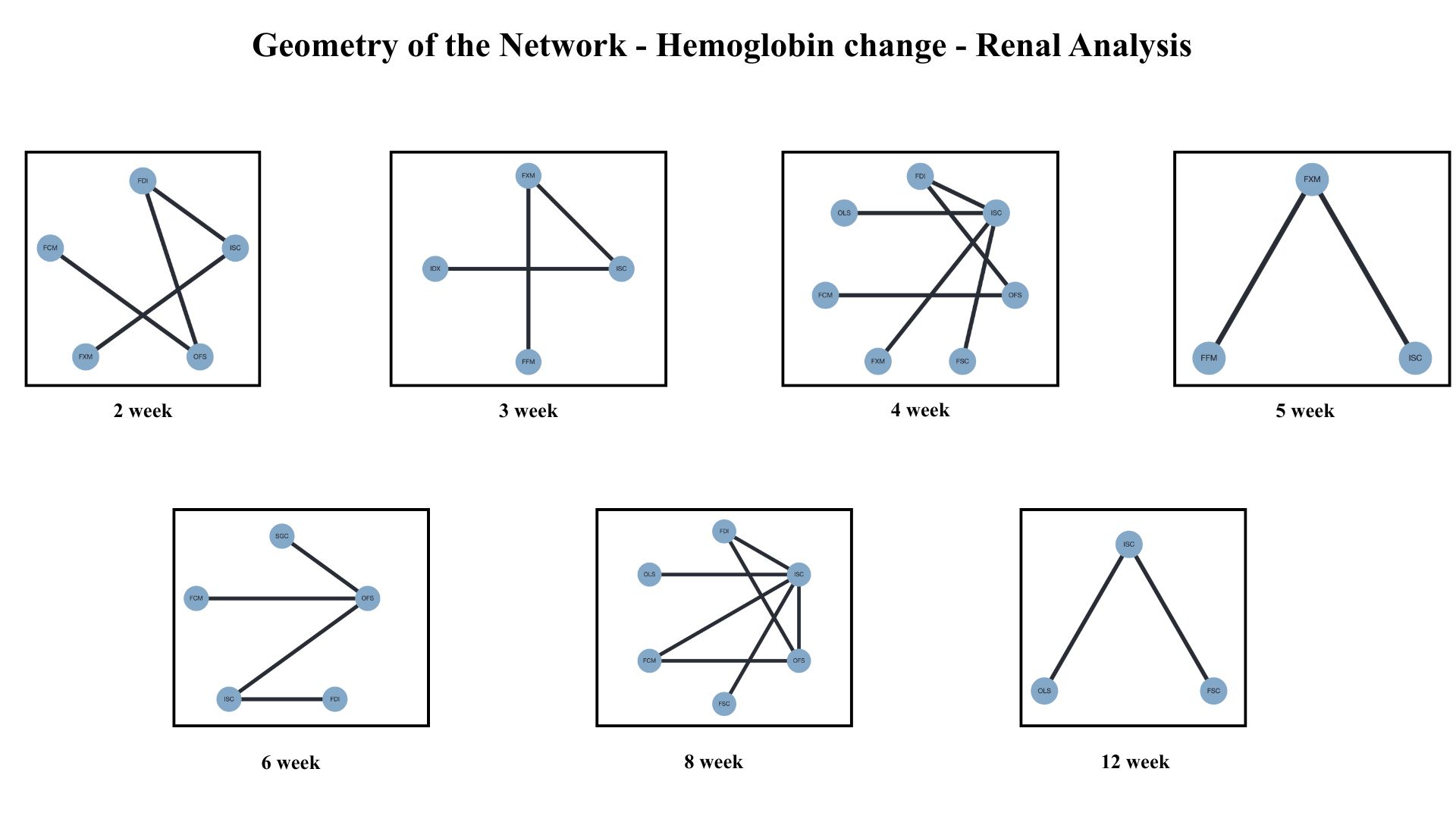


**S9.3 – Hemoglobin change – GI analysis**


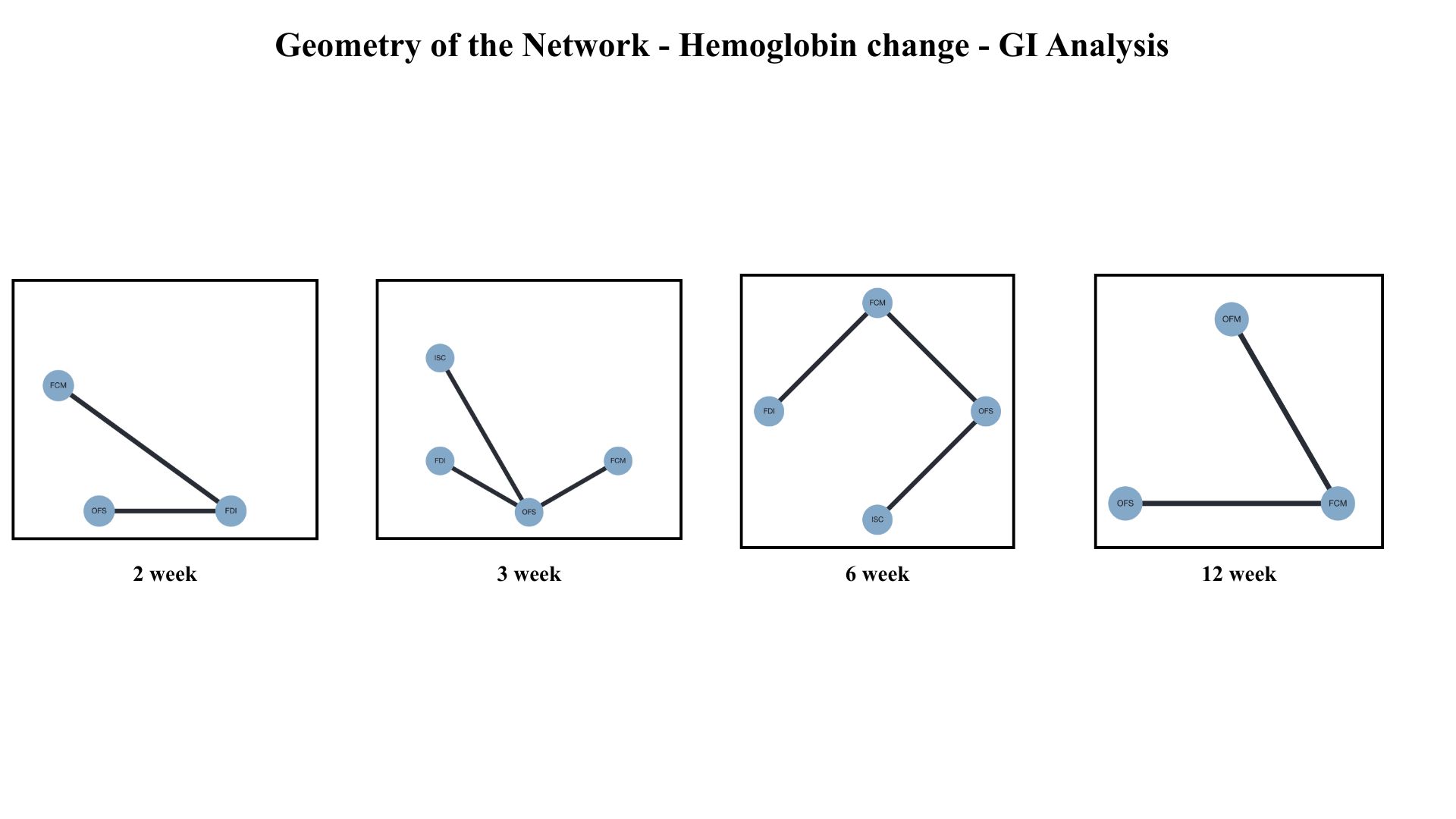


**S9.4 – Ferritin change – Overall analysis**


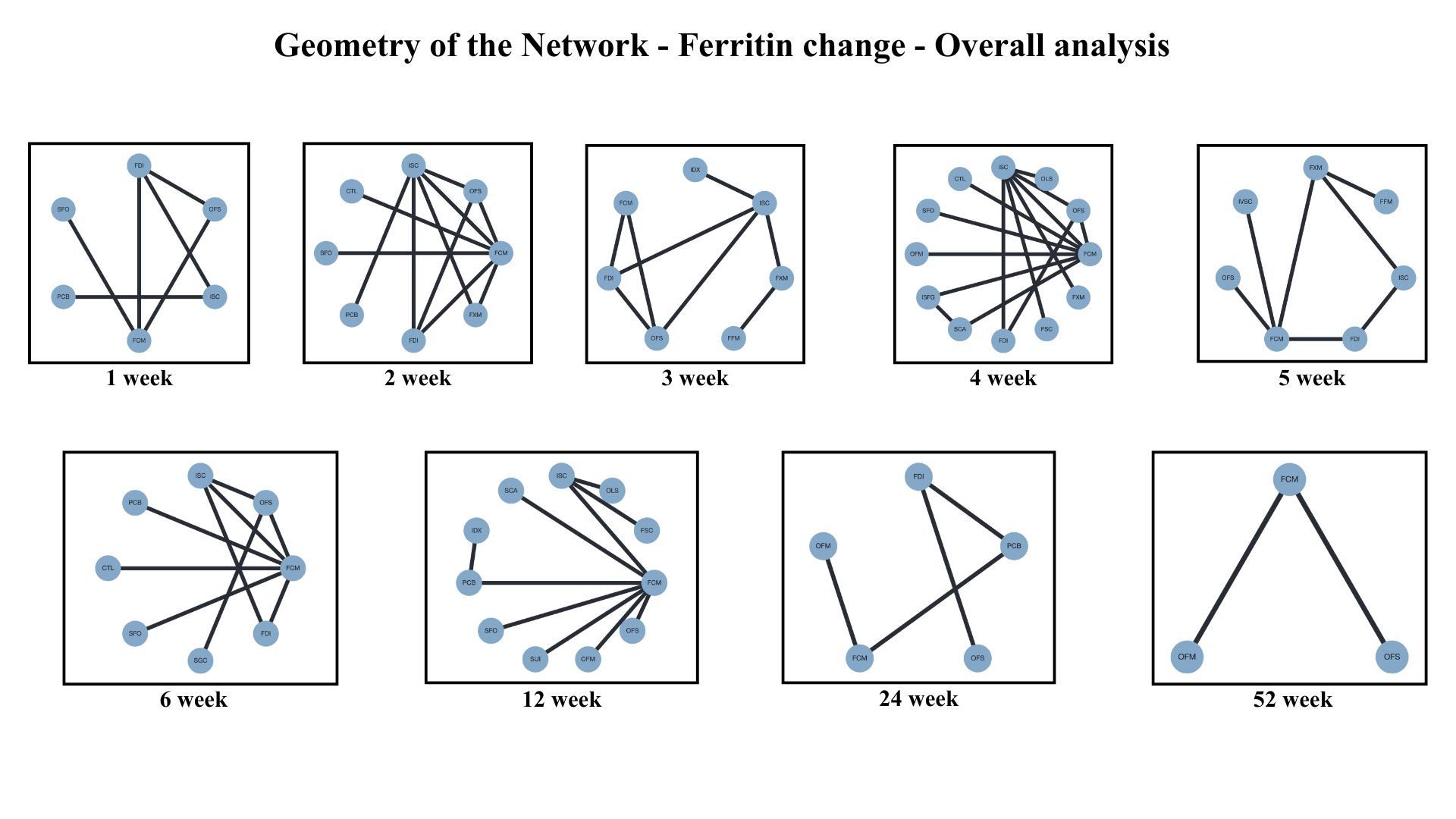


**S9.5 – Ferritin change – Renal analysis**


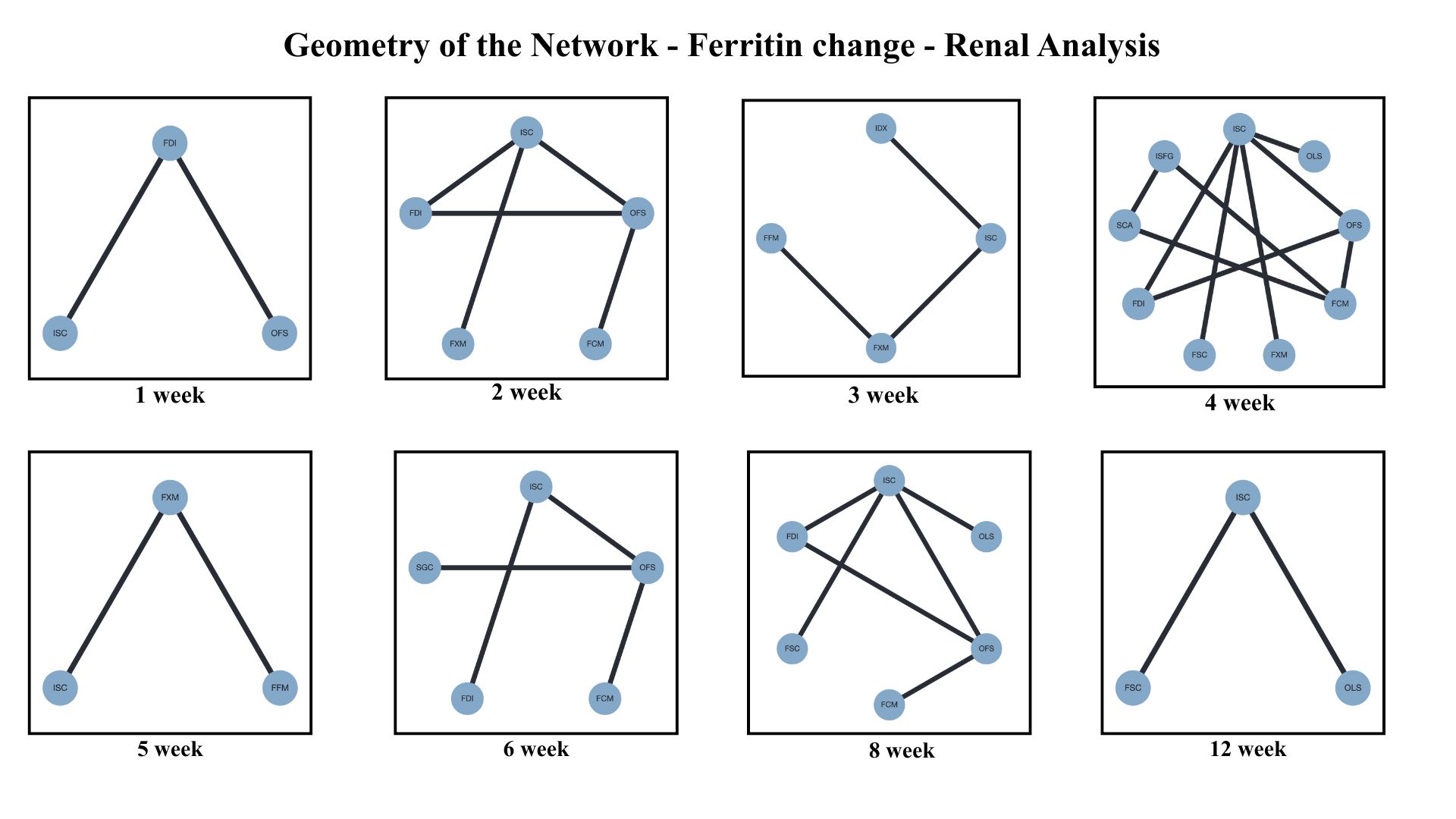


**S9.6 – Ferritin change – GI analysis**


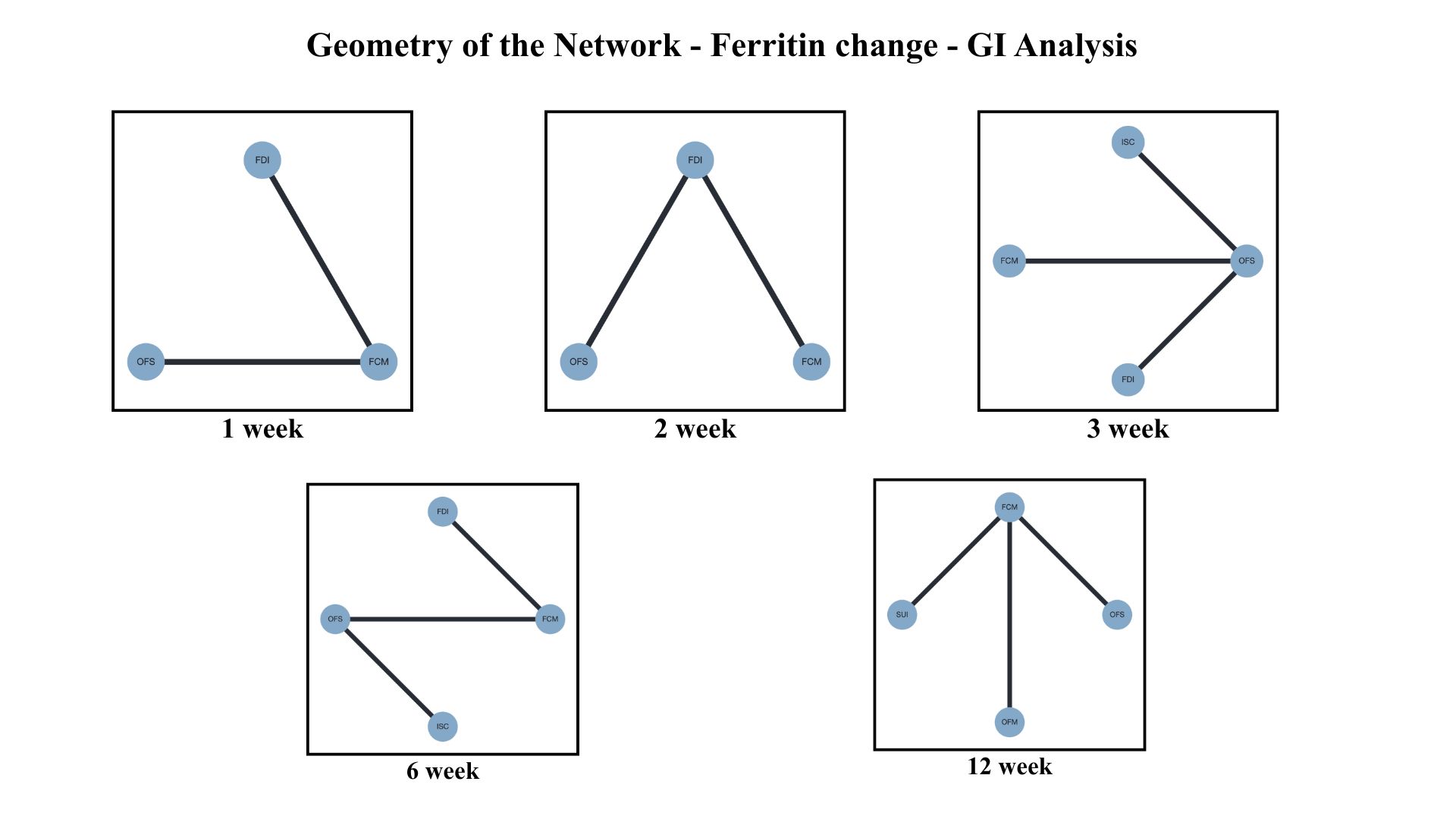


**S9.7 – Transferrin Saturation change – Overall analysis**


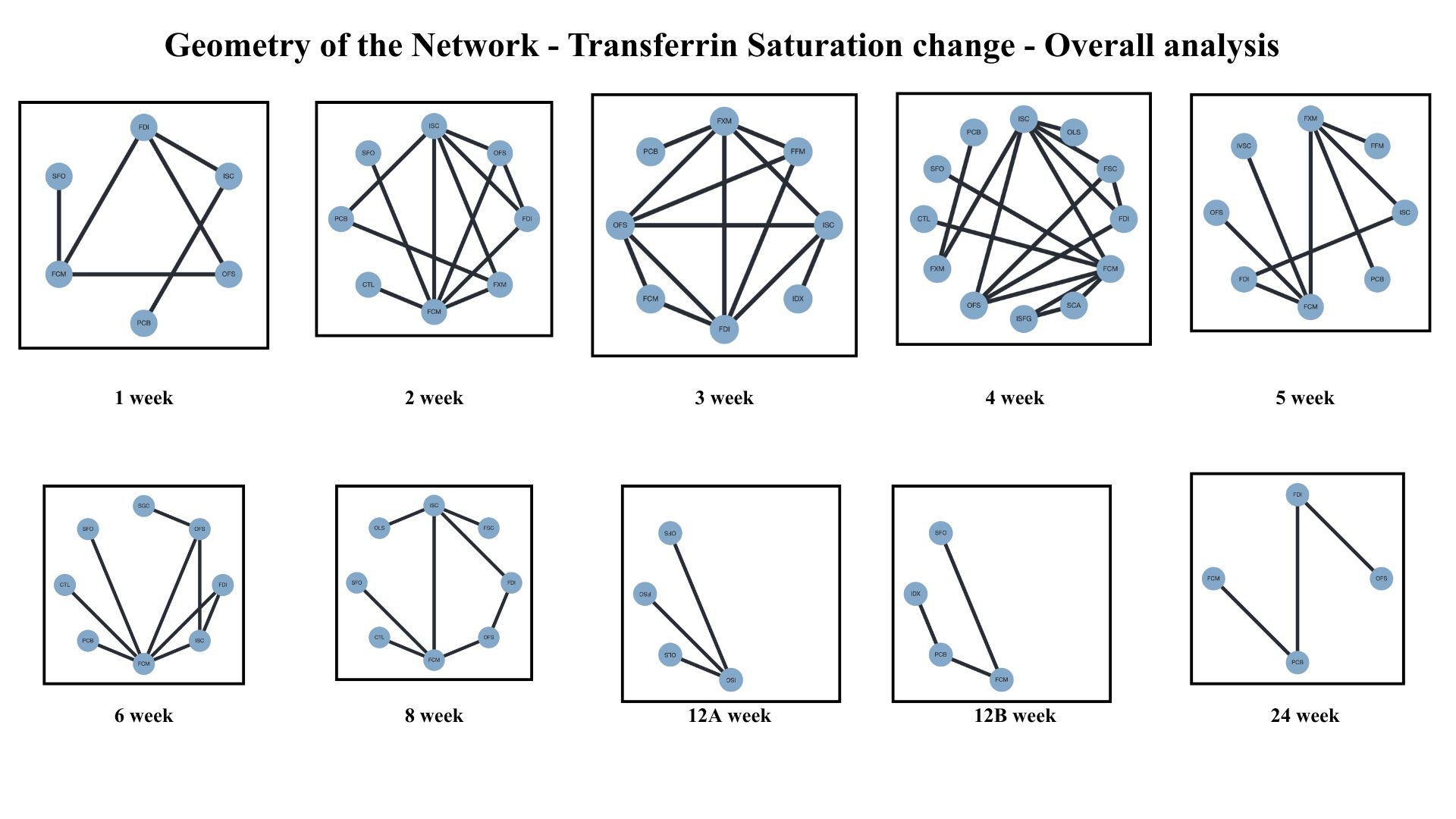


**S9.8 – Transferrin Saturation change – Renal analysis**


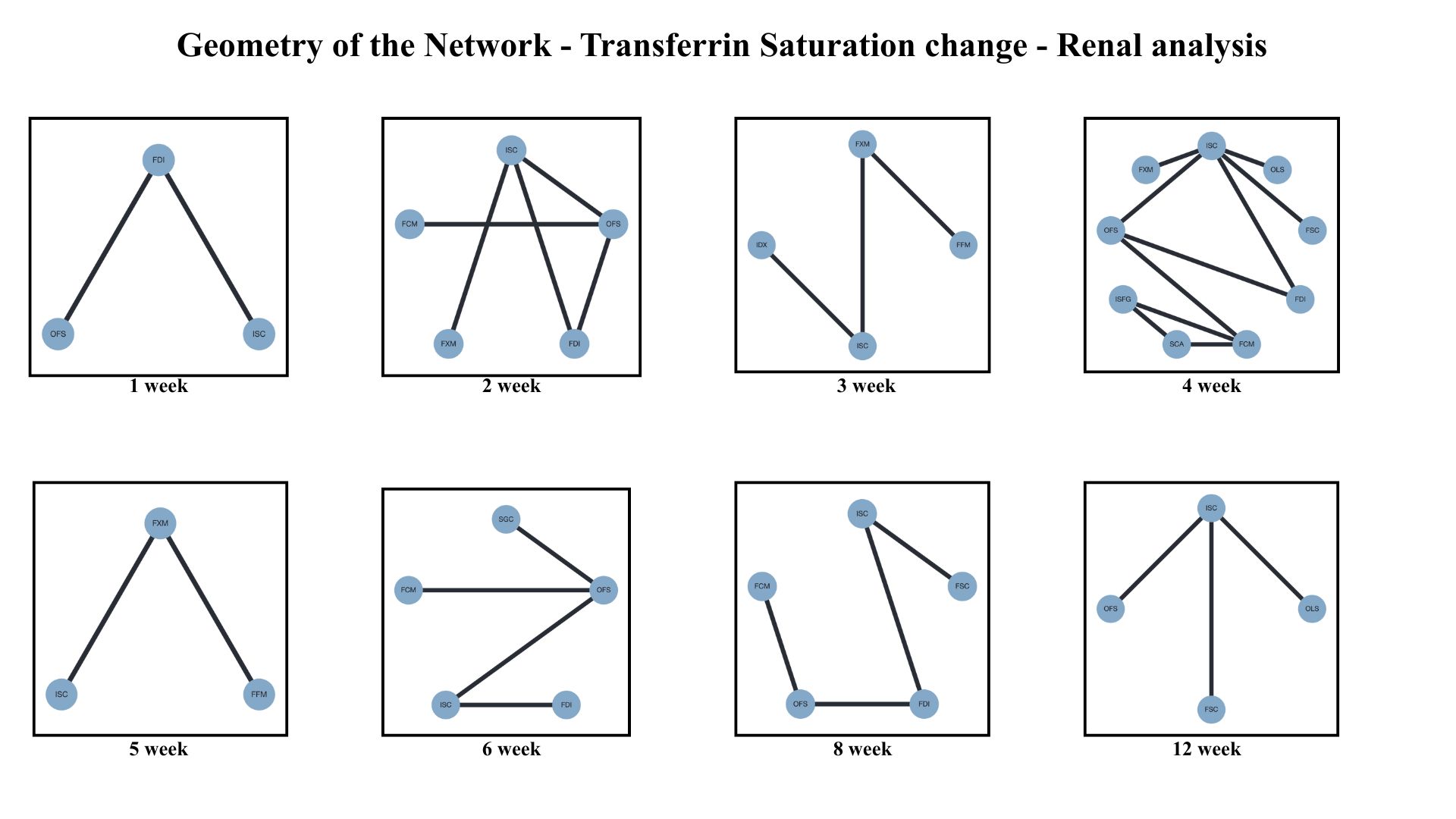


**S9.9 – Transferrin Saturation change – GI analysis**


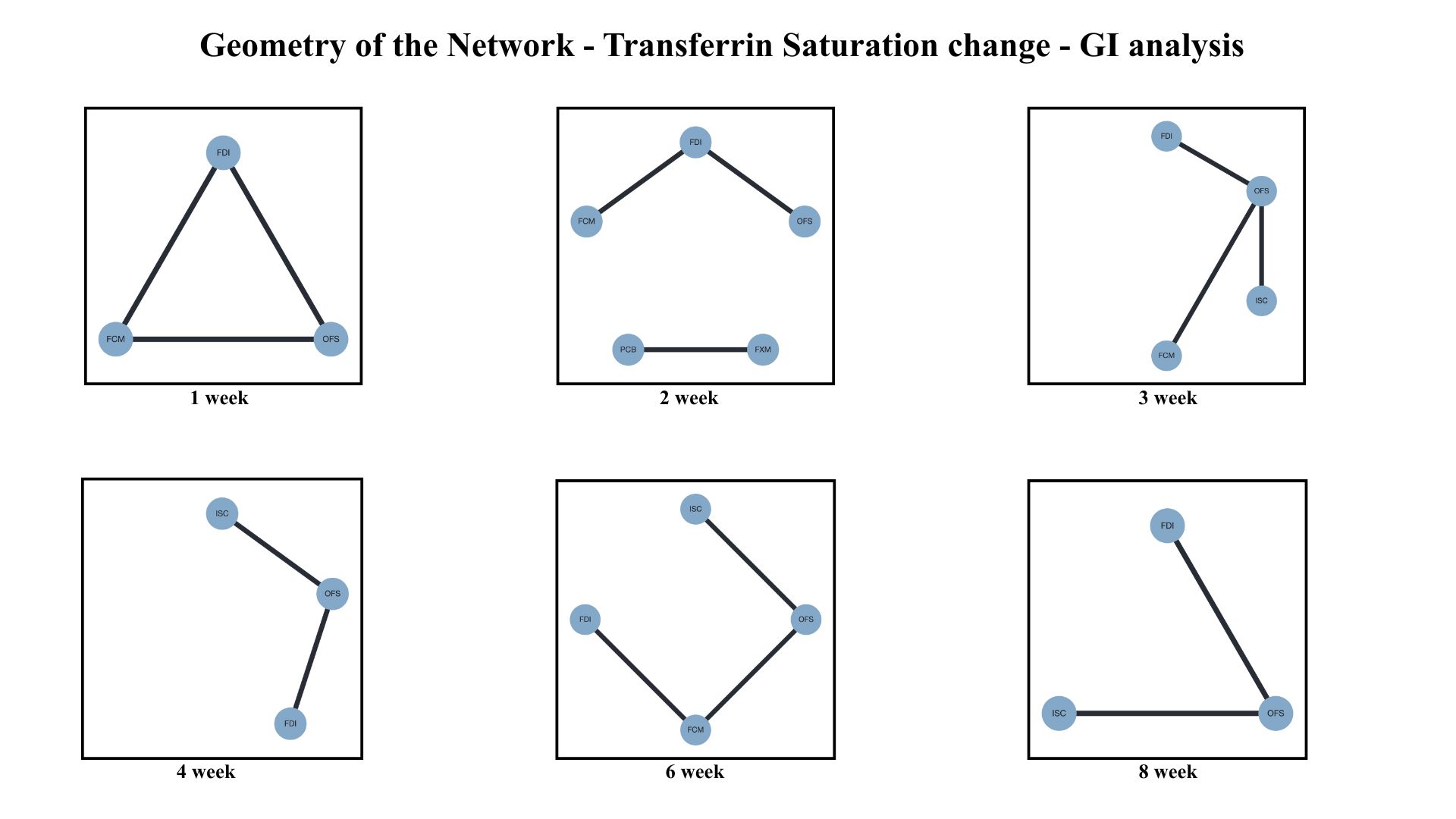


**S9.10 – Any Adverse Events**


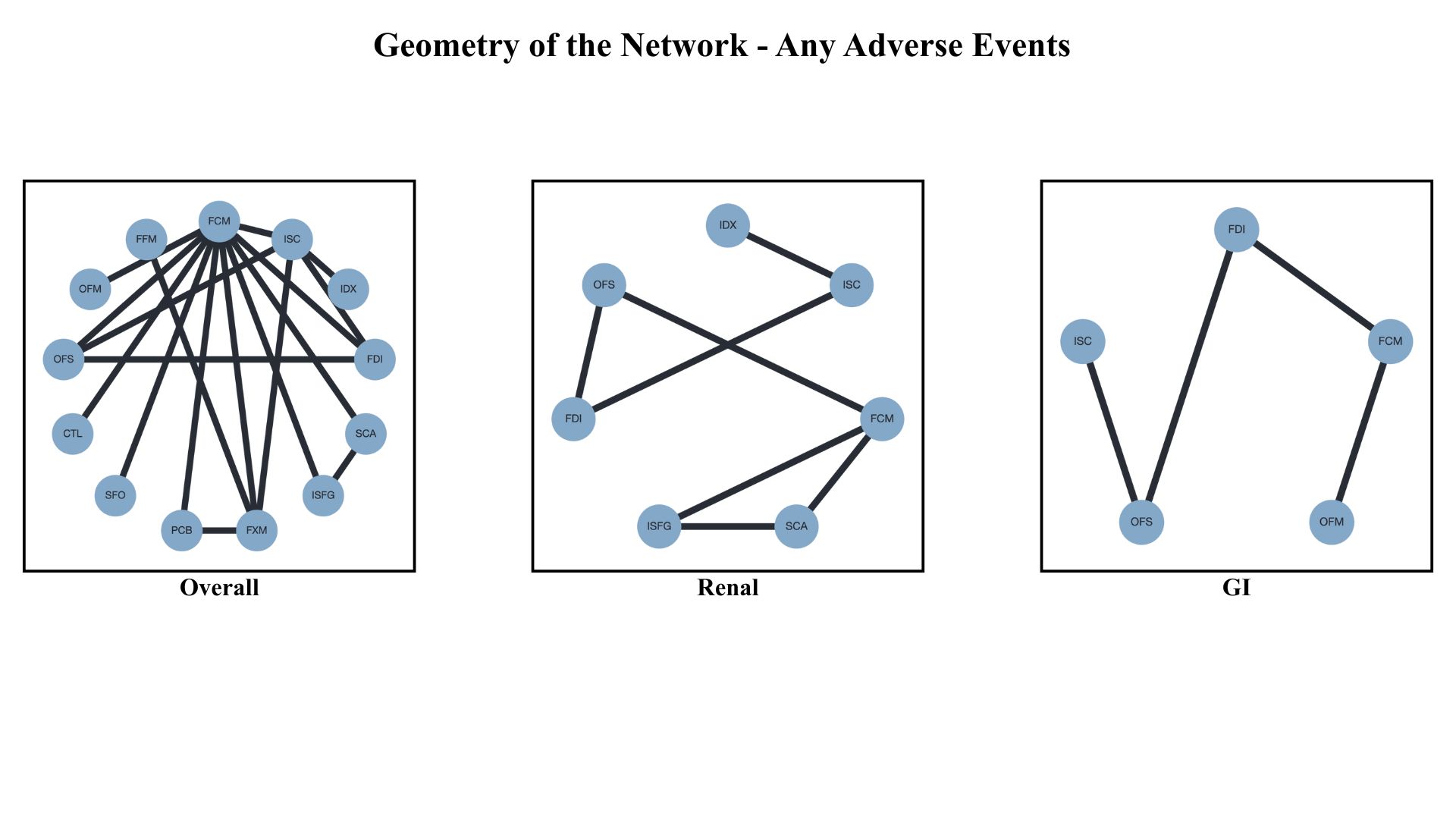


**S9.11 – Serious Adverse Events**


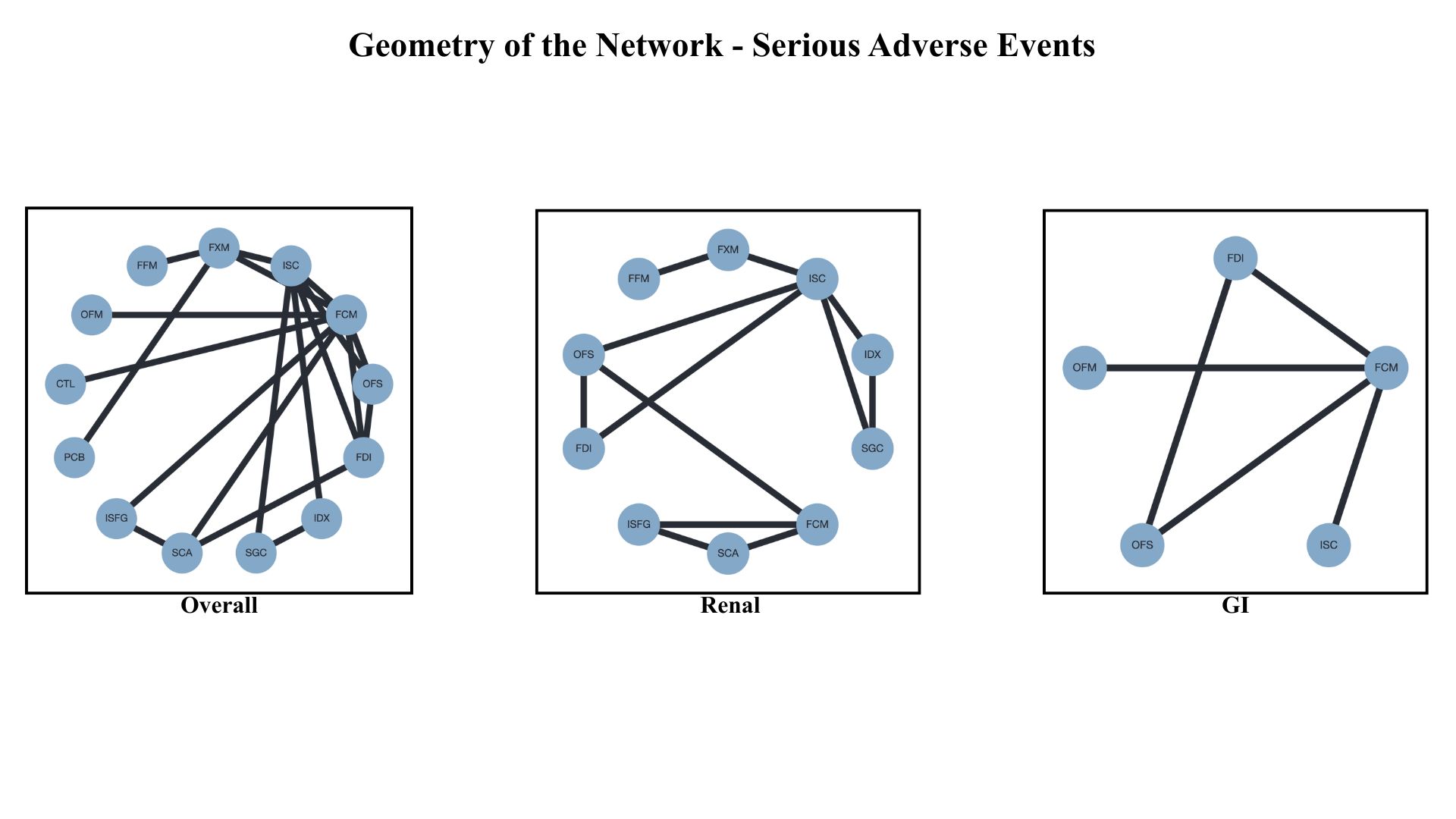


**S9.12 – Hypophosphatemia**


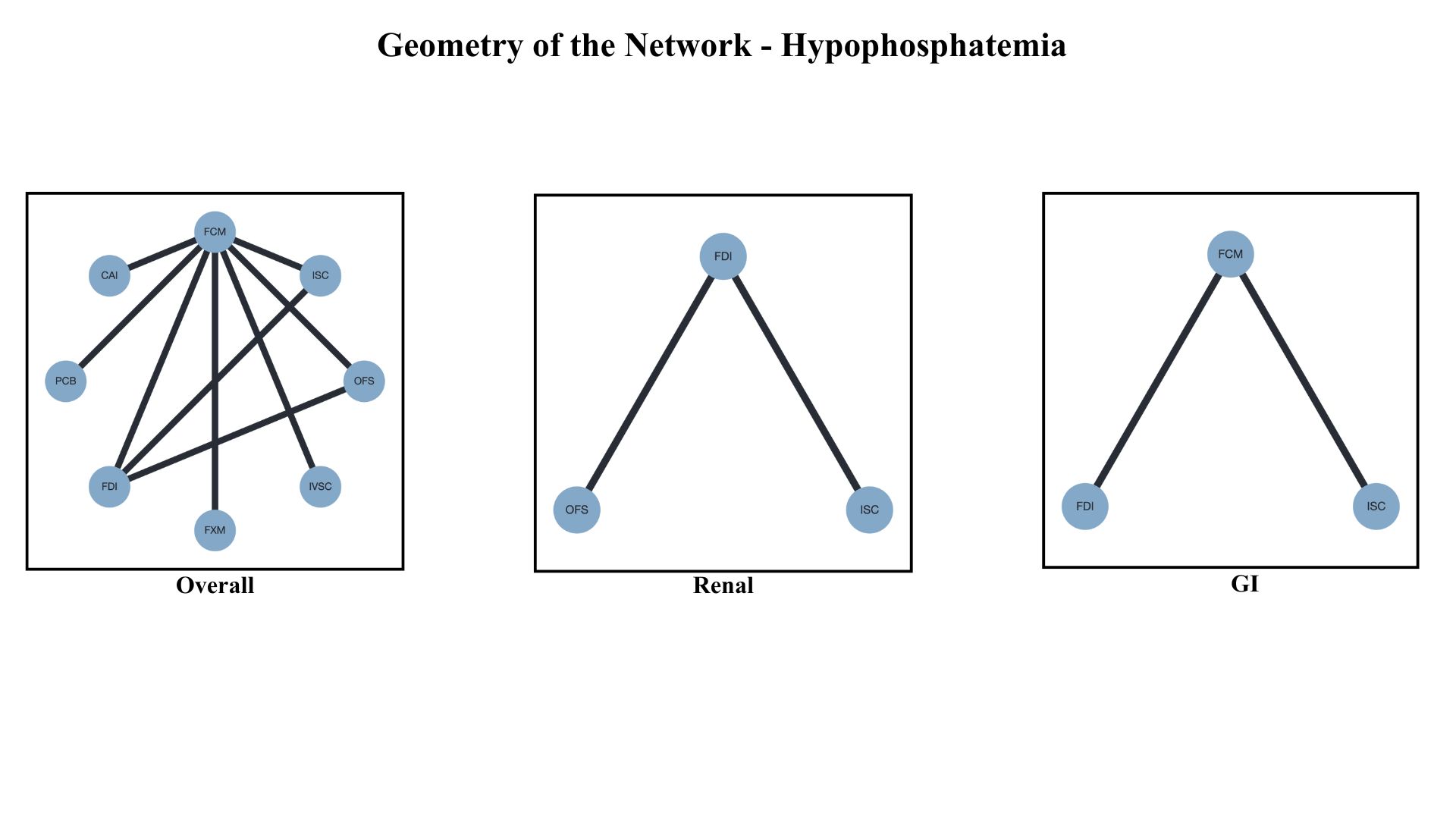


# Supplement 10 – Transitivity Assumption

S10.1 – Hemoglobin change - Overall analysis.


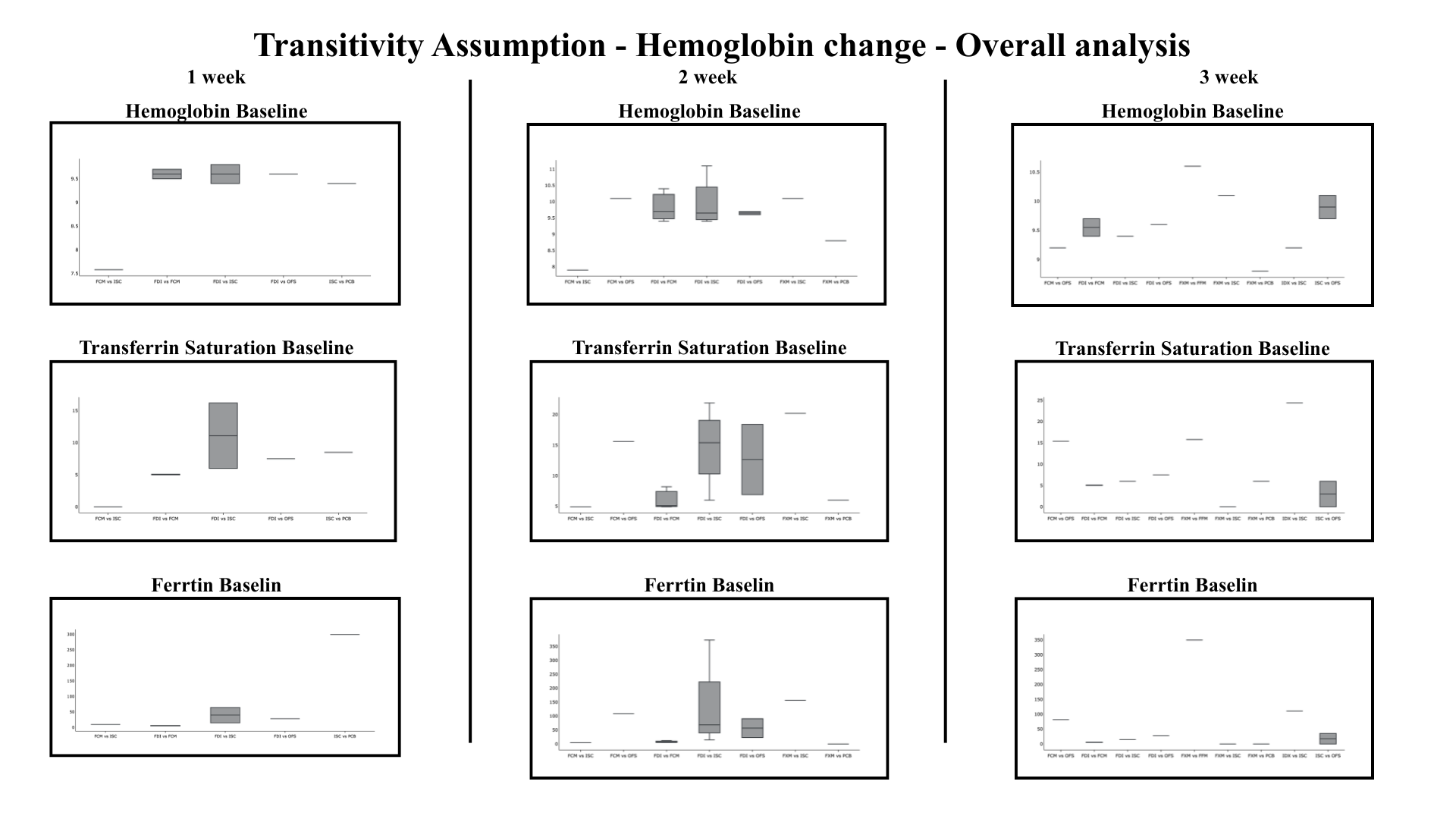


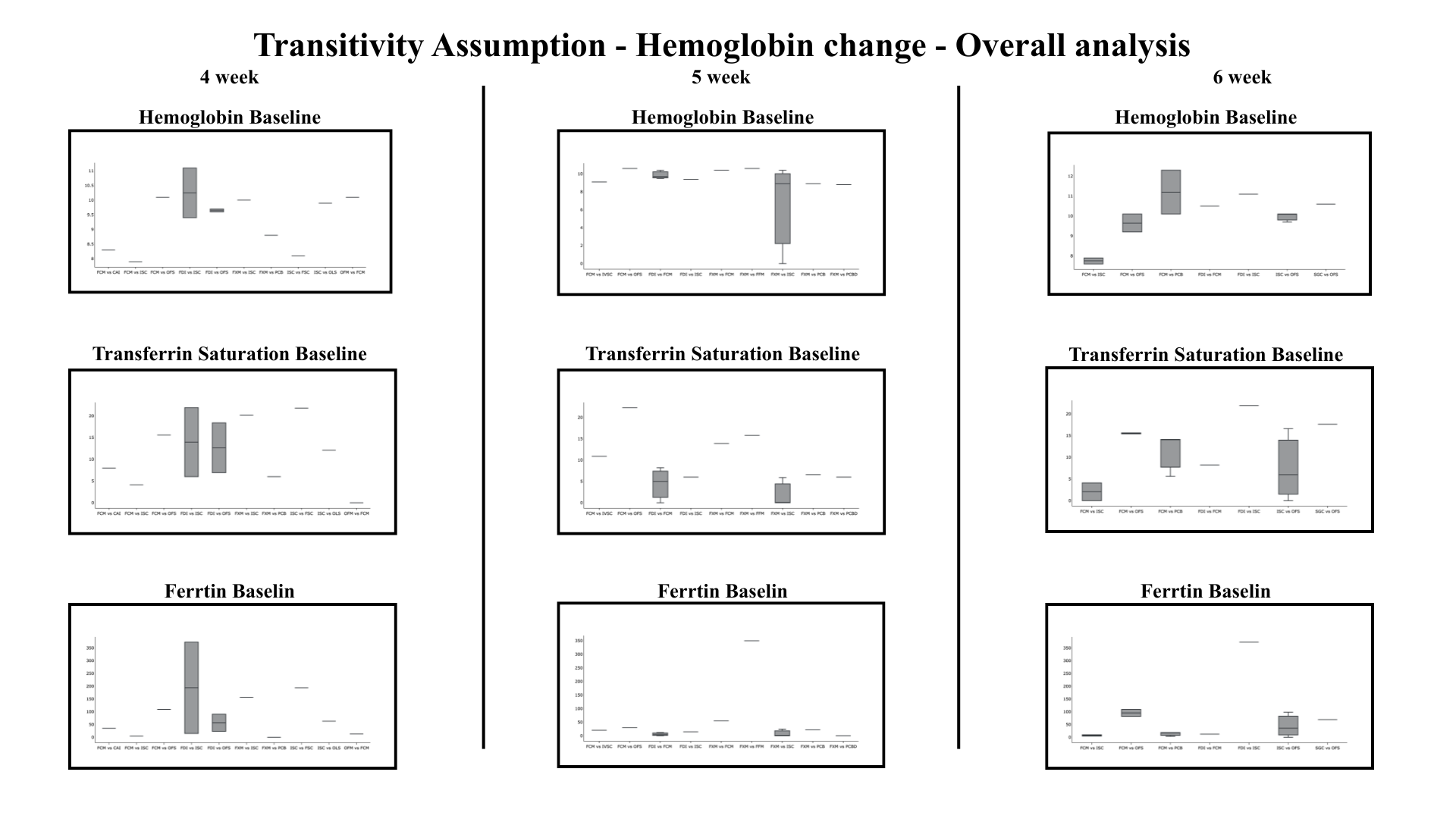


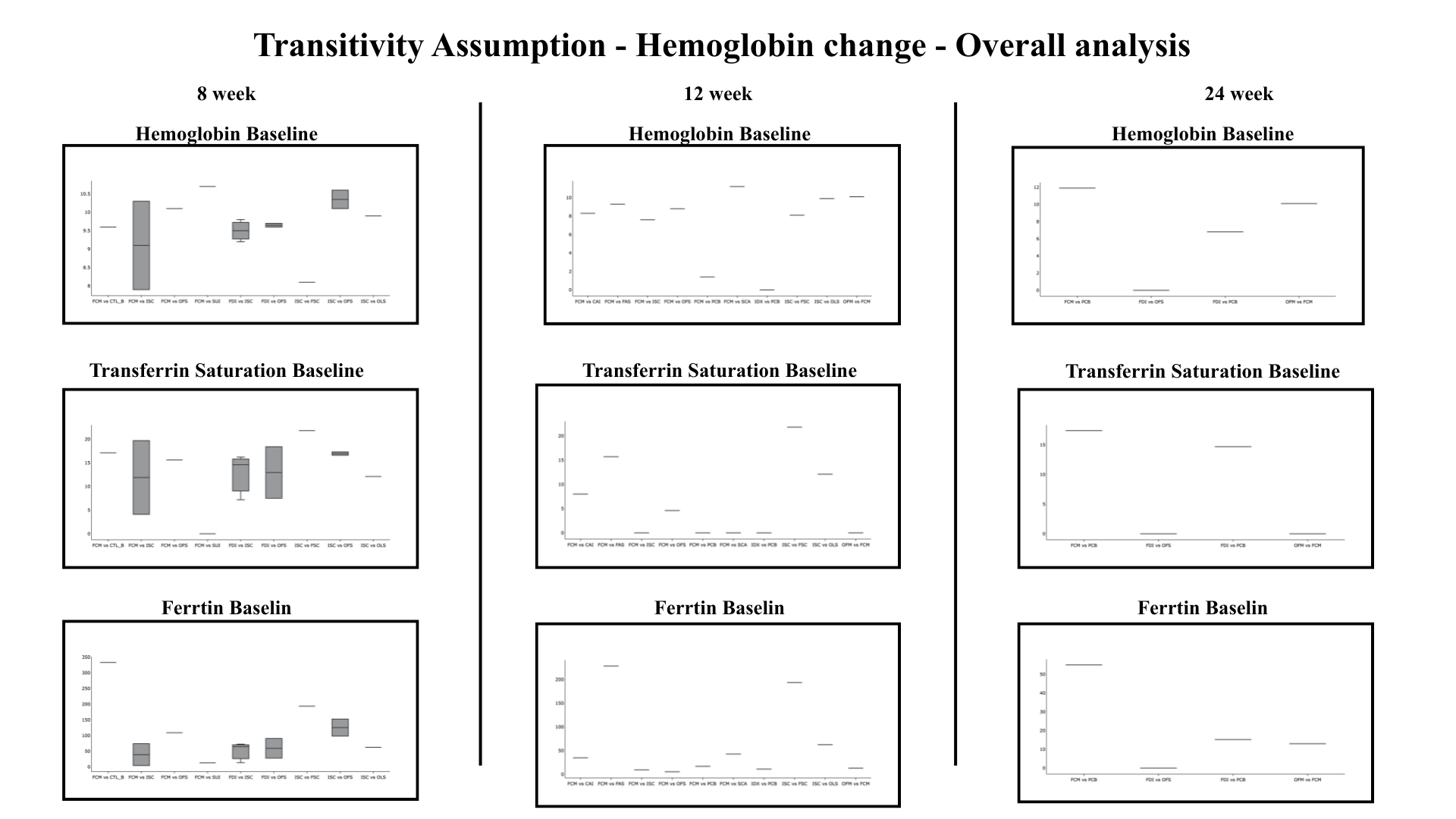


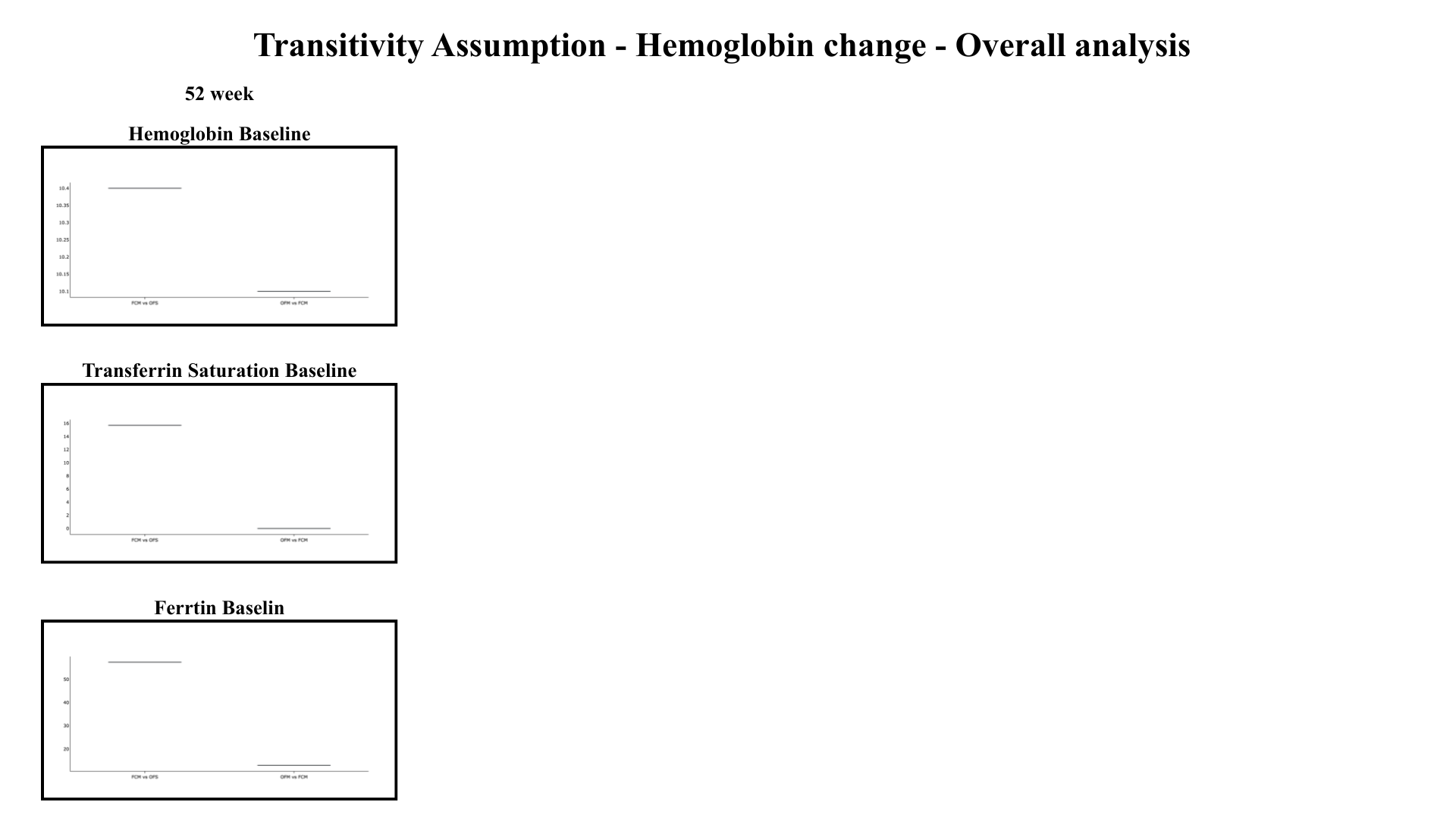


S10.2 – Hemoglobin change - GI analysis.


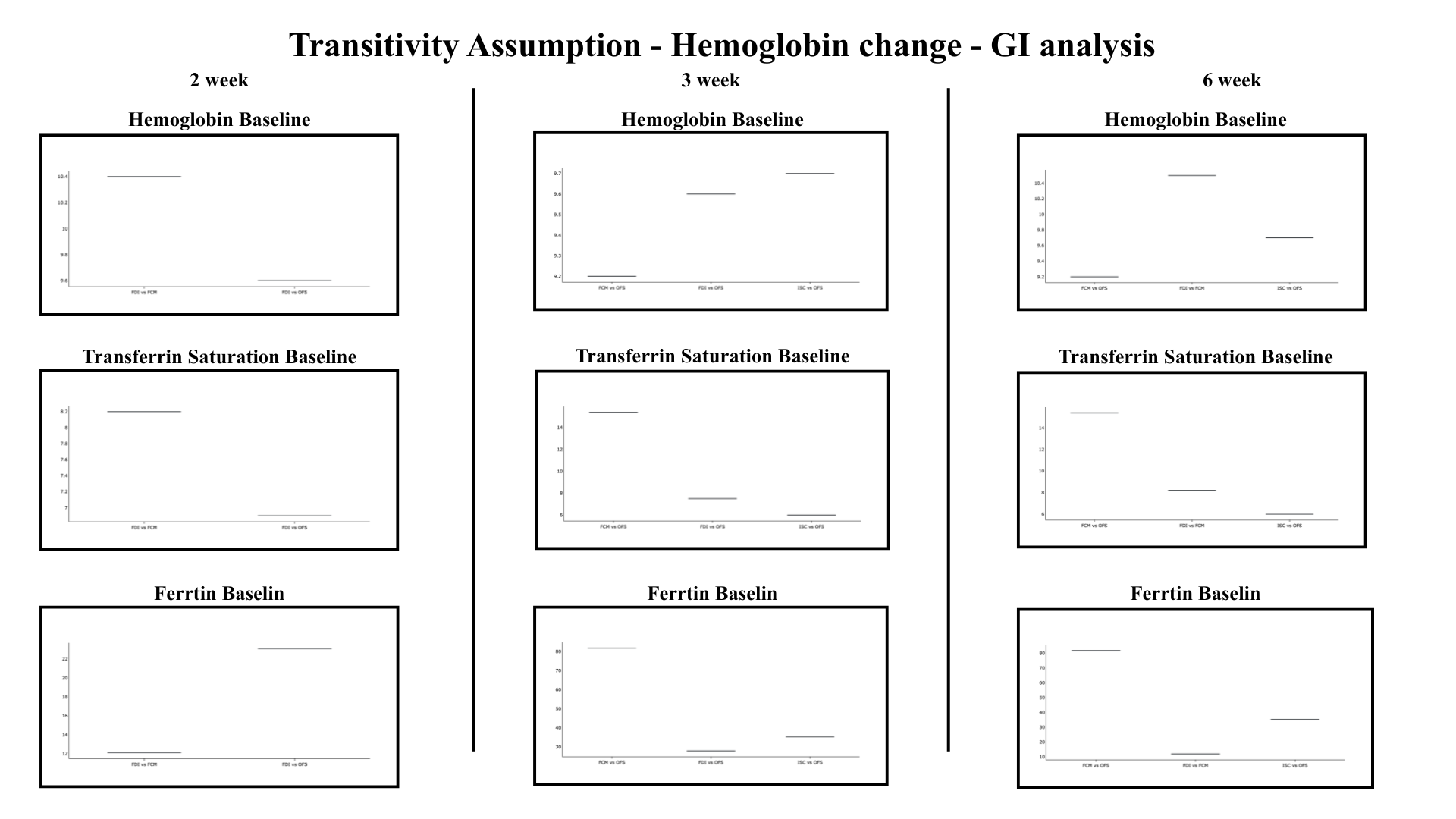


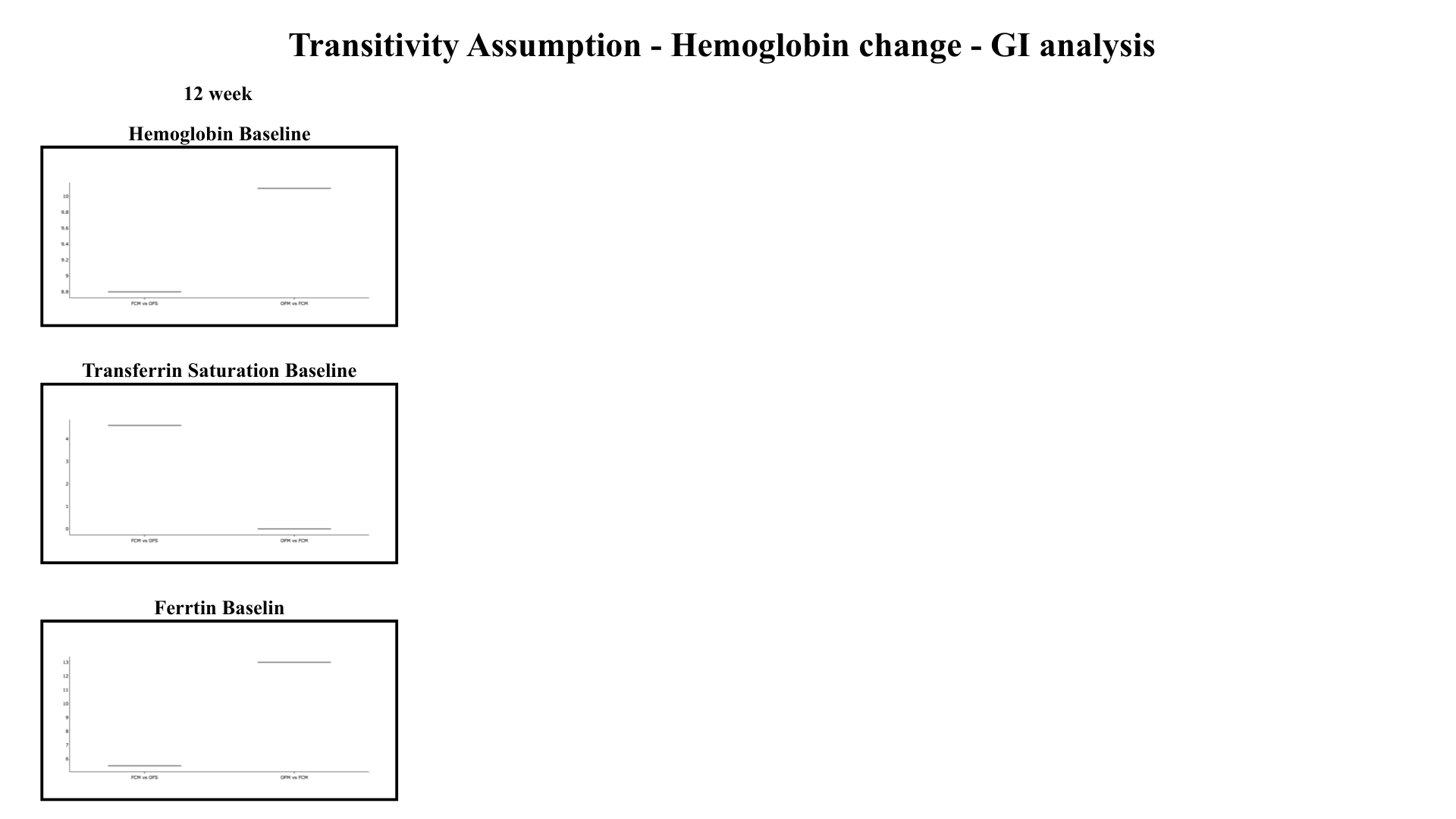


S10.3 – Hemoglobin change - Renal analysis.


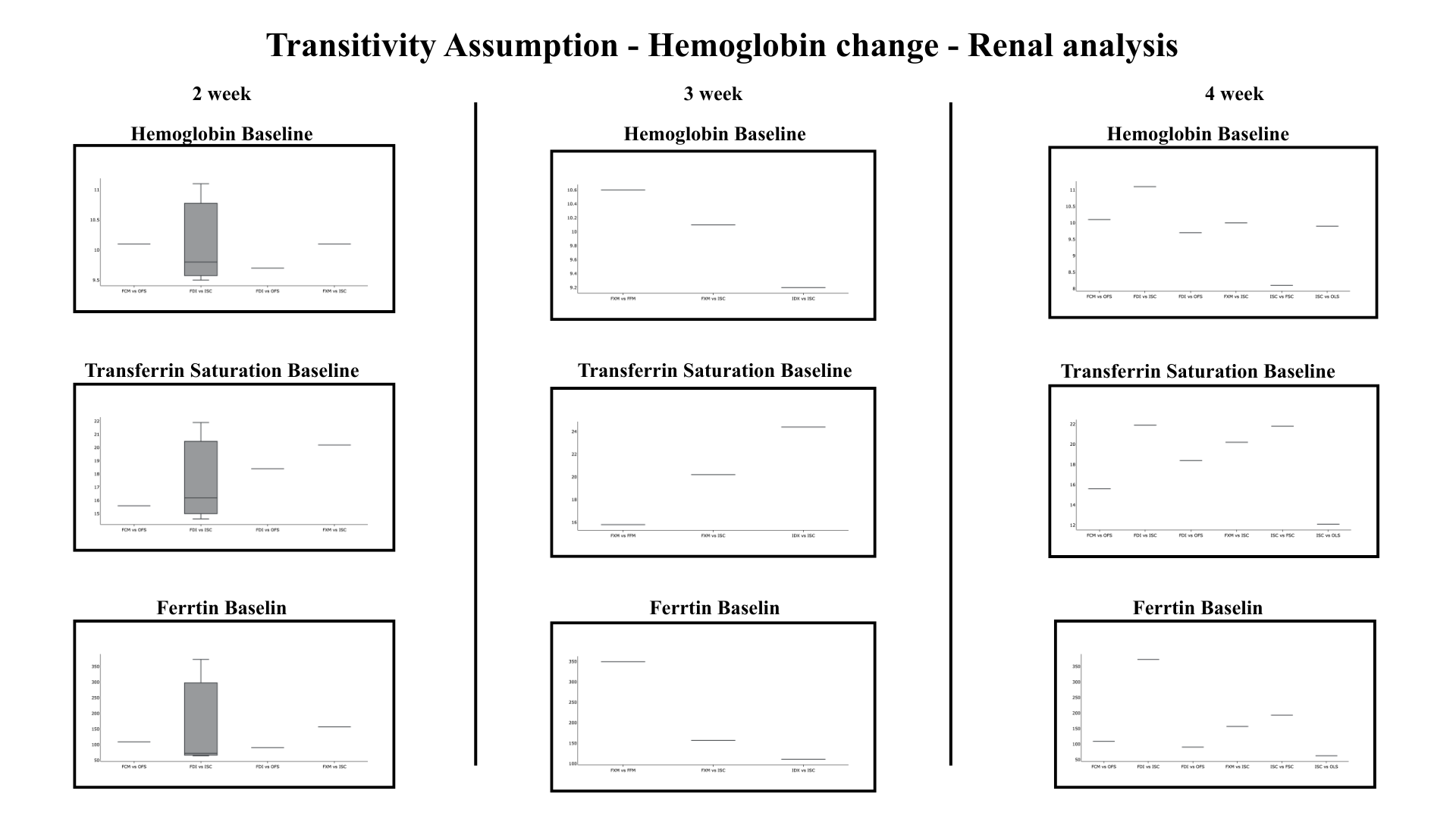


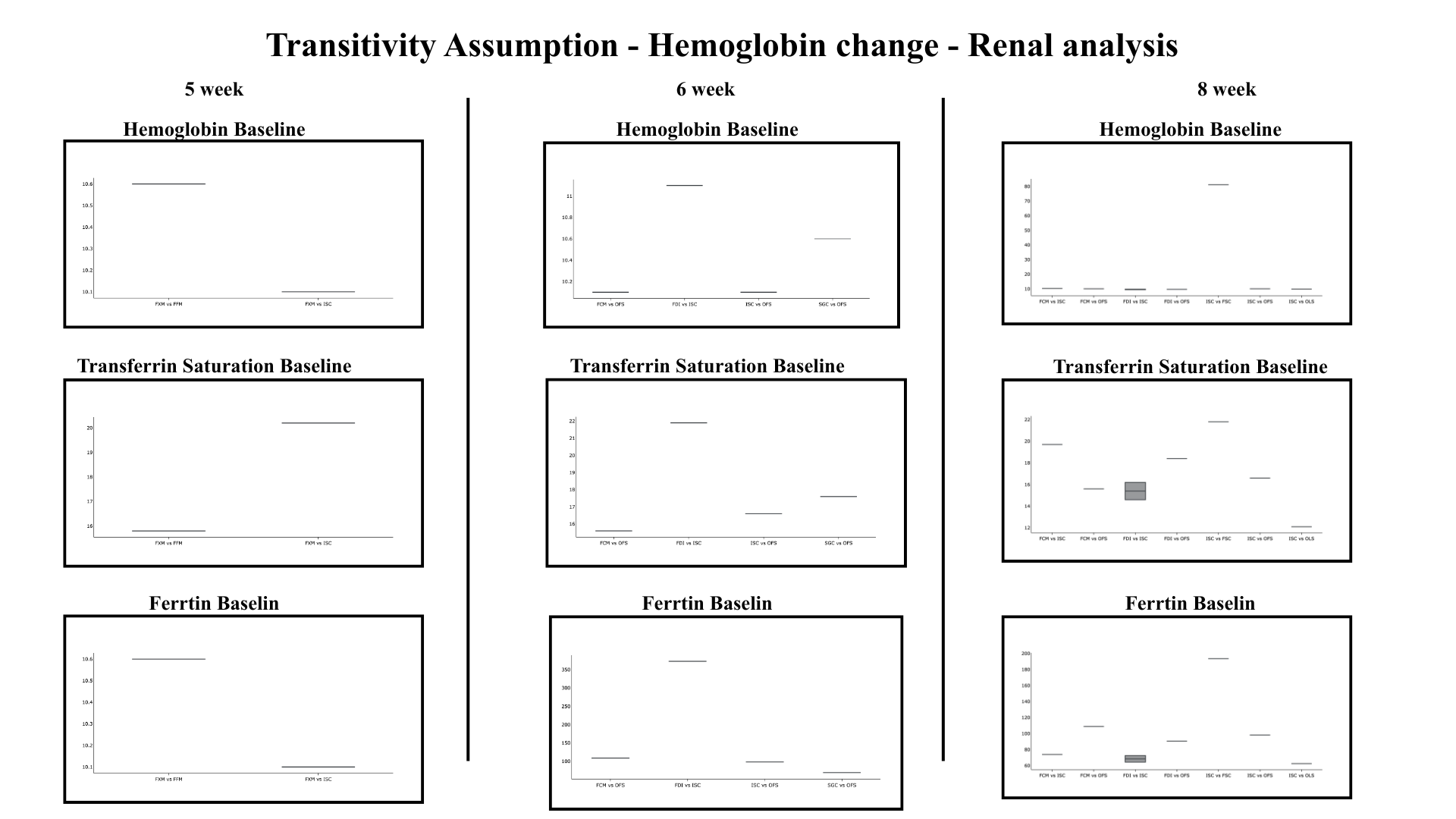


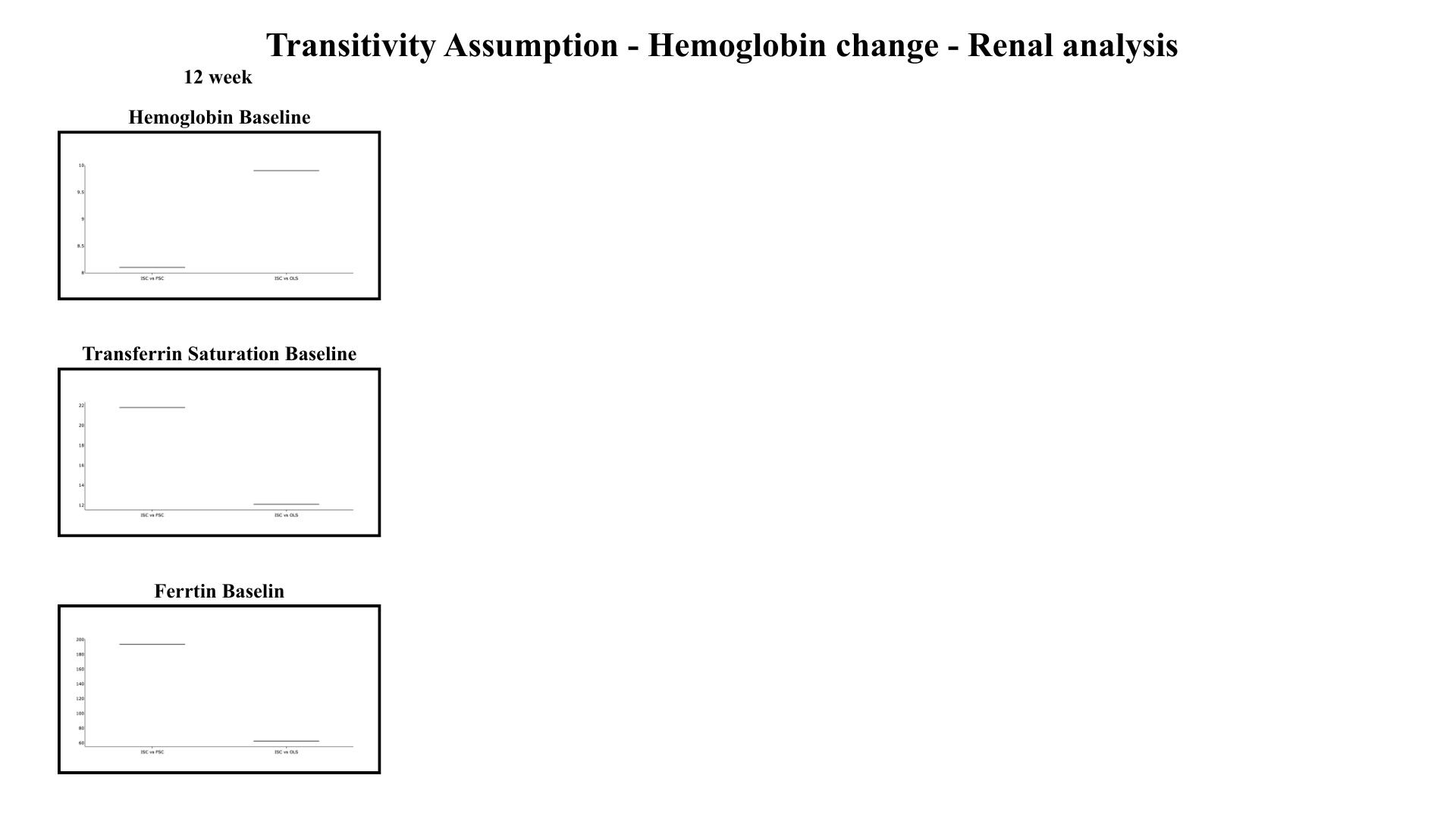


S10.4 – Transferrin saturation change - Overall analysis.


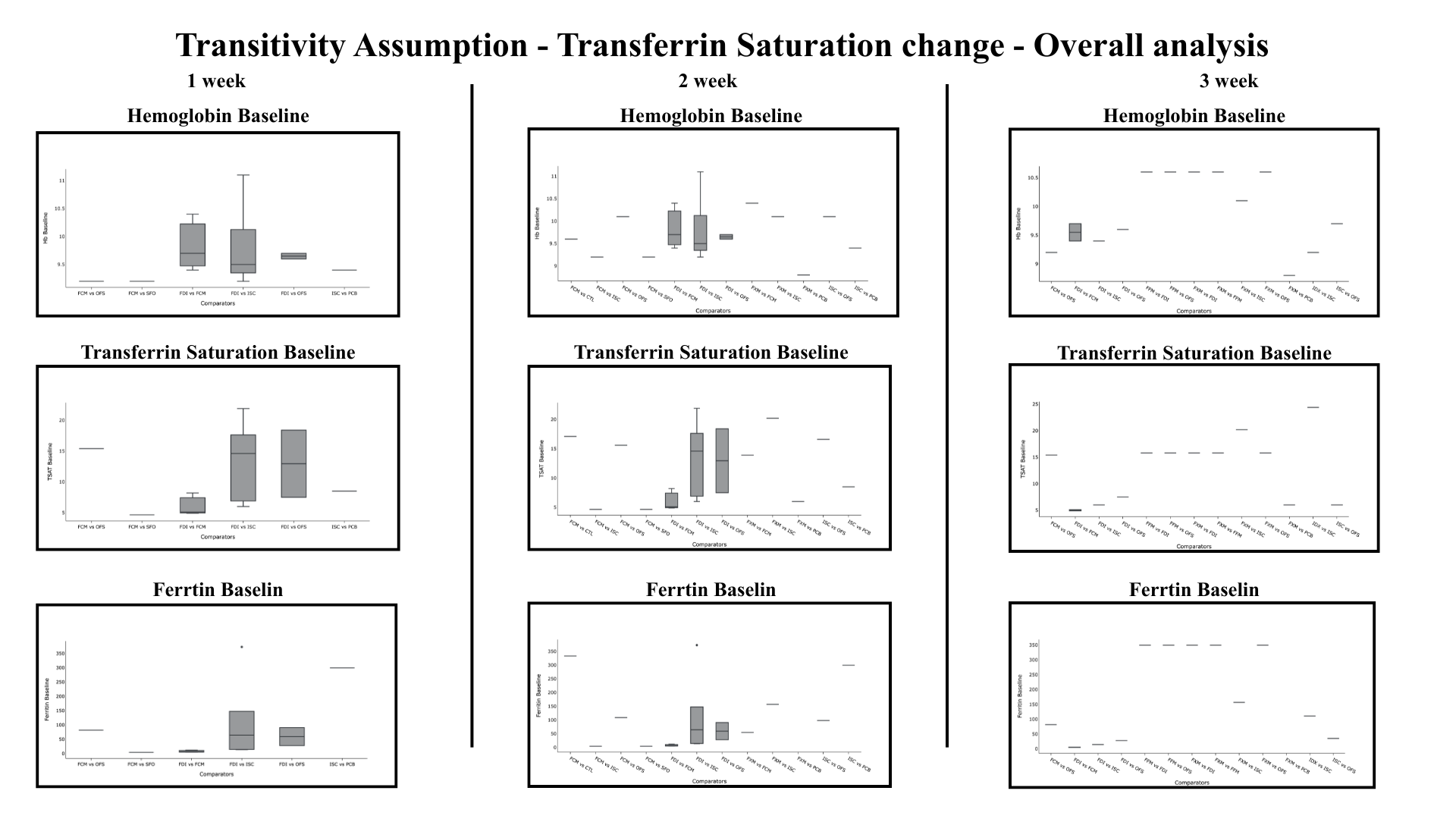


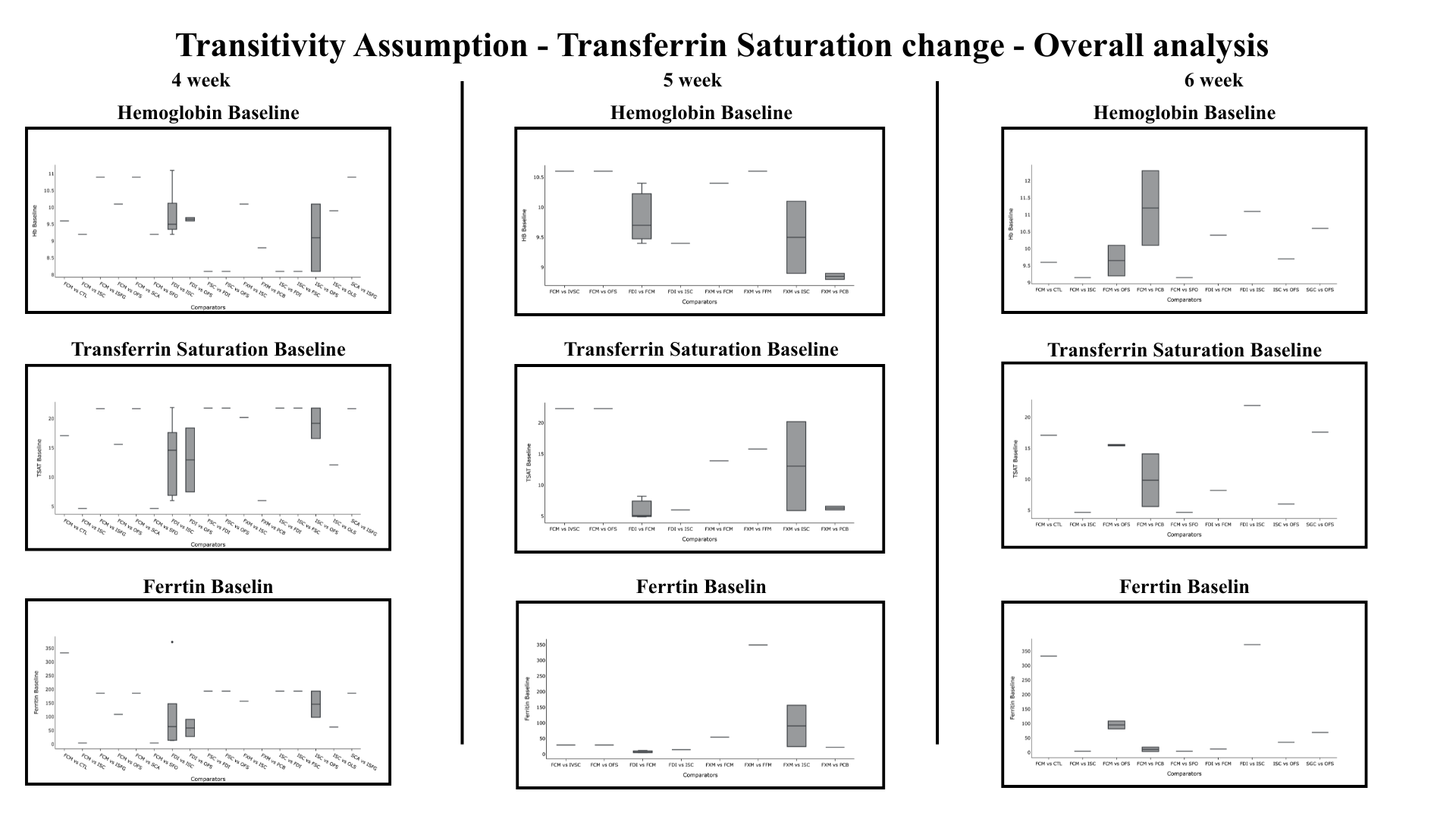


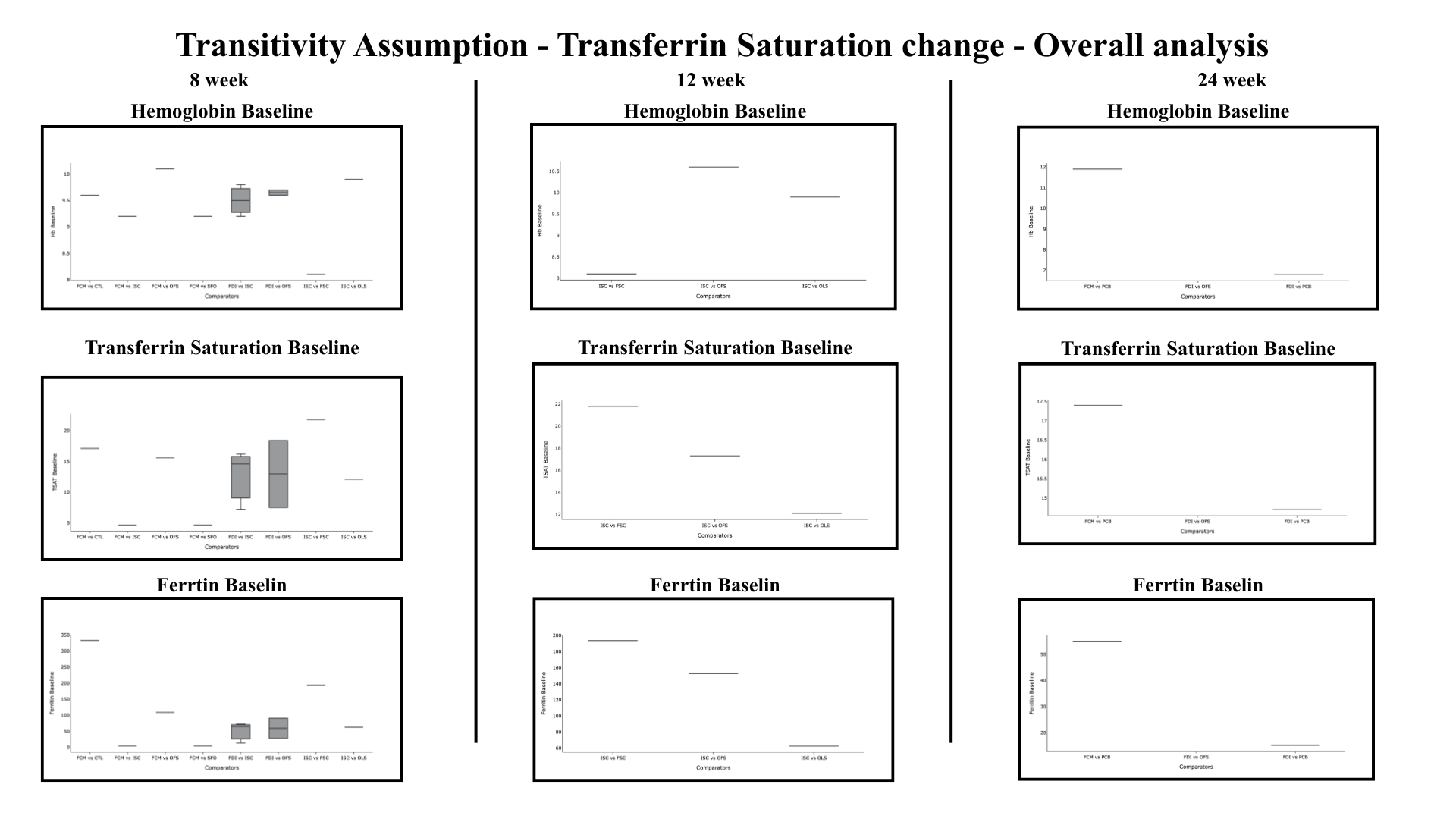


S10.5 – Transferrin saturation change - GI analysis.


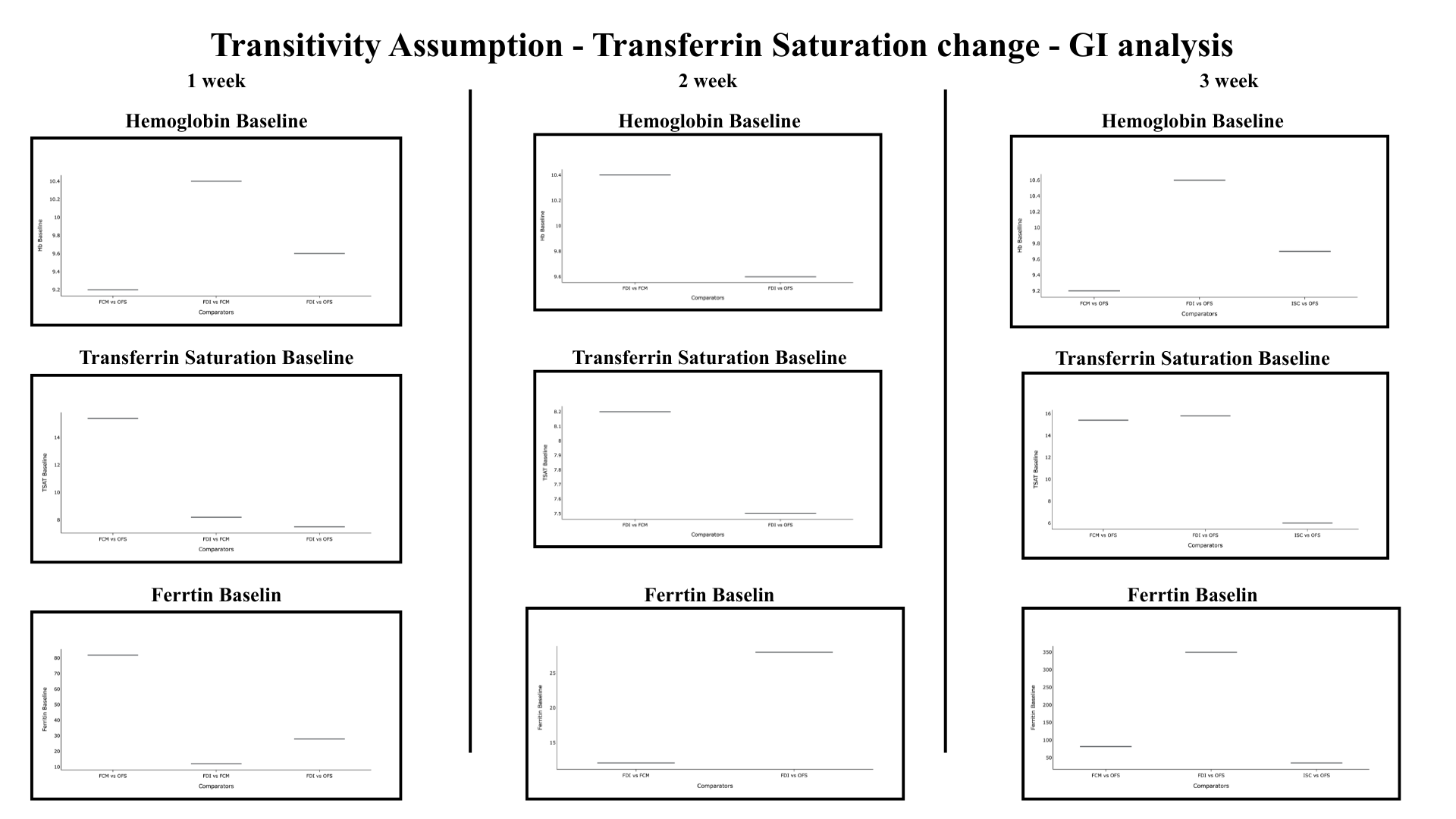


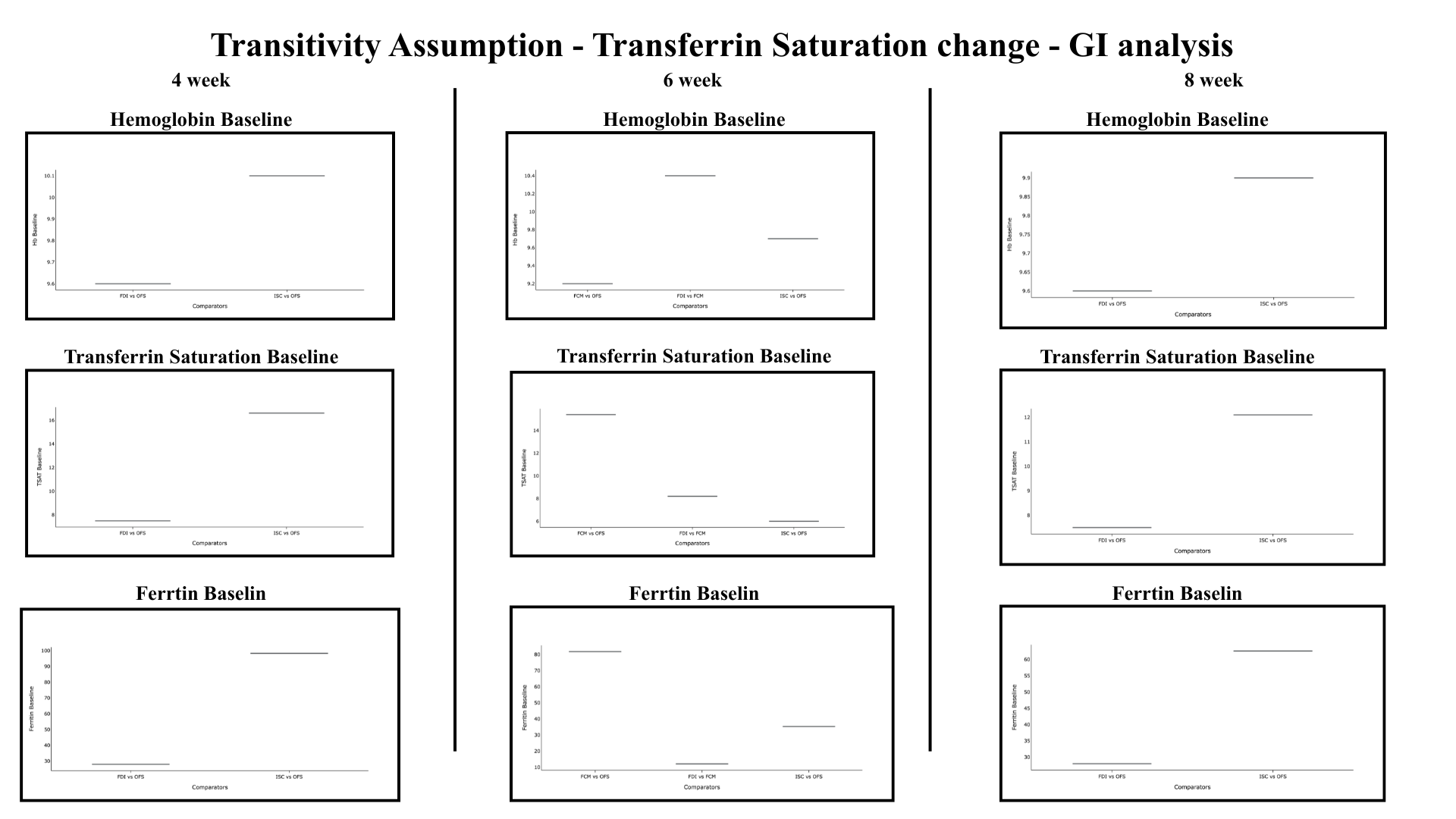


S10.6 – Transferrin saturation change - Renal analysis.


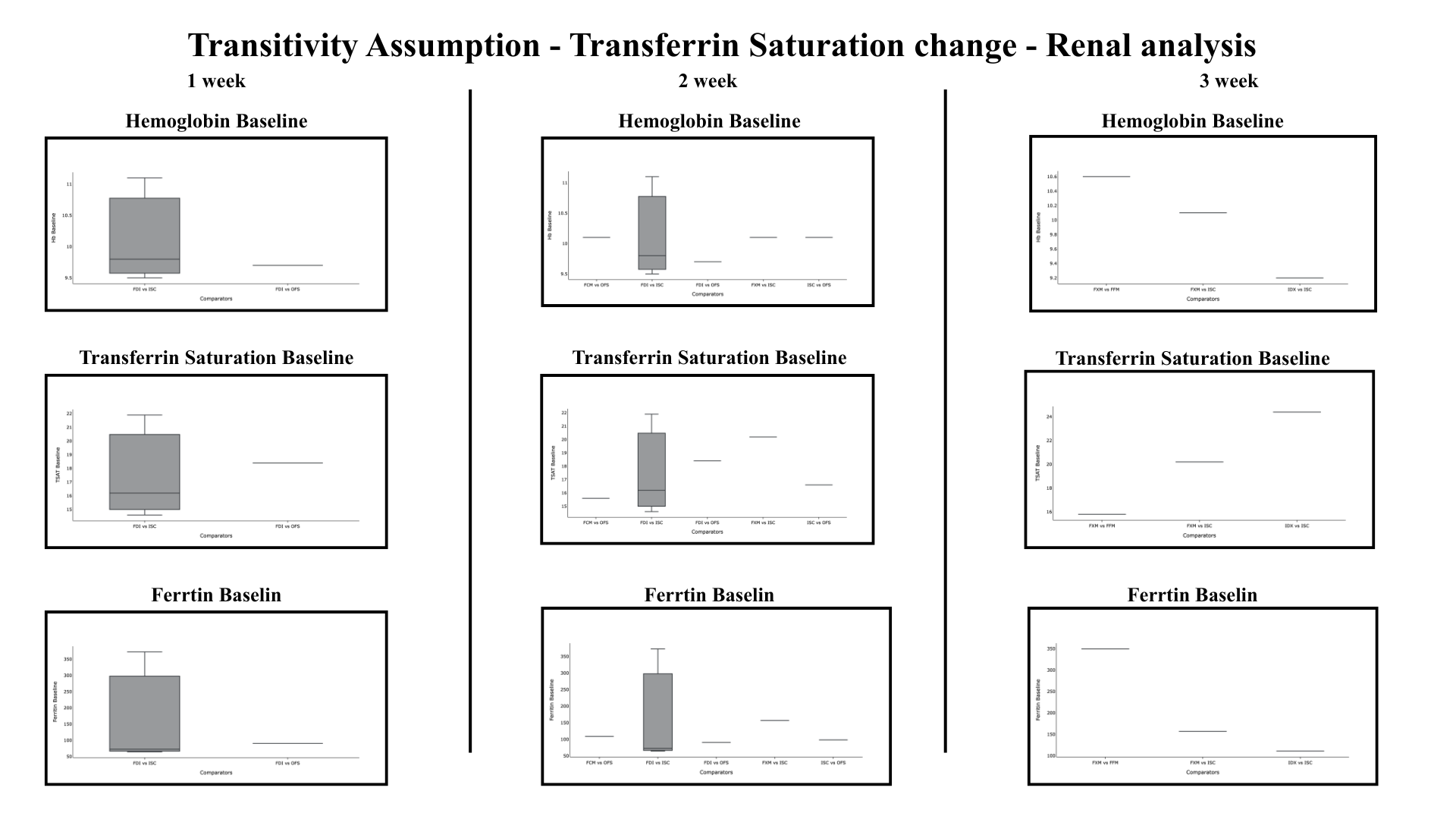


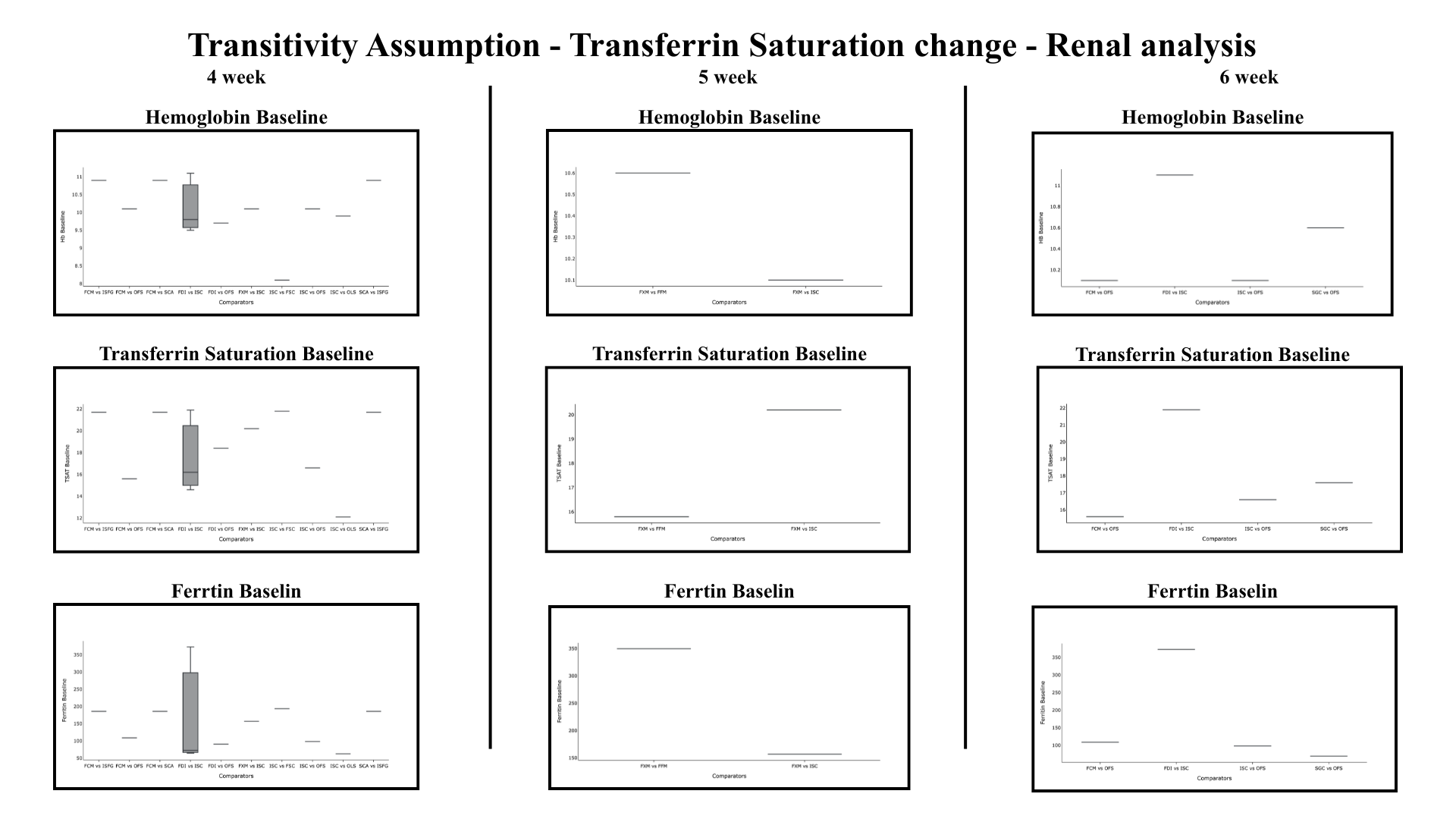


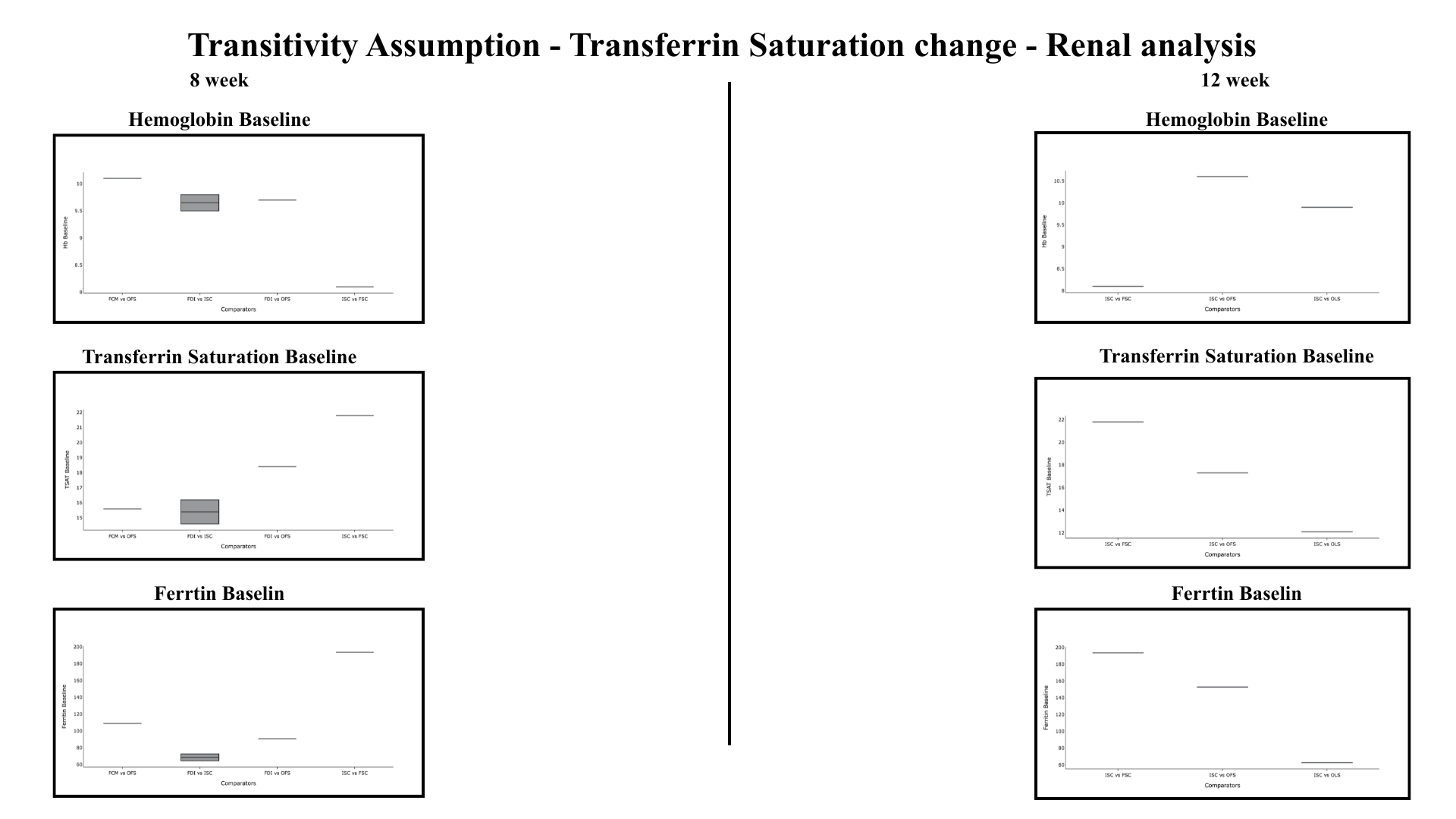


S10.7 – Ferritin change - Overall analysis.


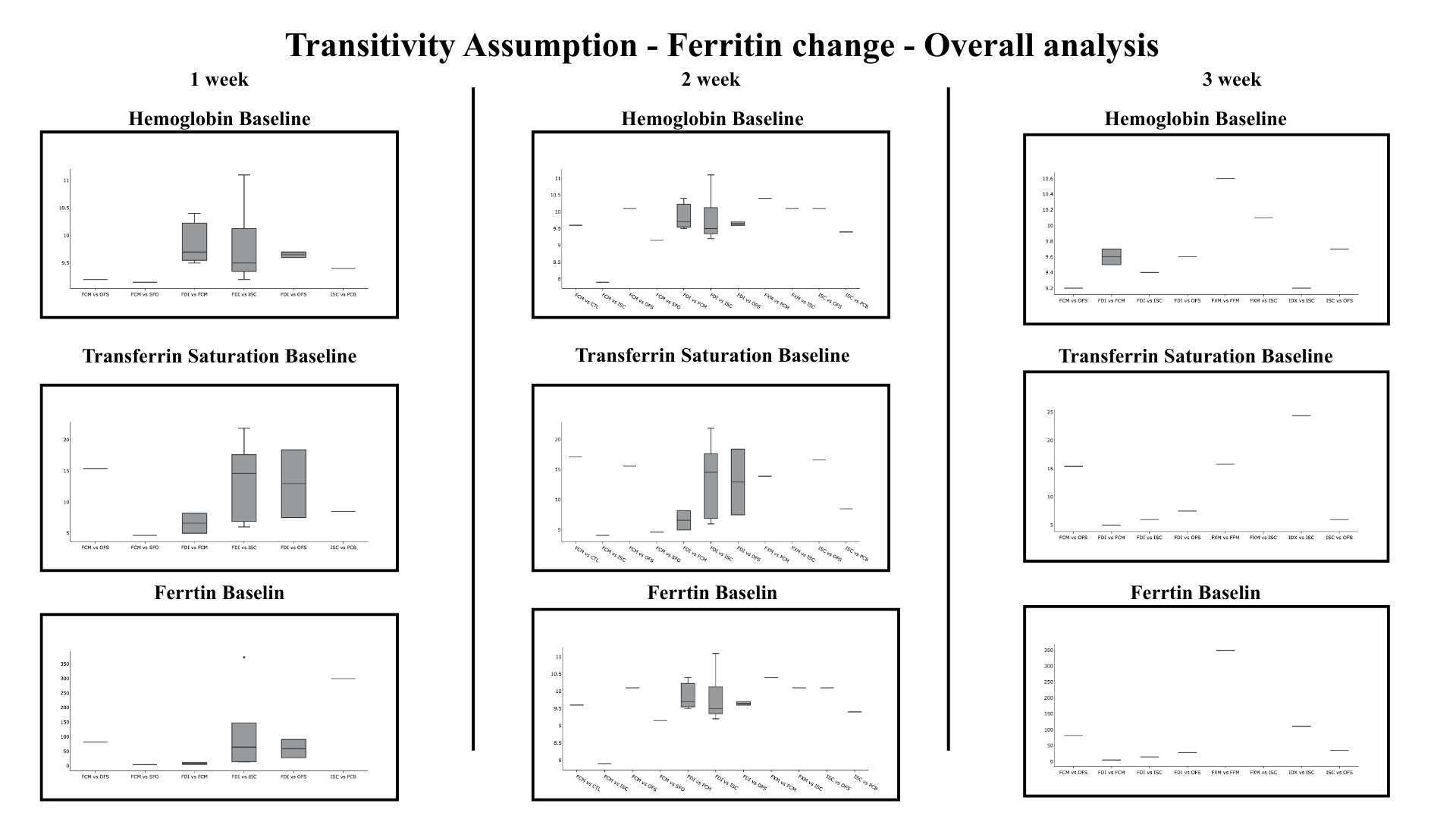


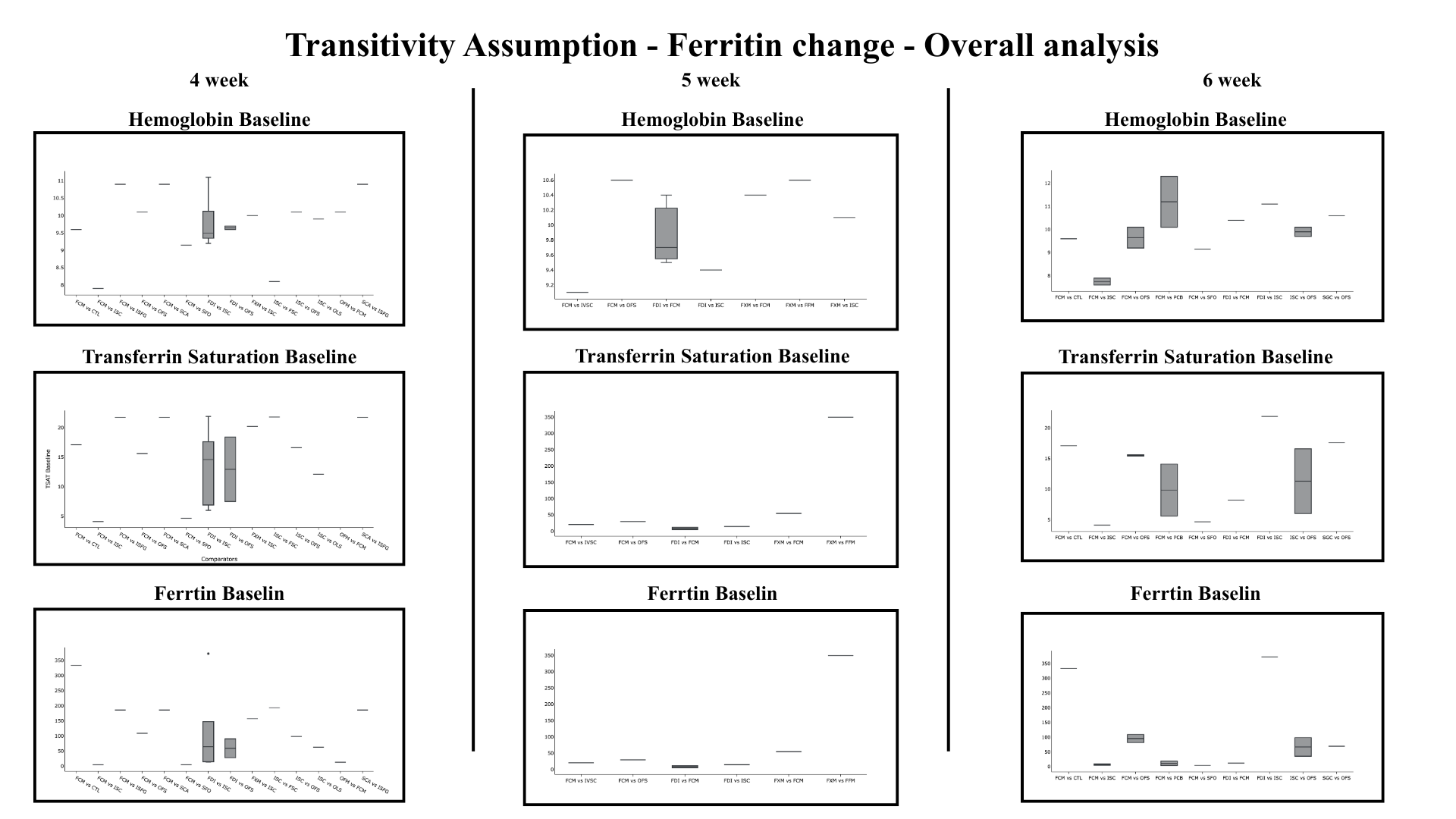


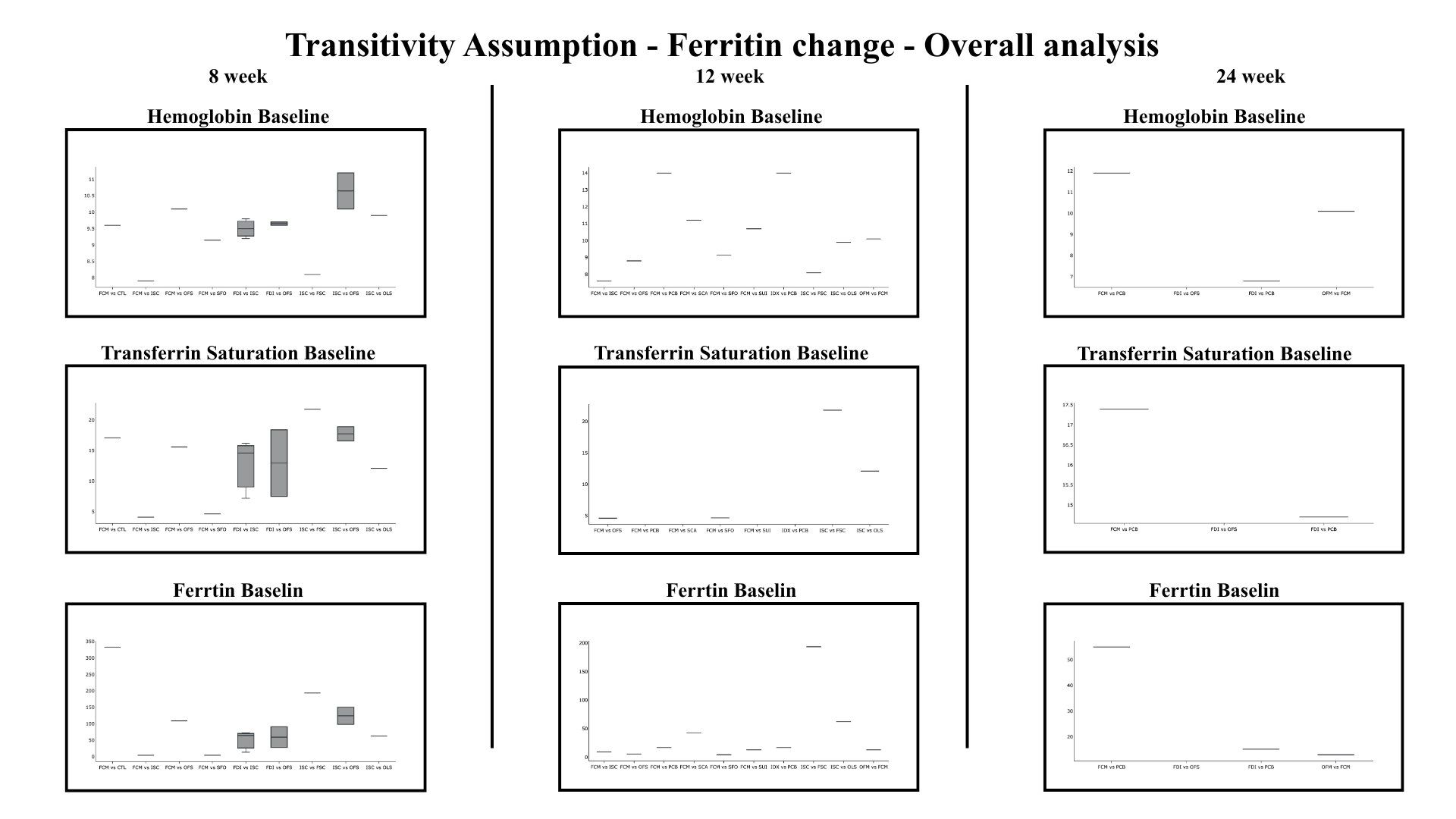


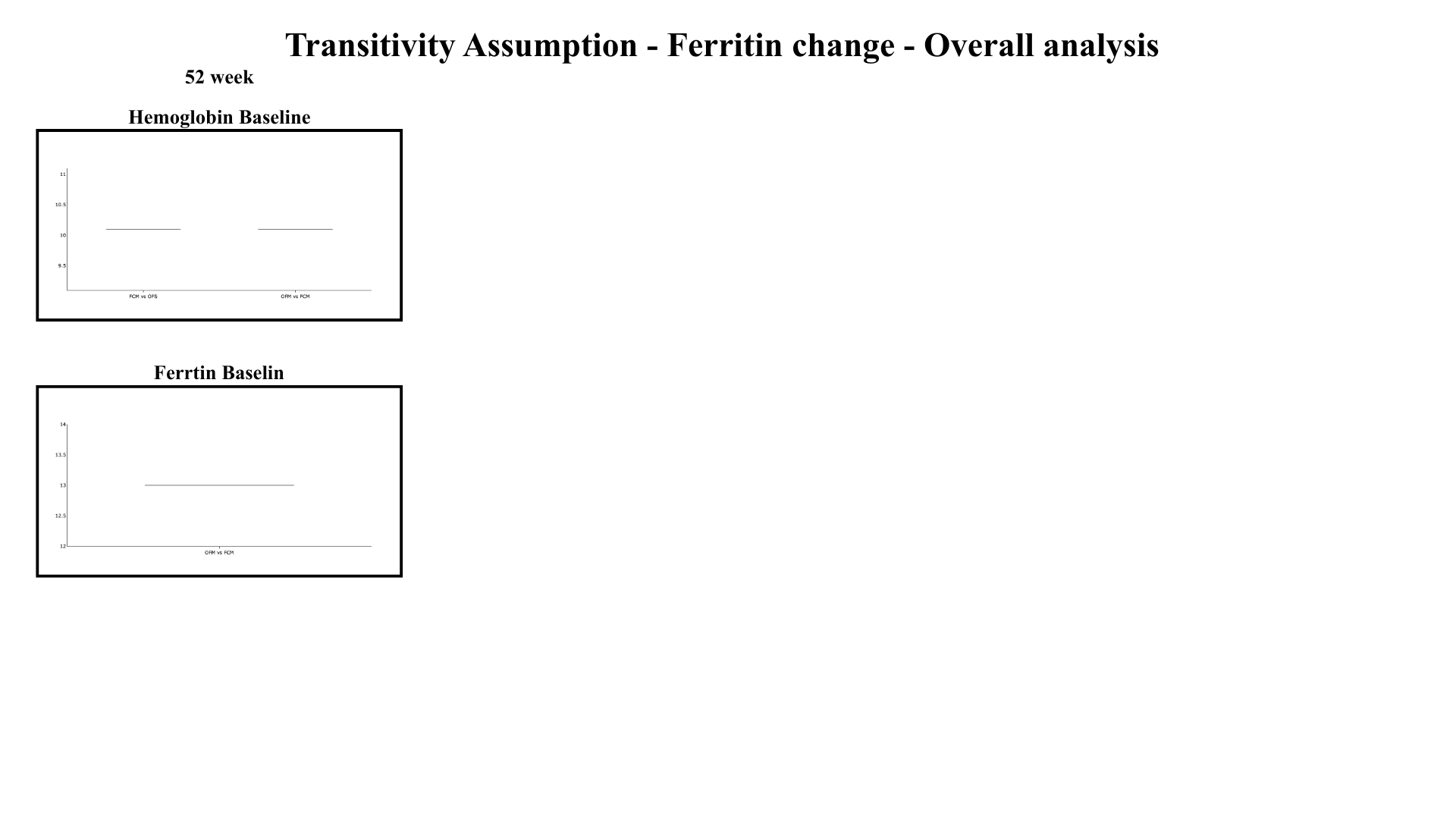


S10.8 – Ferritin change GI analysis.


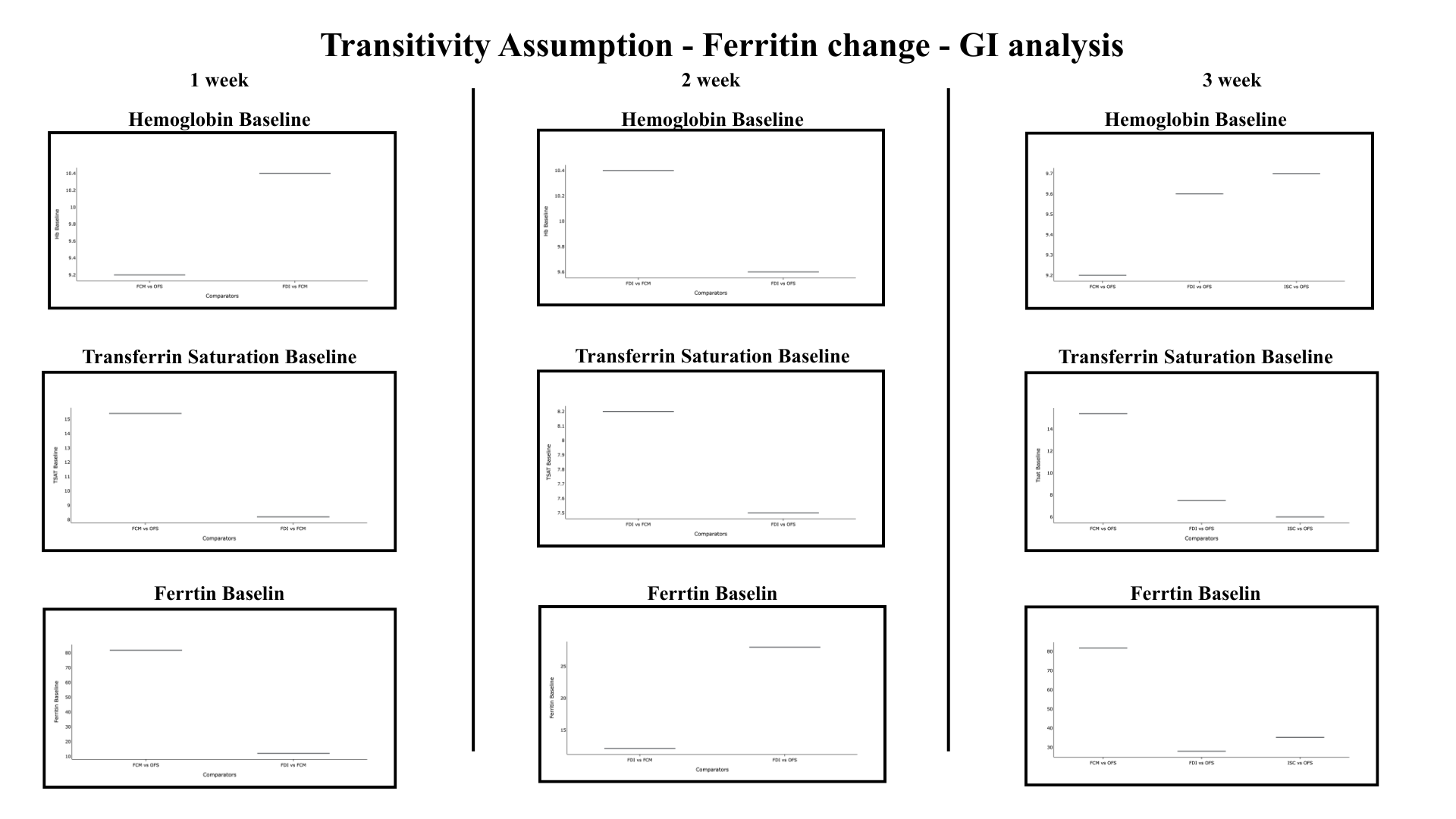


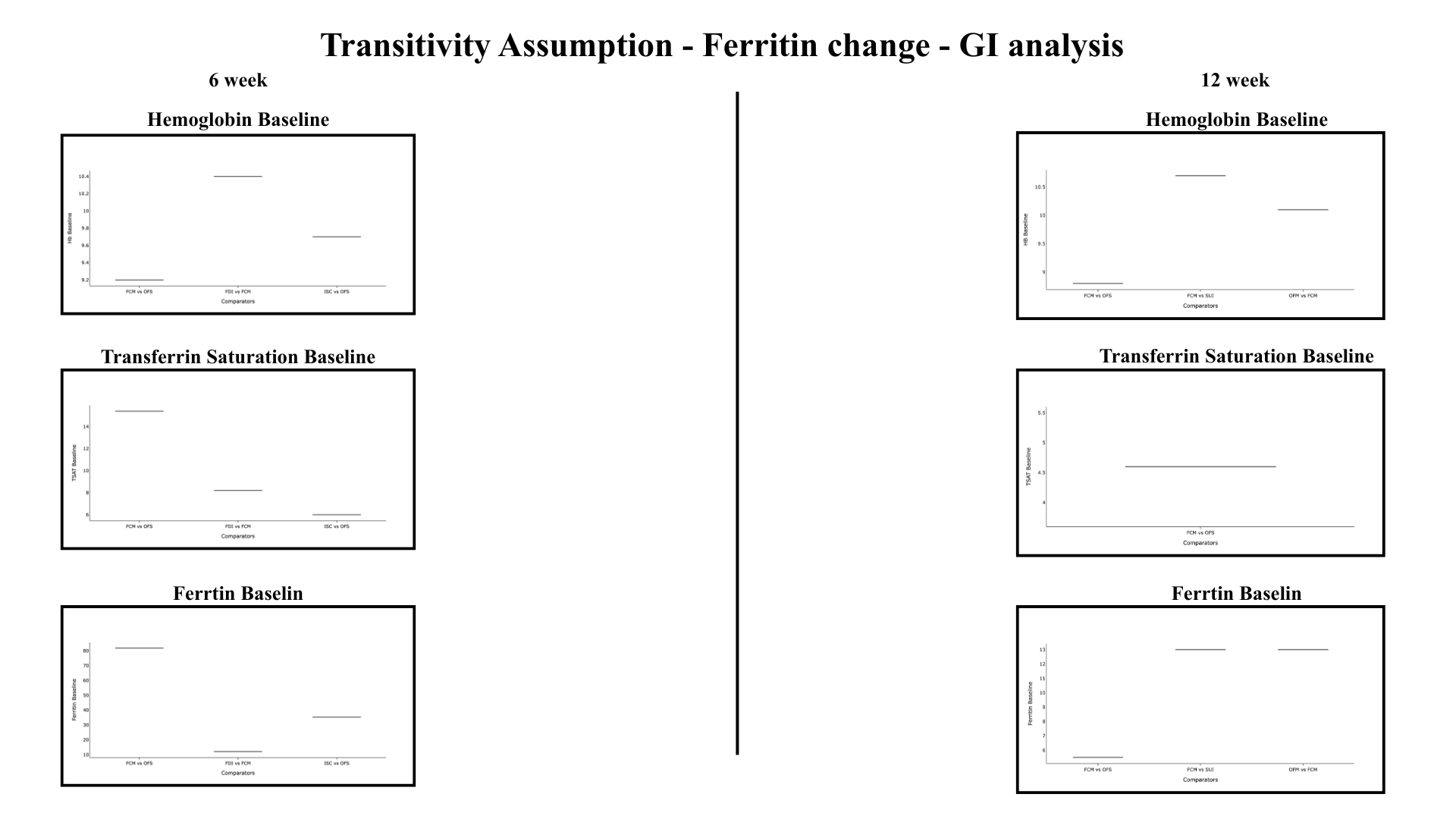


S10.9 – Ferritin change Renal analysis.


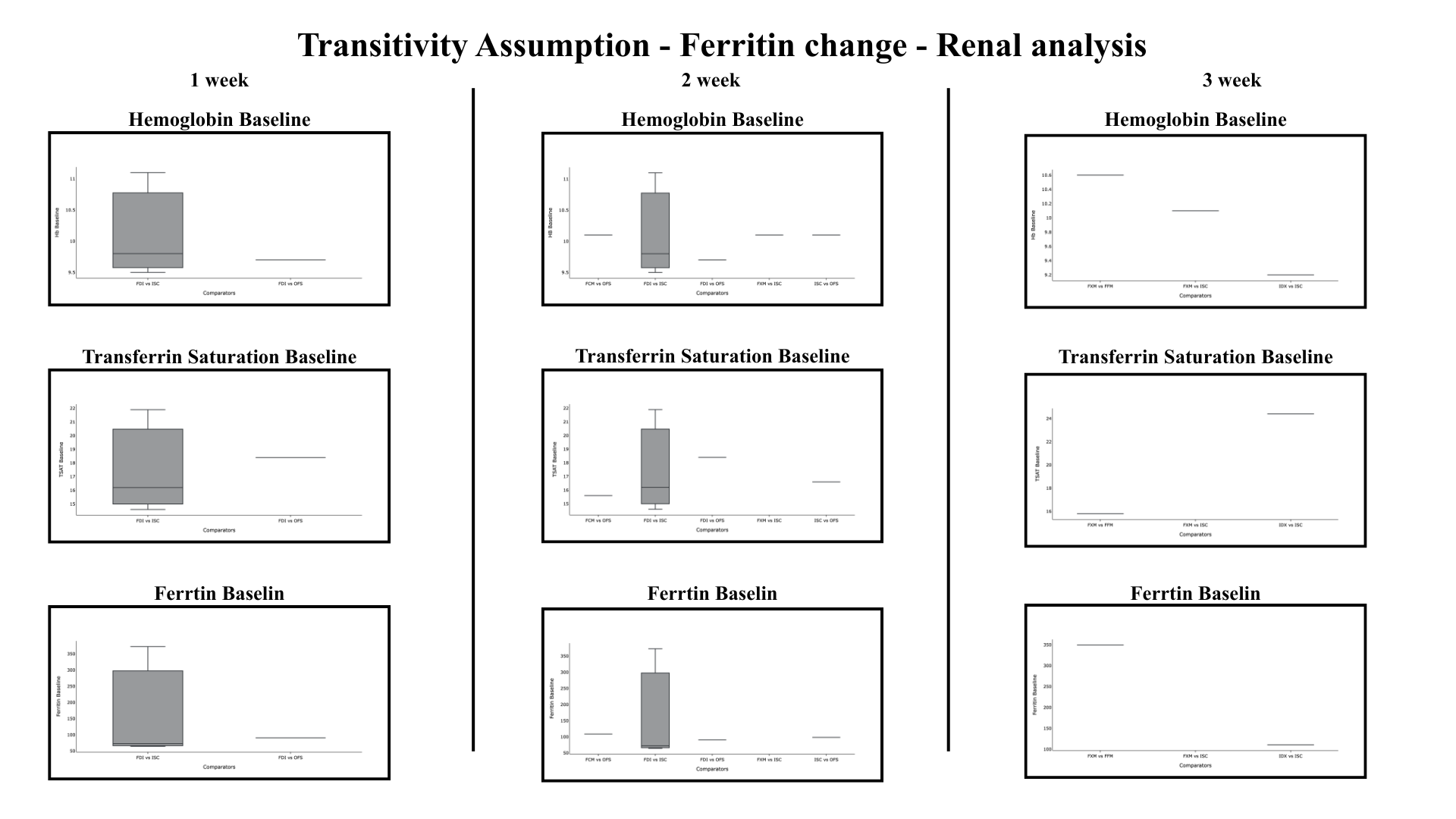


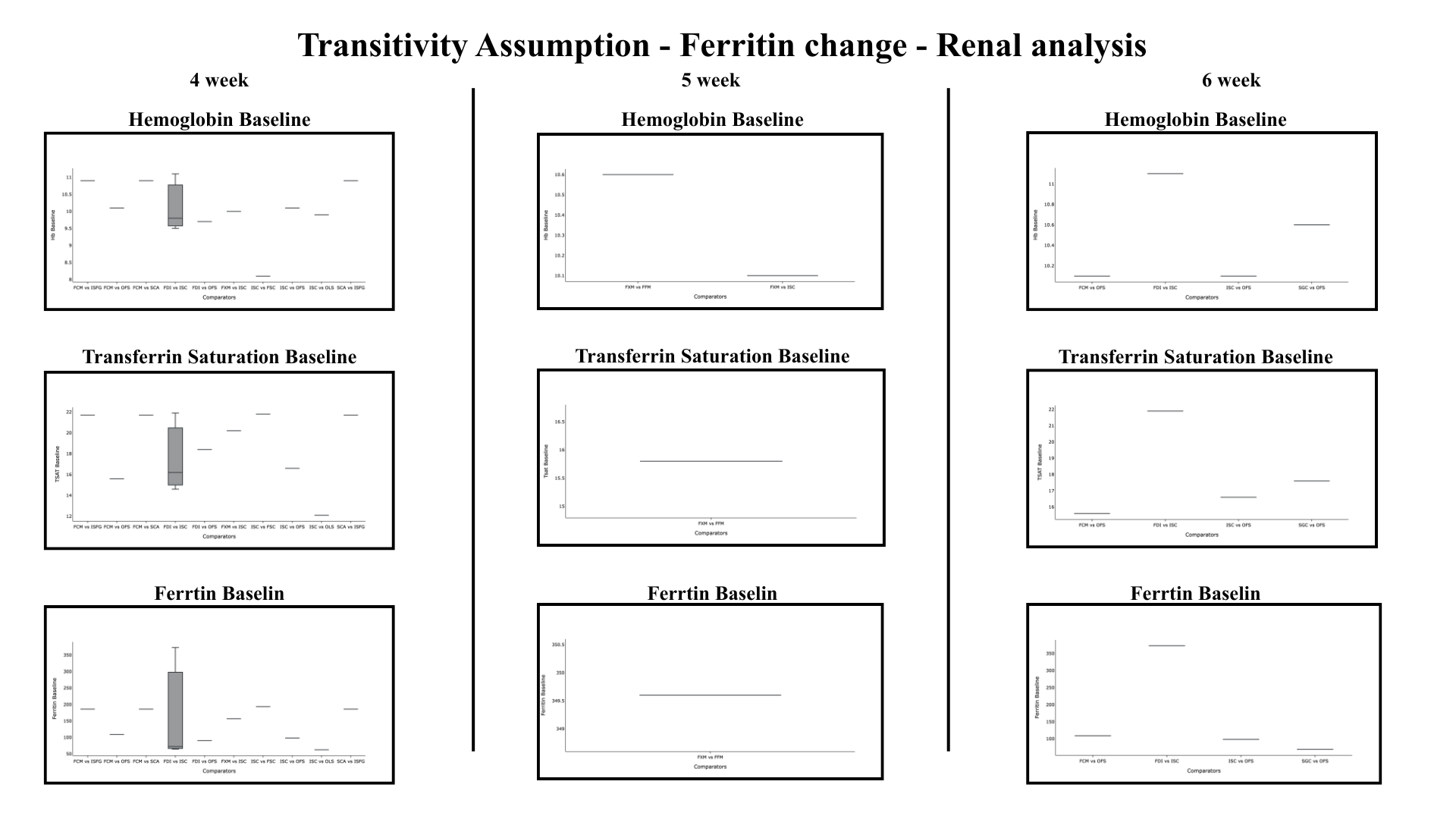


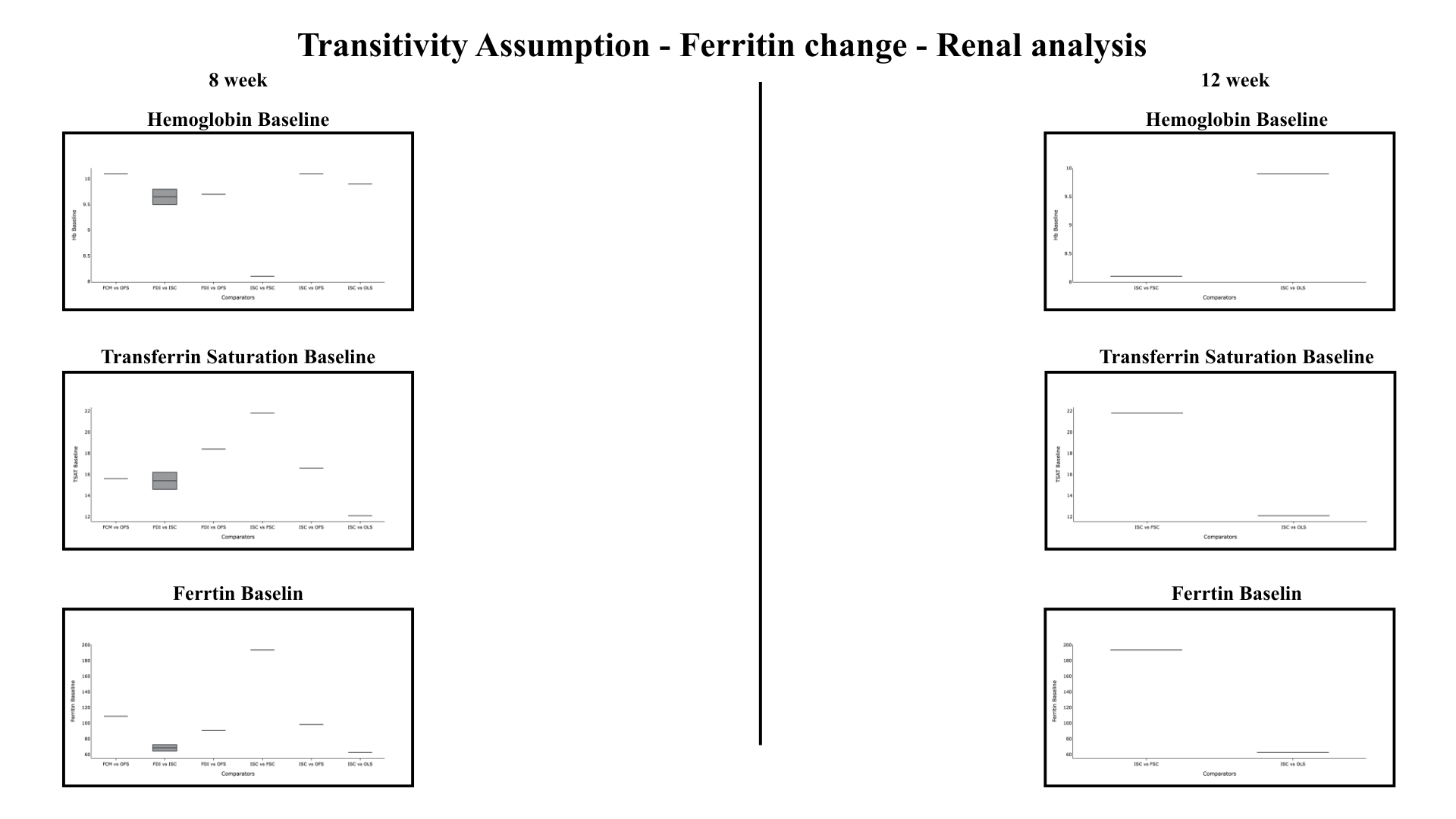


S10.10 – Any Adverse Events - Overall analysis.


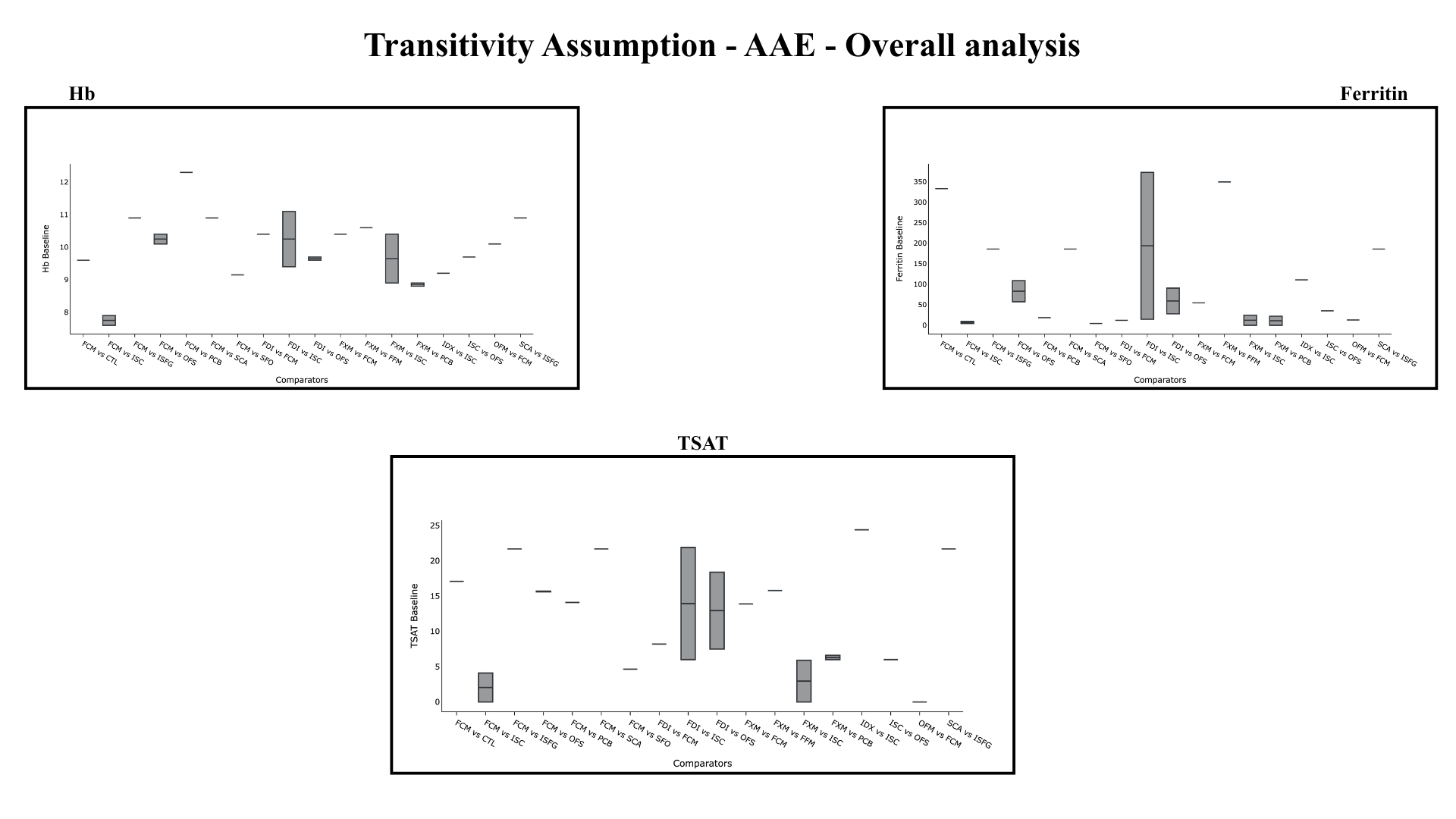


S10.11 – Any Adverse Events – GI analysis.


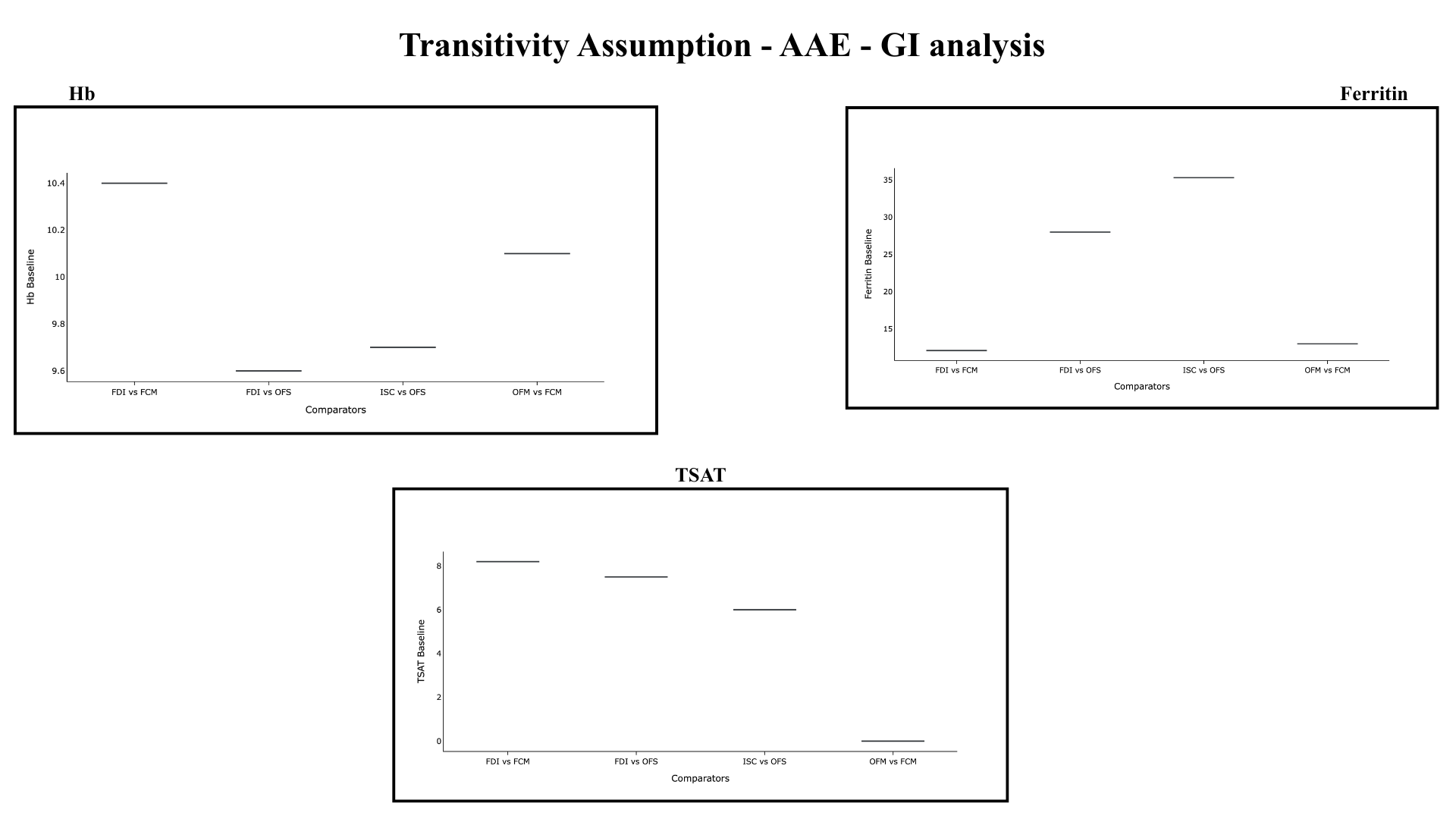


S10.12 – Any Adverse Events - Renal analysis.


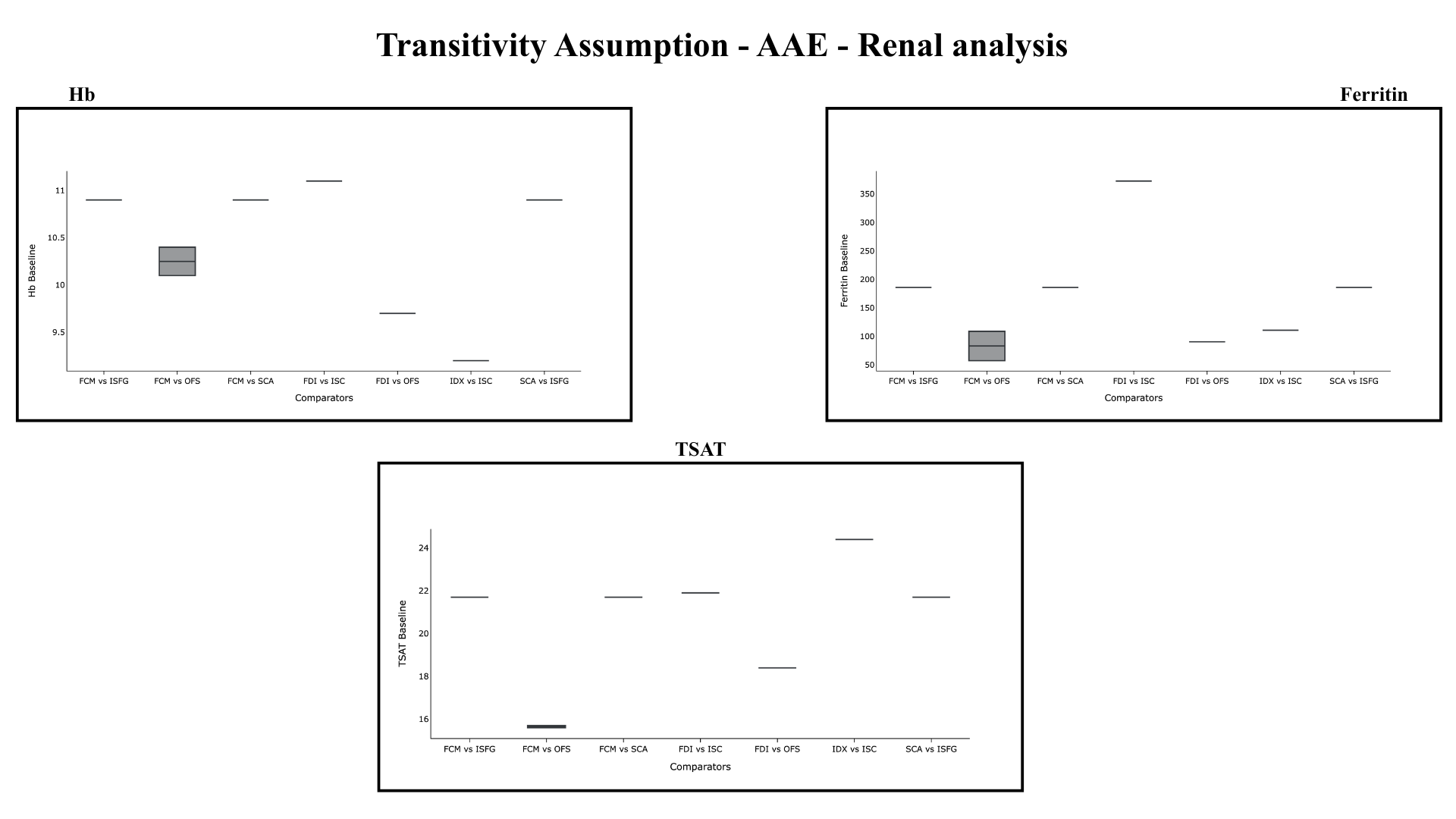


S10.13 – Serious Adverse Events – Overall analysis.


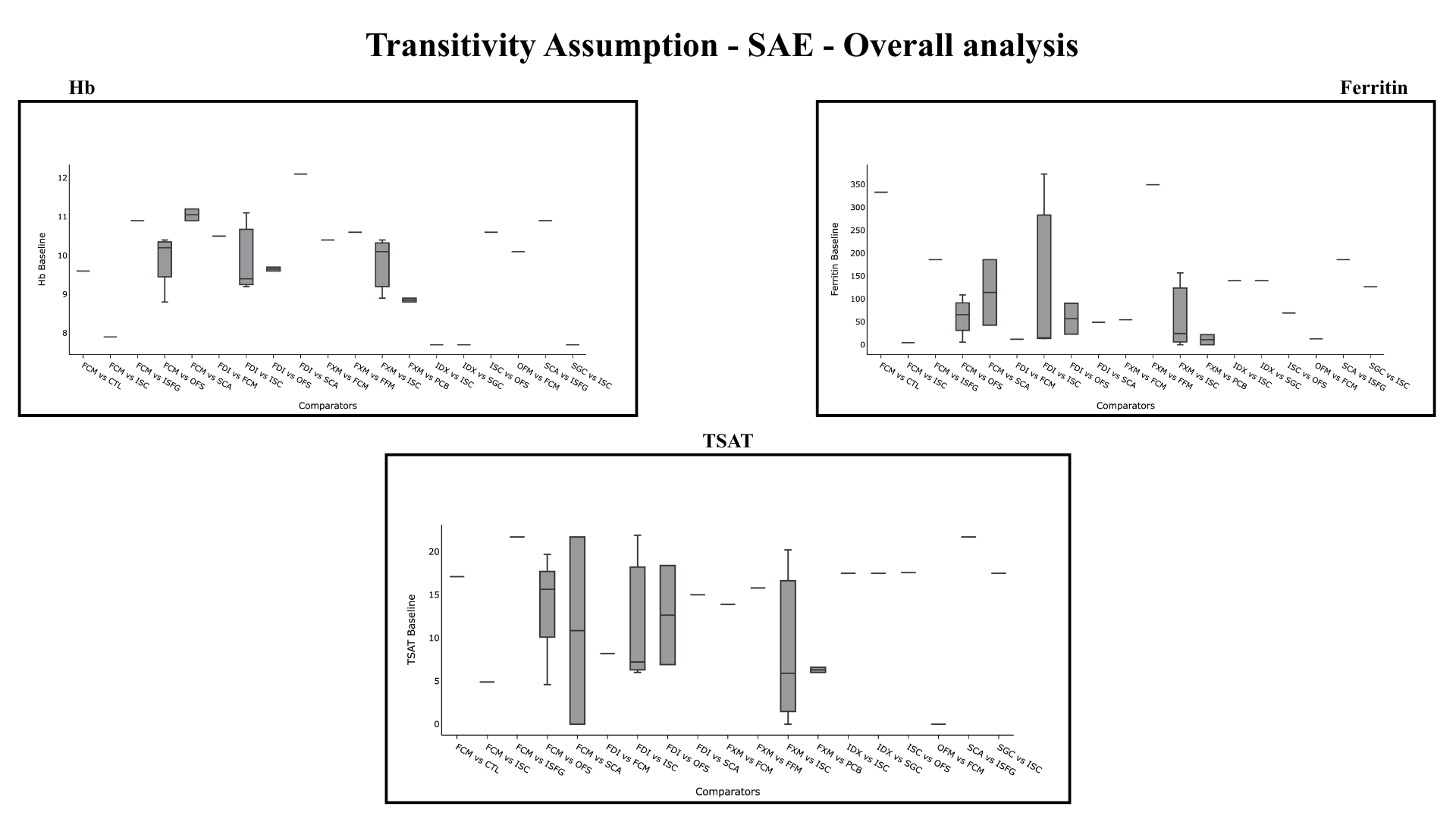


S10.14 – Serious Adverse Events – GI analysis.


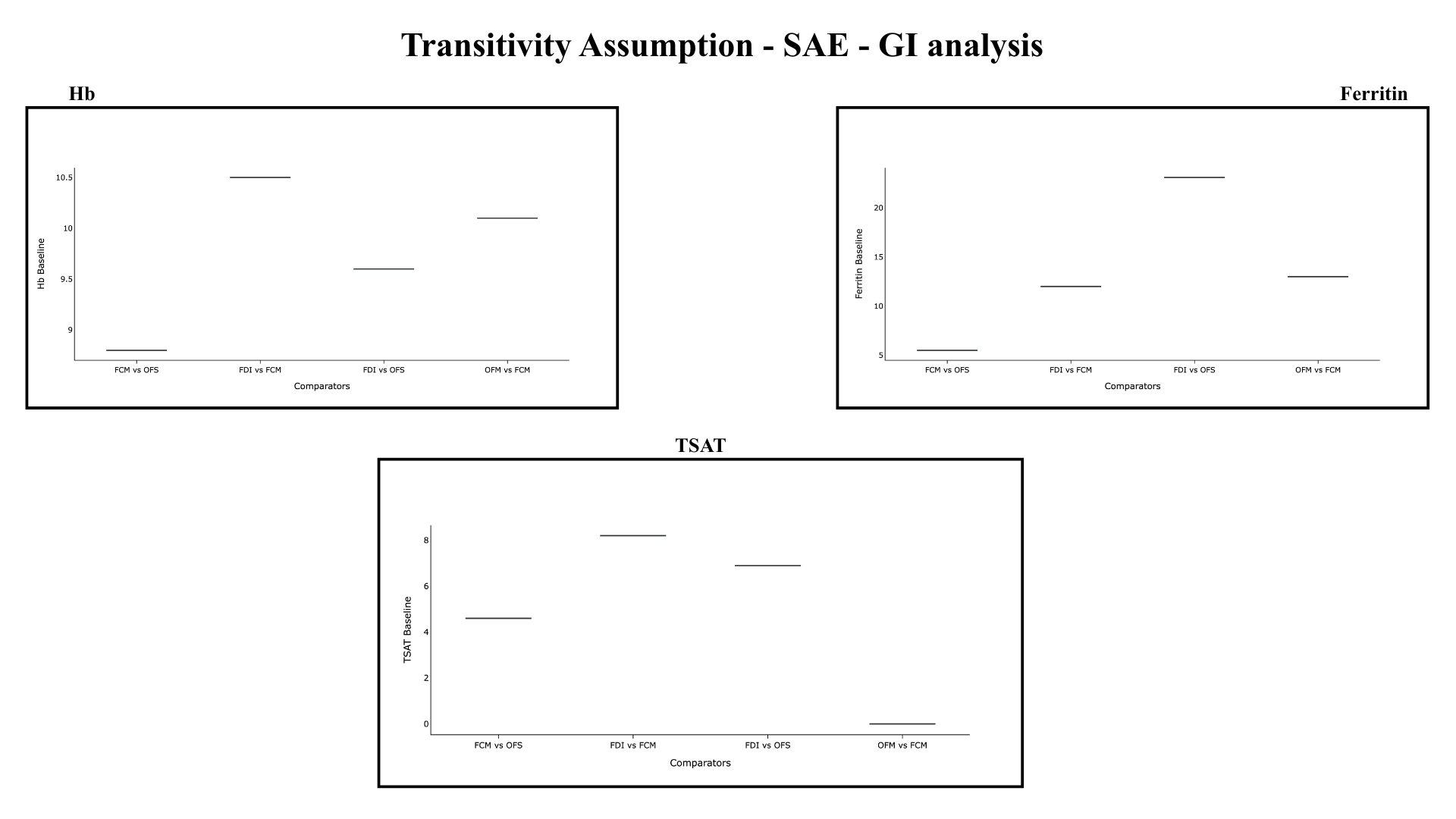


S10.15 – Serious Adverse Events – Renal analysis.


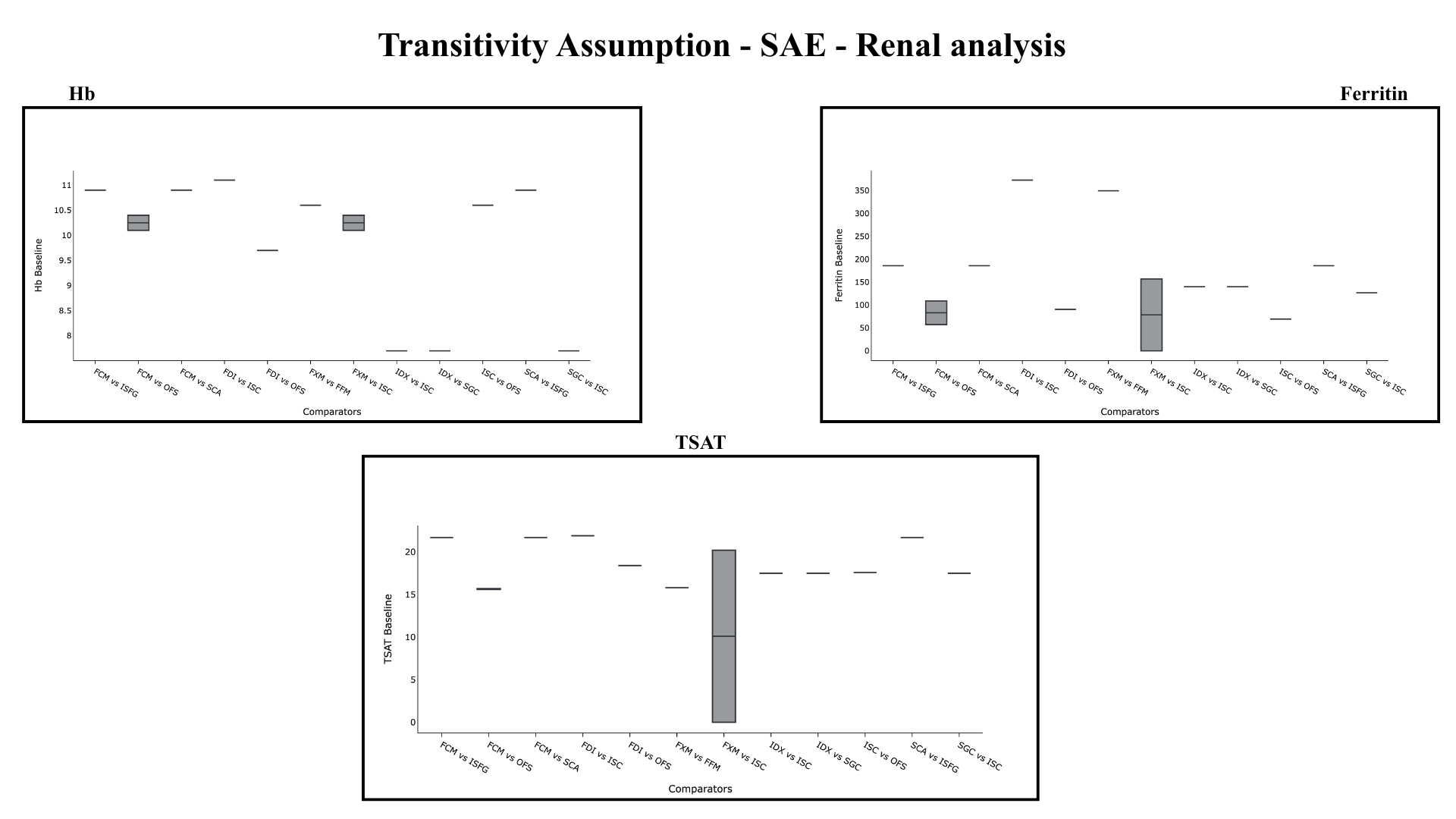


S10.16 – Hypophosphatemia – Overall analysis.


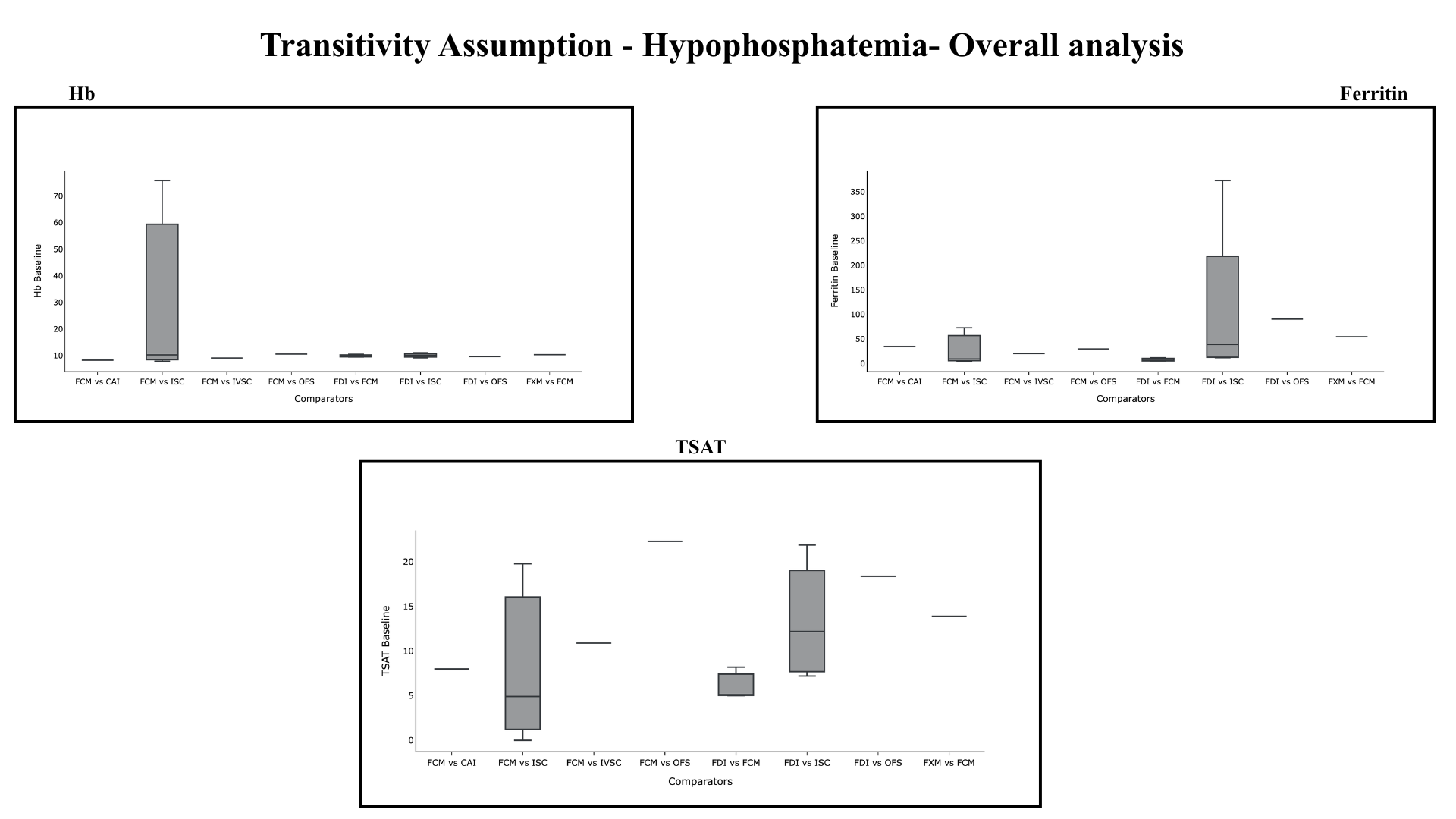


S10.1 – Hypophosphatemia – GI analysis.


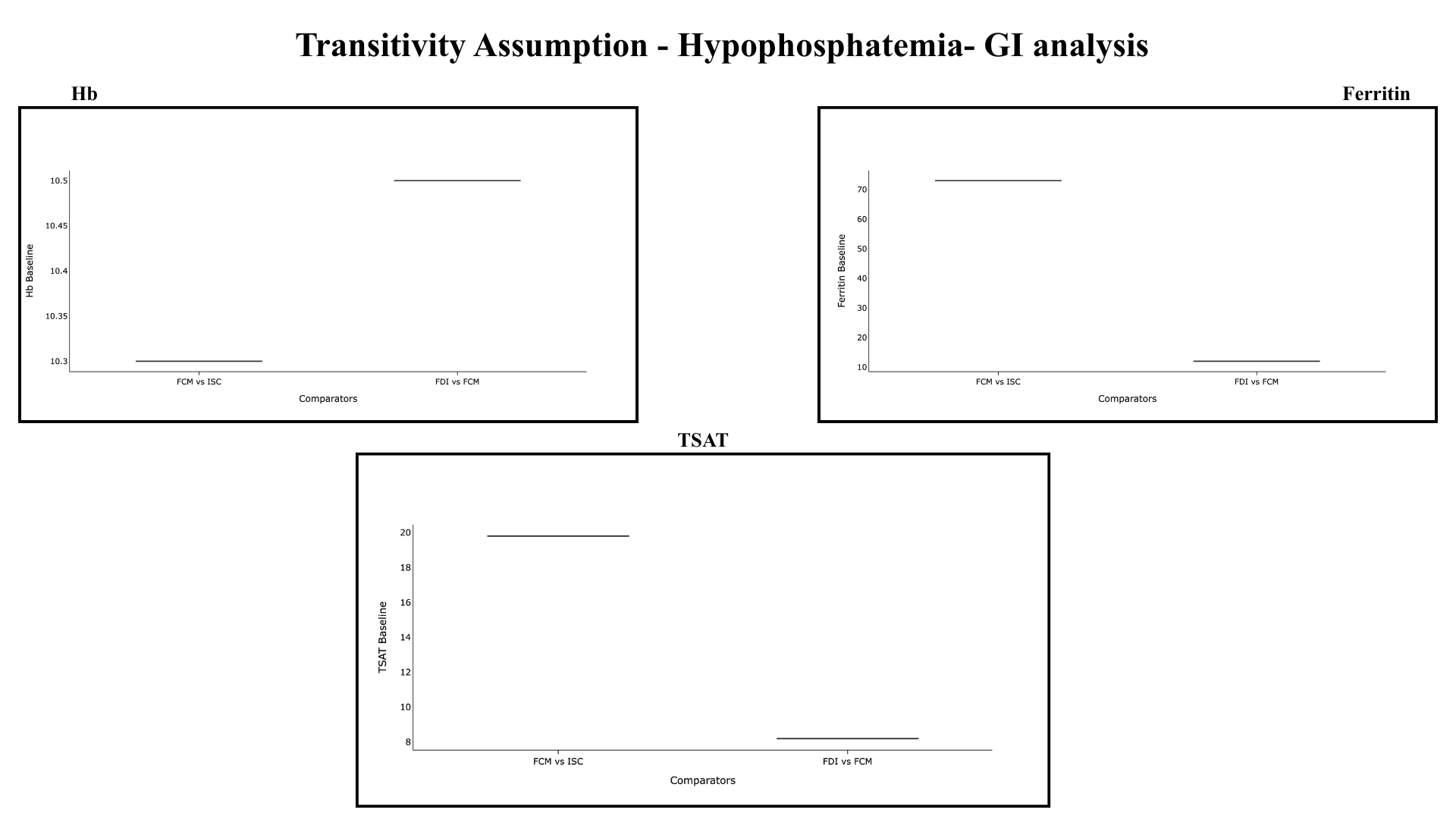


S10.16 – Hypophosphatemia – Renal analysis.


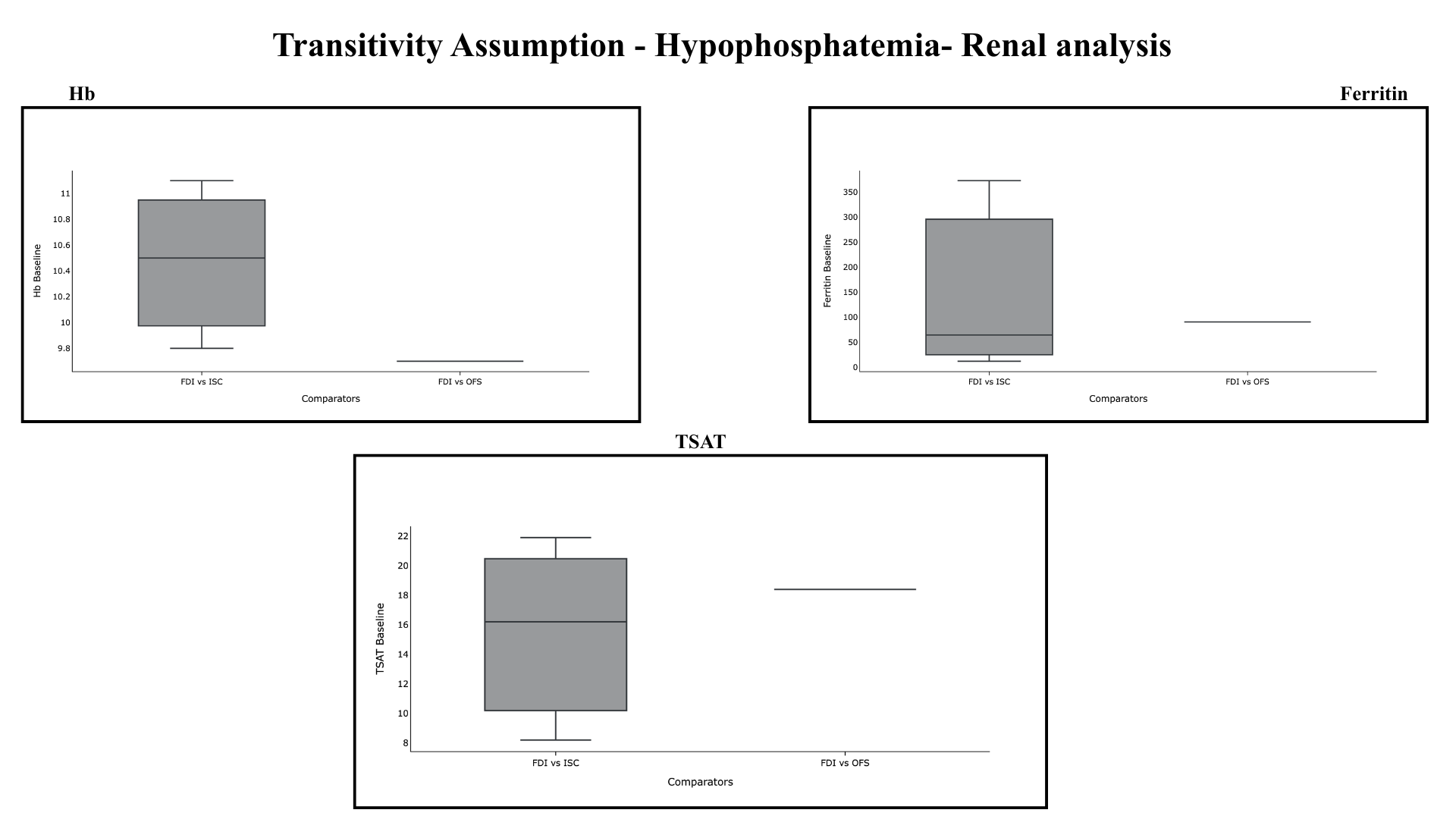


# Supplement 11 - Assessment of inconsistency

S11.1 – Hemoglobin

| **1 week** | | | |
| --- | --- | --- | --- |
| **Comparison** | **direct** | **indirect** | **p-value** |
| FCM vs FDI | 1 | 1.411 | 0.9172 |
| FCM vs ISC | 1.8221 | 1.291 | 0.9172 |
| **2 week** | | | |
| FCM vs FDI | 1.2438 | 0.678 | 0.0587 |
| FCM vs ISC | 0.5712 | 1.644 | 0.0043* |
| FCM vs OFS | 1.2214 | 0.916 | 0.4693 |
| FDI vs ISC | 1.3375 | 0.465 | 0.0043* |
| FDI vs OFS | 1.0268 | 1.369 | 0.4693 |
| **3 week** | | | |
| FCM vs FDI | 1.8193 | 1.58 | 0.7412 |
| FCM vs OFS | 2.2255 | 2.562 | 0.7412 |
| FDI vs ISC | 2.2255 | 0.99 | 0.0424* |
| FDI vs OFS | 0.9048 | 1.744 | 0.0926 |
| ISC vs OFS | 1.0358 | 0.461 | 0.0424* |
| **4 week** | | | |
| FCM vs ISC | 0.6703 | 1.911 | 0.1109 |
| FCM vs OFS | 1.4918 | 0.523 | 0.1109 |
| FDI vs ISC | 1.3884 | 0.487 | 0.1109 |
| FDI vs OFS | 1.0841 | 3.09 | 0.1109 |
| **5 week** | | | |
| FDI vs FCM | 0.6164 | 1.052 | 0.0911 |
| FDI vs ISC | 1.4918 | 0.874 | 0.0911 |
| FXM vs FCM | 0.8187 | 0.48 | 0.0911 |
| FXM vs ISC | 1.1613 | 1.982 | 0.0911 |
| **6 week** | | | |
| FCM vs FDI | 1.1052 | 1.186 | 0.8997 |
| FCM vs ISC | 0.7464 | 1.617 | 0.0574 |
| FCM vs OFS | 1.8844 | 0.846 | 0.0477* |
| FDI vs ISC | 1 | 1.073 | 0.8997 |
| ISC vs OFS | 1.021 | 2.274 | 0.0477* |
| **8 week** | | | |
| FCM vs ISC | 1.0613 | 2.156 | 0.2444 |
| FCM vs OFS | 1.3499 | 0.664 | 0.2444 |
| FDI vs ISC | 1.6506 | 1.121 | 0.4582 |
| FDI vs OFS | 0.9805 | 1.444 | 0.4582 |
| ISC vs OFS | 0.6703 | 0.777 | 0.7876 |

**S11.2 – TSAT**

| **1 week** | | | |
| --- | --- | --- | --- |
| **Comparison** | **direct** | **indirect** | **p-value** |
| FCM vs FDI | 0.02 (0, 0) | 117.34 (0.06, 0.06) | 0.058 |
| FCM vs OFS | 1998.2 (10.44, 10.44) | 0.31 (0, 0) | 0.058 |
| FDI vs OFS | 17.03 (0.07, 0.07) | 110482.98 (78.66, 78.66) | 0.058 |
| **2 week** | | | |
| FCM vs FDI | 215.27 (3.74, 3.74) | 254.89 (1.82, 1.82) | 0.959 |
| FCM vs FXM | 9.97 (0.06, 0.06) | 1.94 (0, 0) | 0.712 |
| FCM vs ISC | 221.41 (0.06, 0.06) | 6315.37 (145.84, 145.84) | 0.468 |
| FCM vs OFS | 2440.6 (3.53, 3.53) | 488.24 (3.06, 3.06) | 0.703 |
| FDI vs ISC | 11.65 (0.75, 0.75) | 42.47 (0.21, 0.21) | 0.671 |
| FDI vs OFS | 8.05 (0.07, 0.07) | 1.61 (0.01, 0.01) | 0.655 |
| FXM vs ISC | 81.45 (0.08, 0.08) | 2307.12 (8.98, 8.98) | 0.461 |
| FXM vs PCB | 4876800.85 (215.49, 215.49) | 457.02 (0.57, 0.57) | 0.131 |
| ISC vs OFS | 0.02 (0, 0) | 0.82 (0.01, 0.01) | 0.380 |
| ISC vs PCB | 3 (0.03, 0.03) | 32057.41 (0.49, 0.49) | 0.131 |
| **3 week** | | | |
| FCM vs FDI | 1225.02 (12.18, 12.18) | 1177.46 (1.89, 1.89) | 0.992 |
| FCM vs OFS | 1998.2 (35.8, 35.8) | 2078.91 (2.28, 2.28) | 0.992 |
| FFM vs FDI | 0 (0, 0) | 0 (0, 0) | 0.479 |
| FFM vs FXM | 0.01 (0, 0) | 0 (0, 0) | 0.364 |
| FFM vs OFS | 0 (0, 0) | 0.01 (0, 0) | 0.089 |
| FXM vs FDI | 0.02 (0, 0) | 0.03 (0, 0) | 0.880 |
| FXM vs ISC | 66.69 (0.19, 0.19) | 1.32 (0, 0) | 0.364 |
| FXM vs OFS | 0 (0, 0) | 0.77 (0, 0) | 0.082 |
| ISC vs FDI | 0 (0, 0) | 0.01 (0, 0) | 0.424 |
| ISC vs OFS | 0.02 (0, 0) | 0 (0, 0) | 0.110 |
| OFS vs FDI | 99.48 (0.06, 0.06) | 0.08 (0, 0) | 0.108 |
| **4 week** | | | |
| FCM vs ISC | 13.46 (0.01, 0.01) | 114.43 (1.36, 1.36) | 0.608 |
| FCM vs OFS | 148.41 (3.97, 3.97) | 17.46 (0.01, 0.01) | 0.608 |
| FDI vs FSC | 270.43 (20.21, 20.21) | 16236.95 (478.74, 478.74) | 0.067 |
| FDI vs ISC | 2.68 (0.84, 0.84) | 688.11 (4.27, 4.27) | 0.037* |
| FDI vs OFS | 15.86 (0.74, 0.74) | 0.99 (0.02, 0.02) | 0.278 |
| FSC vs ISC | 0 (0, 0) | 0.06 (0, 0) | 0.221 |
| FSC vs OFS | 0 (0, 0) | 0.02 (0, 0) | 0.181 |
| ISC vs OFS | 0.07 (0, 0) | 27.7 (0.96, 0.96) | 0.017* |
| **5 week** | | | |
| FCM vs FDI | 31.33 (0.4, 0.4) | 3.36 (0, 0) | 0.662 |
| FCM vs FXM | 14.88 (0.06, 0.06) | 138.66 (0.03, 0.03) | 0.662 |
| FDI vs ISC | 33.12 (0.13, 0.13) | 3.55 (0, 0) | 0.662 |
| FXM vs ISC | 7.48 (0.09, 0.09) | 69.72 (0.01, 0.01) | 0.662 |
| **6 week** | | | |
| FCM vs ISC | 9.03 (0.01, 0.01) | 9348.44 (197.6, 197.6) | 0.082 |
| FCM vs OFS | 282.3 (24.52, 24.52) | 0.27 (0, 0) | 0.082 |
| ISC vs OFS | 0.03 (0, 0) | 31.28 (0.02, 0.02) | 0.082 |
| **8 week** | | | |
| FCM vs ISC | 164.02 (0.25, 0.25) | 16.88 (0.43, 0.43) | 0.551 |
| FCM vs OFS | 81.45 (4.91, 4.91) | 791.64 (0.78, 0.78) | 0.551 |
| FDI vs ISC | 1.84 (0.67, 0.67) | 17.9 (0.01, 0.01) | 0.551 |
| FDI vs OFS | 8.89 (1.04, 1.04) | 0.91 (0, 0) | 0.551 |

**S11.2 – Ferritin**

| **1 week** | | | |
| --- | --- | --- | --- |
| **Comparison** | **direct** | **indirect** | **p-value** |
| FCM vs FDI | 1.877 | 2.1011 | 0.116 |
| FCM vs OFS | 1.53 | 1.3706 | 0.116 |
| FDI vs OFS | 7.3 | 8.171 | 0.116 |
| **2 week** | | | |
| FCM vs FDI | 5.021 | 0 | 0.280 |
| FCM vs FXM | 7.16 | 0 | 0.218 |
| FCM vs ISC | 0 | 9.0994 | 0.001* |
| FCM vs OFS | 7.5 | 5.20861 | 0.158 |
| FDI vs ISC | 1.113 | 3.3805 | 0.808 |
| FDI vs OFS | 3.5 | 2.55273 | 0.344 |
| FXM vs ISC | 2.28 | 0 | 0.218 |
| ISC vs OFS | 0 | 6.4435 | 0.031* |
| **3 week** | | | |
| FCM vs FDI | 2.864 | 6.0154 | 0.248 |
| FCM vs OFS | 9.05 | 4.3085 | 0.248 |
| FDI vs ISC | 1.451 | 6.2709 | 0.890 |
| FDI vs OFS | 9.762 | 4.5076 | 0.338 |
| OFS vs ISC | 152.933 | 0 | 0.890 |
| **4 week** | | | |
| FCM vs ISC | 0 | 1.5609 | 0.003* |
| FCM vs OFS | 1.34 | 0 | 0.003* |
| FDI vs ISC | 2.26 | 5.068 | 0.081 |
| FDI vs OFS | 5.309 | 0 | 0.081 |
| ISC vs OFS | 0 | 4.76247 | 0.000* |
| **5 week** | | | |
| FCM vs FDI | 1.083 | 3.1233 | 0.088 |
| FCM vs FXM | 4.101 | 0 | 0.088 |
| FDI vs ISC | 1.546 | 4.4562 | 0.088 |
| FXM vs ISC | 1.177 | 0 | 0.088 |
| **6 week** | | | |
| FCM vs ISC | 0 | 1.4765 | 0.041* |
| FCM vs OFS | 4.82 | 0 | 0.041* |
| ISC vs OFS | 0 | 4.6308 | 0.041* |
| **8 week** | | | |
| FCM vs ISC | 0 | 7.2734 | 0.0008* |
| FCM vs OFS | 2.81 | 0 | 0.0008* |
| FDI vs ISC | 11.5133 | 1.83109 | 0.021 |
| FDI vs OFS | 3.976 | 0 | 0.021 |
| ISC vs OFS | 0 | 6.35052 | 0.0002* |

**S11.3 – Any Adverse Event**

| **Comparison** | **direct** | **indirect** | **p-value** |
| --- | --- | --- | --- |
| FCM vs FDI | 0.9796 | 0.989 | 0.920 |
| FCM vs FXM | 1.1291 | 1.199 | 0.578 |
| FCM vs ISC | 1.1761 | 1.079 | 0.455 |
| FCM vs OFS | 1.0037 | 1.028 | 0.803 |
| FCM vs PCB | 1.403 | 1.296 | 0.666 |
| FDI vs ISC | 1.1099 | 1.14 | 0.875 |
| FDI vs OFS | 1.0216 | 1.023 | 0.991 |
| FXM vs ISC | 0.9569 | 0.993 | 0.748 |
| FXM vs PCB | 1.1284 | 1.222 | 0.666 |
| OFS vs ISC | 0.8333 | 1.122 | 0.367 |

**S11.4 – Serious Adverse Event**

| **Comparison** | **direct** | **indirect** | **p-value** |
| --- | --- | --- | --- |
| FCM vs FDI | 1.1755 | 1.026 | 0.822 |
| FCM vs FXM | 0.9693 | 1.284 | 0.364 |
| FCM vs ISC | 1.4616 | 1.138 | 0.606 |
| FCM vs ISFG | 0.3709 | 2.398 | 0.006* |
| FCM vs OFS | 1.258 | 0.79 | 0.059 |
| FCM vs SCA | 0.399 | 1.317 | 0.006* |
| FDI vs ISC | 1.0817 | 1.124 | 0.953 |
| FDI vs OFS | 0.8413 | 1.836 | 0.034* |
| FDI vs SCA | 0.9577 | 0.29 | 0.006* |
| FXM vs ISC | 0.9822 | 1.301 | 0.364 |
| ISFG vs SCA | 1.0759 | 21.81 | 0.006* |
| OFS vs ISC | 1.0497 | 0.903 | 0.587 |

**S11.5 – Hypophosphatemia**

| **Comparison** | **direct** | **indirect** | **p-value** |
| --- | --- | --- | --- |
| FCM vs FDI | 8.1688 | 2.4029 | 0.260 |
| FCM vs ISC | 5.301 | 22.111 | 0.200 |
| FCM vs OFS | 19.5396 | 9.8697 | 0.78**1** |
| FDI vs ISC | 2.6749 | 0.6413 | 0.200 |
| FDI vs OFS | 2.0526 | 4.0637 | 0.781 |

# Supplement 12 - Results to Hb - Subgroup Analysis: Renal

| **Intravenous Iron Agents** | **Comparator** | **Timepoint**  **(Weeks)** | **Estimatives** | **MD** | **95% CI** | **Certainly of Evidence** | **TREND** |
| --- | --- | --- | --- | --- | --- | --- | --- |
| **FCM** | **FDI** | 2w | NMA | 0,20 | (-0.08, 0.48) | Moderate | 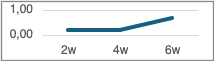 |
|  | **FDI** | 4w | NMA | 0,20 | (-0.02, 0.42) | Moderate |  |
|  | **FDI** | 6w | NMA | 0,70 | (0.23, 1.17) | Moderate |  |
|  | **ISC** | 2w | NMA | 0,39 | (0.09, 0.70) | Low | 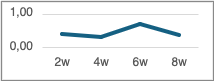 |
|  | **ISC** | 4w | NMA | 0,30 | (0.03, 0.57) | Very Low |  |
|  | **ISC** | 6w | NMA | 0,70 | (0.36, 1.04) | Moderate |  |
|  | **ISC** | 8w | NMA | 0,36 | (0.02, 0.70) | Low |  |
|  | **FXM** | 2w | NMA | 0,09 | (-0.23, 0.42) | Low | 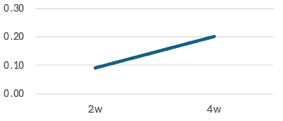 |
|  | **FXM** | 4w | NMA | 0,20 | (-0.07, 0.47) | Very Low |  |
|  | **SGC** | 6w | NMA | 0,20 | (-0.22, 0.62) | Very Low | - |
|  | **OFS** | 2w | NMA | 0,20 | (0.00, 0.40) | Low | 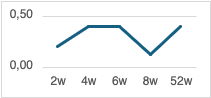 |
|  | **OFS** | 4w | NMA | 0,40 | (0.23, 0.57) | Low |  |
|  | **OFS** | 6w | NMA | 0,40 | (0.22, 0.58) | Very Low |  |
|  | **OFS** | 8w | NMA | 0,12 | (-0.24, 0.47) | Low |  |
|  | **OFS** | 52w | Direct | 0,40 | (-2.97, 3.77) | Low |  |
| **FDI** | **ISC** | 1w | Direct | 0,20 | (0.08, 0.32) | Moderate | 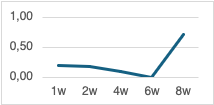 |
|  | **ISC** | 2w | NMA | 0,19 | (0.09, 0.30) | Low |  |
|  | **ISC** | 4w | NMA | 0,10 | (-0.06, 0.26) | Very Low |  |
|  | **ISC** | 6w | NMA | 0,00 | (-0.32, 0.32) | Moderate |  |
|  | **ISC** | 8w | NMA | 0,72 | (0.46, 0.99) | Low |  |
|  | **FXM** | 2w | NMA | -0,11 | (-0.27, 0.05) | Low | 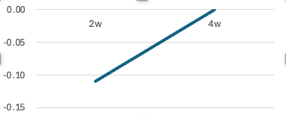 |
|  | **FXM** | 4w | NMA | 0,00 | (-0.16, 0.16) | Very Low |  |
|  | **SGC** | 6w | NMA | -0,50 | (-1.08, 0.08) | Very Low | - |
|  | **OFS** | 2w | NMA | 0,00 | (-0.20, 0.20) | Moderate | 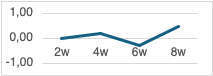 |
|  | **OFS** | 4w | NMA | 0,20 | (0.06, 0.34) | Moderate |  |
|  | **OFS** | 6w | NMA | -0,30 | (-0.73, 0.13) | Low |  |
|  | **OFS** | 8w | NMA | 0,48 | (0.13, 0.83) | Moderate |  |
| **ISC** | **FXM** | 2w | NMA | -0,30 | (-0.42, -0.18) | Very Low | 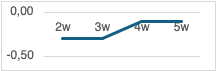 |
|  | **FXM** | 3w | NMA | -0,30 | (-0.33, -0.27) | Very Low |  |
|  | **FXM** | 4w | NMA | -0,10 | (-0.13, -0.07) | Very Low |  |
|  | **FXM** | 5w | NMA | -0,10 | (-0.13, -0.07) | Very Low |  |
|  | **IDX** | 3w | NMA | 0,20 | (-0.19, 0.59) | Very Low | - |
|  | **SGC** | 6w | NMA | -0,50 | (-0.98, -0.02) | Moderate | - |
|  | **OFS** | 2w | NMA | -0,19 | (-0.42, 0.03) | Low | 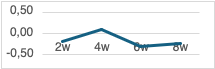 |
|  | **OFS** | 4w | NMA | 0,10 | (-0.11, 0.31) | Very Low |  |
|  | **OFS** | 6w | NMA | -0,30 | (-0.59, -0.01) | Low |  |
|  | **OFS** | 8w | NMA | -0,25 | (-0.57, 0.08) | Moderate |  |
| **FXM** | **IDX** | 3w | NMA | 0,50 | (0.11, 0.89) | Very Low | - |
|  | **OFS** | 2w | NMA | 0,11 | (-0.15, 0.36) | Very Low | 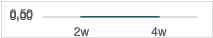 |
|  | **OFS** | 4w | NMA | 0,20 | (-0.01, 0.41) | Very Low |  |

**Legend:** FCM - Ferric carboxymaltose, FDI - Ferric derisomaltose, FXM – Ferumoxytol, IDX - Iron dextran, ISC - Iron sucrose, OFS - Oral Ferrous Sulfate, SGC – Sodium ferric gluconate complex.

# Supplement 13 - Results to Hb - Subgroup Analysis: Gastrointestinal

| **Intravenous Iron Agents** | **Comparator** | **Timepoint   (Weeks)** | **Estimatives** | **MD** | **95% CI** | **Certainly of Evidence** | **TREND** |
| --- | --- | --- | --- | --- | --- | --- | --- |
| **FCM** | **FDI** | 2w | Network | 0,00 | (-0.62, 0.62) | Moderate | 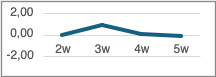 |
|  |  | 3w | Network | 0,90 | (0.19, 1.61) | Moderate |  |
|  |  | 4w | Network | 0,10 | (-0.48, 0.68) | Moderate |  |
|  |  | 5w | Direct | -0,10 | (-0,67, 0,47) | Low |  |
|  | **ISC** | 2w | Network | 0,80 | (0.29, 1.31) | Low | 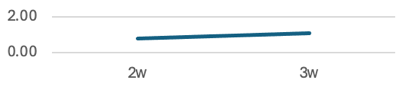 |
|  |  | 3w | Network | 1,10 | (0.39, 1.81) | Low |  |
|  | **OFS** | 2w | Network | 0,00 | (-0.78, 0.78) | Low | 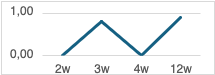 |
|  |  | 3w | Network | 0,80 | (0.30, 1.30) | Moderate |  |
|  |  | 4w | Network | 1,20 | (0.50, 1.90) | Moderate |  |
|  |  | 12w | Network | 0,90 | (-4.24, 6.04) | Low |  |
| **FDI** | **ISC** | 3w | Network | -0,10 | (-0.61, 0.41) | Very Low | 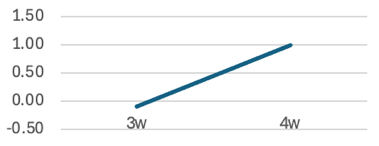 |
|  |  | 4w | Network | 1,00 | (0.08, 1.92) | Low |  |
|  | **OFS** | 1w | Direct | 0,30 | (-0,02, 0,42) | Moderate | 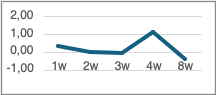 |
|  |  | 2w | Network | 0,00 | (-0,47, 0,47) | Moderate |  |
|  |  | 3w | Network | -0,10 | (-0.60, 0.40) | Very Low |  |
|  |  | 4w | Network | 1,10 | (0.19, 2.01) | High |  |
|  |  | 8w | Direct | -0,39 | (-0.45, -0.33) | Moderate |  |
| **ISC** | **OFS** | 3w | Network | 0,00 | (-0.09, 0.09) | Very Low | 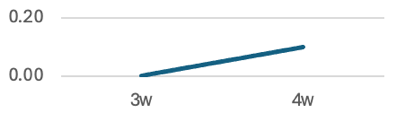 |
|  |  | 4w | Network | 0,10 | (-0.02, 0.22) | Very Low |  |

**Legend:** FCM - Ferric carboxymaltose, FDI - Ferric derisomaltose, FXM – Ferumoxytol, IDX - Iron dextran, ISC - Iron sucrose, OFS - Oral Ferrous Sulfate, SGC – Sodium ferric gluconate complex.

# Supplement 14 - Results to Ferritin - Subgroup Analysis: Renal

| **Intravenous  Iron Agents** | **Comparator** | **Timepoint   (Weeks)** | **Estimative** | **MD** | **95% CI** | **Certainly of Evidence** | **TREND** |
| --- | --- | --- | --- | --- | --- | --- | --- |
| **FCM** | **FDI** | 2w | Netwrok | 587,15 | (116.89, 1,057.42) | Moderate | 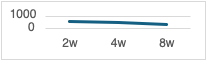 |
|  |  | 4w | Network | 485,03 | (134.50, 835.57) | Low |  |
|  |  | 8w | Network | 321,02 | (-34.52, 676.57) | Very low |  |
|  | **ISC** | 2w | Netwrok | 754,37 | (288.22, 1,222.52) | Low | 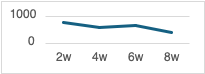 |
|  |  | 4w | Network | 571,06 | (223.26, 918.86) | Low |  |
|  |  | 6w | Network | 657,45 | (413.88, 901.02) | Very low |  |
|  |  | 8w | Network | 396,57 | (42.96, 750.18) | Very low |  |
|  | **FXM** | 2w | Netwrok | 507,17 | (-4.14, 1,018.49) | Low | 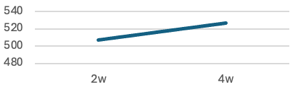 |
|  |  | 4w | Network | 526,66 | (130.83, 922.49) | Very low |  |
|  | **SGC** | 6w | Network | 235,30 | (-21.19, 491.79) | Very low | - |
|  | **OFS** | 2w | Netwrok | 612,20 | (175.44, 1,048.96) | Moderate | 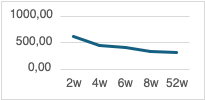 |
|  |  | 4w | Network | 451,6 | (135.87, 767.33) | Low |  |
|  |  | 6w | Network | 411,40 | (171.54, 651.26) | Very low |  |
|  |  | 8w | Network | 328,00 | (26.79, 629.21) | Very low |  |
|  |  | 52w | Direct | 314,00 | (288.91, 339.09) | Moderate |  |
| **FDI** | **ISC** | 1w | Network | 254,37 | (125.15, 358.58) | Low | 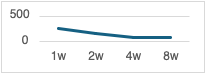 |
|  |  | 2w | Netwrok | 167,22 | (58.30, 276.14) | Low |  |
|  |  | 4w | Network | 86,02 | (-11.57, 183.62) | Very low |  |
|  |  | 8w | Network | 75,55 | (-74.67, 225.77) | Very low |  |
|  | **FXM** | 2w | Netwrok | -79,98 | (-316.64, 156.69) | Very low | 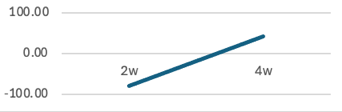 |
|  |  | 4w | Network | 41,62 | (-171.08, 254.32) | Very low |  |
|  | **OFS** | 1w | Network | -66,63 | (-380.92, 247.66) | Low | 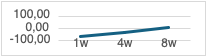 |
|  |  | 4w | Network | -33,43 | (-185.72, 118.69) | Low |  |
|  |  | 8w | Network | 6,98 | (-181.93, 195.88) | Very low |  |
| **ISC** | **FXM** | 2w | Netwrok | -247,20 | (-457.31, -37.09) | Low | 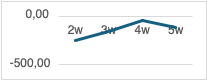 |
|  |  | 3w | Network | -156,50 | (-246.12, -66,88) | Very low |  |
|  |  | 4w | Network | -44,40 | (-233.39, 144.59) | Very low |  |
|  |  | 5w | Network | -122,20 | (-194.84, -49.56) | Low |  |
|  | **IDX** | 3w | Network | -2,70 | (-55.58, 50.18) | Very low | - |
|  | **SGC** | 6w | Network | -176,10 | (-266.96, -85.24) | Very low | - |
|  | **OFS** | 4w | Network | -119,46 | (-265.34, 26.43) | Very low | 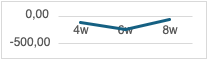 |
|  |  | 6w | Network | -246,05 | (-288.41, 203.69) | Low |  |
|  |  | 8w | Network | -68,57 | (-253.80, 116.66) | Very low |  |
| **FXM** | **IDX** | 3w | Network | 153,80 | (49.74, 257.86) | Very low | - |
|  | **OFS** | 4w | Network | -75,06 | (-313.80, 163.69) | Very low | - |
| **SGC** | **OFS** | 6w | Network | 176,10 | (85.24, 266.96) | Very low | - |

**Legend:** FCM - Ferric carboxymaltose, FDI - Ferric derisomaltose, FXM – Ferumoxytol, IDX - Iron dextran, ISC - Iron sucrose, OFS - Oral Ferrous Sulfate, SGC – Sodium ferric gluconate complex.

# Supplement 15 - Results to Ferritin - Subgroup Analysis: GI

| **Intravenous  Iron Agents** | **Comparator** | **Timepoint   (Weeks)** | **Estimative** | **MD** | **95% CI** | **Certainly of Evidence** | **TREND** |
| --- | --- | --- | --- | --- | --- | --- | --- |
| **FCM** | **FDI** | 1w | Network | 109,40 | (26.83, 191.97) | Moderate | 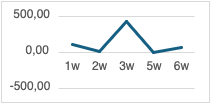 |
|  |  | 2w | Netwrok | 7,40 | (-38.07, 52.87) | Low |  |
|  |  | 3w | Network | 423,60 | (396.03, 451.17) | Moderate |  |
|  |  | 5w | Direct | -5,90 | (-33.63, 21.83) | Moderate |  |
|  |  | 6w | Network | 59,10 | (-17.20, 135.40) | Low |  |
|  | **ISC** | 1w | Network | 599,10 | (598.15, 600.05) | Moderate | 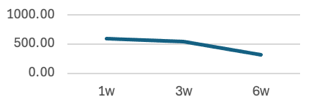 |
|  |  | 3w | Network | 539,10 | (537.76, 540.44) | Very Low |  |
|  |  | 6w | Network | 321,20 | (320.07, 322.33) | Very Low |  |
|  | **OFS** | 1w | Network | 599,10 | (598.15, 600.05) | Moderate | 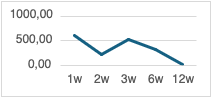 |
|  |  | 2w | Netwrok | 226,20 | (179.04, 273.36) | Moderate |  |
|  |  | 3w | Network | 534,10 | (582.80, 535.40) | Moderate |  |
|  |  | 6w | Network | 315,10 | (314.01, 316.19) | Low |  |
|  |  | 12w | Network | 16,50 | (-3.21, 36.21) | Low |  |
| **FDI** | **ISC** | 3w | Network | 115,50 | (87.95, 143.05) | Very Low | 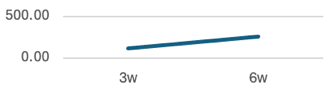 |
|  |  | 6w | Network | 262,10 | (185.79, 338.41) | Moderate |  |
|  | **OFS** | 1w | Network | 489,70 | (407.13, 572.27) | Moderate | 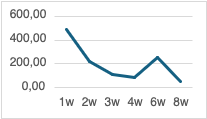 |
|  |  | 2w | Netwrok | 218,80 | (206.29, 231.31) | Moderate |  |
|  |  | 3w | Network | 110,50 | (82.96, 138.04) | Moderate |  |
|  |  | 4w | Network | 86,50 | (68.64, 104.36) | Moderate |  |
|  |  | 6w | Network | 256,00 | (178.69, 332.31) | Low |  |
|  |  | 8w | Direct | 49,40 | (38.58, 60.22) | Moderate |  |
| **ISC** | **OFS** | 3w | Network | -5,00 | (-5.33, -4.67) | Moderate | 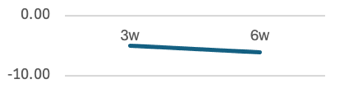 |
|  |  | 6w | Network | -6,10 | (-6.40, -5.80) | Moderate |  |

**Legend:** FCM - Ferric carboxymaltose, FDI - Ferric derisomaltose, FXM – Ferumoxytol, IDX - Iron dextran, ISC - Iron sucrose, OFS - Oral Ferrous Sulfate, SGC – Sodium ferric gluconate complex.

# Supplement 16 - Results to Transferrin Saturation (TSAT) - Subgroup Analysis: Renal

| **Intravenous  Iron Agents** | **Comparator** | **Timepoint   (Weeks)** | **Estimative** | **MD** | **95% CI** | **Certainly of Evidence** | **TREND** |
| --- | --- | --- | --- | --- | --- | --- | --- |
| **FCM** | **FDI** | 2w | Network | 4,99 | (-1.66, 11.63) | Moderate | 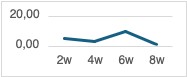 |
|  |  | 4w | Network | 2,76 | (-2.84, 8.37) | Low |  |
|  |  | 6w | Network | 9,90 | (4.53, 15.27) | Moderate |  |
|  |  | 8w | Network | 1,20 | (-1.91, 4.31) | Low |  |
|  | **ISC** | 2w | Network | 6,97 | (0.28, 13.66) | High | 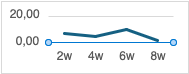 |
|  |  | 4w | Network | 4,36 | (-1.31, 10.04) | Low |  |
|  |  | 6w | Network | 9,80 | (4.44, 15.16) | Moderate |  |
|  |  | 8w | Network | 1,67 | (-1.55, 4.89) | Very Low |  |
|  | **FXM** | 2w | Network | 2,57 | (-6.41, 11.55) | Moderate | 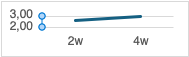 |
|  |  | 4w | Network | 2,96 | (-5.00, 10.92) | Very Low |  |
|  | **SGC** | 6w | Network | 1,20 | (-4.13, 6.53) | Very Low | - |
|  | **OFS** | 2w | Network | 7,80 | (2.30, 13.30) | Moderate | 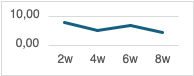 |
|  |  | 4w | Network | 5,00 | (0.57, 9.43) | Moderate |  |
|  |  | 6w | Network | 6,60 | (2.76, 10.44) | Moderate |  |
|  |  | 8w | Network | 4,40 | (1.93, 6.87) | Low |  |
| **FDI** | **ISC** | 1w | Network | 6,54 | (1.17, 11.91) | Low | 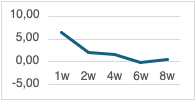 |
|  |  | 2w | Network | 1,98 | (-0.08, 4.05) | Very Low |  |
|  |  | 4w | Network | 1,60 | (-0.82, 4.02) | Very Low |  |
|  |  | 6w | Network | -0,10 | (-0.42, 0.22) | Very Low |  |
|  |  | 8w | Network | 0,47 | (-0.40, 1.34) | Low |  |
|  | **FXM** | 2w | Network | -2,42 | (-8.75, 3.92) | Moderate | 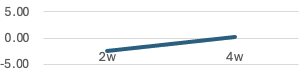 |
|  |  | 4w | Network | 0,20 | (-5.89, 6.29) | Very Low |  |
|  | **SGC** | 6w | Network | -8,70 | (-13.96, -3.44) | Very Low | - |
|  | **OFS** | 1w | Network | 8,40 | (-0.36, 17.16) | Low | 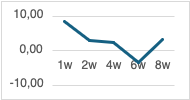 |
|  |  | 2w | Network | 2,81 | (-0.91, 6.54) | Low |  |
|  |  | 4w | Network | 2,24 | (-1.20, 5.67) | Moderate |  |
|  |  | 6w | Network | -3,30 | (-7.05, 0.45) | Very Low |  |
|  |  | 8w | Network | 3,20 | (1.32, 5.08) | Moderate |  |
| **ISC** | **FXM** | 2w | Network | -4,40 | (-10.39, 1.59) | Low | 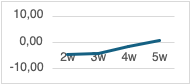 |
|  |  | 3w | Network | -4,20 | (-8.49, 0.09) | Low |  |
|  |  | 4w | Network | -1,40 | (-6.99, 4.19) | Very Low |  |
|  |  | 5w | Network | 0,60 | (-3.90, 5.10) | Low |  |
|  | **IDX** | 3w | Network | -0,70 | (-4.48, 3.08) | Very Low | - |
|  | **SGC** | 6w | Network | -8,60 | (-13.85, -3.35) | Very Low | - |
|  | **OFS** | 1w | Network | 1,86 | (-8.41, 12.14) | Very Low | 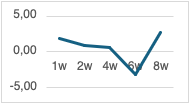 |
|  |  | 2w | Network | 0,83 | (-2.98, 4.64) | Low |  |
|  |  | 4w | Network | 0,64 | (-2.91, 4.19) | Low |  |
|  |  | 6w | Network | -3,20 | (-6.93, 0.53) | Very Low |  |
|  |  | 8w | Network | 2,73 | (0.66, 4.80) | Low |  |
| **FXM** | **IDX** | 3w | Network | 3,50 | (-2.22, 9.22) | Low | - |
|  | **OFS** | 2w | Network | 5,23 | (-1.87, 12.33) | Moderate | 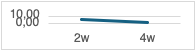 |
|  |  | 4w | Network | 2,04 | (-4.58, 8.66) | Very Low |  |
| **SGC** | **OFS** | 6w | Network | 5,40 | (1.71, 9.09) | Very Low | - |

**Legend:** FCM - Ferric carboxymaltose, FDI - Ferric derisomaltose, FXM – Ferumoxytol, IDX - Iron dextran, ISC - Iron sucrose, OFS - Oral Ferrous Sulfate, SGC – Sodium ferric gluconate complex.

# Supplement 17- Results to Transferrin Saturation (TSAT) - Subgroup Analysis: GI

| **Intravenous  Iron Agents** | **Comparator** | **Timepoint   (Weeks)** | **Estimative** | **MD** | **95% CI** | **Certainly of Evidence** | **TREND** |
| --- | --- | --- | --- | --- | --- | --- | --- |
| **FCM** | **FDI** | 1w | Network | 1,12 | (-14.90. 17.13) | Low | 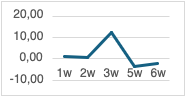 |
|  |  | 2w | Network | 0,70 | (-4.42, 5.82) | Low |  |
|  |  | 3w | Network | 12,20 | (6.01, 18.39) | Moderate |  |
|  |  | 5w | Network | -3,40 | (-9.02, 2.22) | Moderate |  |
|  |  | 6w | Network | -2,10 | (-9.86, 5.66) | Low |  |
|  | **ISC** | 3w | Network | 11,40 | (9.85, 12.95) | Moderate | 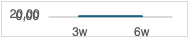 |
|  |  | 6w | Network | 8,80 | (7.68, 9.92) | Very Low |  |
|  | **OFS** | 1w | Network | 2,28 | (-13.30, 17.86) | Very Low | 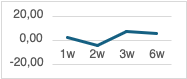 |
|  |  | 2w | Network | -4,60 | (-12.80, 3.60) | Low |  |
|  |  | 3w | Network | 7,60 | (7.10, 8.10) | Moderate |  |
|  |  | 6w | Network | 5,30 | (4.82, 5.78) | Very Low |  |
| **FDI** | **ISC** | 3w | Network | -0,80 | (-7.14, 5.54) | Low | 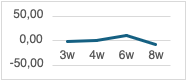 |
|  |  | 4w | Network | -0,70 | (-7.61, 6.21) | Very Low |  |
|  |  | 6w | Network | 10,90 | (3.06, 18.74) | Low |  |
|  |  | 8w | Network | -7,60 | (-13.30, -1.90) | Low |  |
|  | **OFS** | 1w | Network | 1,16 | (-14.88, 17.21) | Very Low | 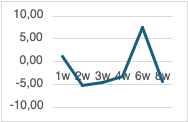 |
|  |  | 2w | Network | -5,30 | (-11.71, 1.11) | Low |  |
|  |  | 3w | Network | -4,60 | (-10.77, 1.57) | Moderate |  |
|  |  | 4w | Network | -3,30 | (-9.14, 2.54) | Moderate |  |
|  |  | 6w | Network | 7,40 | (-0.38, 15.18) | Very Low |  |
|  |  | 8w | Network | -4,30 | (-9.98, 1.38) | Low |  |
| **ISC** | **OFS** | 3w | Network | -3,80 | (-5.26, -2.34) | Moderate | 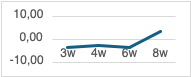 |
|  |  | 4w | Network | -2,60 | (-6.30, 1.10) | Moderate |  |
|  |  | 6w | Network | -3,50 | (-4.51, -2.49) | Very Low |  |
|  |  | 8w | Network | 3,30 | (2.85, 3.75) | Low |  |

**Legend:** FCM - Ferric carboxymaltose, FDI - Ferric derisomaltose, FXM – Ferumoxytol, IDX - Iron dextran, ISC - Iron sucrose, OFS - Oral Ferrous Sulfate, SGC – Sodium ferric gluconate complex.

# Supplement 18 - Results to Any Adverse Event - Overall Analysis

| **Intravenous agent** | **Comparator** | **Estimative** | **MD** | **95% CI** | **Certainty of Evidence** |
| --- | --- | --- | --- | --- | --- |
| FCM | FDI | Network | 0.98 | (0.90, 1.08) | Low |
|  | ISC | Network | 1.12 | (1.00, 1.25) | Low |
|  | FXM | Network | **1.16** | **(1.04, 1.28)** | Moderate |
|  | IDX | Network | 0.60 | (0.22, 1.62) | Low |
|  | OFS | Network | 1.01 | (0.95, 1.07) | Low |
| FDI | ISC | Network | 1.13 | (0.99, 1.30) | Low |
|  | FXM | Network | **1.17** | **(1.03, 1.34)** | Low |
|  | IDX | Network | 0.61 | (0.23, 1.65) | Low |
|  | OFS | Network | 1.02 | (0.93, 1.12) | Low |
| ISC | FXM | Network | 1.04 | (0.94, 1.14) | Low |
|  | IDX | Network | 0.54 | (0.20, 1.44) | Low |
|  | OFS | Network | 0.9 | (0.80, 1.02) | Low |
| FXM | IDX | Network | 0.52 | (0.19, 1.40) | Low |
|  | OFS | Network | **0.87** | **(0.77, 0.98)** | Low |
| IDX | OFS | Network | 1.67 | (0.62, 4.51) | Low |

**Ranking**

**Legend:** CAI - Carbonil iron tablet, CTL – Control, FAS - Ferrous ascorbate, FCM - Ferric carboxymaltose, FDI - Ferric derisomaltose, FFM - Ferrous fumarate, FSC - Ferrous succinate, FXM – Ferumoxytol, IDX - Iron dextran, ISC - Iron sucrose, ISFG - Iron sucrose or sodium ferric gluconate, IVSC - Intravenous Standard-of-care, OFM - Oral ferric maltol, OFS - Oral Ferrous Sulfate, OLS - Oral lipossomal iron, PCB – Placebo, SCA - Standard of care, SFO - Saccharated ferric oxide, SGC – Sodium ferric gluconate complex, SUI - Sucrosial Iron.

**League Table – Any adverse event**

| **Intravenous agent** | **CTL** | **FCM** | **FDI** | **FFM** | **FXM** | **IDX** | **ISC** | **ISFG** | **OFM** | **OFS** | **PCB** | **SCA** | **SFO** |
| --- | --- | --- | --- | --- | --- | --- | --- | --- | --- | --- | --- | --- | --- |
| **CTL** | CTL | 0.15  (0.02, 1.02) | . | . | . | . | . | . | . | . | . | . | . |
| **FCM** | 0.15  (0.02, 1.02) | FCM | 0.98  (0.85, 1.13) | . | 1.13  (0.99, 1.29) | . | 1.18  (0.99, 1.40) | 0.77  (0.58, 1.01) | 0.60  (0.45, 0.80) | 1.00  (0.94, 1.07) | 1.40  (1.04, 1.90) | 0.92  (0.71, 1.20) | 1.01  (0.81, 1.27) |
| **FDI** | 0.14  (0.02, 1.00) | 0.98  (0.90, 1.08) | FDI | . | . | . | 1.11  (0.82, 1.50) | . | . | 1.02  (0.90, 1.16) | . | . | . |
| **FFM** | 0.15  (0.02, 1.04) | 1.00  (0.76, 1.32) | 1.02  (0.76, 1.36) | FFM | 1.15  (0.89, 1.49) | . | . | . | . | . | . | . | . |
| **FXM** | 0.17  (0.02, 1.18) | 1.16  (1.04, 1.28) | 1.17  (1.03, 1.34) | 1.15  (0.89, 1.49) | FXM | . | 0.96  (0.86, 1.07) | . | . | . | 1.13  (0.96, 1.33) | . | . |
| **IDX** | 0.09  (0.01, 0.78) | 0.60  (0.22, 1.62) | 0.61  (0.23, 1.65) | 0.60  (0.22, 1.67) | 0.52  (0.19, 1.40) | IDX | 1.85  (0.69, 4.96) | . | . | . | . | . | . |
| **ISC** | 0.16  (0.02, 1.14) | 1.12  (1.00, 1.25) | 1.13  (0.99, 1.30) | 1.11  (0.85, 1.46) | 0.97  (0.88, 1.06) | 1.85  (0.69, 4.96) | ISC | . | . | 1.20  (0.64, 2.26) | . | . | . |
| **ISFG** | 0.11  (0.02, 0.80) | 0.77  (0.58, 1.01) | 0.78  (0.58, 1.04) | 0.77  (0.52, 1.13) | 0.66  (0.49, 0.89) | 1.28  (0.46, 3.57) | 0.69  (0.51, 0.93) | ISFG | . | . | . | 1.20  (0.92, 1.57) | . |
| **OFM** | 0.09  (0.01, 0.63) | 0.60  (0.45, 0.80) | 0.61  (0.45, 0.83) | 0.60  (0.40, 0.89) | 0.52  (0.38, 0.71) | 1.00  (0.36, 2.80) | 0.54  (0.40, 0.73) | 0.78  (0.53, 1.17) | OFM | . | . | . | . |
| **OFS** | 0.15  (0.02, 1.03) | 1.01  (0.95, 1.07) | 1.02  (0.93, 1.12) | 1.00  (0.76, 1.33) | 0.87  (0.77, 0.98) | 1.67  (0.62, 4.51) | 0.90  (0.80, 1.02) | 1.31  (0.99, 1.74) | 1.67  (1.25, 2.24) | OFS | . | . | . |
| **PCB** | 0.19  (0.03, 1.36) | 1.33  (1.12, 1.57) | 1.35  (1.12, 1.63) | 1.32  (0.98, 1.78) | 1.15  (0.99, 1.33) | 2.20  (0.81, 5.99) | 1.19  (1.00, 1.41) | 1.73  (1.25, 2.38) | 2.20  (1.58, 3.07) | 1.32  (1.11, 1.57) | PCB | . | . |
| **SCA** | 0.13  (0.02, 0.96) | 0.92  (0.71, 1.20) | 0.94  (0.71, 1.24) | 0.92  (0.63, 1.35) | 0.80  (0.60, 1.06) | 1.53  (0.55, 4.28) | 0.83  (0.62, 1.10) | 1.20  (0.92, 1.57) | 1.53  (1.04, 2.26) | 0.92  (0.70, 1.20) | 0.70  (0.51, 0.95) | SCA | . |
| **SFO** | 0.15  (0.02, 1.05) | 1.01  (0.81, 1.27) | 1.03  (0.81, 1.31) | 1.01  (0.71, 1.44) | 0.88  (0.69, 1.12) | 1.69  (0.61, 4.65) | 0.91  (0.71, 1.17) | 1.32  (0.93, 1.88) | 1.69  (1.17, 2.42) | 1.01  (0.80, 1.27) | 0.76  (0.58, 1.01) | 1.10  (0.78, 1.55) | SFO |

**Legend:** CAI - Carbonil iron tablet, CTL – Control, FAS - Ferrous ascorbate, FCM - Ferric carboxymaltose, FDI - Ferric derisomaltose, FFM - Ferrous fumarate, FSC - Ferrous succinate, FXM – Ferumoxytol, IDX - Iron dextran, ISC - Iron sucrose, ISFG - Iron sucrose or sodium ferric gluconate, IVSC - Intravenous Standard-of-care, OFM - Oral ferric maltol, OFS - Oral Ferrous Sulfate, OLS - Oral lipossomal iron, PCB – Placebo, SCA - Standard of care, SFO - Saccharated ferric oxide, SGC – Sodium ferric gluconate complex, SUI - Sucrosial Iron.

# Supplement 19 - Results to Any Adverse Event - Renal Analysis

| **Intravenous agent** | **Comparator** | **Estimative** | **MD** | **95% CI** | **Certainty of Evidence** |
| --- | --- | --- | --- | --- | --- |
| FCM | FDI | Network | 0.96 | (0.53, 1,74) | Very Low |
|  | ISC | Network | 0.85 | (0.47, 1.66) | Very Low |
|  | IDX | Network | 0.46 | (0.12, 1.82) | Very Low |
|  | OFS | Network | 0.89 | (0.64, 1.23) | Very Low |
| FDI | ISC | Network | 0.88 | (0.47, 1.66) | Very Low |
|  | IDX | Network | 0.48 | (0.14, 1.65) | Very Low |
|  | OFS | Network | 0.92 | (0.56, 1.51) | Very Low |
| ISC | IDX | Network | 0.54 | (0.18, 1.57) | Very Low |
|  | OFS | Network | 1.04 | (0.47, 2.31) | Very Low |
| IDX | OFS | Network | 1.93 | (0.51, 7.33) | Very Low |

**Ranking - Any Adverse Event - Renal subgroup**

**Legend:** CAI - Carbonil iron tablet, CTL – Control, FAS - Ferrous ascorbate, FCM - Ferric carboxymaltose, FDI - Ferric derisomaltose, FFM - Ferrous fumarate, FSC - Ferrous succinate, FXM – Ferumoxytol, IDX - Iron dextran, ISC - Iron sucrose, ISFG - Iron sucrose or sodium ferric gluconate, IVSC - Intravenous Standard-of-care, OFM - Oral ferric maltol, OFS - Oral Ferrous Sulfate, OLS - Oral lipossomal iron, PCB – Placebo, SCA - Standard of care, SFO - Saccharated ferric oxide, SGC – Sodium ferric gluconate complex, SUI - Sucrosial Iron.

**League Table – Any adverse event – Renal subgroup**

| **Intravenous agent** | **FCM** | **FDI** | **IDX** | **ISC** | **ISFG** | **OFS** | **SCA** |
| --- | --- | --- | --- | --- | --- | --- | --- |
| **FCM** | **FCM** | . | . | . | 0.77  (0.46,1.27) | 0.89  (0.64,1.23) | 0.92  (0.56,1.52) |
| **FDI** | 0.96  (0.53,1.74) | FDI | . | 0.88  (0.47,1.66) | . | 0.92  (0.56,1.51) | . |
| **IDX** | 0.46  (0.12,1.82) | 0.48  (0.14,1.65) | IDX | 1.85  (0.64,5.41) | . | . | . |
| **ISC** | 0.85  (0.36,2.02) | 0.88  (0.47,1.66) | 1.85  (0.64,5.41) | ISC | . | . | . |
| **ISFG** | 0.77  (0.46,1.27) | 0.80  (0.37,1.73) | 1.67  (0.39,7.21) | 0.90  (0.33,2.44) | ISFG | . | 1.20  (0.73,1.98) |
| **OFS** | 0.89  (0.64,1.23) | 0.92  (0.56,1.51) | 1.93  (0.51,7.33) | 1.04  (0.47,2.31) | 1.16  (0.63,2.10) | OFS | . |
| **SCA** | 0.92  (0.56,1.52) | 0.96  (0.44,2.07) | 2.01  (0.47,8.66) | 1.08  (0.40,2.93) | 1.20  (0.73,1.98) | 1.04  (0.58,1.88) | SCA |

**Legend:** CAI - Carbonil iron tablet, CTL – Control, FAS - Ferrous ascorbate, FCM - Ferric carboxymaltose, FDI - Ferric derisomaltose, FFM - Ferrous fumarate, FSC - Ferrous succinate, FXM – Ferumoxytol, IDX - Iron dextran, ISC - Iron sucrose, ISFG - Iron sucrose or sodium ferric gluconate, IVSC - Intravenous Standard-of-care, OFM - Oral ferric maltol, OFS - Oral Ferrous Sulfate, OLS - Oral lipossomal iron, PCB – Placebo, SCA - Standard of care, SFO - Saccharated ferric oxide, SGC – Sodium ferric gluconate complex, SUI - Sucrosial Iron.

# Supplement 20 - Results to Any Adverse Event - GI Analysis

| **Intravenous agent** | **Comparator** | **Estimative** | **MD** | **95% CI** | **Certainty of Evidence** |
| --- | --- | --- | --- | --- | --- |
| FCM | FDI | Network | 0.98 | (0.86, 1.11) | Very Low |
|  | ISC | Network | 0.92 | (0.45, 1.88) | Very Low |
|  | OFS | Network | 1.11 | (0.80, 1.54) | Very Low |
| FDI | ISC | Network | 0.94 | (0.47, 1.90) | Very Low |
|  | OFS | Network | 1.13 | (0.84, 1.53) | Very Low |
| ISC | OFS | Network | 1.20 | (0.64, 2.26) | Very Low |

**Ranking - Any Adverse Event - GI sobgroup**

**League Table – Any adverse event – GI subgroup**

| **Intravenous agent** | **FCM** | **FDI** | **ISC** | **OFM** | **OFS** |
| --- | --- | --- | --- | --- | --- |
| **FCM** | FCM | 0.98  (0.86,1.11) | . | 0.60  (0.46,0.80) | . |
| **FDI** | 0.98  (0.86,1.11) | FDI | . | . | 1.13  (0.84,1.53) |
| **ISC** | 0.92  (0.45,1.88) | 0.94  (0.47,1.90) | ISC | . | 1.20  (0.64,2.26) |
| **OFM** | 0.60  (0.46,0.80) | 0.61  (0.45,0.84) | 0.65  (0.30,1.40) | OFM | . |
| **OFS** | 1.11  (0.80,1.54) | 1.13  (0.84,1.53) | 1.20  (0.64,2.26) | 1.84  (1.20,2.84) | OFS |

**Legend:** CAI - Carbonil iron tablet, CTL – Control, FAS - Ferrous ascorbate, FCM - Ferric carboxymaltose, FDI - Ferric derisomaltose, FFM - Ferrous fumarate, FSC - Ferrous succinate, FXM – Ferumoxytol, IDX - Iron dextran, ISC - Iron sucrose, ISFG - Iron sucrose or sodium ferric gluconate, IVSC - Intravenous Standard-of-care, OFM - Oral ferric maltol, OFS - Oral Ferrous Sulfate, OLS - Oral lipossomal iron, PCB – Placebo, SCA - Standard of care, SFO - Saccharated ferric oxide, SGC – Sodium ferric gluconate complex, SUI - Sucrosial Iron.

# Supplement 21 - Results to Serious Adverse Event - Overall Analysis

| **Intravenous agent** | **Comparator** | **Estimative** | **RR** | **95% CI** | **Certainty of evidence** |
| --- | --- | --- | --- | --- | --- |
| FCM | FDI | Network | 1.04 | (0.72, 1.50) | Low |
|  | ISC | Network | 1.16 | (0.89, 1.53) | Low |
|  | FXM | Network | 1.14 | (0.84, 1.54) | Low |
|  | IDX | Network | 0.16 | (0.02, 1.32) | Low |
|  | SGC | Network | 0.55 | (0.05, 6.10) | Low |
|  | OFS | Network | 1.02 | (0.76, 1.37) | Very Low |
| FDI | ISC | Network | 1.12 | (0.74, 1.68) | Low |
|  | FXM | Network | 1.09 | (0.71, 1.69) | Low |
|  | IDX | Network | 0.16 | (0.02, 1.30) | Low |
|  | SGC | Network | 0.53 | (0.05, 5.97) | Low |
|  | OFS | Network | 1.11 | (0.79, 1.58) | Very Low |
| ISC | FXM | Network | 0.98 | (0.79, 1.21) | Low |
|  | IDX | Network | 0.14 | (0.02, 1.11) | Low |
|  | SGC | Network | 0.47 | (0.04, 5.16) | Low |
|  | OFS | Network | 1.00 | (0.78, 1.28) | Low |
| FXM | IDX | Network | 0.14 | (0.02, 1.15) | Low |
|  | SGC | Network | 0.49 | (0.04, 5.33) | Low |
|  | OFS | Network | 1.02 | (0.76, 1.37) | Low |
| IDX | SGC | Network | 3.41 | (0.72, 16.05) | Low |
|  | OFS | Network | 7.16 | 0.88, 58.12) | Low |
| SGC | OFS | Network | 2.10 | (0.19, 23.14) | Low |

**Ranking – Serious adverse events**

**Legend:** CAI - Carbonil iron tablet, CTL – Control, FAS - Ferrous ascorbate, FCM - Ferric carboxymaltose, FDI - Ferric derisomaltose, FFM - Ferrous fumarate, FSC - Ferrous succinate, FXM – Ferumoxytol, IDX - Iron dextran, ISC - Iron sucrose, ISFG - Iron sucrose or sodium ferric gluconate, IVSC - Intravenous Standard-of-care, OFM - Oral ferric maltol, OFS - Oral Ferrous Sulfate, OLS - Oral lipossomal iron, PCB – Placebo, SCA - Standard of care, SFO - Saccharated ferric oxide, SGC – Sodium ferric gluconate complex, SUI - Sucrosial Iron.

**League Table – Serious adverse events**

| **Intravenous agent** | **CTL** | **FCM** | **FDI** | **FFM** | **FXM** | **IDX** | **ISC** | **ISFG** | **OFM** | **OFS** | **PCB** | **SCA** | **SGC** |
| --- | --- | --- | --- | --- | --- | --- | --- | --- | --- | --- | --- | --- | --- |
| **CTL** | CTL | 0.25  (0.01, 5.34) | . | . | . | . | . | . | . | . | . | . | . |
| **FCM** | 0.25  (0.01, 5.34) | FCM | 1.18  (0.38, 3.60) | . | 0.97  (0.61, 1.53) | . | 1.46  (0.59, 3.62) | 0.37  (0.17, 0.83) | 0.35  (0.12, 1.06) | 1.26  (1.03, 1.54) | . | 0.40  (0.19, 0.85) | . |
| **FDI** | 0.26  (0.01, 5.68) | 1.04  (0.72, 1.50) | FDI | . | . | . | 1.08  (0.33, 3.58) | . | . | 0.84  (0.54, 1.30) | . | 0.96  (0.89, 1.03) | . |
| **FFM** | 0.29  (0.01, 6.83) | 1.17  (0.55, 2.49) | 1.12  (0.49, 2.55) | FFM | 0.97  (0.49, 1.95) | . | . | . | . | . | . | . | . |
| **FXM** | 0.28  (0.01, 6.16) | 1.14  (0.84, 1.54) | 1.09  (0.71, 1.69) | 0.97  (0.49, 1.95) | FXM | . | 0.98  (0.78, 1.24) | . | . | . | 0.87  (0.39, 1.94) | . | . |
| **IDX** | 0.04  (0.00, 1.65) | 0.16  (0.02, 1.32) | 0.16  (0.02, 1.30) | 0.14  (0.02, 1.26) | 0.14  (0.02, 1.15) | IDX | 7.19  (0.90,57.49) | . | . | . | . | . | 3.41  (0.72,16.05) |
| **ISC** | 0.29  (0.01, 6.29) | 1.16  (0.89, 1.53) | 1.12  (0.74, 1.68) | 1.00  (0.48, 2.06) | 1.02  (0.83, 1.27) | 7.19  (0.90, 57.49) | ISC | . | . | 0.95  (0.71, 1.28) | . | . | 0.47  (0.04, 5.16) |
| **ISFG** | 0.18  (0.01, 4.10) | 0.72  (0.38, 1.37) | 0.69  (0.38, 1.25) | 0.62  (0.23, 1.64) | 0.63  (0.32, 1.27) | 4.44  (0.50, 39.53) | 0.62  (0.31, 1.22) | ISFG | . | . | . | 1.08  (0.58, 2.00) | . |
| **OFM** | 0.09  (0.00, 2.28) | 0.35  (0.12, 1.06) | 0.34  (0.11, 1.08) | 0.30  (0.08, 1.15) | 0.31  (0.10, 0.97) | 2.18  (0.20, 23.29) | 0.30  (0.10, 0.94) | 0.49  (0.14, 1.76) | OFM | . | . | . | . |
| **OFS** | 0.29  (0.01, 6.22) | 1.16  (0.97, 1.39) | 1.11  (0.79, 1.58) | 0.99  (0.47, 2.11) | 1.02  (0.76, 1.37) | 7.16  (0.88, 58.12) | 1.00  (0.78, 1.28) | 1.61  (0.85, 3.08) | 3.29  (1.07, 10.07) | OFS | . | . | . |
| **PCB** | 0.24  (0.01, 5.92) | 0.99  (0.42, 2.32) | 0.95  (0.38, 2.36) | 0.84  (0.29, 2.44) | 0.87  (0.39, 1.94) | 6.09  (0.65, 57.13) | 0.85  (0.37, 1.94) | 1.37  (0.47, 3.97) | 2.80  (0.69, 11.31) | 0.85  (0.36, 2.00) | PCB | . | . |
| **SCA** | 0.24  (0.01, 5.39) | 0.99  (0.68, 1.43) | 0.95  (0.88, 1.02) | 0.85  (0.37, 1.92) | 0.87  (0.56, 1.35) | 6.10  (0.73, 50.80) | 0.85  (0.56, 1.28) | 1.37  (0.76, 2.49) | 2.80  (0.87, 8.97) | 0.85  (0.60, 1.21) | 1.00  (0.40, 2.50) | SCA | . |
| **SGC** | 0.14  (0.00, 6.74) | 0.55  (0.05, 6.10) | 0.53  (0.05, 5.97) | 0.47  (0.04, 5.73) | 0.49  (0.04, 5.33) | 3.41  (0.72, 16.05) | 0.47  (0.04, 5.16) | 0.77  (0.06, 9.18) | 1.57  (0.11, 22.02) | 0.48  (0.04, 5.24) | 0.56  (0.04, 7.01) | 0.56  (0.05, 6.29) | SGC |

**Legend:** CAI - Carbonil iron tablet, CTL – Control, FAS - Ferrous ascorbate, FCM - Ferric carboxymaltose, FDI - Ferric derisomaltose, FFM - Ferrous fumarate, FSC - Ferrous succinate, FXM – Ferumoxytol, IDX - Iron dextran, ISC - Iron sucrose, ISFG - Iron sucrose or sodium ferric gluconate, IVSC - Intravenous Standard-of-care, OFM - Oral ferric maltol, OFS - Oral Ferrous Sulfate, OLS - Oral lipossomal iron, PCB – Placebo, SCA - Standard of care, SFO - Saccharated ferric oxide, SGC – Sodium ferric gluconate complex, SUI - Sucrosial Iron.

# Supplement 22 - Results to Serious Adverse Event - Renal Analysis

| **Intravenous agent** | **Comparator** | **Estimative** | **MD** | **95% CI** | **Certainty of evidence** |
| --- | --- | --- | --- | --- | --- |
| FCM | FDI | Network | 1.81 | (0.82, 3.98) | Low |
|  | ISC | Network | 1.36 | (0.95, 1.94) | Low |
|  | FXM | Network | 1.43 | (0.93, 2.20) | Low |
|  | IDX | Network | 0.19 | (0.02, 1.56) | Low |
|  | SGC | Network | 0.64 | (0.06, 7.20) | Low |
|  | OFS | Network | **1.27** | **(1.04, 1.56)** | Very Low |
| FDI | ISC | Network | 0.75 | (0.34, 1.67) | Low |
|  | FXM | Network | 0.79 | (0.34, 1.82) | Low |
|  | IDX | Network | **0.10** | **(0.01, 0.97)** | Low |
|  | SGC | Network | 0.36 | (0.03, 4.42) | Low |
|  | OFS | Network | 0.7 | (0.33, 1.50) | Very Low |
| ISC | FXM | Network | 1.05 | (0.83, 1.34) | Low |
|  | IDX | Network | 0.14 | (0.02, 1.11) | Low |
|  | SGC | Network | 0.47 | (0.04, 5.16) | Low |
|  | OFS | Network | 0.94 | (0.70, 1.25) | Low |
| FXM | IDX | Network | 0.13 | (0.02, 1.07) | Low |
|  | SGC | Network | 0.45 | (0.04, 4.96) | Low |
|  | OFS | Network | 0.89 | (0.61, 1.30) | Low |
| IDX | SGC | Network | 3.41 | (0.72, 16.04) | Low |
|  | OFS | Network | 6.73 | (0.82, 54.91) | Low |
| SGC | OFS | Network | 1.97 | (0.18, 21.85) | Low |

**Ranking -** Serious Adverse Event - Renal Analysis

**Legend:** CAI - Carbonil iron tablet, CTL – Control, FAS - Ferrous ascorbate, FCM - Ferric carboxymaltose, FDI - Ferric derisomaltose, FFM - Ferrous fumarate, FSC - Ferrous succinate, FXM – Ferumoxytol, IDX - Iron dextran, ISC - Iron sucrose, ISFG - Iron sucrose or sodium ferric gluconate, IVSC - Intravenous Standard-of-care, OFM - Oral ferric maltol, OFS - Oral Ferrous Sulfate, OLS - Oral lipossomal iron, PCB – Placebo, SCA - Standard of care, SFO - Saccharated ferric oxide, SGC – Sodium ferric gluconate complex, SUI - Sucrosial Iron.

**League Table -** Serious Adverse Event - Renal Analysis

| **Intravenous agent** | **FCM** | **FDI** | **FFM** | **FXM** | **IDX** | **ISC** | **ISFG** | **OFS** | **SCA** | **SGC** |
| --- | --- | --- | --- | --- | --- | --- | --- | --- | --- | --- |
| **FCM** | FCM | . | . | . | . | . | 0,37  (0.17, 0.83) | 1,27  (1.04, 1.56) | 0,4  (0.19, 0.85) | . |
| **FDI** | 1,81  (0.82, 3.98) | FDI | . | . | . | 1,99  (0.22,17.76) | . | 0,62  (0.27, 1.38) | . | . |
| **FFM** | 1,47  (0.65, 3.32) | 0,81  (0.27, 2.40) | FFM | 0,97  (0.49, 1.95) | . | . | . | . | . | . |
| **FXM** | 1,43  (0.93, 2.20) | 0,79  (0.34, 1.82) | 0,97  (0.49, 1.95) | FXM | . | 0,95  (0.75, 1.21) | . | . | . | . |
| **IDX** | 0,19  (0.02, 1.56) | 0,1  (0.01, 0.97) | 0,13  (0.01, 1.17) | 0,13  (0.02, 1.07) | IDX | 7,19  (0.90,57.47) | . | . | . | 3,41  (0.72,16.04) |
| **ISC** | 1,36  (0.95, 1.94) | 0,75  (0.34, 1.67) | 0,93  (0.44, 1.93) | 0,95  (0.75, 1.21) | 7,19  (0.90,57.47) | ISC | . | 0,95  (0.71, 1.28) | . | 0,47  (0.04, 5.16) |
| **ISFG** | 0,37  (0.17, 0.83) | 0,2  (0.07, 0.63) | 0,25  (0.08, 0.79) | 0,26 | 1,96  (0.21,18.73) | 0,27  (0.11, 0.66) | ISFG | . | 1,08  (0.58, 2.00) | . |
| **OFS** | 1,27  (1.04, 1.56) | 0,7  (0.33, 1.50) | 0,87  (0.39, 1.91) | 0,89  (0.61, 1.30) | 6,73  (0.82,54.90) | 0,94  (0.70, 1.25) | 3,43  (1.50, 7.86) | OFS | . | . |
| **SCA** | 0,4  (0.19, 0.85) | 0,22  (0.07, 0.65) | 0,27  (0.09, 0.82) | 0,28  (0.12, 0.66) | 2,11  (0.22,19.80) | 0,29  (0.13, 0.67) | 1,08  (0.58, 2.00) | 0,31  (0.14, 0.68) | SCA | . |
| **SGC** | 0,64  (0.06, 7.20) | 0,36  (0.03, 4.42) | 0,44  (0.04, 5.33) | 0,45  (0.04, 4.96) | 3,41  (0.72,16.04) | 0,47  (0.04, 5.16) | 1,74  (0.14,22.10) | 0,51  (0.05, 5.61) | 1,62  (0.13,20.22) | SGC |

**Legend:** CAI - Carbonil iron tablet, CTL – Control, FAS - Ferrous ascorbate, FCM - Ferric carboxymaltose, FDI - Ferric derisomaltose, FFM - Ferrous fumarate, FSC - Ferrous succinate, FXM – Ferumoxytol, IDX - Iron dextran, ISC - Iron sucrose, ISFG - Iron sucrose or sodium ferric gluconate, IVSC - Intravenous Standard-of-care, OFM - Oral ferric maltol, OFS - Oral Ferrous Sulfate, OLS - Oral lipossomal iron, PCB – Placebo, SCA - Standard of care, SFO - Saccharated ferric oxide, SGC – Sodium ferric gluconate complex, SUI - Sucrosial Iron.

# Supplement 23 - Results to Serious Adverse Event - GI Analysis

| **Intravenousagent** | **Comparator** | **Estimative** | **MD** | **95% CI** | **Certainty of evidence** |
| --- | --- | --- | --- | --- | --- |
| FCM | FDI | Network | 1.25 | (0.43, 3.62) | Low |
|  | ISC | Network | 2.94 | (0.12, 71.78) | Very Low |
|  | OFS | Network | 5.99 | (0.98, 36.47) | Very Low |
| FDI | ISC | Network | 2.35 | (0.08, 68.37) | Very Low |
|  | OFS | Network | 4.80 | (0.87, 26.49) | Very Low |
| ISC | OFS | Network | 2.04 | (0.05, 80.05) | Very Low |

**Ranking -** Serious Adverse Event - GI Analysis

**League Table -** Serious Adverse Event - GI Analysis

| **Intravenous agent** | **FCM** | **FDI** | **ISC** | **OFM** | **OFS** |
| --- | --- | --- | --- | --- | --- |
| **FCM** | FCM | 1.18  (0.38, 3.60) | 2.94  (0.12, 71.78) | 0.35  (0.12, 1.06) | 8.77  (0.52,148.42) |
| **FDI** | 1.25  (0.43, 3.62) | FDI | . | . | 3.91  (0.50, 30.87) |
| **ISC** | 2.94  (0.12, 71.78) | 2.35  (0.08, 68.37) | ISC | . | . |
| **OFM** | 0.35  (0.12, 1.06) | 0.28  (0.06, 1.31) | 0.12  (0.00, 3.53) | OFM | . |
| **OFS** | 5.99  (0.98, 36.47) | 4.80  (0.87, 26.49) | 2.04  (0.05, 80.04) | 16.97  (2.04, 141.00) | OFS |

**Legend:** CAI - Carbonil iron tablet, CTL – Control, FAS - Ferrous ascorbate, FCM - Ferric carboxymaltose, FDI - Ferric derisomaltose, FFM - Ferrous fumarate, FSC - Ferrous succinate, FXM – Ferumoxytol, IDX - Iron dextran, ISC - Iron sucrose, ISFG - Iron sucrose or sodium ferric gluconate, IVSC - Intravenous Standard-of-care, OFM - Oral ferric maltol, OFS - Oral Ferrous Sulfate, OLS - Oral lipossomal iron, PCB – Placebo, SCA - Standard of care, SFO - Saccharated ferric oxide, SGC – Sodium ferric gluconate complex, SUI - Sucrosial Iron.

# Supplement 24 - Results to Hypophosphatemia - Overall Analysis

| **Intravenous**  **agent** | **Comparator** | **Estimative** | **RR** | **95% CI** | **Certainty of evidence** |
| --- | --- | --- | --- | --- | --- |
| FCM | FDI | Network | **4.97** | **(1.75, 14.17)** | Moderate |
|  | ISC | Network | **8.33** | **(3.01, 23.06)** | Moderate |
|  | FXM | Network | **49.92** | **(7.51, 331.86)** | Moderate |
|  | OFS | Network | **13.45** | **(1.22, 147.84)** | Moderate |
| FDI | ISC | Network | 1.68 | (0.60, 4.68) | Moderate |
|  | FXM | Network | **10.03** | **(1.15, 87.40)** | Moderate |
|  | OFS | Network | 2.7 | (0.25, 28.71) | Moderate |
| ISC | FXM | Network | 5.99 | (0.70, 51.44) | Moderate |
|  | OFS | Network | 1.61 | (0.13, 19.32) | Moderate |
| FXM | OFS | Network | 0.27 | (0.01, 5.72) | Moderate |

**Ranking – hypophosphatemia – Overall population**

**Legend:** CAI - Carbonil iron tablet, CTL – Control, FAS - Ferrous ascorbate, FCM - Ferric carboxymaltose, FDI - Ferric derisomaltose, FFM - Ferrous fumarate, FSC - Ferrous succinate, FXM – Ferumoxytol, IDX - Iron dextran, ISC - Iron sucrose, ISFG - Iron sucrose or sodium ferric gluconate, IVSC - Intravenous Standard-of-care, OFM - Oral ferric maltol, OFS - Oral Ferrous Sulfate, OLS - Oral lipossomal iron, PCB – Placebo, SCA - Standard of care, SFO - Saccharated ferric oxide, SGC – Sodium ferric gluconate complex, SUI - Sucrosial Iron.

**League Table - hypophosphatemia – Overall population**

| **Intravenous agent** | **CAI** | **FCM** | **FDI** | **FXM** | **ISC** | **IVSC** | **OFS** | **PCB** |
| --- | --- | --- | --- | --- | --- | --- | --- | --- |
| **CAI** | CAI | 0.05  (0.00, 0.95) | . | . | . | . | . | . |
| **FCM** | 0.05  (0.00, 0.95) | FCM | 8.17  (2.10, 31.75) | 49.92  (7.51,331.85) | 5.30  (1.55, 18.16) | 28.08  (0.82,967.16) | 19.54  (0.56,686.24) | 1.62  (0.18, 14.17) |
| **FDI** | 0.26  (0.01, 5.69) | 4.97  (1.75, 14.17) | FDI | . | 2.67  (0.76, 9.35) | . | 2.05  (0.10, 43.76) | . |
| **FXM** | 2.59  (0.08, 83.49) | 49.92  (7.51, 331.85) | 10.03  (1.15, 87.39) | FXM | . | . | . | . |
| **ISC** | 0.43  (0.02, 9.44) | 8.33  (3.01, 23.06) | 1.68  (0.60, 4.68) | 0.17  (0.02, 1.43) | ISC | . | . | . |
| **IVSC** | 1.46  (0.01, 142.50) | 28.08  (0.82, 967.16) | 5.65  (0.14, 226.25) | 0.56  (0.01, 31.16) | 3.37  (0.08, 133.94) | IVSC | . | . |
| **OFS** | 0.70  (0.02, 30.31) | 13.45  (1.22, 147.84) | 2.70  (0.25, 28.71) | 0.27  (0.01, 5.72) | 1.61  (0.13, 19.32) | 0.48  (0.01, 34.41) | OFS | . |
| **PCB** | 0.08  (0.00, 3.17) | 1.62  (0.18, 14.17) | 0.33  (0.03, 3.62) | 0.03  (0.00, 0.58) | 0.19  (0.02, 2.13) | 0.06  (0.00, 3.66) | 0.12  (0.00, 3.05) | PCB |

**Legend:** CAI - Carbonil iron tablet, CTL – Control, FAS - Ferrous ascorbate, FCM - Ferric carboxymaltose, FDI - Ferric derisomaltose, FFM - Ferrous fumarate, FSC - Ferrous succinate, FXM – Ferumoxytol, IDX - Iron dextran, IQR -interquartile range, ISC - Iron sucrose, ISFG - Iron sucrose or sodium ferric gluconate, IVSC - Intravenous Standard-of-care, OFM - Oral ferric maltol, OFS - Oral Ferrous Sulfate, OLS - Oral lipossomal iron, PCB – Placebo, SCA - Standard of care, SFO - Saccharated ferric oxide, SGC – Sodium ferric gluconate complex, SUI - Sucrosial Iron.

# Supplement 25 - Results to Hypophosphatemia - Renal Analysis

| **Intravenous agent** | **Comparator** | **Estimative** | **MD** | **95% CI** | **Certainty of evidence** |
| --- | --- | --- | --- | --- | --- |
| FDI | ISC | **Network** | **3.79** | **(1.81, 7.95)** | Moderate |
|  | OFS | **Network** | **3.79** | **(1.81, 7.95)** | Moderate |
| ISC | OFS | Network | 0.54 | (0.05, 5.41) | Low |

**Ranking -** Hypophosphatemia - Renal Analysis

**League Table -** Hypophosphatemia - Renal Analysis

| **Intravenous agent** | **FDI** | **ISC** | **OFS** |
| --- | --- | --- | --- |
| **FDI** | FDI | 3.79  (1.81, 7.95) | 2.05  (0.23,18.16) |
| **ISC** | 3.79  (1.81, 7.95) | ISC | . |
| **OFS** | 2.05  (0.23,18.16) | 0.54  (0.05, 5.41) | OFS |

**Legend:** FDI - Ferric derisomaltose, ISC - Iron sucrose, OFS - Oral Ferrous Sulfate.

# Supplement 26 - Results to Hypophosphatemia - GI Analysis

| **Intravenous agent** | **Comparator** | **Estimative** | **MD** | **95% CI** | **Certainty of evidence** |
| --- | --- | --- | --- | --- | --- |
| FCM | FDI | Network | **4.73** | **(2.16, 10.37)** | Low |
|  | ISC | Network | **22.50** | **(11.81, 42.89)** | Low |
| FDI | ISC | Network | 4.75 | (1.72, 13.12) | Low |

**Ranking -** Hypophosphatemia - GI Analysis

**League Table -** Hypophosphatemia - GI Analysis

| **Treatment** | **FCM** | **FDI** | **ISC** |
| --- | --- | --- | --- |
| **FCM** | FCM | 4.73  (2.16,10.37) | 22.50  (11.81,42.89) |
| **FDI** | 4.73  (2.16,10.37) | FDI | . |
| **ISC** | 22.50  (11.81,42.89) | 4.75  (1.72,13.12) | ISC |

**Legend:** FCM - Ferric carboxymaltose, FDI - Ferric derisomaltose, ISC - Iron sucrose.
